# Dehydrogenative desaturation-relay via formation of multicenter-stabilized radical intermediates

**DOI:** 10.1038/s41467-017-02381-8

**Published:** 2017-12-22

**Authors:** Yaping Shang, Xiaoming Jie, Krishna Jonnada, Syeda Nahid Zafar, Weiping Su

**Affiliations:** State Key Laboratory of Structural Chemistry, Fujian Institute of Research on the Structure of Matter, Chinese Academy of Sciences, Fuzhou, 350002 China

## Abstract

In organic molecules, the reactivity at the carbon atom next to the functional group is dramatically different from that at other carbon atoms. Herein, we report that a versatile copper-catalyzed method enables successive dehydrogenation or dehydrogenation of ketones, aldehydes, alcohols, α,β-unsaturated diesters, and *N*-heterocycles to furnish stereodefined conjugated dienecarbonyls, polyenecarbonyls, and nitrogen-containing heteroarenes. On the basis of mechanistic studies, the copper-catalyzed successive dehydrogenation process proceeds via the initial α,β-desaturation followed by further dehydrogenative desaturation of the resultant enone intermediate, demonstrating that the reactivity at α-carbon is transferred through carbon–carbon double bond or longer π-system to the carbon atoms at the positions γ, ε, and η to carbonyl groups. The dehydrogenative desaturation–relay is ascribed to the formation of an unusual radical intermediate stabilized by 5- or 7,- or 9-center π-systems. The discovery of successive dehydrogenation may open the door to functionalizations of the positions distant from functional groups in organic molecules.

## Abstract (web summary)

Synthesis of valuable polyene molecules under mild conditions and with good functional groups tolerance is of paramount importance. Here, the authors show the versatile copper-catalyzed successive dehydrogenation of a variety of organic substrates affording highly conjugated unsaturated products.

## Introduction

Transition-metal-catalyzed dehydrogenation reactions are emerging as a powerful tool for molecular modifications^1, 2^. In this context, considerable efforts have been devoted to develop catalytic dehydrogenation of ketones and aldehydes^3–5^, as well as esters, nitriles, and amides^6–8^ since the obtained α,β-unsaturated carbonyls and nitriles are readily manipulated by numerous methods to access pharmaceuticals and other biologically active compounds. On the other hand, the oxidative methods for dehydrogenative desaturation via generation of radical intermediates have been developed^9–13^. Recently, the methods that perform the dehydrogenative desaturation in tandem with the reaction of unsaturated compounds have been established by our group^14, 15^ and others^16–19^ to access the cascade approaches to β-functionalization of saturated carbonyl compounds. During this investigation, we disclosed that a copper-catalyzed reaction of ketones with 2,2,6,6-tetramethylpiperidine-*N*-oxyl (TEMPO)^20^ generated α,β-unsaturated ketones^15^, the reaction pathway of which involved formation of carbonyl α-radical intermediate, combination of TEMPO with this α-radical intermediate^21^, and final elimination of 2,2,6,6-tetramethyl-*N*-hydroxypiperidine (TEMPOH) from the resulting α-TEMPO ketone adduct to release enone product.

**Fig. 1:**
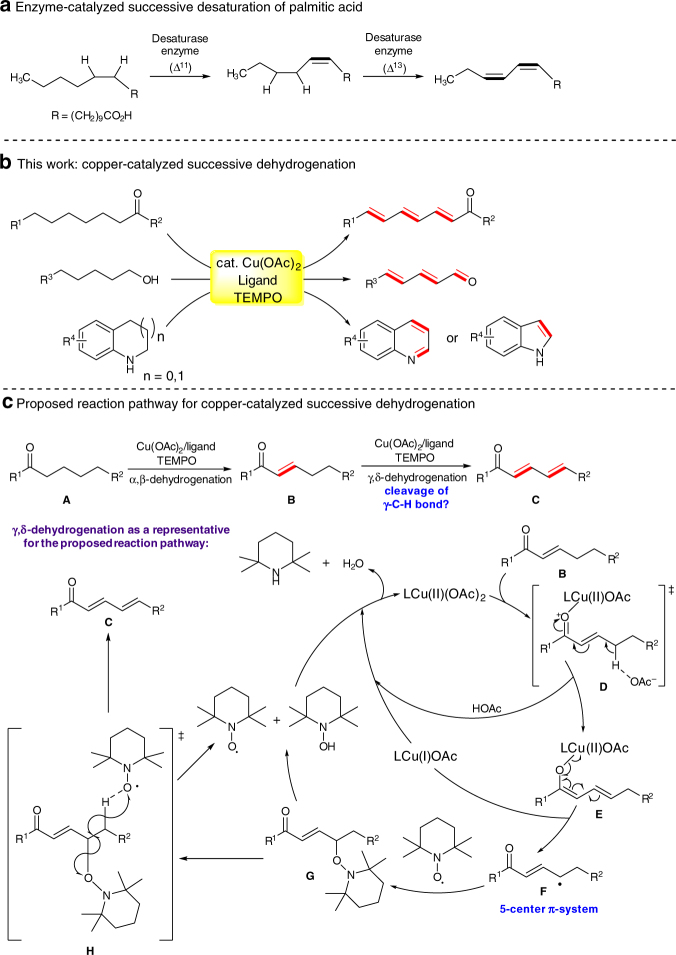
Design of copper-catalyzed successive dehydrogenation. **a** Enzymatic consecutive desaturation in nature. **b** Successive dehydrogenation of carbonyl compounds and *N*-heterocycles using copper/TEMPO system (this work). **c** Description of the proposed mechanism for copper-catalyzed successive dehydrogenation

In nature, enzyme-catalyzed consecutive dehydrogenation of fatty acids (Fig. 1a) suggests an efficient approach to synthesis of multiple conjugated carbon chains in a step- and atom-economical fashion^22^. However, the laboratory substitute of this biosynthesis process remains elusive presumably due to the challenges posed by the poor chemical selectivity of reactive acyclic monoene intermediates.

Herein, we report that a copper catalyst supported by bidentate nitrogen-containing ligand, in combination with use of TEMPO as an oxidant, is capable of affecting consecutive dehydrogenation or dehydrogenation of various saturated ketones, aldehydes, alcohols, α,β-unsaturated diesters, and *N*-heterocycles to construct diverse stereodefined dienecarbonyls, polyenecarbonyls, and nitrogen-containing heteroarenes in generally good yields. As revealed by mechanistic studies, this copper-catalyzed successive dehydrogenative desaturation is a stepwise process that starts by α,β-desaturation, illustrating that the carbonyl-induced dehydrogenation reactivity of the α-carbon can be transferred through carbon–carbon double bond or a longer π-system to the carbons at the positions γ, ε, and η to carbonyls. The dehydrogenative desaturation–relay is ascribed to formation of the radical intermediates stabilized by 5-, or 7-, or 9-center π-systems. As such, this copper-catalyzed successive dehydrogenation reaction mechanistically differs from the recently pioneered dehydroaromatization of cyclohexanones^23^, cyclohexenes^24^, and alkanes^25^ in which dehydrogenation reactions occur at the identical carbon atoms relative to functional groups.

## Results

### Reaction design

Our previous discovery of a copper-catalyzed dehydrogenative α,β-desaturation of ketone to enone led us to question whether a successive dehydrogenative desaturation of ketones bearing longer acyclic aliphatic chains might be possible, which resulted in the establishment of a versatile catalytic method for successive dehydrogenation of diverse saturated substrates (Fig. 1b). A prospective reaction pathway for Cu-catalyzed successive dehydrogenation of carbonyls is outlined in Fig. 1c, in which formation of γ-enone radical intermediate **F** is the critical step. We hypothesized that the γ-C(*sp* ^3^)–H bond of enone could be polarized toward deprotonation, as illustrated in transition state **D** and generation of a Cu(II)–dienolate complex intermediate **E** ^26^, when carbonyls, in combination with metal catalyst, could have a sufficient polarizing effect on the γ-C(*sp* ^3^)–H bond through C–C double bond. In intermediate **E**, the homolysis of Cu(II)–O bond could occur to yield γ-enone radical intermediate **F** and release Cu(I) catalyst, in which intermediate **F** could be stabilized by a delocalization system of five π-electrons^27^. Coupling of the γ-enone radical intermediate **F** with TEMPO produced intermediate **G** and subsequent β-elimination via intermediate **H** would ultimately afford dienone product **C**. In analogy to the proposed dienone formation, polyenone structural motif would be accessible if the polarizing effect of carbonyl group could be delivered to the remote C(*sp* ^3^)–H bond by two or more than two conjugated carbon–carbon double bonds so as to activate this C–H bond for formation of a radical distant from carbonyl.

In light of our proposed reaction pathway, the successive dehydrogenative desaturations of ketone would strongly rely on the polarizing effect that carbonyls exert on C(*sp* ^3^)–H bonds through a carbon–carbon double bond, or conjugated diene or triene subunits. However, such an effect is rapidly weakened as the separation between carbonyl group and the targeted C(*sp* ^3^)–H bond increases. We envisioned that the effect of carbonyl groups on these C(*sp* ^3^)–H bonds could be enhanced by a metal complex catalyst that could serve as an electron-withdrawing Lewis acid to further polarize carbonyl groups via formation of Lewis acid–base adduct. Accordingly, the metal catalyst would be required to play a two-fold role: strengthening the ability of carbonyl group to polarize the remote C(*sp* ^3^)–H bond, and generating a radical intermediate stabilized by a conjugated π-system. We speculated that the choice of a proper ligand would lead to the optimal metal catalyst for the successive dehydrogenation process because an auxiliary ligand is able to simultaneously modulate Lewis acidity and redox nature of a metal catalyst.

Our exploration of this kind of reaction is inspired by the pioneering work on the photocatalytic direct β-arylation of saturated carbonyls via β-enamine radical intermediate formation^27^, carbene-catalyzed β-functionalization reaction of esters^28^, and some of allylic C–H functionalization reactions^29^, of which the key reaction intermediates are all stabilized by conjugated systems. The delocalized mutiple-center radical intermediate proposed herein, if accessible, would provide an entry into achieving functionalizations of the positions distant from carbonyl groups^30, 31^.

### Development of copper-catalyzed successive dehydrogenation

**Fig. 2:**
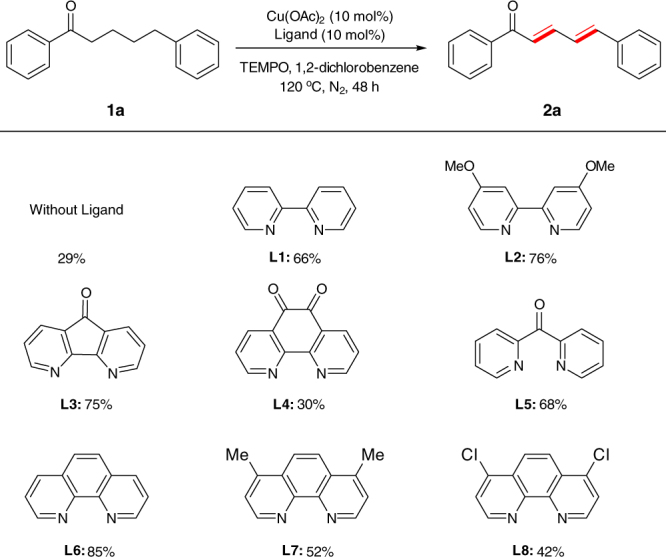
Ligand optimization for copper-catalyzed successive dehydrogenation of ketone. Reaction conditions: ketone (0.2 mmol), Cu(OAc)_2_ (10 mol%), ligand (10 mol%), TEMPO (2.0 equiv.), 1,2-dichlorobenzene (1.0 mL), 120 °C, N_2_, 48 h. Yields were determined by gas chromatography analysis using *n*-dodecane as an internal standard

With these considerations in mind, we initially investigated the successive dehydrogenation of 1,5-diphenylpentan-1-one (**1a**) with TEMPO as an oxidant, Cu(OAc)_2_ as a catalyst, and a variety of *N*-containing bidentate ligands (Fig. 2). Gratifyingly, we found that Cu(OAc)_2_ could catalyze the desired successive dehydrogenation in the absence of any ligand to afford the stereodefined (*E*,*E*)-diene product in 29% yield. Introducing bidentate ligands such as 2,2ʹ-bipyridne (**L1**) and 4,4ʹ-dimethoxy-2,2ʹ-bipyridine (**L2**) significantly improved the yield of the desired product. Of the ligands examined, unsubstituted phenanthroline (**L6**) gives the best yield (85% yield) for the targeted product. Other phenanthroline ligands bearing electron-withdrawing (**L8**) or electron-donating (**L2**) substituents are inferior to unsubstituted phenanthroline, reflecting that the effect of a ligand on the reaction outcome is complicated.

### Substrate scope and synthetic applications

**Fig. 3:**
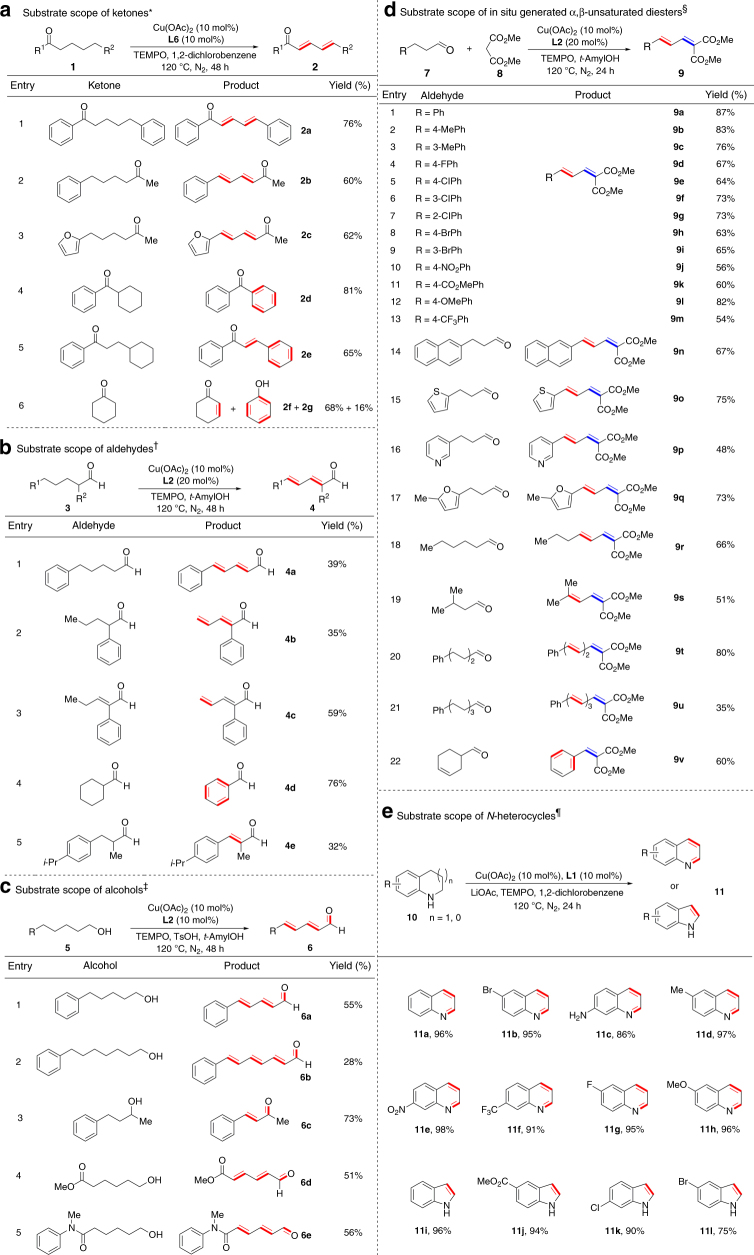
Substrate scope of copper-catalyzed successive dehydrogenation. **a** Scope of ketones. **b** Scope of aldehydes. **c** Scope of alcohols. **d** Scope of α,β-unsaturated diesters. **e** Scope of *N*-heterocycles. ^*^General conditions: ketone (0.2 mmol), Cu(OAc)_2_ (10 mol%), **L6** (10 mol%), TEMPO (2.0 equiv.), 1,2-dichlorobenzene (1.0 mL), 120 °C, N_2_, 48 h. ^†^General conditions: aldehyde (0.2 mmol), Cu(OAc)_2_ (10 mol%), **L2** (20 mol%), TEMPO (2.0 equiv.), *tert*-amyl alcohol (1.0 mL), 120 °C, N_2_, 48 h. ^‡^General conditions: alcohol (0.4 mmol), Cu(OAc)_2_ (10 mol%), **L2** (10 mol%), TEMPO (3.0 equiv.), TsOH (10 mol%), *tert*-amyl alcohol (3.0 mL), 120 °C, N_2_, 48 h. ^§^General conditions: aldehyde (0.2 mmol), dimethyl malonate (0.5 mmol), Cu(OAc)_2_ (10 mol%), **L2** (20 mol%), TEMPO (1.0 equiv.), *tert*-amyl alcohol (1.0 mL), 120 °C, N_2_, 24 h. ^¶^General conditions: saturated *N*-heterocycle (0.5 mmol), Cu(OAc)_2_ (10 mol%), **L1** (10 mol%), TEMPO (2.0 equiv.), LiOAc (1.0 equiv), 1,2-dichlorobenzene (1.0 mL), 100–120 °C, N_2_, 48 h. Double bonds generated via dehydrogenative desaturation are denoted by red lines, and double bonds generated via Knoevenagel condensation are denoted by blue lines. All yields are isolated yields except for **2f**, **2g**, and **4d** in which GC yields are reported. For detailed reaction conditions, please see the Supplementary Methods

This Cu-catalyzed successive dehydrogenation process proved to be general in terms of substrate scope. As shown in Fig. 3a, both alkyl aryl ketone and dialkyl ketone underwent successive dehydrogenation to generate (*E*,*E*)-diene products in good yields (**2a**–**2c**). Cyclohexyl phenyl ketone went through dehydrogenative aromatization to form benzophenone (**2d**), and the cyclohexyl group separated from carbonyl by a (CH_2_)_2_ subunit still exhibited similar reactivity (**2e**). For cyclic substrate, cyclohexanone underwent dehydrogenation to produce a mixture of cyclohexenone (**2f**, 68% yield) and phenol (**2g**, 16% yield). Similarly to ketones, a series of aldehydes could also participate in the successive dehydrogenation (**4a**–**4d**) with different optimized ligands and solvents (Fig. 3b). α-Substituents did not interfere with this dehydrogenation process (**4b**, **4d**, and **4e**), and again, cyclohexanecarboxaldehyde could be converted to benzaldehyde via dehydrogenative aromatization (**4d**). Dehydrogenation of α,β-unsaturated aldehyde to generate diene aldehyde (**4c**) illustrated the potential of this dehydrogenation method in late elaboration of complicated molecules. Besides ketones and aldehydes, both primary and secondary alcohols were also suitable substrates in this transformation (**6a**–**6e**) (Fig. 3c) due to in situ oxidation of alcohols by TEMPO to generate the corresponding aldehydes and ketones^32, 33^. In this transformation, TsOH as an additive may facilitate dissociation of OAc anion to generate the Cu(II)-alkoxide species that is a key intermediate in the Cu(II)/TEMPO-promoted oxidation of alcohols to carbonyls^32^. Gratifyingly, successive dehydrogenation of primary alcohols produced the same products as the reaction of aldehydes but afforded higher yields (**6a**). The successive dehydrogenation process of alcohols was observed to enable generation of four conjugated double bonds from the corresponding saturated alcohol (**6b**), and tolerate ester and amide groups at the other end of carbon chains of alcohols (**6d** and **6e**).

The α,β-unsaturated diesters generated in situ from Knoevenagel condensation of 3-arylpropanals with dimethyl malonates also participated in dehydrogenation to form various diene products in high yields (**9a**–**9q**) in the presence of one equivalent of TEMPO (Fig. 3d). This cascade condensation/dehydrogenation process is compatible with diverse substituents and substitution positions on the phenyl ring of 3-arylpropanals as well as their heterocyclic variants. Such a cascade process was also applicable to the alkyl aldehydes that lack aryl groups at the end of carbon chains (**9r** and **9s**). Multiple successive dehydrogenations were also observed to furnish trienes (**9t**) and even tetraenes (**9u**). The reaction of cyclohex-3-ene-1-carbaldehyde led to dehydrogenative aromatization of cyclohexene moiety (**9v**).

In addition to carbonyl compounds, we were pleased to find that this catalytic dehydrogenation was also capable of delivering aromatic *N*-heterocycles from their saturated counterparts (Fig. 3e)^34–37^ under slightly modified conditions. In this transformation, LiOAc may act as a base for deprotonation of N–H and C–H bonds. Various tetrahydroquinolines bearing bromo, amino, methyl, nitro, trifluoromethyl, fluoro, and methoxy groups were dehydrogenated in excellent yield (**11a**–**11h**). Besides tetrahydroquinolines, NH-free indolines were also suitable substrates to afford various substituted indoles in excellent yield (**11i**–**11l**). These findings not only provide a rare example of copper-catalyzed dehydrogenation of *N*-heterocycles, but also demonstrate again the applicability and generality of our protocol.

**Fig. 4:**
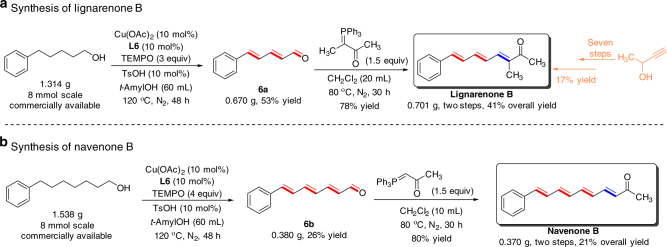
Synthetic application. **a** Synthesis of natural product lignarenone B. **b** Synthesis of natural product navenone B

The ability of the successive dehydrogenation reaction to construct stereodefined dienone or polyenone scaffolds encouraged us to utilize this approach to streamline syntheses of related natural products (Fig. 4). Lignarenone B, an alarm pheromone found in Mediterranean mollusk *Scaphander lignarius* ^38^, possesses a phenyl-conjugated trienone moiety in its molecular framework. The synthesis of lignarenone B has been previously achieved by way of a seven-step sequence starting from but-3-yn-2-ol in 17% overall yield^39^. By using our method for successive dehydrogenation, the facile preparative-scale synthesis of lignarenone B could be achieved in 41% overall yield through a two-step process that consists of the successive dehydrogenation of commercially available 5-phenyl-1-pentanol to (2*E*,4*E*)-5-phenylpenta-2,4-dienal, and subsequent Wittig olefination of dienal to afford lignarenone B. Likewise, navenone B, another alarm pheromone isolated from the opisthobranch *Navanax inermis*, could be obtained in 21% total yield starting from 7-phenylheptan-1-ol. These two examples clearly demonstrate the potential of our approach to simplify syntheses of polyenone scaffolds.

### Mechanistic studies

**Fig. 5:**
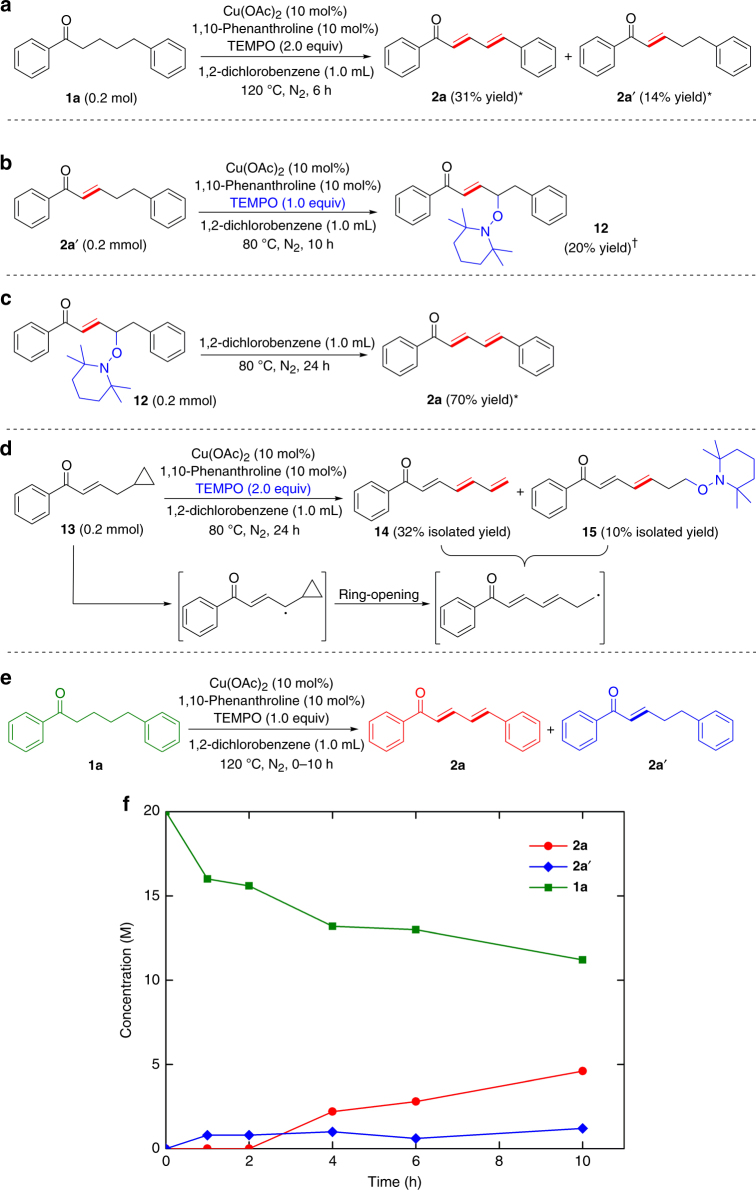
Mechanistic studies. **a** Observation of both dienone and enone products before reaction completion. **b** Identification of γ-TEMPO-substituted enone **12**. **c** Dehydrogenation of γ-TEMPO-substituted enone to dienone. **d** The radical probe experiment. **e** The dehydrogenation of **1a** (green) to **2a** (red). **f** Kinetic time course of the dehydrogenation of **1a** (green) to **2a** (red) and **2a**ʹ (blue) using a lower amount of TEMPO. ^*^GC yields using *n*-dodecane as an internal standard. ^†^Yield determined by ^1^H NMR analysis

During the investigation of successive dehydrogenation of 1,5-diphenyl-1-pentanone (**1a**), we observed that this reaction produced a mixture of (2*E*,4*E*)-1,5-diphenylpenta-2,4-dien-1-one (**2a**) and (*E*)-1,5-diphenylpent-2-en-1-one (**2a**ʹ) after 6 h (Fig. 5a) and that (*E*)-1,5-diphenylpent-2-en-1-one (**2a**ʹ) could be converted to (2*E*,4*E*)-1,5-diphenylpenta-2,4-dien-1-one (**2a**) in 62% yield under standard conditions within 5 h (Supplementary Equation 2). These observations indicate that the successive dehydrogenation is a stepwise process that consists of initial α,β-desaturation and further dehydrogenation of the resulting α,β-unsaturated carbonyls. By analyzing the outcomes of the dehydrogenation reaction of (*E*)-1,5-diphenylpent-2-en-1-one (**2a**ʹ) that was carried out for 10 h with Cu(OAc)_2_/1,10-phenanthroline and one equivalent of TEMPO at 80 °C, we identified γ-TEMPO-substituted enone **12** (Fig. 5b), an intermediate (**G** in Fig. 1c) in the proposed mechanism for the successive dehydrogenation reaction. This γ-TEMPO-substituted enone was presynthesized using other methods, and proved to undergo β-elimination at 80 °C to generate dienone (Fig. 5c). To further support the formation of γ-enone radical intermediate, radical clock experiments have been performed using γ-cyclopropyl enone as a probe (Fig. 5d). γ-Cyclopropyl enone was found to undergo ring opening at 80 °C under conditions similar to successive dehydrogenation to generate trienone in 32% yield and dienone with terminal TEMPO substituent in 10% yield. These experiments provided strong evidence to support that the successive dehydrogenation of ketone to dienone proceeds via γ-enone radical intermediate and γ-TEMPO-substituted enone intermediate. The time course of the successive dehydrogenation reaction of **1a** (Fig. 5e), which was conducted with one equivalent of TEMPO under otherwise identical conditions, shows that the low concentration of **2a**ʹ from α,β-desaturation of **1a** is almost unchanged in the observed reaction period and that the concentration of successive dehydrogenation product **2a** steadily increases. Considering that the rate of α,β-desaturation of ketone proved to be a first-order dependence on the ketone reactant^15^, and that the concentration of **1a** is about 9 times higher than that of **2a**ʹ in the reaction system, the unchanged concentration of **2a**ʹ during the reaction course indicates that the second dehydrogenation step (γ,δ-dehydrogenation of **2a**ʹ to **2a**) is faster than the first dehydrogenation step (α,β-desaturation of **1a** to **2a**ʹ).

## Discussion

We have developed a versatile copper-catalyzed method for successive dehydrogenation of a diverse array of carbonyls, alcohols, and nitrogen-containing heterocycles to furnish dienes, polyenes, and (hetero)arenes in generally good yields with a high level of stereoselectivity, providing a complement to the existing methods for syntheses of dienes and polyenes^40–42^. The achievement of the copper-catalyzed method for high-yielding and stereoselective successive dehydrogenation is attributed to identification of the mild conditions that overcome the problems encountered by the traditional diene synthesis, such as a lack of stereoselectivity and acid-induced instability of diene products^43^. Mechanistic investigations have uncovered that the dehydrogenation reactivity of α-carbon can be delivered, through carbon–carbon double bond or multiple conjugated carbon–carbon double bond, to the saturated carbon atoms distant from carbonyl groups, and that the successive dehydrogenation reaction involves formation of an unusual radical intermediate stabilized by multicenter π-system. In this stepwise successive dehydrogenation process, the γ,δ-dehydrogenation of enone is faster than the initial α,β-dehydrogenation of saturated ketone, presumably because the delocalized five-center γ-enone radical intermediate is more stable than carbonyl α-radical intermediate. Our findings may provide an entry into accessing functionalizations at γ-position of enone, ε-position of conjugated dienecarbonyl, and η-position of conjugated trienecarbonyl, or cascade carbonyl dehydrogenation/functionalization of saturated carbon process by way of formation of radical intermediate stabilized by multicenter π-system.

## Methods

### General

All reactions were conducted under a nitrogen atmosphere in dried glassware with dry solvents. Unless otherwise noted, materials were purchased from commercial suppliers and used directly without further purification. Anhydrous Cu(OAc)_2_ was purchased from Acros. 1,2-dichlorobenzene, DMF, and *tert*-amyl alcohol were distilled over CaH_2_ and stored under nitrogen. Toluene, 1, 4-dioxane, and DME were distilled from Na and stored under nitrogen. ^1^H NMR (400 MHz), ^13^C NMR (100 MHz), and ^19^F NMR (377 MHz) spectra were recorded in CDCl_3_ solutions using a Bruker AVANCE 400 spectrometer. The chemical shift values (*δ*) were calibrated using TMS (0 ppm for ^1^H) and residual undeuterated solvent CHCl_3_ (77.0 ppm for ^13^C). High-resolution mass spectra (HRMS) were performed by the Shanghai Mass Spectrometry Center in Shanghai Institute of Organic Chemistry, Chinese Academy of Sciences (Instrument: Thermo Fisher Scientific LTQ FT Ultra, Operation Mode: DART Positive).

### General procedure for copper-catalyzed successive dehydrogenation of ketones

In a nitrogen-filled glovebox, a 25 mL Schlenk tube equipped with a stir bar was charged with ketone **1** (0.2 mmol), Cu(OAc)_2_ (0.0036 g, 0.02 mmol, 10 mol%), 1,10-phenanthroline (0.0036 g, 0.02 mmol, 10 mol%), and TEMPO (0.0630 g, 0.4 mmol). The tube was fitted with a rubber septum and moved out of the glove box. Then, 1, 2-dichlorobenzene (1.0 mL) was added to the Schlenk tube through the rubber septum using syringes, and then, the septum was replaced with a Teflon screwcap under nitrogen flow. The reaction mixture was stirred at 120 °C for 48 h. Upon cooling to room temperature, the reaction mixture was filtered through a pad of silica gel and washed with 10 mL of ethyl acetate. The filtrate was concentrated under reduced pressure and purified by flash chromatography on silica gel (petroleum ether/EtOAc) to provide the corresponding dienone product **2**.

For the successive dehydrogenation of aldehydes, alcohols, diesters, and *N*-heterocycles, the optimal conditions are modified with different ligands, solvents, as well as the amount of TEMPO. Complete experimental details can be found in the Supplementary Methods.

### Data availability

For NMR spectra of starting materials and products, please see Supplementary Figs. 1–160. All other data are available from the authors upon reasonable request.

## Electronic supplementary material

Supplementary Information

Peer Review File
