# Supplementary material for: Dehydrogenative desaturation-relay via formation of multicenter-stabilized radical intermediates
Source: Nat Commun. 2017 Dec 22;8:2273. doi: 10.1038/s41467-017-02381-8 (PMC5741636; doi:10.1038/s41467-017-02381-8)
Supplement: Supplementary file 1 — Supplementary Information [file 41467_2017_2381_MOESM1_ESM.pdf]

Supplementary Figures

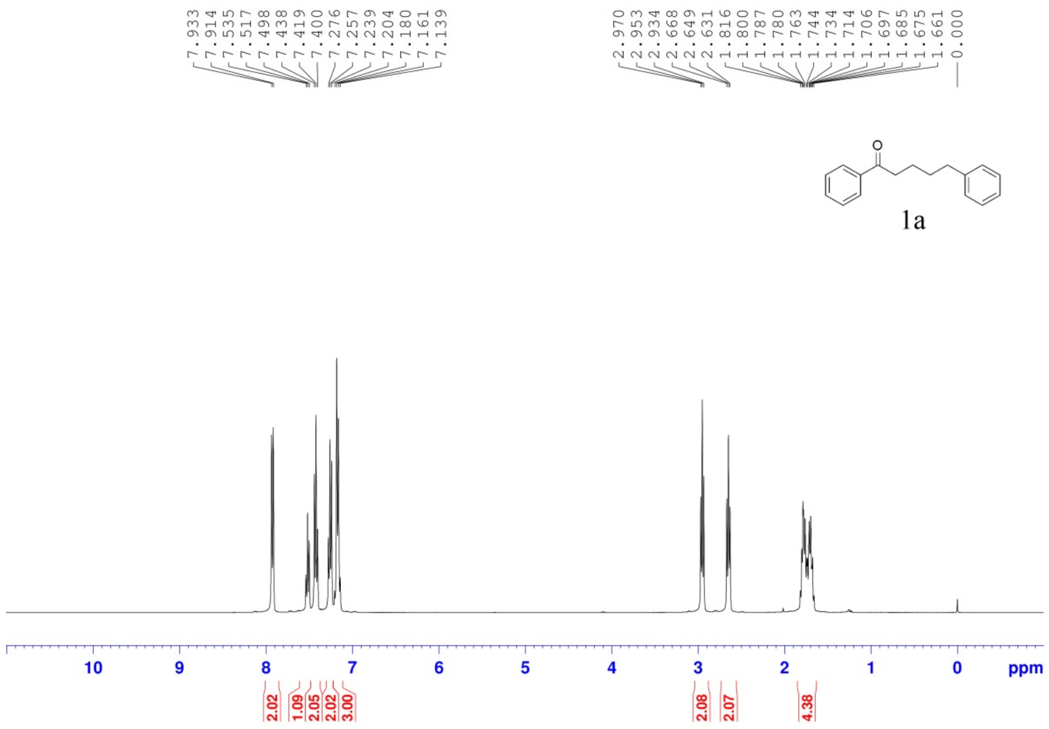

Supplementary Figure 1. ^{1}H NMR spectrum for 1,5-diphenylpentan-1-one (**1a**).

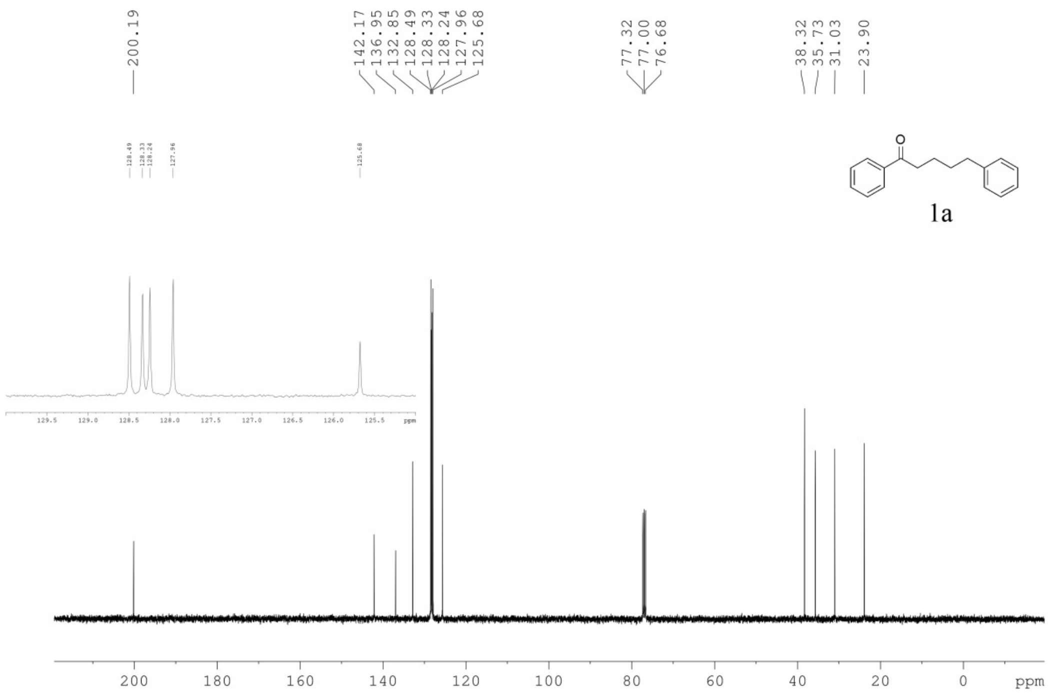

Supplementary Figure 2. ^{13}C NMR spectrum for 1,5-diphenylpentan-1-one (**1a**).

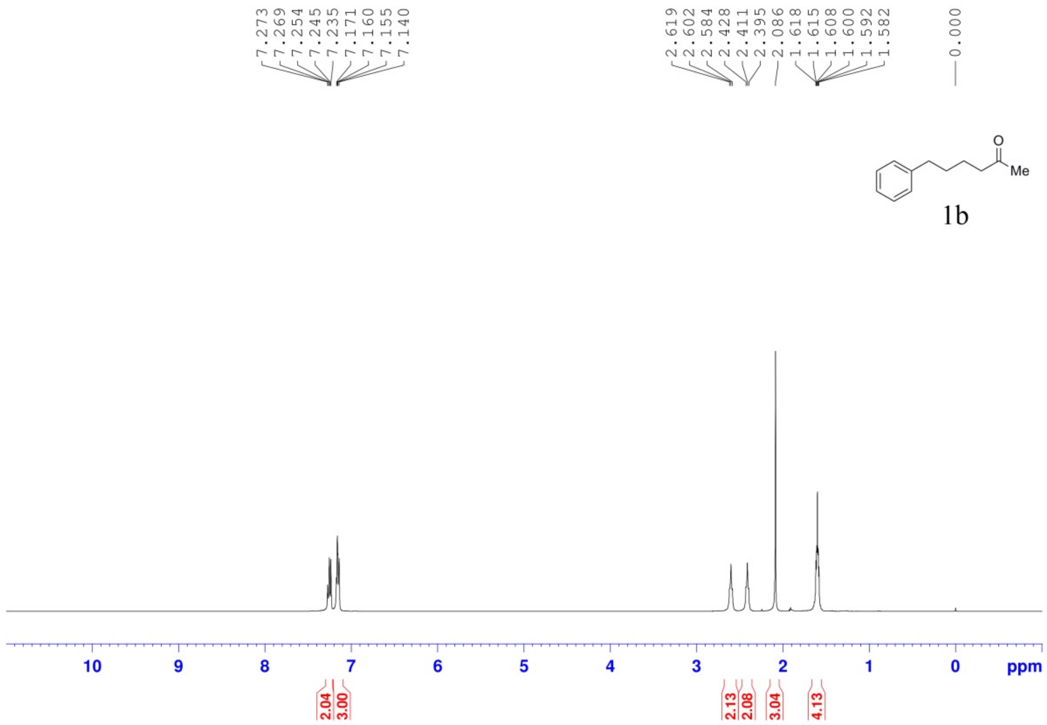

Supplementary Figure 3. ^{1}H NMR spectrum for 6-phenylhexan-2-one (**1b**).

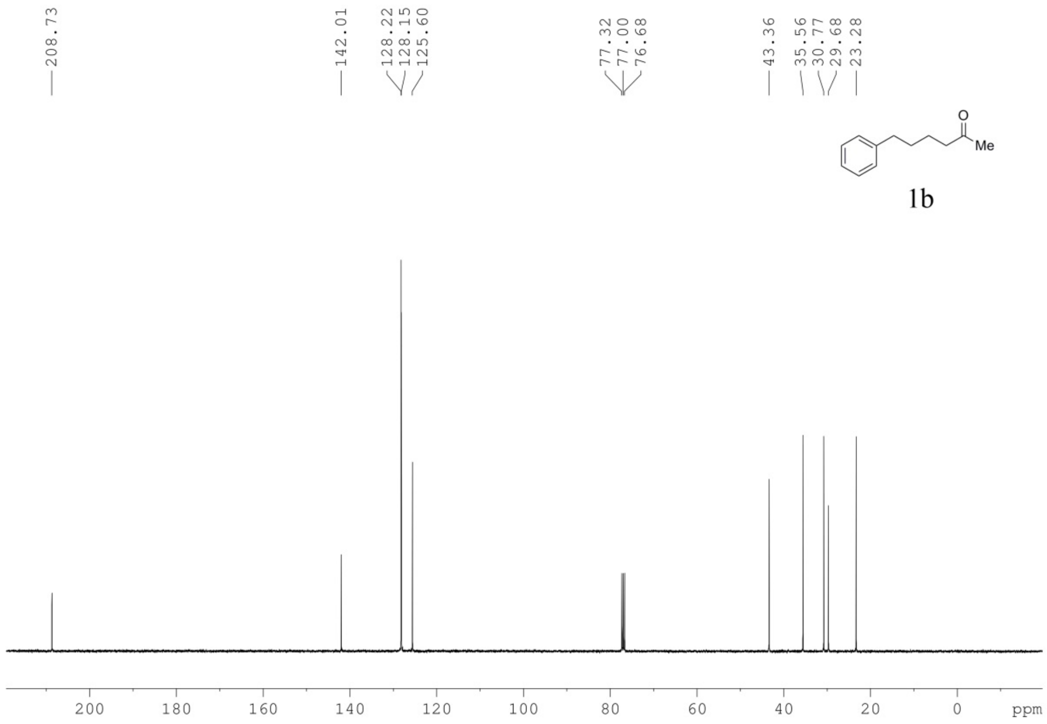

Supplementary Figure 4. ^{13}C NMR spectrum for 6-phenylhexan-2-one (**1b**).

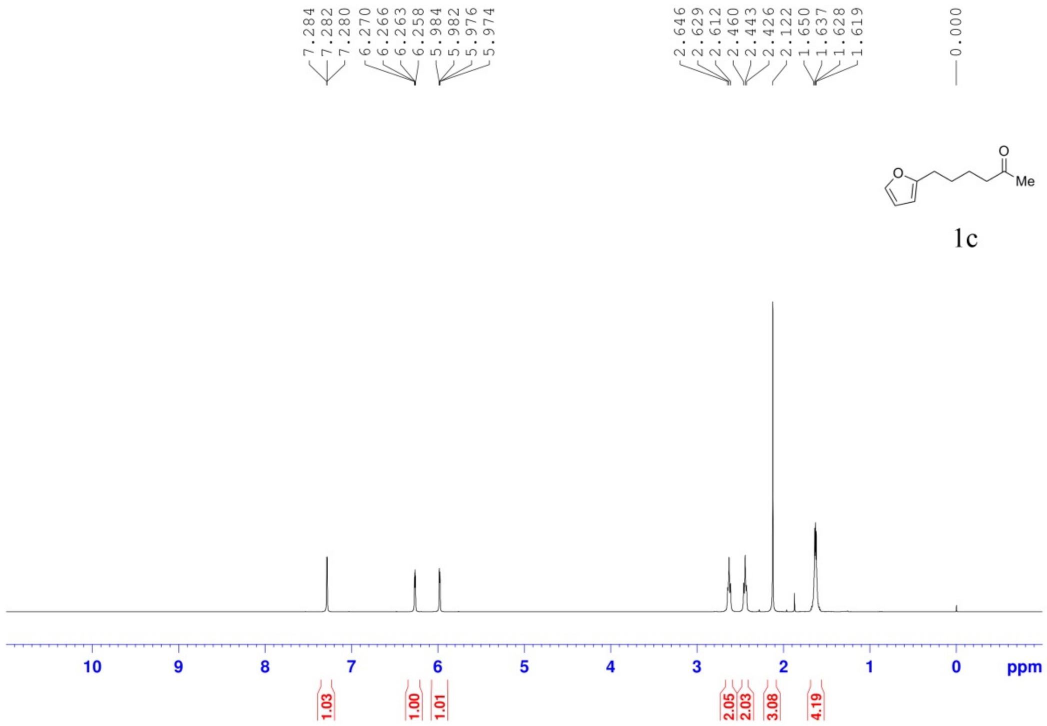

Supplementary Figure 5. ^{1}H NMR spectrum for 6-(furan-2-yl)hexan-2-one (**1c**).

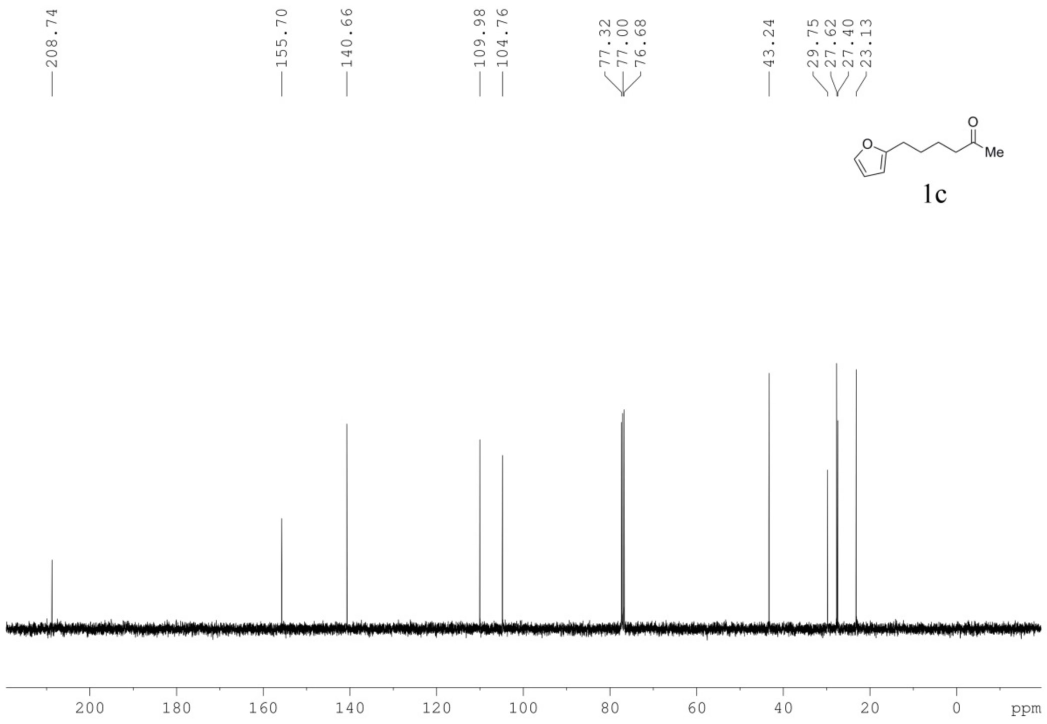

Supplementary Figure 6. ^{13}C NMR spectrum for 6-(furan-2-yl)hexan-2-one (**1c**).

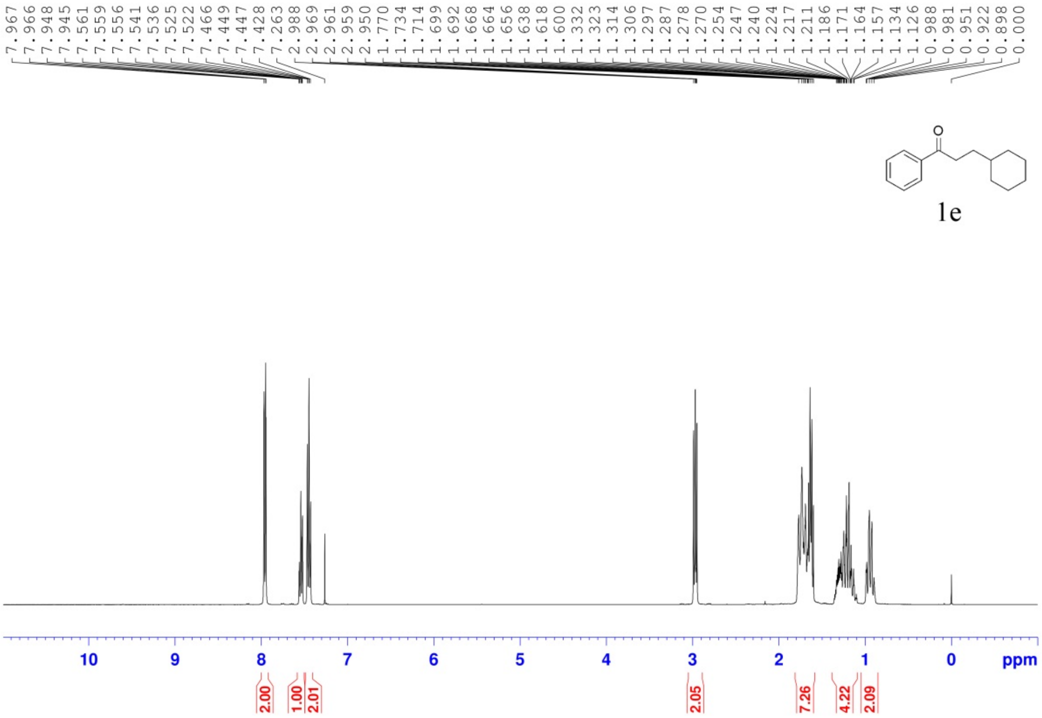

Supplementary Figure 7. ^{1}H NMR spectrum for 3-cyclohexyl-1-phenylpropan-1-one (**1e**).

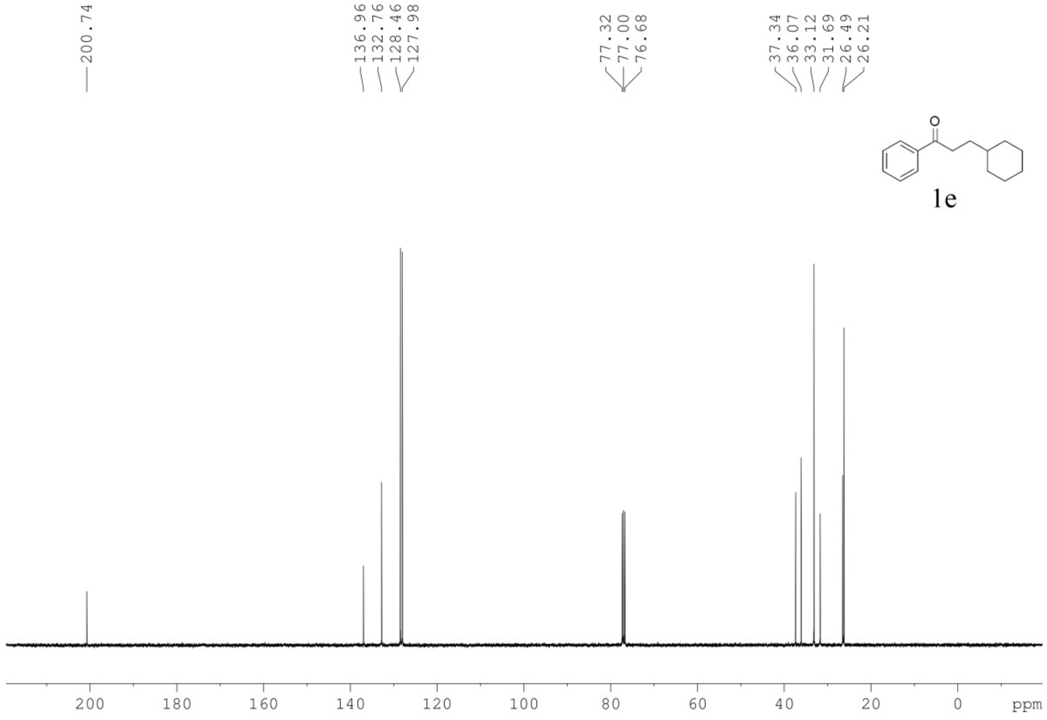

Supplementary Figure 8. ^{13}C NMR spectrum for 3-cyclohexyl-1-phenylpropan-1-one (**1e**).

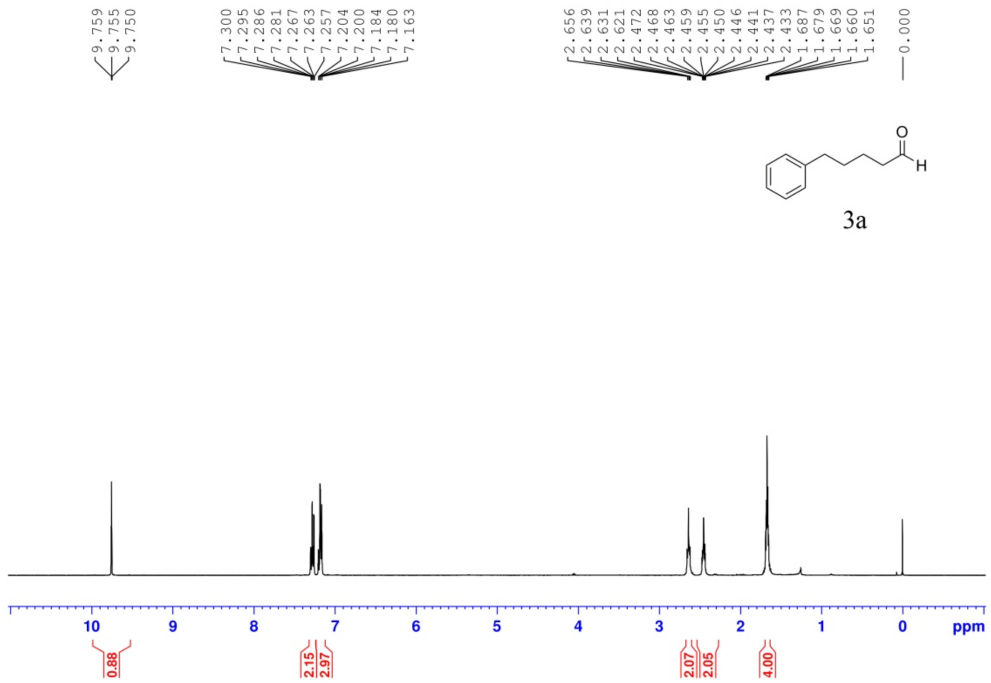

Supplementary Figure 9. ^{1}H NMR spectrum for 5-phenylpentanal (**3a**).

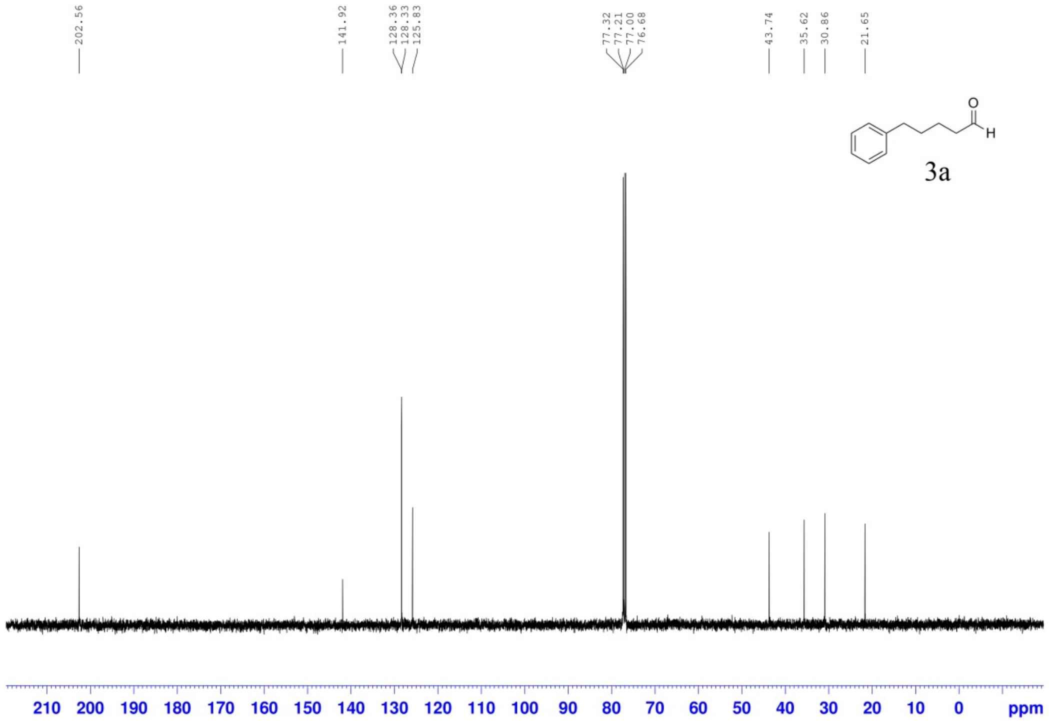

Supplementary Figure 10. ^{13}C NMR spectrum for 5-phenylpentanal (**3a**).

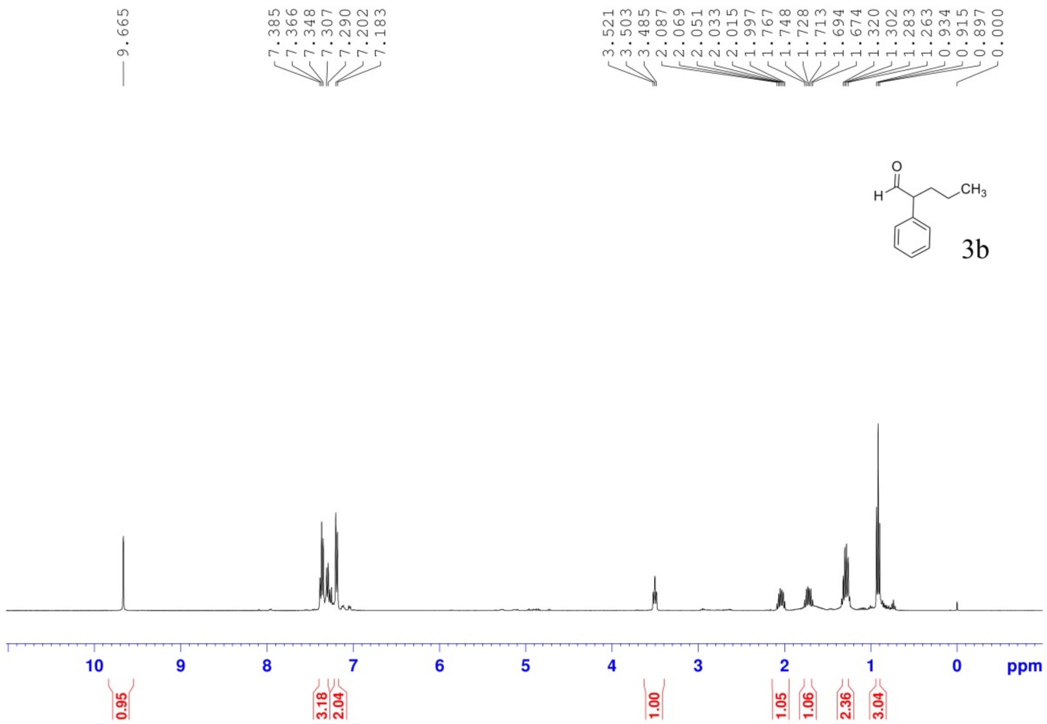

Supplementary Figure 11. ^{1}H NMR spectrum for 2-phenylpentanal (**3b**).

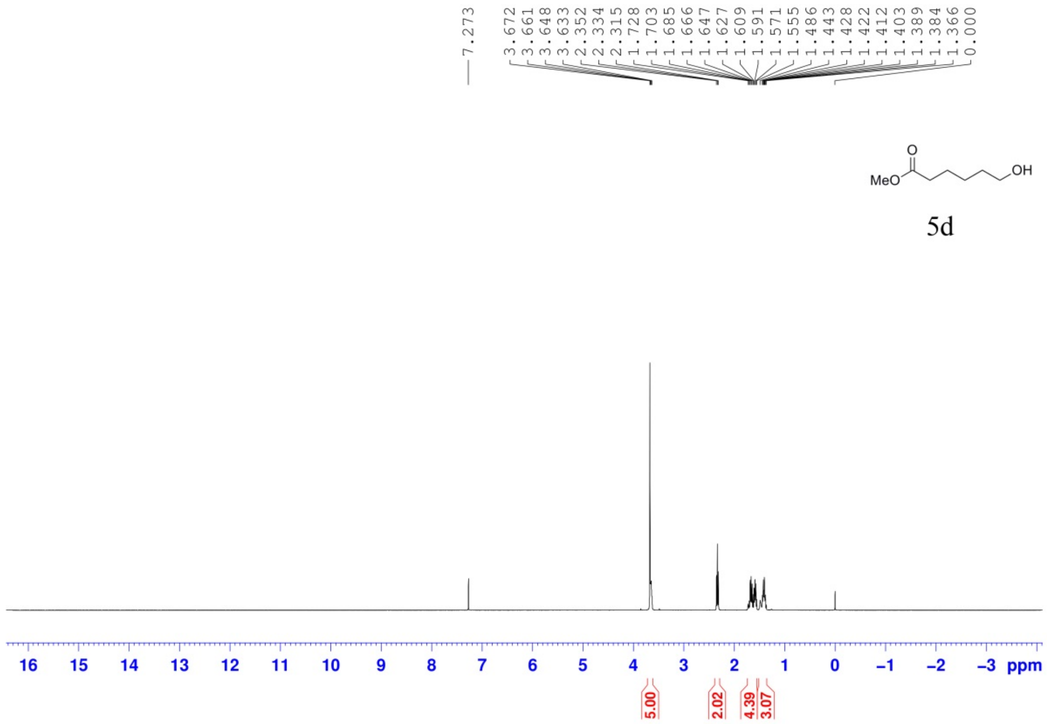

Supplementary Figure 12. ^{1}H NMR spectrum for methyl 6-hydroxyhexanoate (**5d**).

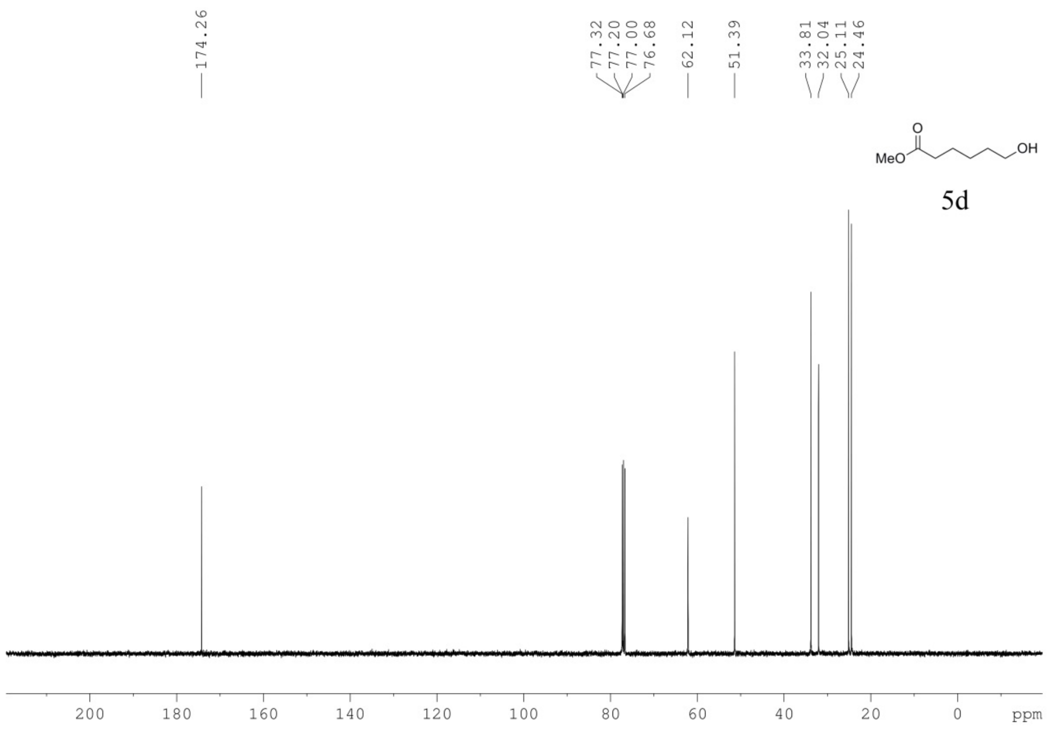

Supplementary Figure 13. ^{13}C NMR spectrum for methyl 6-hydroxyhexanoate (**5d**).

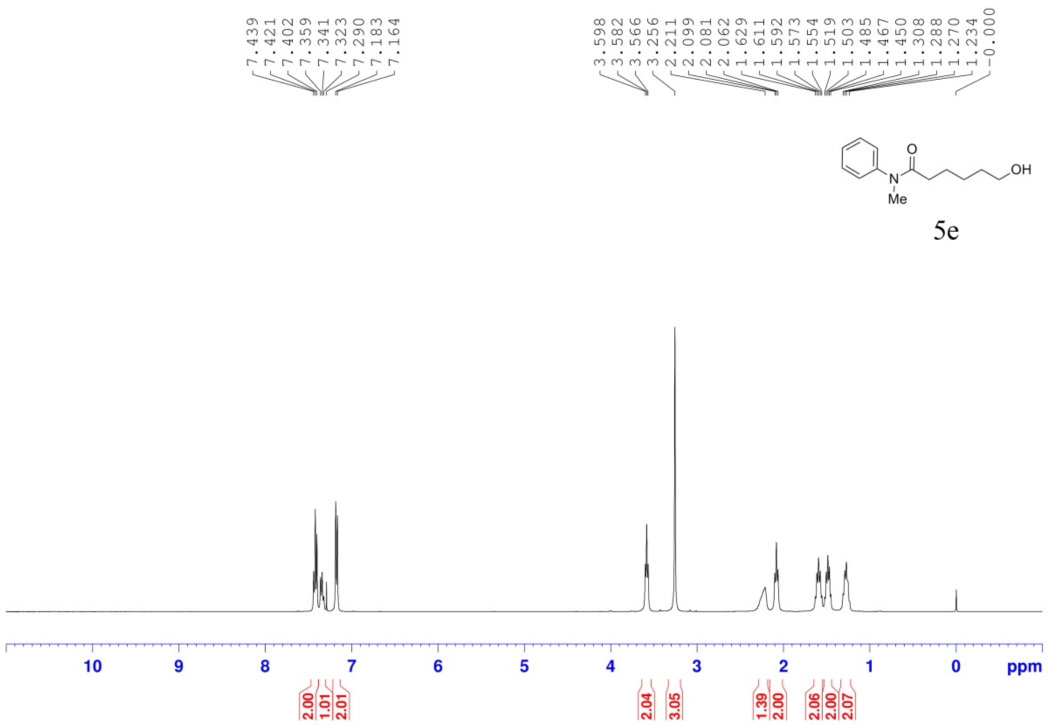

Supplementary Figure 14. ^{1}H NMR spectrum for 6-hydroxy-*N*-methyl-*N*-phenyl hexanamide (**5e**).

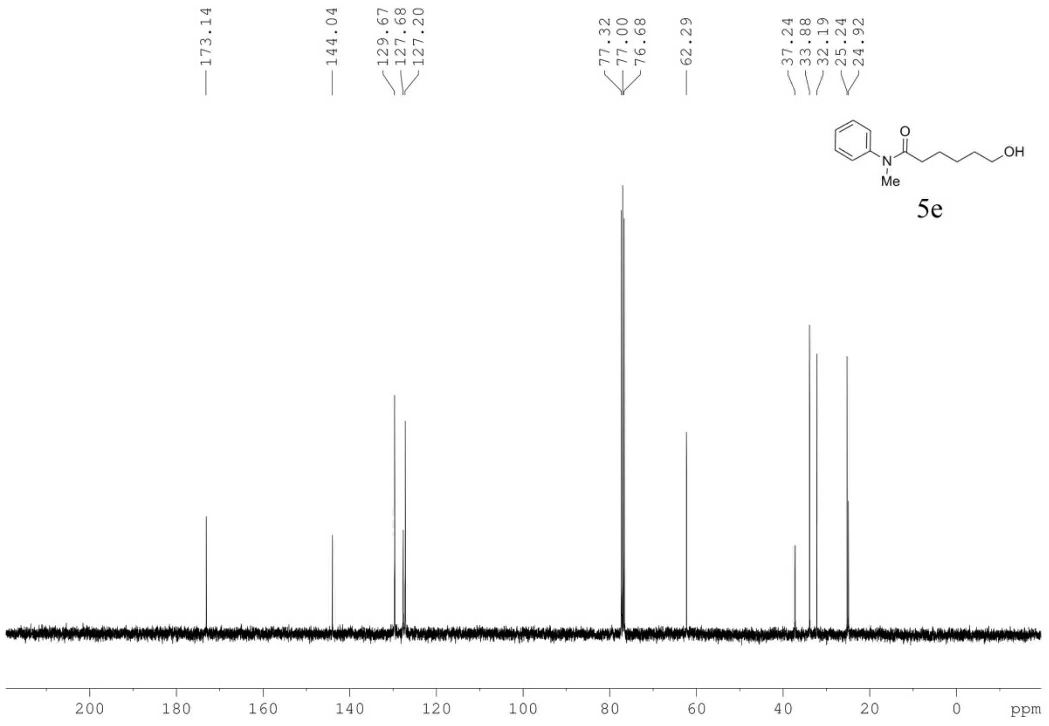

Supplementary Figure 15. ^{13}C NMR spectrum for 6-hydroxy-*N*-methyl-*N*-phenyl hexanamide (**5e**).

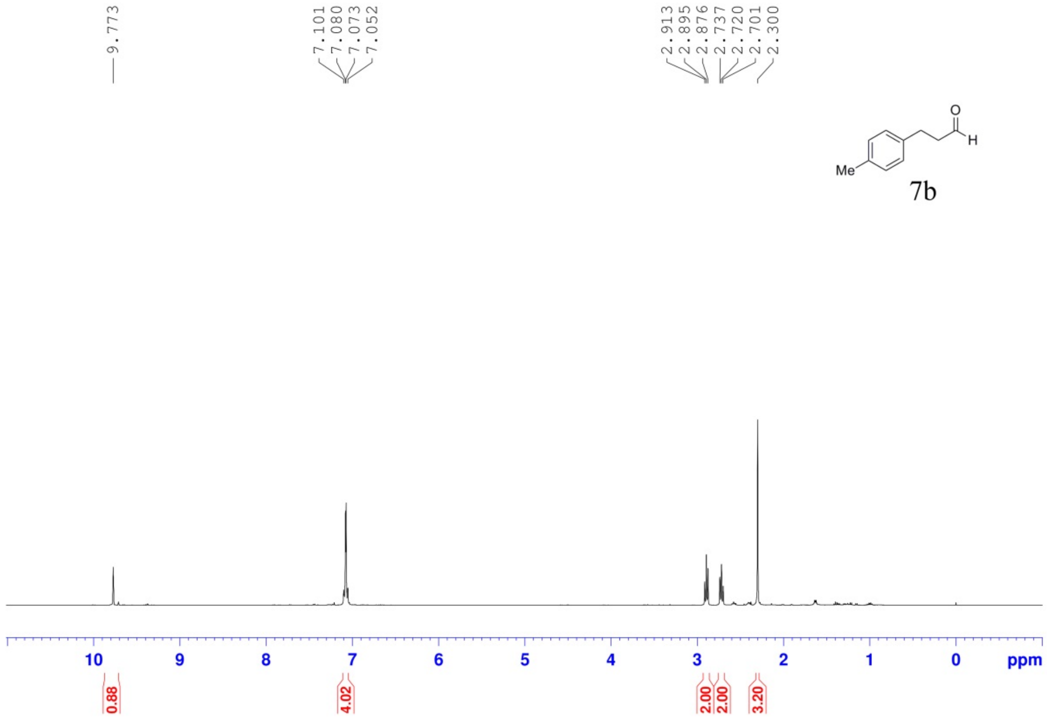

Supplementary Figure 16. ^{1}H NMR spectrum for 3-(*p*-tolyl)propanal (**7b**).

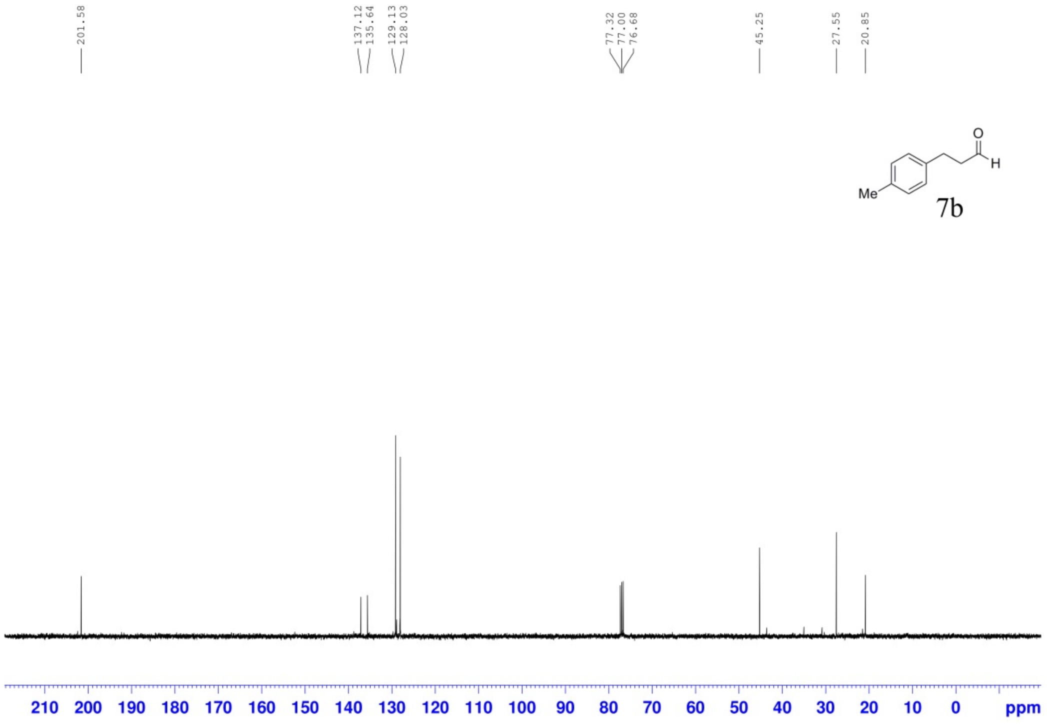

Supplementary Figure 17. ^{13}C NMR spectrum for 3-(*p*-tolyl)propanal (**7b**).

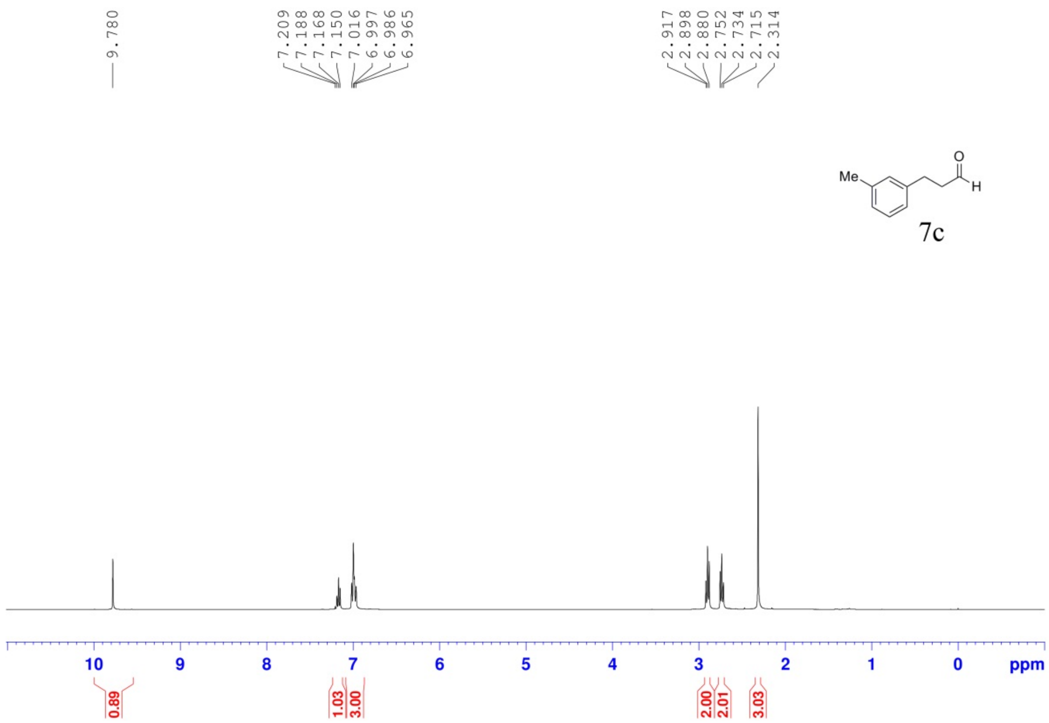

Supplementary Figure 18. ^{1}H NMR spectrum for 3-(*m*-tolyl)propanal (**7c**).

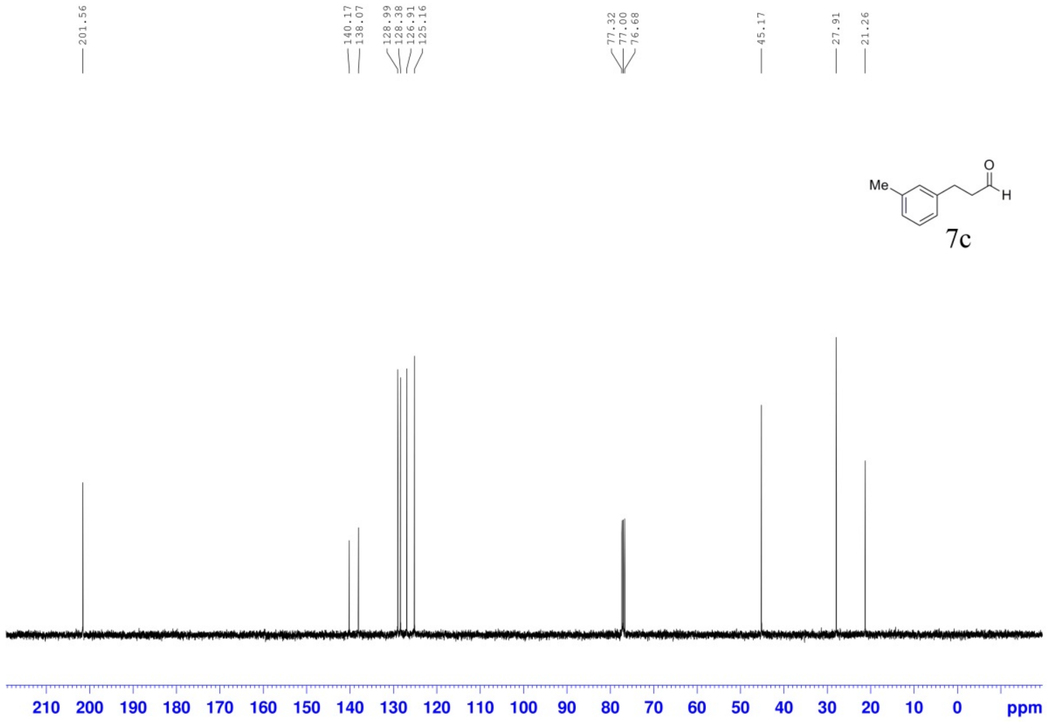

Supplementary Figure 19. ^{13}C NMR spectrum for 3-(*m*-tolyl)propanal (**7c**).

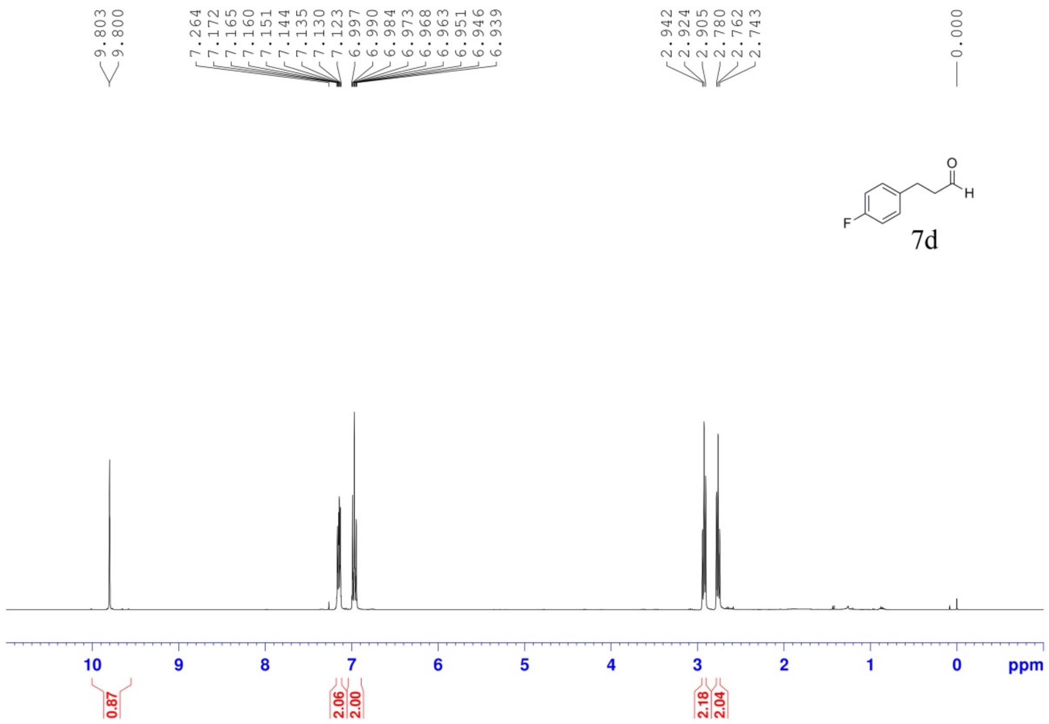

Supplementary Figure 20. ^{1}H NMR spectrum for 3-(4-fluorophenyl)propanal **(7d)**.

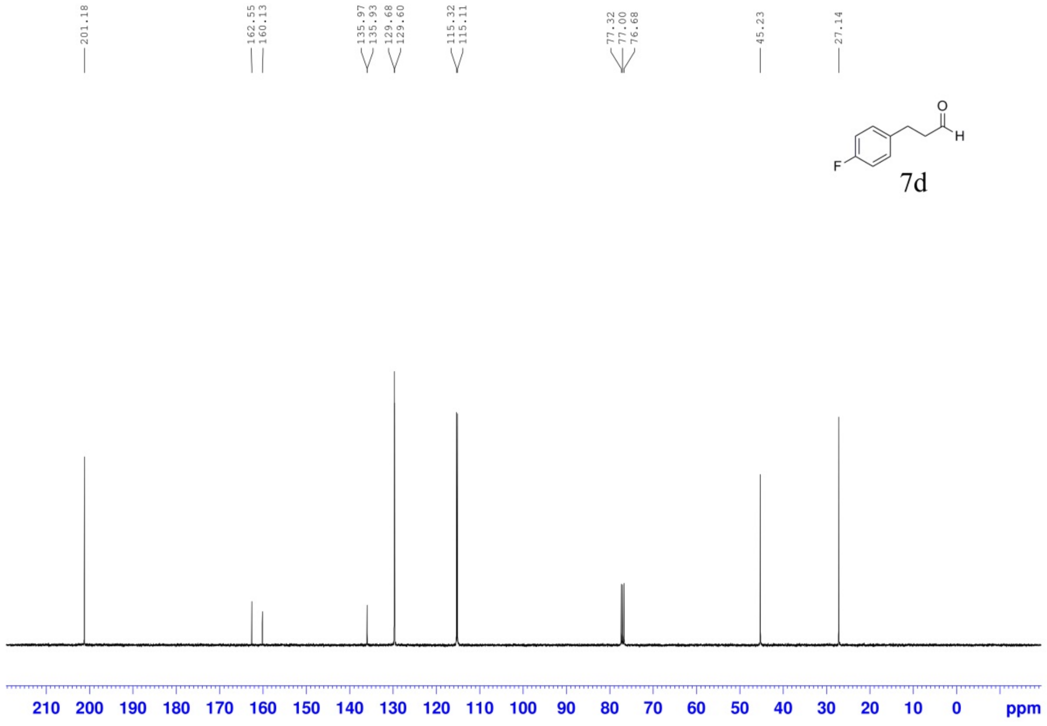

Supplementary Figure 21. ^{13}C NMR spectrum for 3-(4-fluorophenyl)propanal **(7d)**.

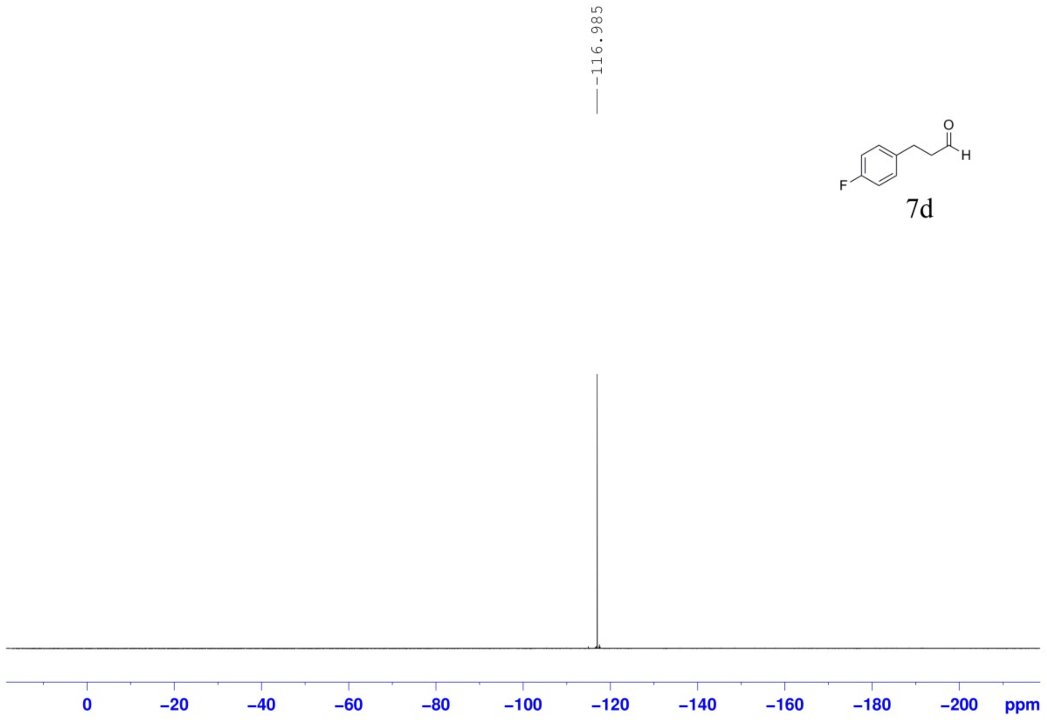

Supplementary Figure 22. ^{19}F NMR spectrum for 3-(4-fluorophenyl)propanal **(7d).**

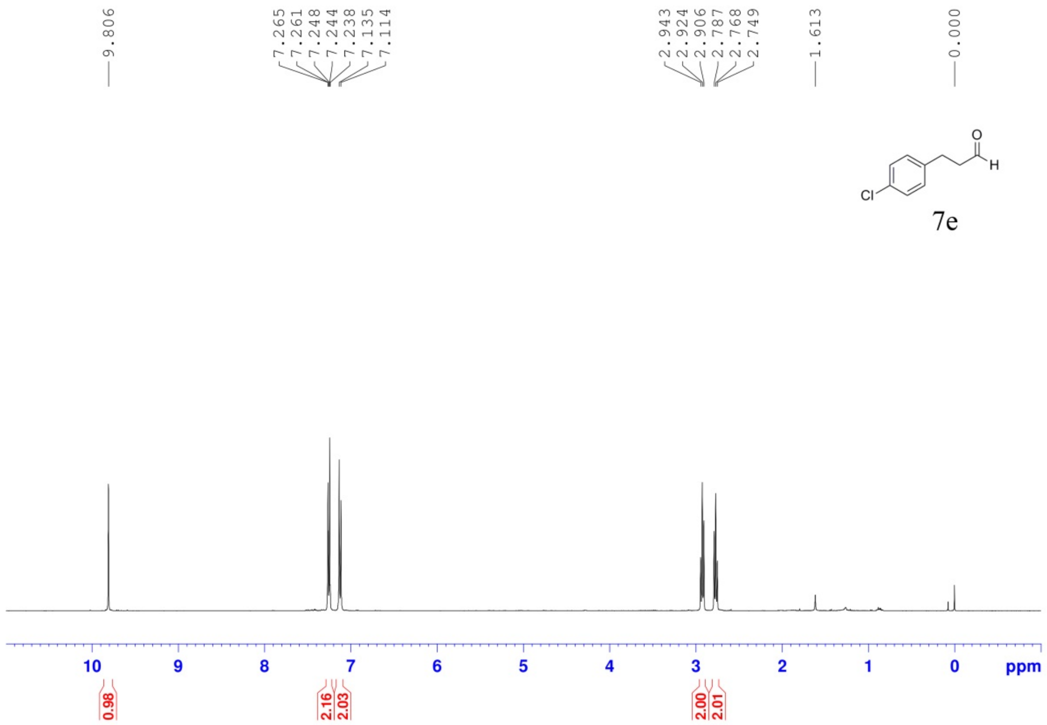

Supplementary Figure 23. ^{1}H NMR spectrum for 3-(4-chlorophenyl)propanal **(7e)**.

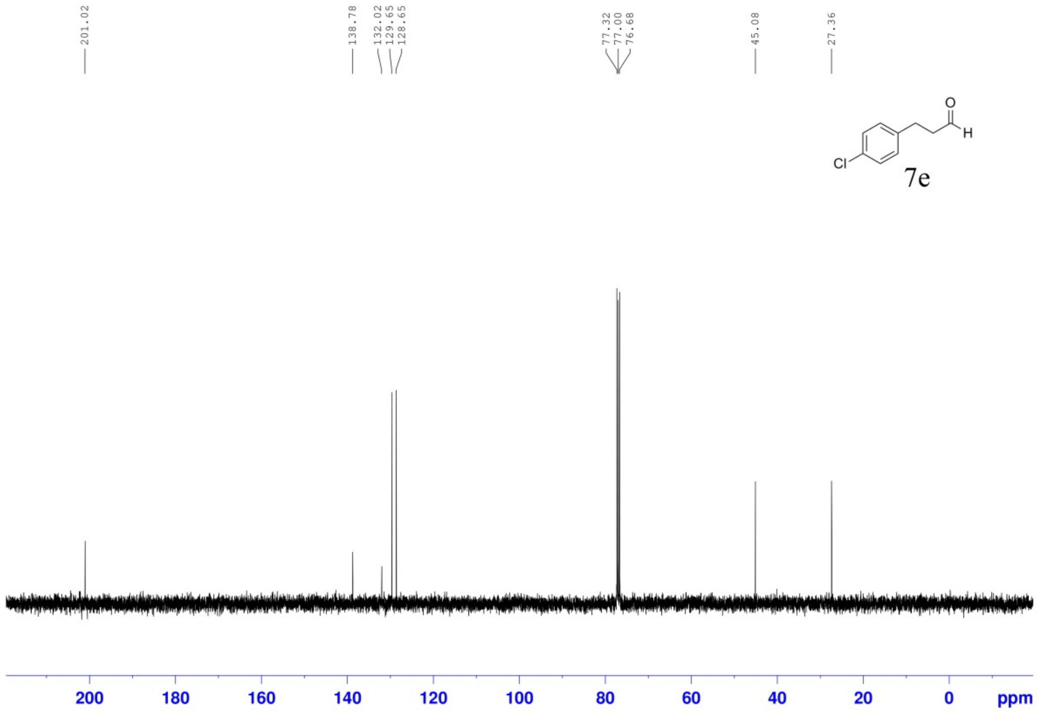

Supplementary Figure 24. ^{13}C NMR spectrum for 3-(4-chlorophenyl)propanal **(7e).**

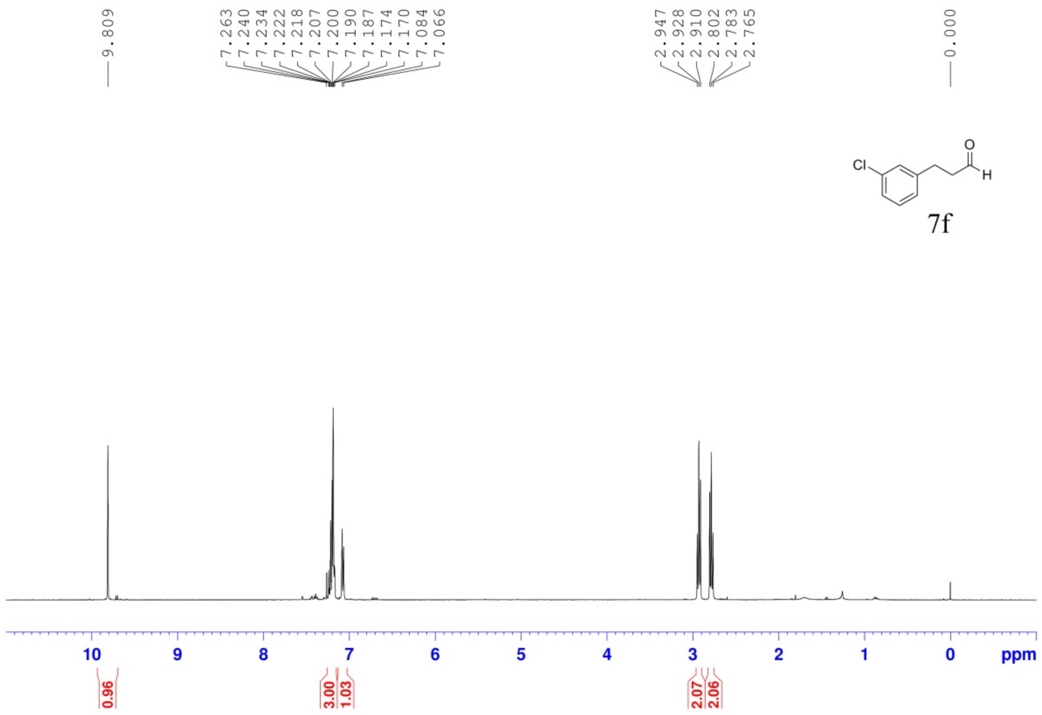

Supplementary Figure 25. ^{1}H NMR spectrum for 3-(3-chlorophenyl)propanal **(7f)**.

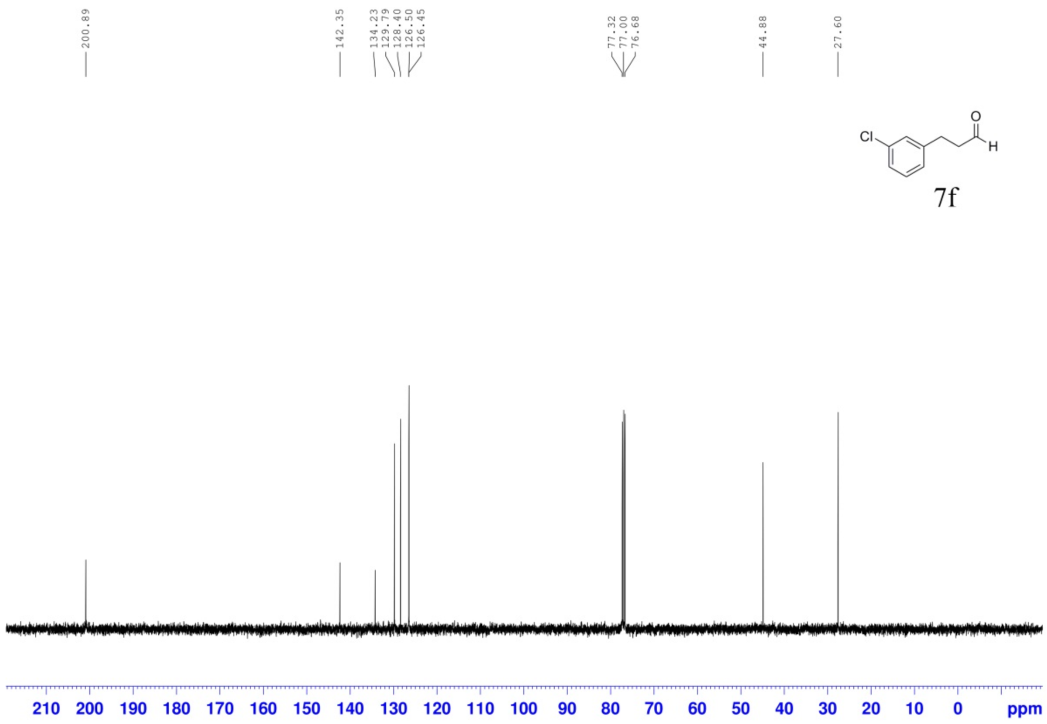

Supplementary Figure 26. ^{13}C NMR spectrum for 3-(3-chlorophenyl)propanal **(7f)**.

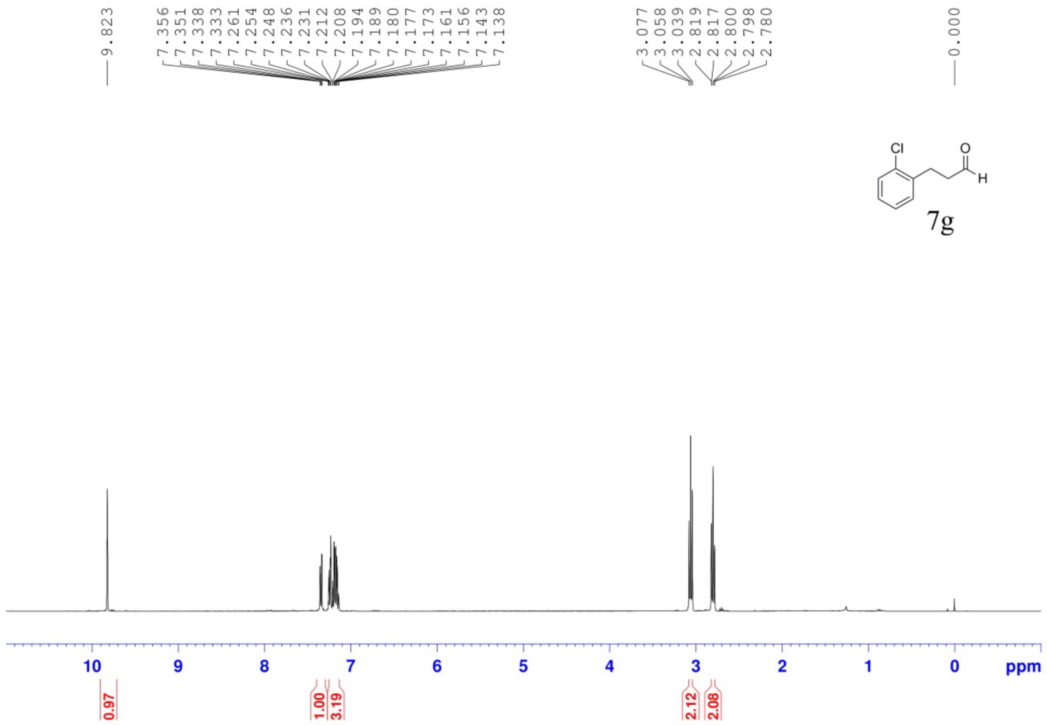

Supplementary Figure 27. ^{1}H NMR spectrum for 3-(2-chlorophenyl)propanal **(7g)**.

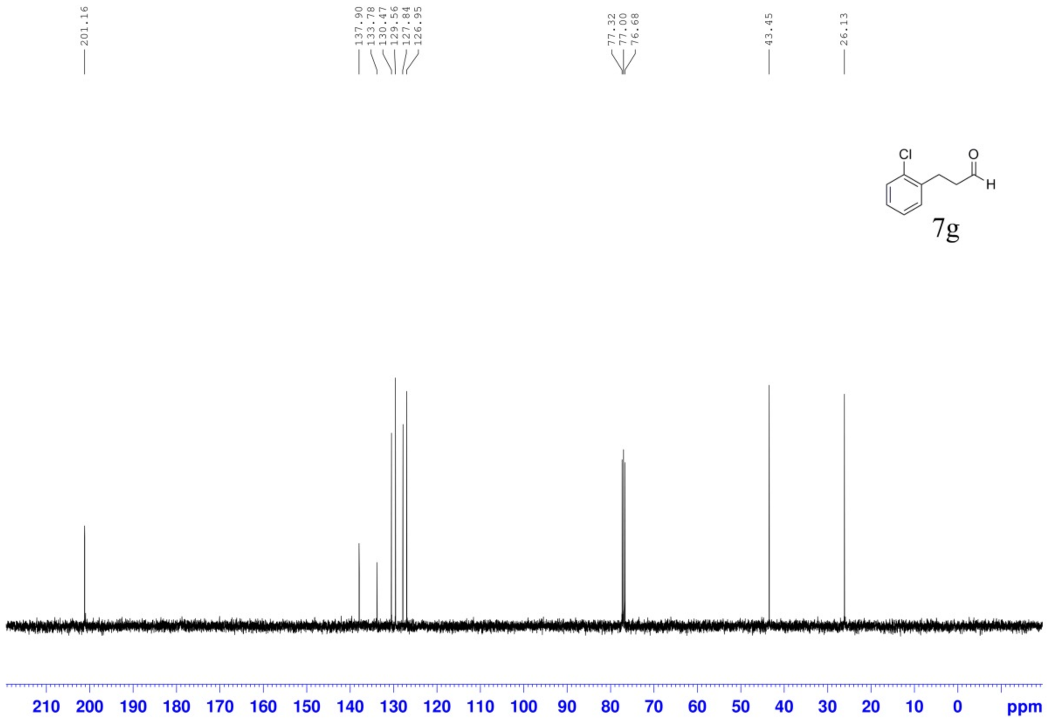

Supplementary Figure 28. ^{13}C NMR spectrum for 3-(2-chlorophenyl)propanal **(7g)**.

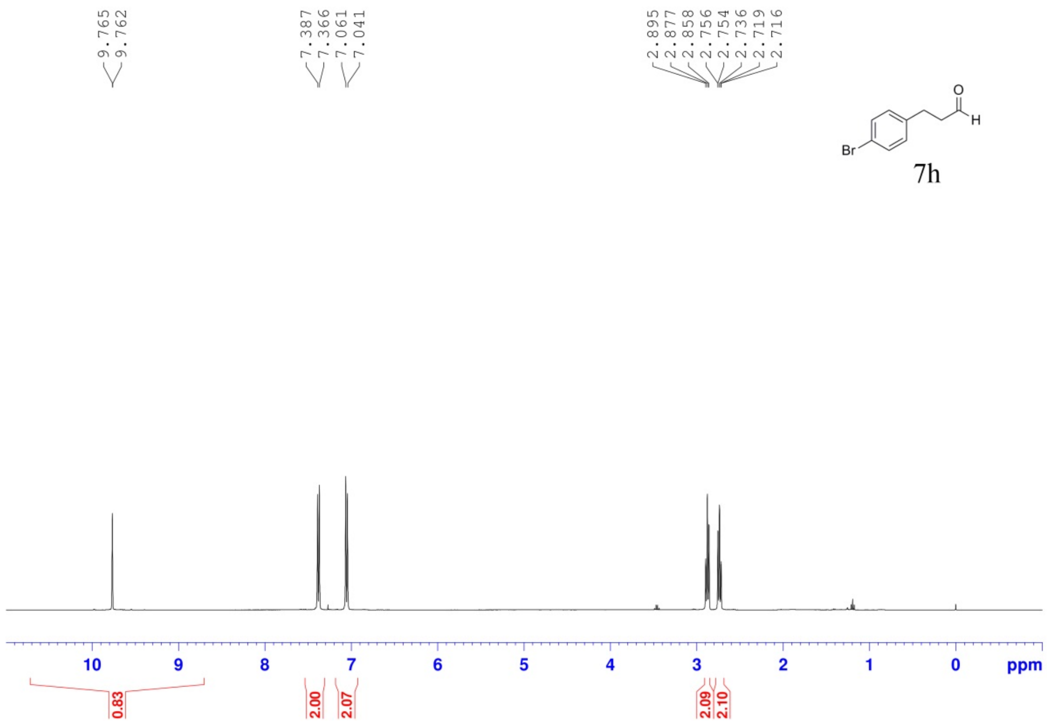

Supplementary Figure 29. ^{1}H NMR spectrum for 3-(4-bromophenyl)propanal **(7h)**.

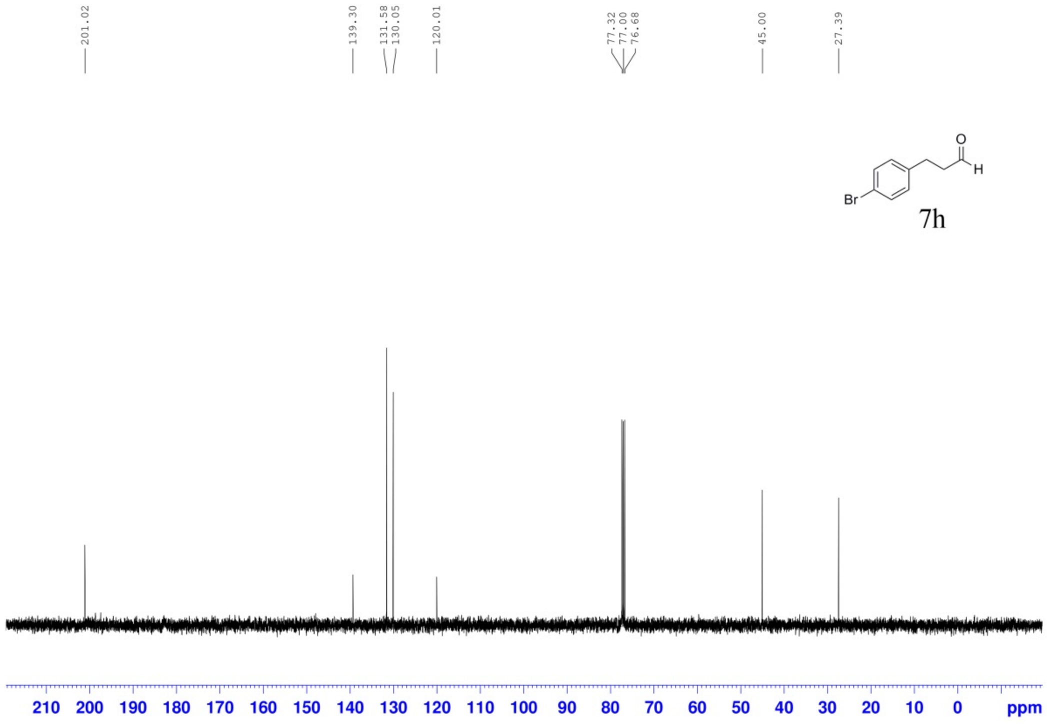

Supplementary Figure 30. ^{13}C NMR spectrum for 3-(4-bromophenyl)propanal **(7h)**.

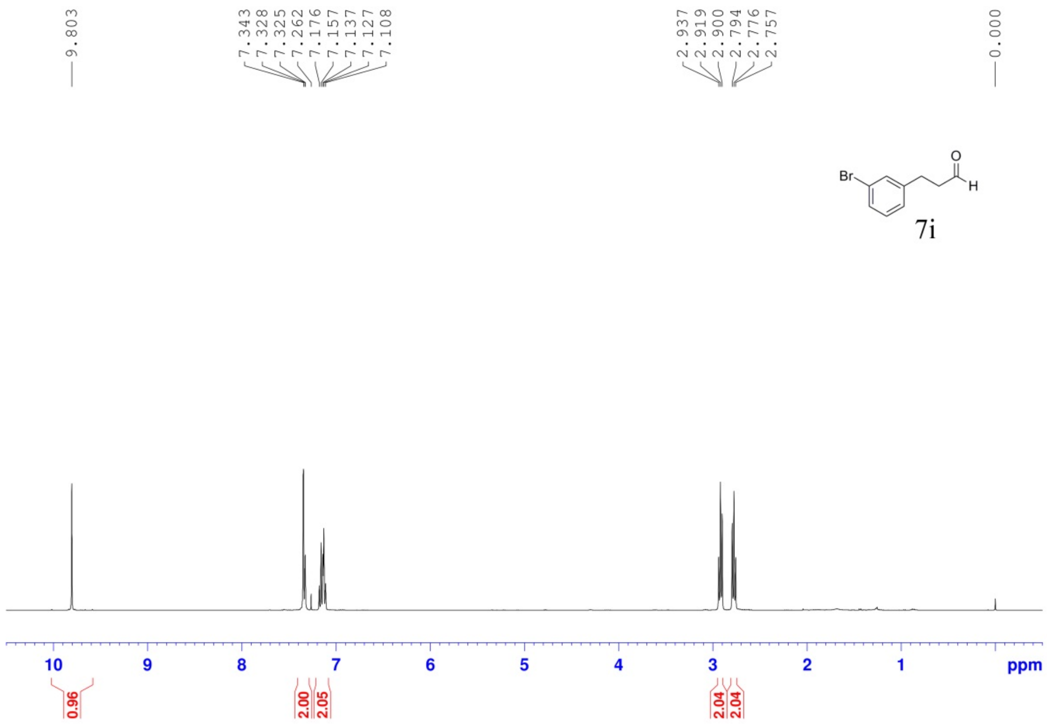

Supplementary Figure 31. ^{1}H NMR spectrum for 3-(3-bromophenyl)propanal **(7i)**.

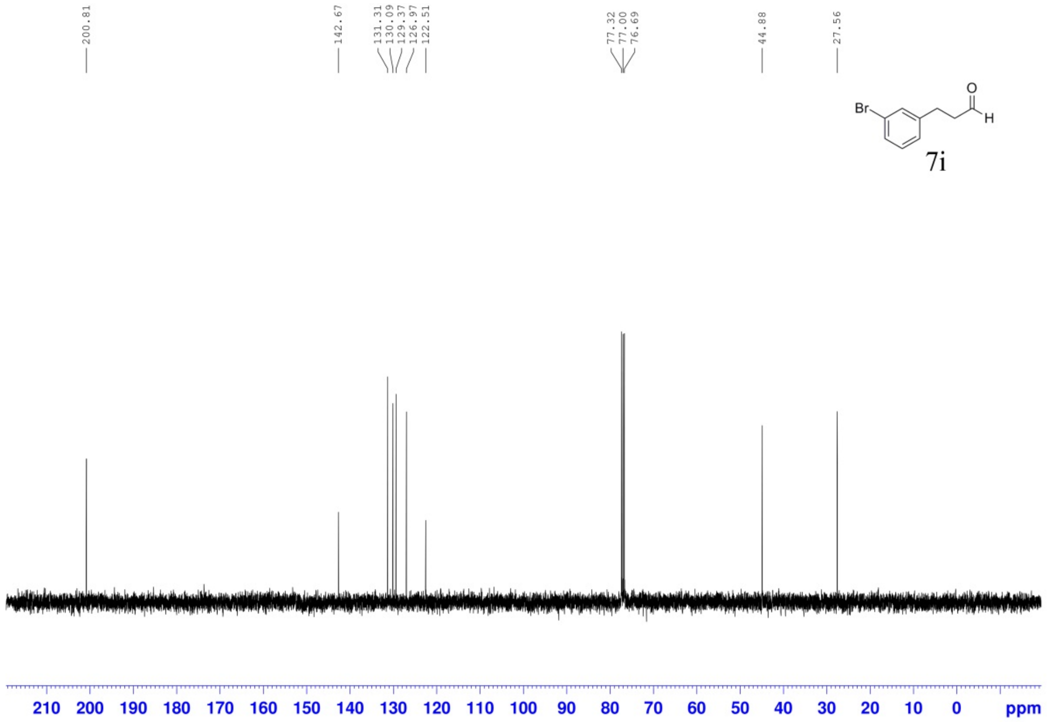

Supplementary Figure 32. ^{13}C NMR spectrum for 3-(3-bromophenyl)propanal **(7i)**.

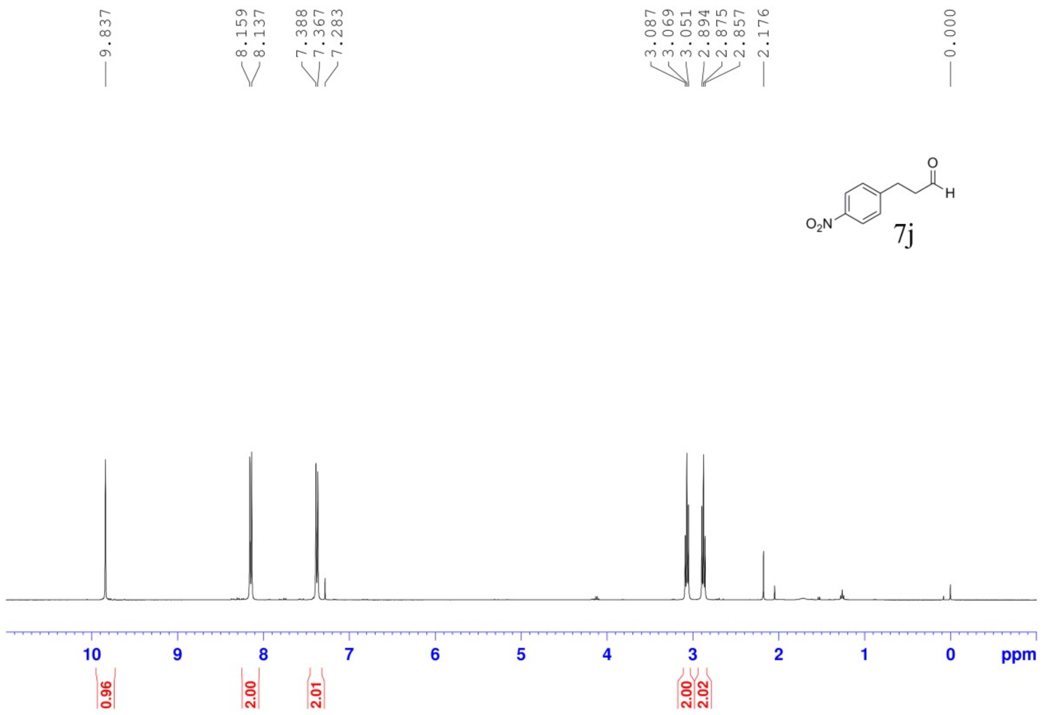

Supplementary Figure 33. ^{1}H NMR spectrum for 3-(4-nitrophenyl)propanal **(7j)**.

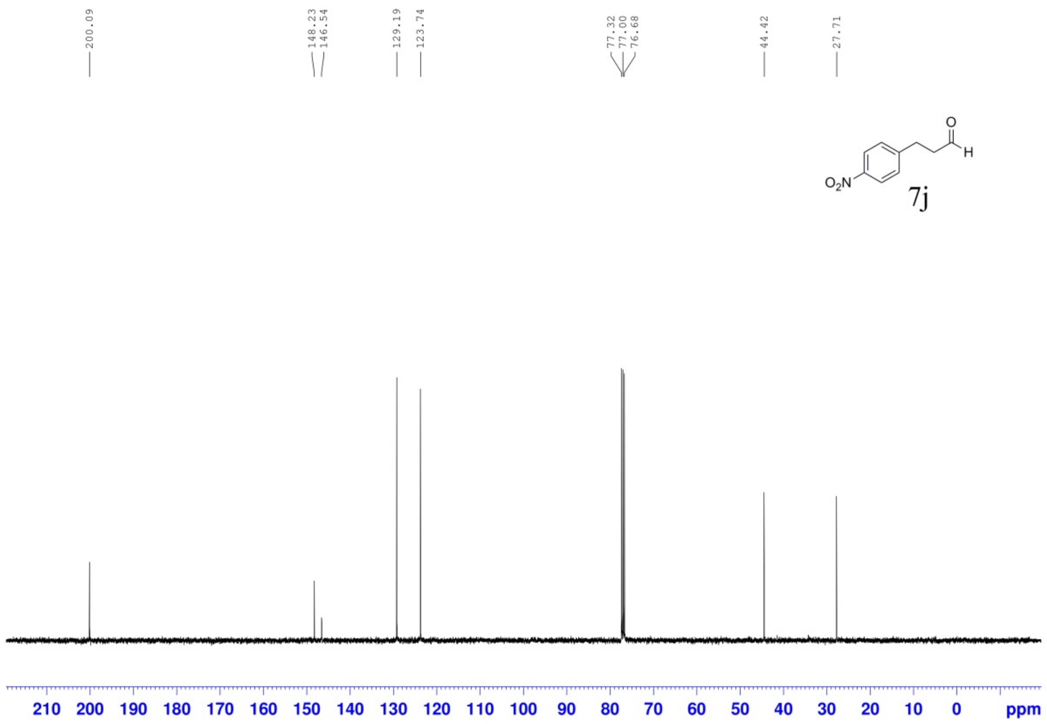

Supplementary Figure 34. ^{13}C NMR spectrum for 3-(4-nitrophenyl)propanal **(7j)**.

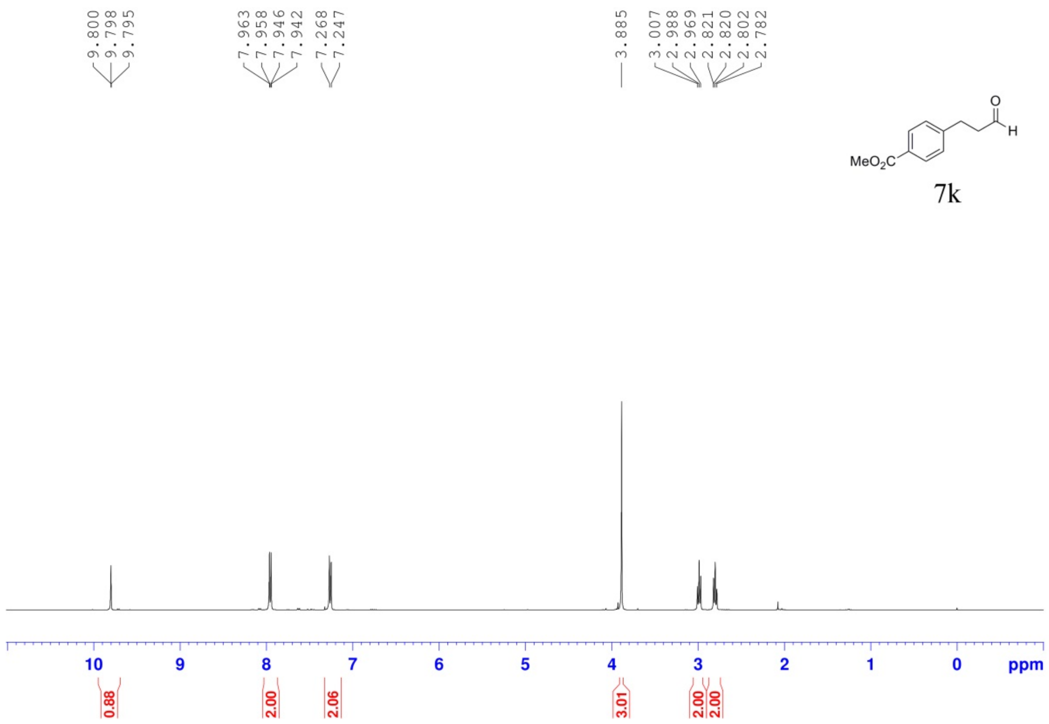

Supplementary Figure 35. ^{1}H NMR spectrum for 4-(3-oxopropyl)benzoate **(7k)**.

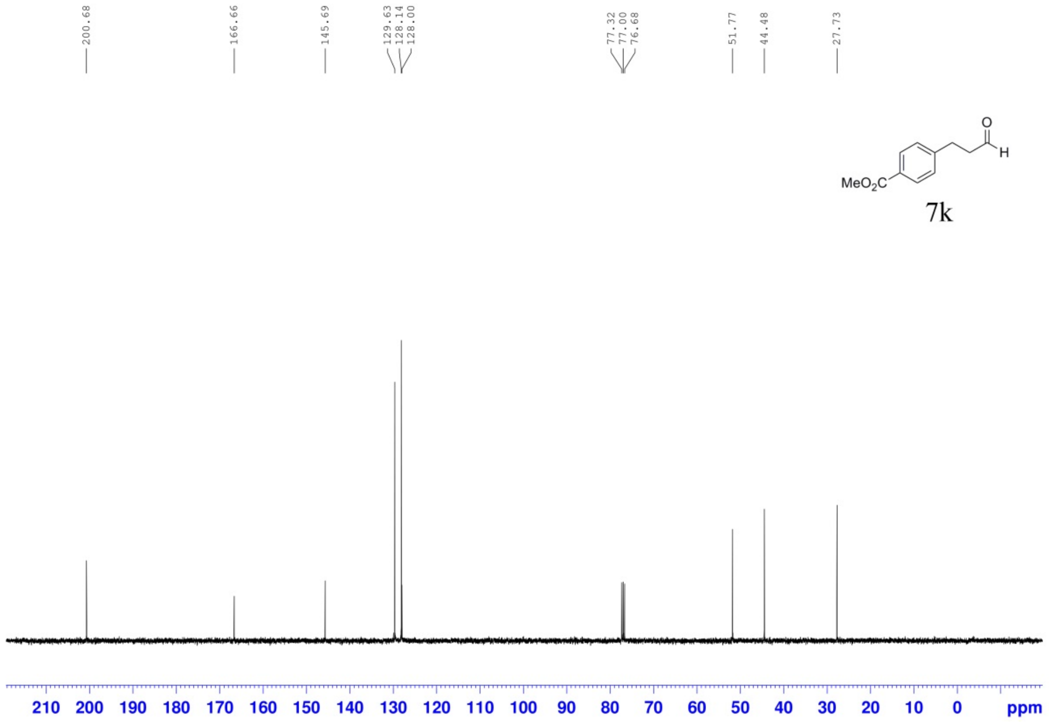

Supplementary Figure 36. ^{13}C NMR spectrum for 4-(3-oxopropyl)benzoate **(7k)**.

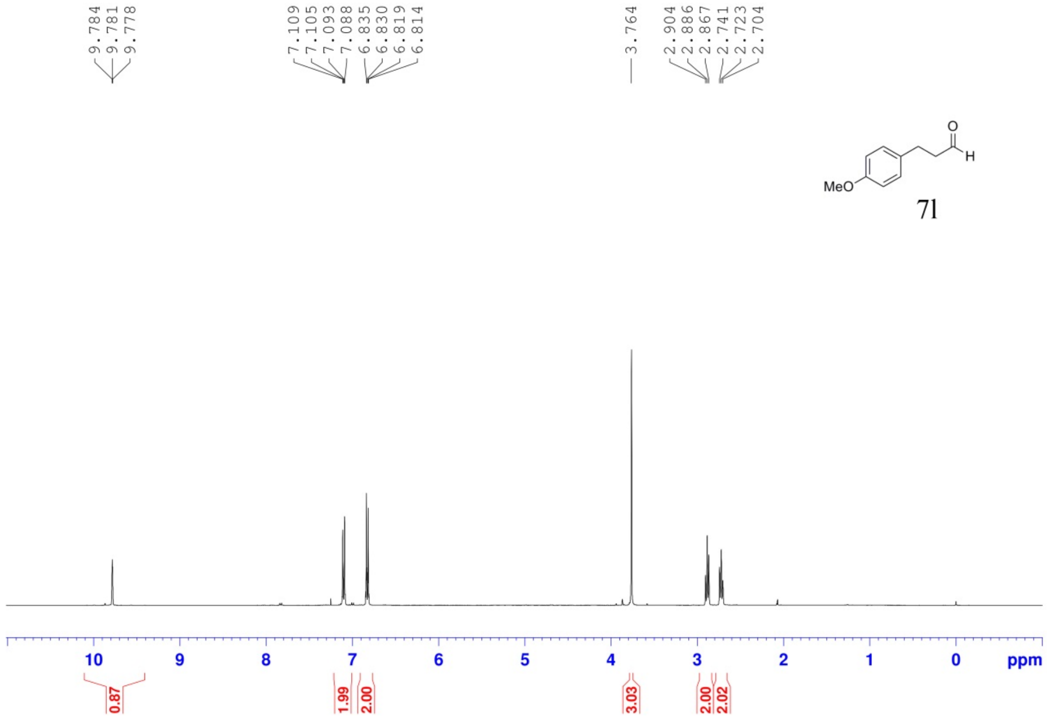

Supplementary Figure 37. ^{1}H NMR spectrum for 3-(4-methoxyphenyl)propanal **(7l)**.

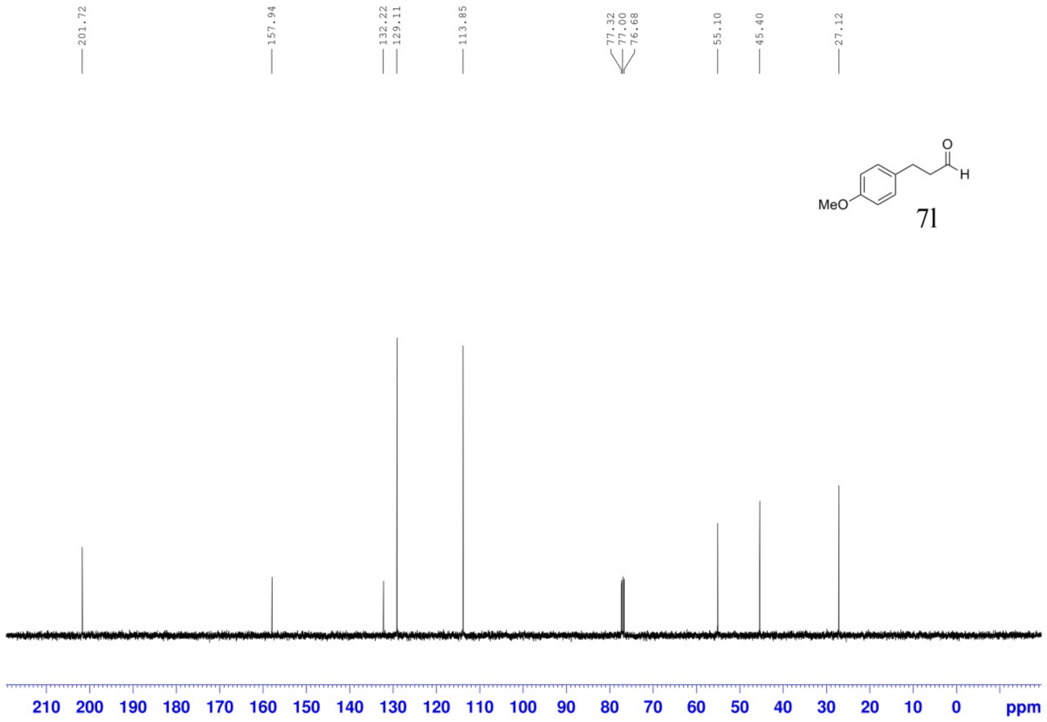

Supplementary Figure 38. ^{13}C NMR spectrum for 3-(4-methoxyphenyl)propanal **(7l)**.

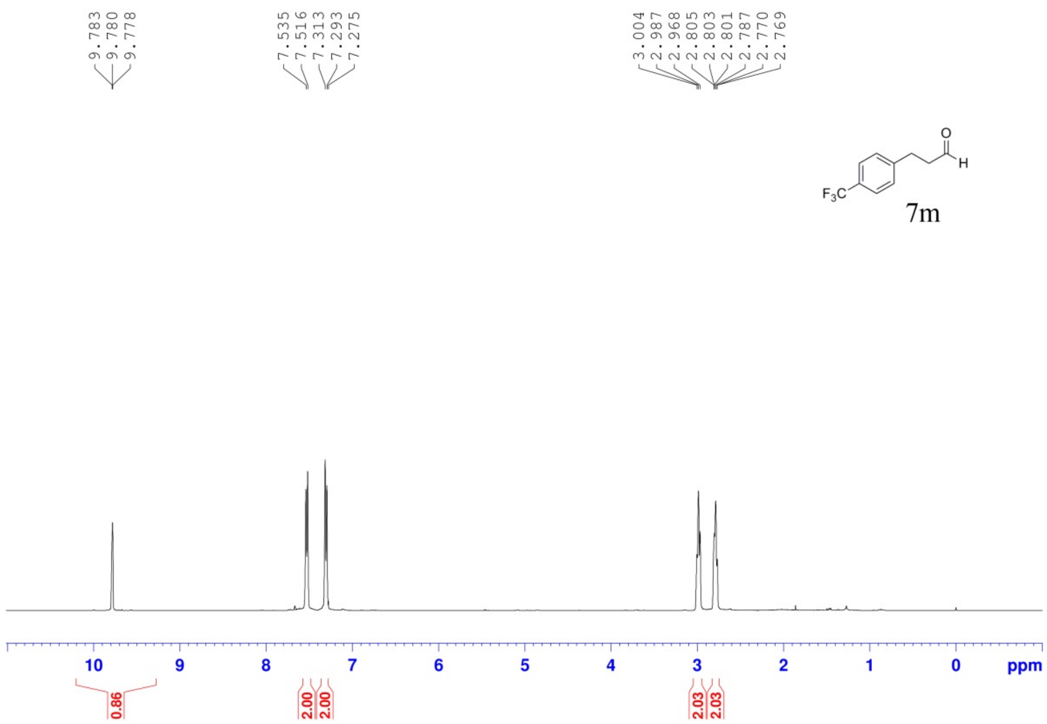

Supplementary Figure 39. ^{1}H NMR spectrum for 3-(4-(trifluoromethyl)phenyl) propanal **(7m)**.

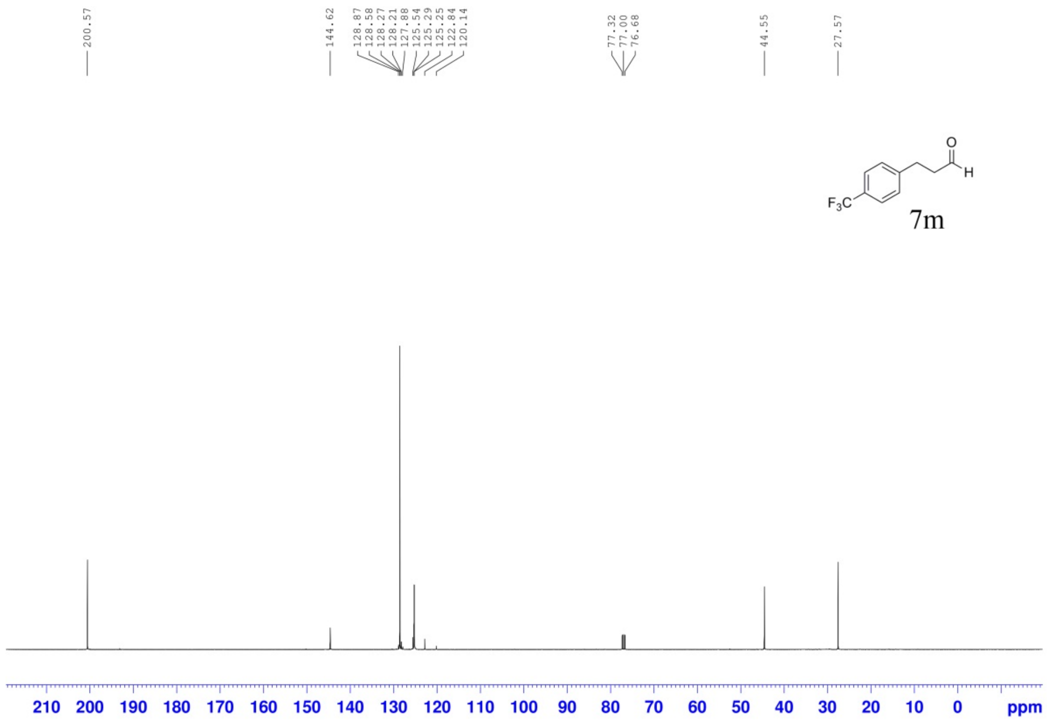

Supplementary Figure 40. ^{13}C NMR spectrum for 3-(4-(trifluoromethyl)phenyl) propanal **(7m)**.

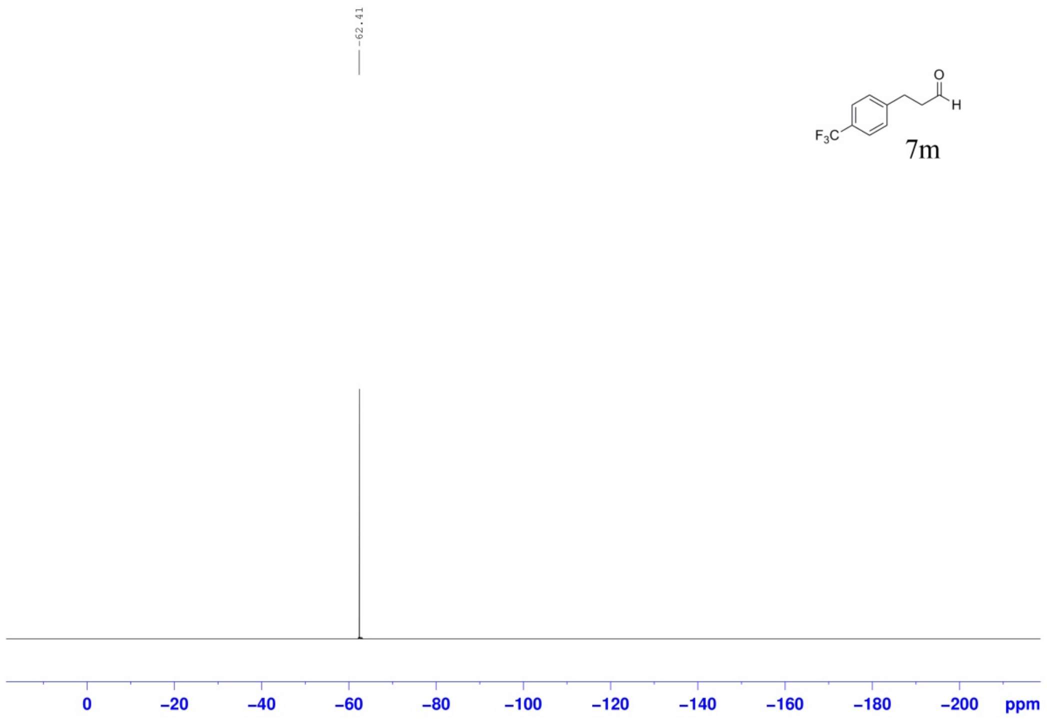

Supplementary Figure 41. ^{19}F NMR spectrum for 3-(4-(trifluoromethyl)phenyl) propanal **(7m)**.

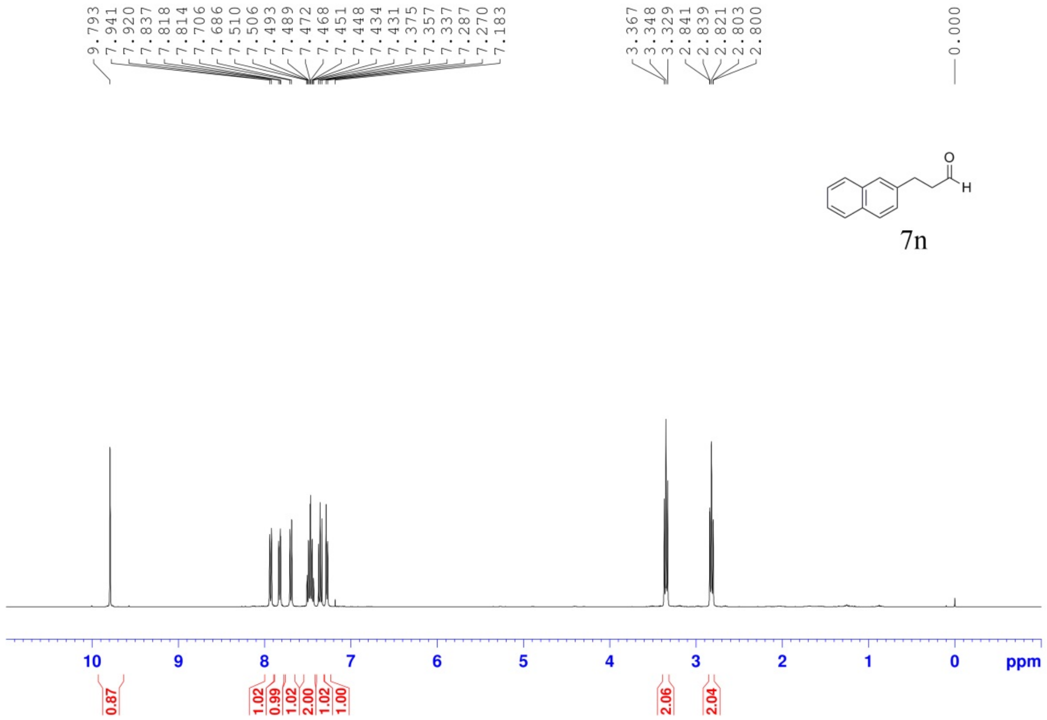

Supplementary Figure 42. ^{1}H NMR spectrum for 3-(naphthalen-2-yl)propanal **(7n)**.

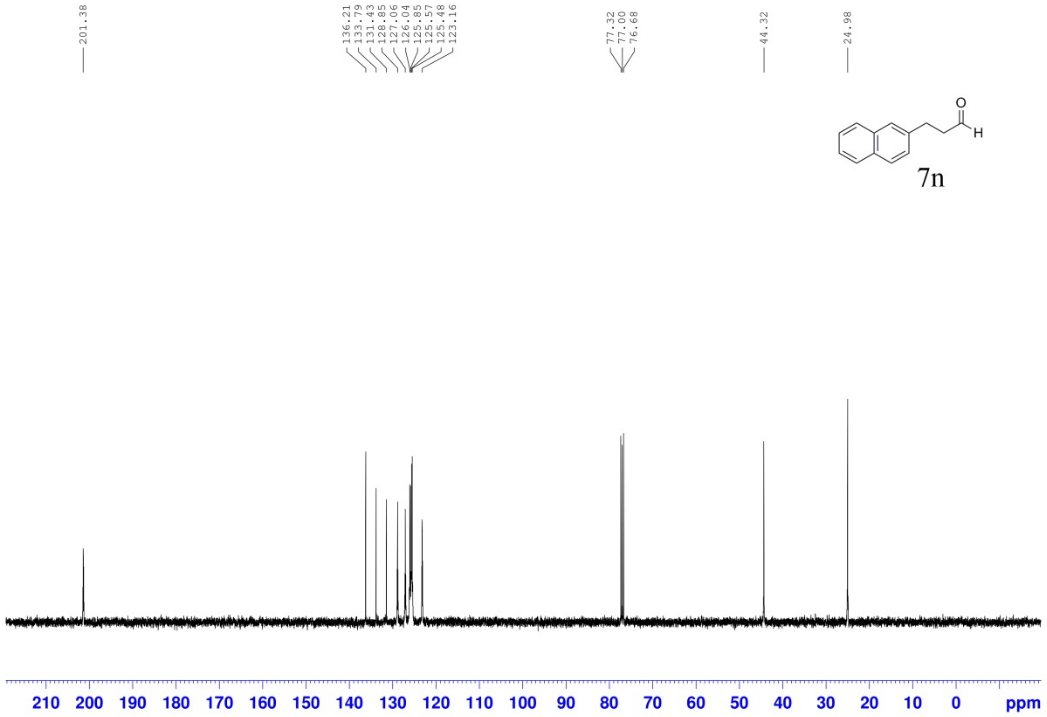

Supplementary Figure 43. ^{13}C NMR spectrum for 3-(naphthalen-2-yl)propanal **(7n)**.

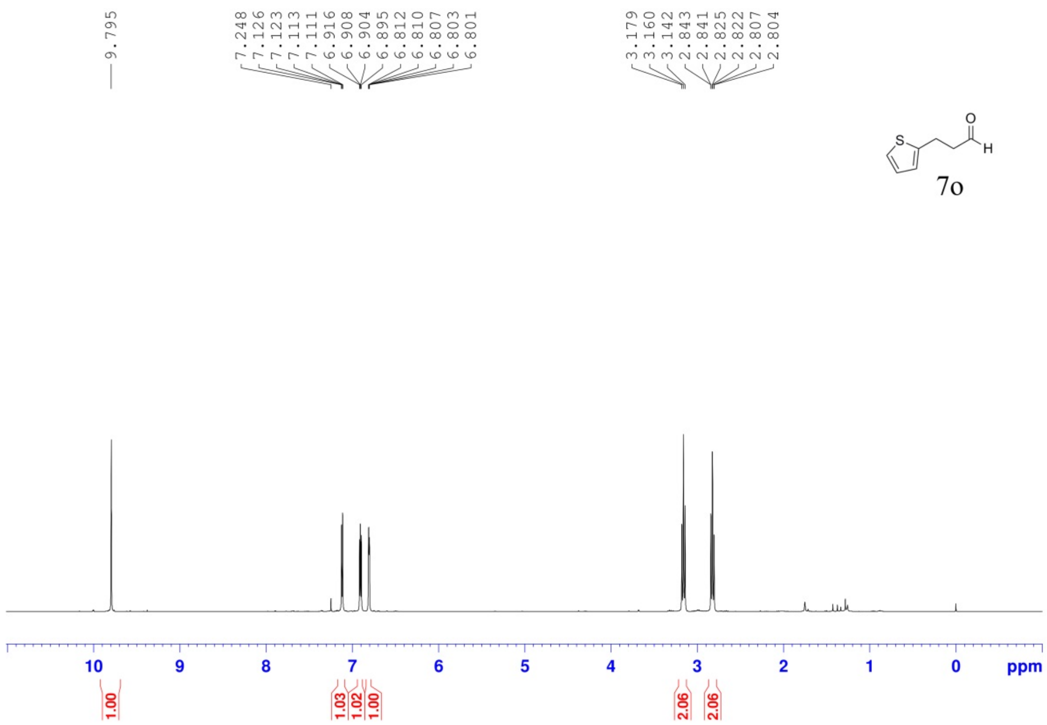

Supplementary Figure 44. ^{1}H NMR spectrum for 3-(thiophen-2-yl)propanal **(7o)**.

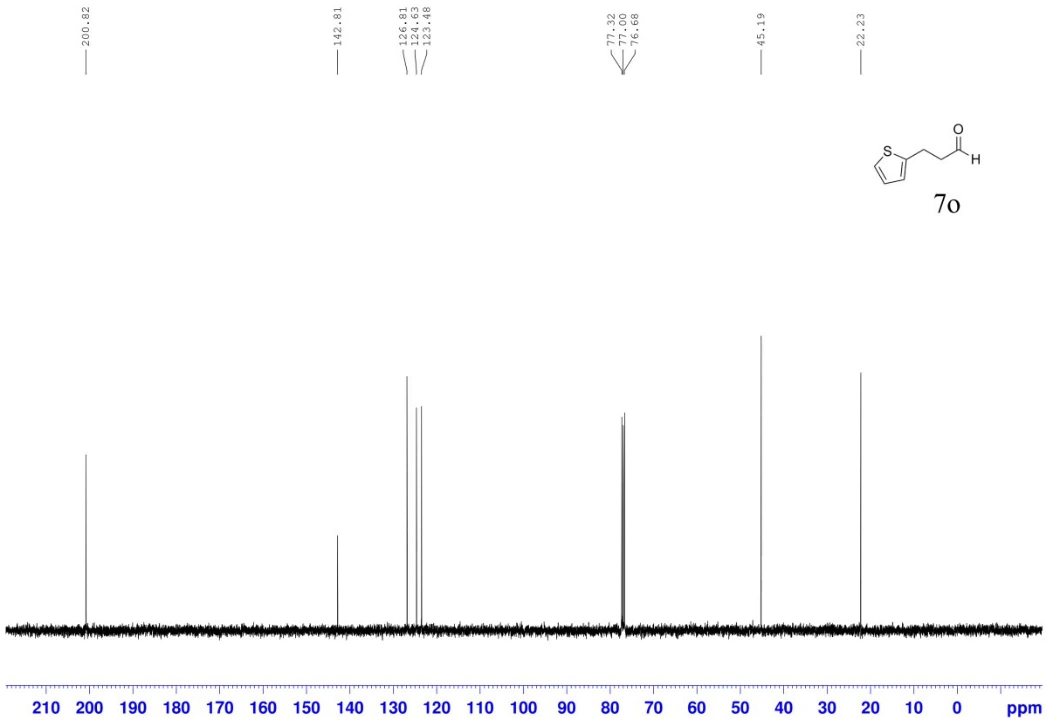

Supplementary Figure 45. ^{13}C NMR spectrum for 3-(thiophen-2-yl)propanal **(7o)**.

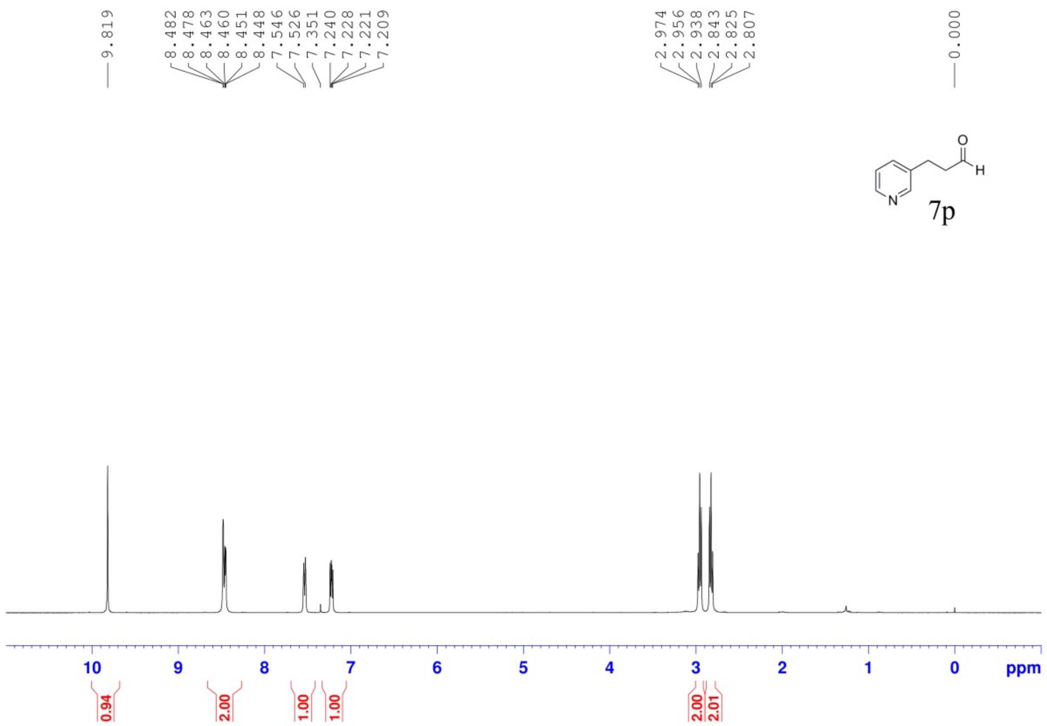

Supplementary Figure 46. ^{1}H NMR spectrum for 3-(pyridin-3-yl)propanal **(7p)**.

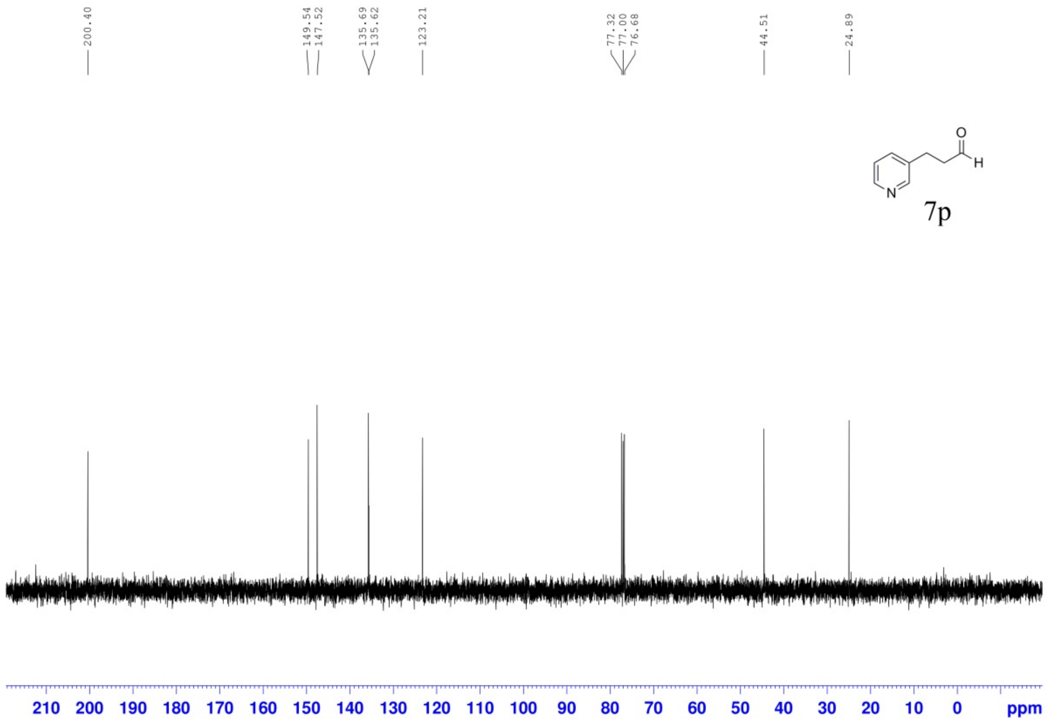

Supplementary Figure 47. ^{13}C NMR spectrum for 3-(pyridin-3-yl)propanal **(7p)**.

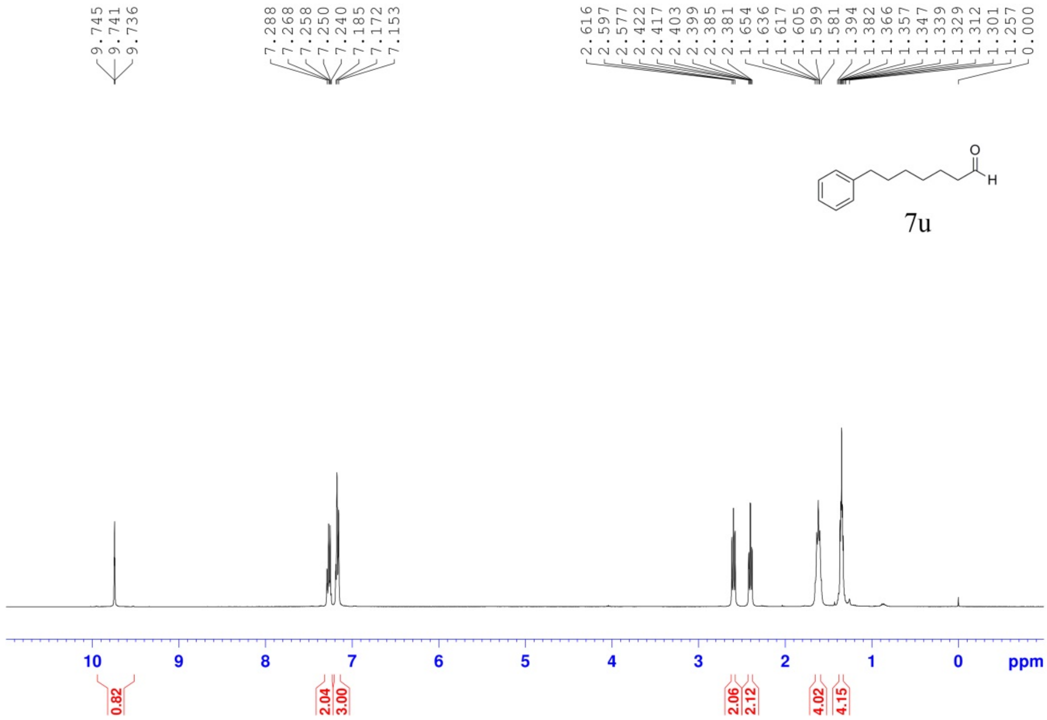

Supplementary Figure 48. ^{1}H NMR spectrum for 7-phenylheptanal **(7u)**.

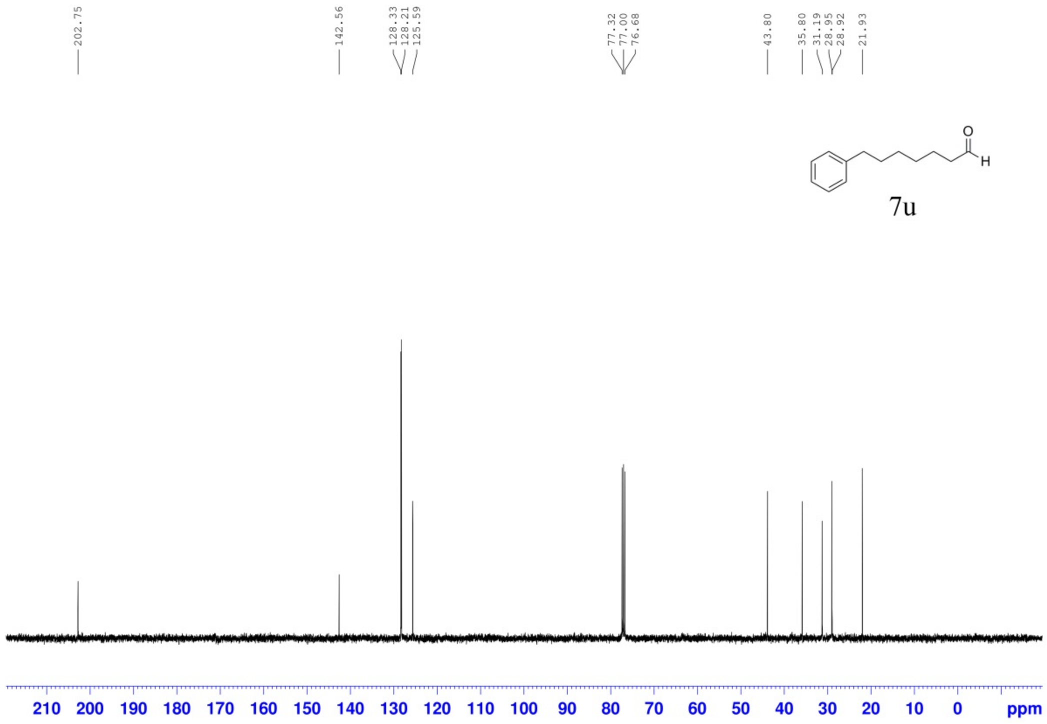

Supplementary Figure 49. ^{13}C NMR spectrum for 7-phenylheptanal **(7u)**.

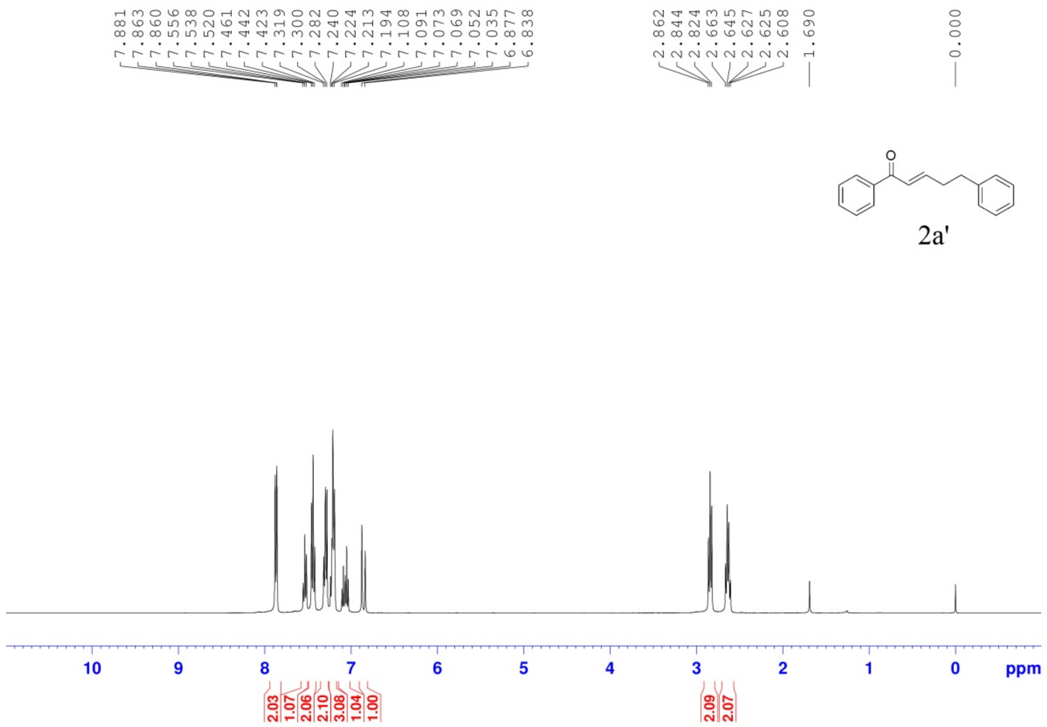

Supplementary Figure 50. ^{1}H NMR spectrum for (*E*)-1,5-diphenylpent-2-en-1-one **(2a')**.

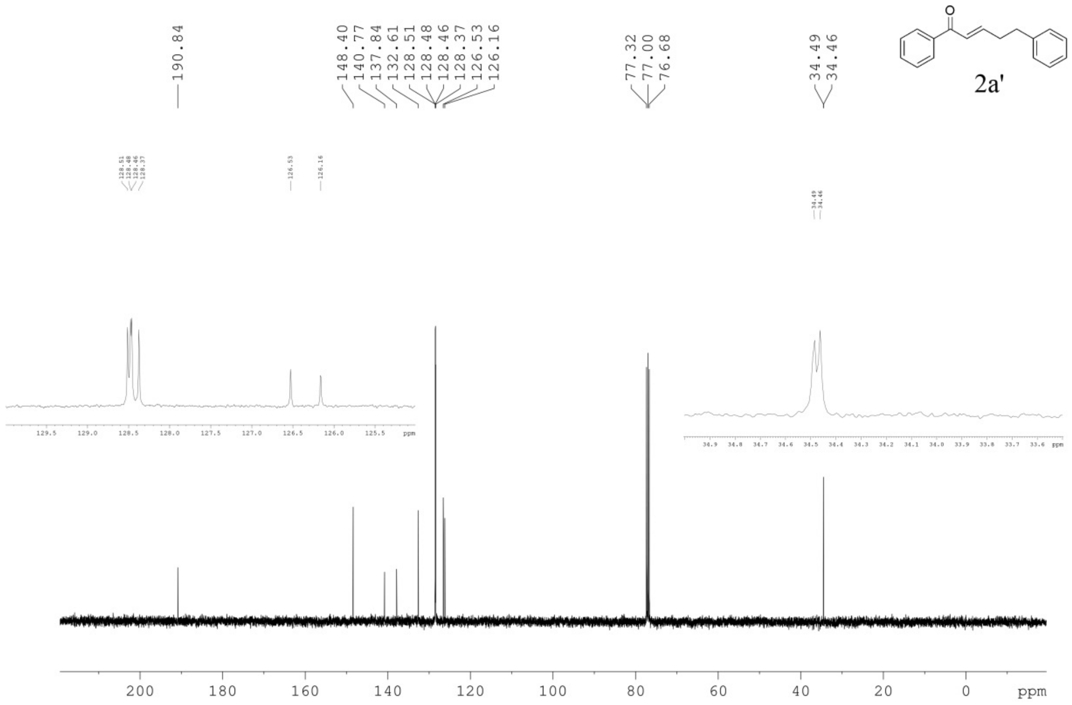

Supplementary Figure 51. ^{13}C NMR spectrum for (*E*)-1,5-diphenylpent-2-en-1-one **(2a')**.

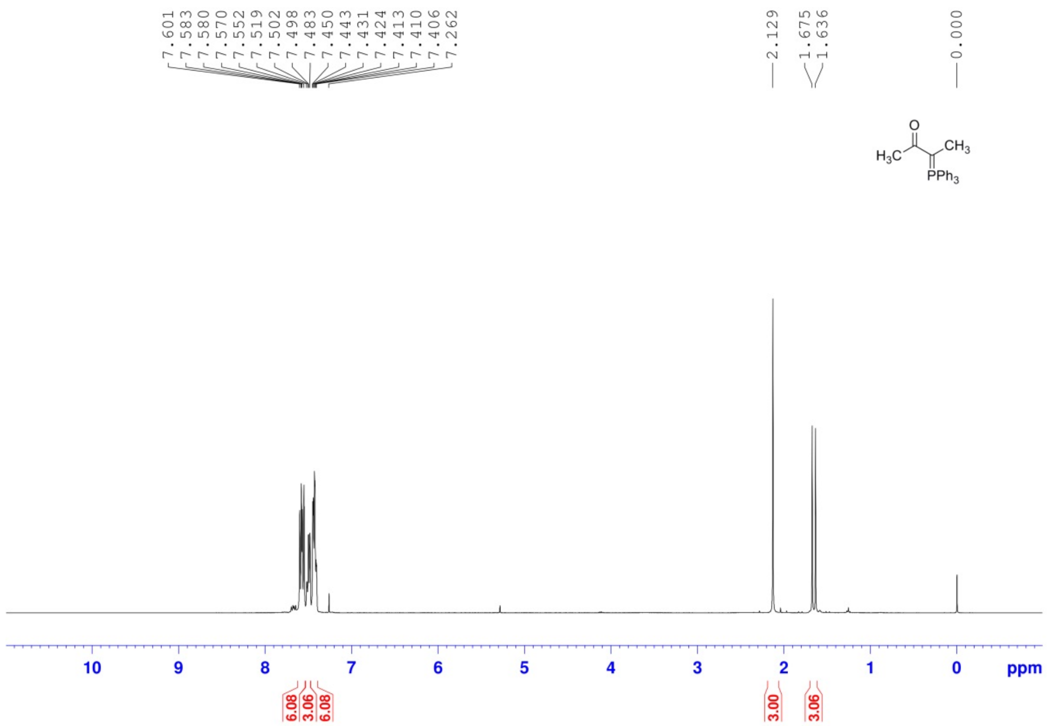

Supplementary Figure 52. ^{1}H NMR spectrum for 3-(triphenylphosphoranylidene) butan-2-one.

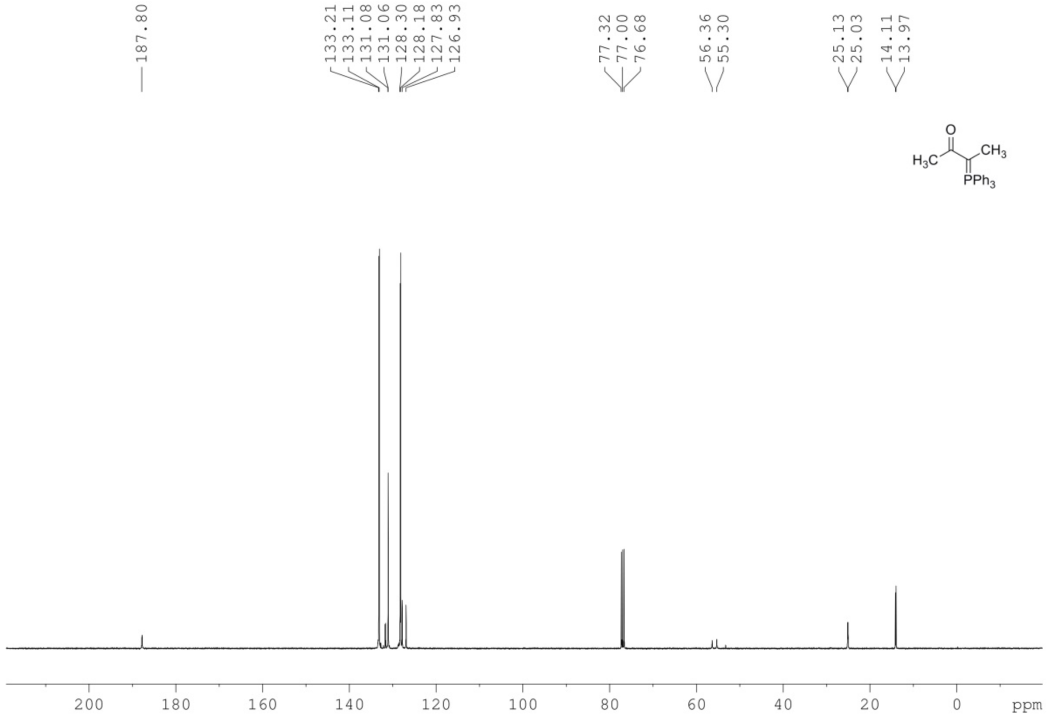

Supplementary Figure 53. ^{13}C NMR spectrum for 3-(triphenylphosphoranylidene) butan-2-one.

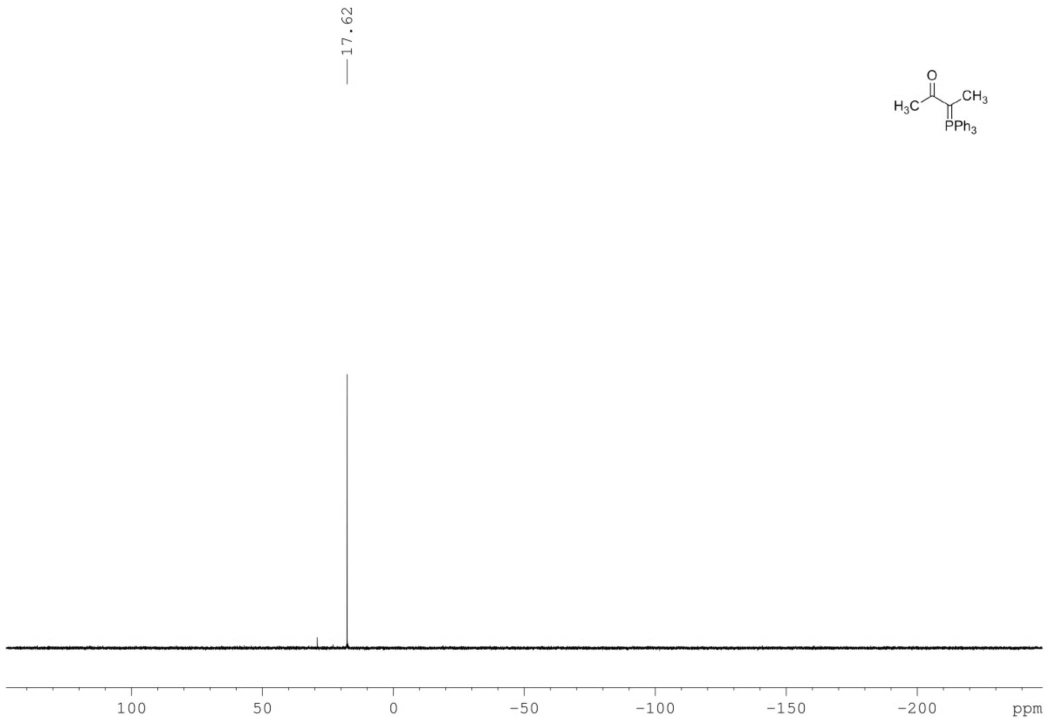

Supplementary Figure 54. ^{31}P NMR spectrum for 3-(triphenylphosphoranylidene) butan-2-one.

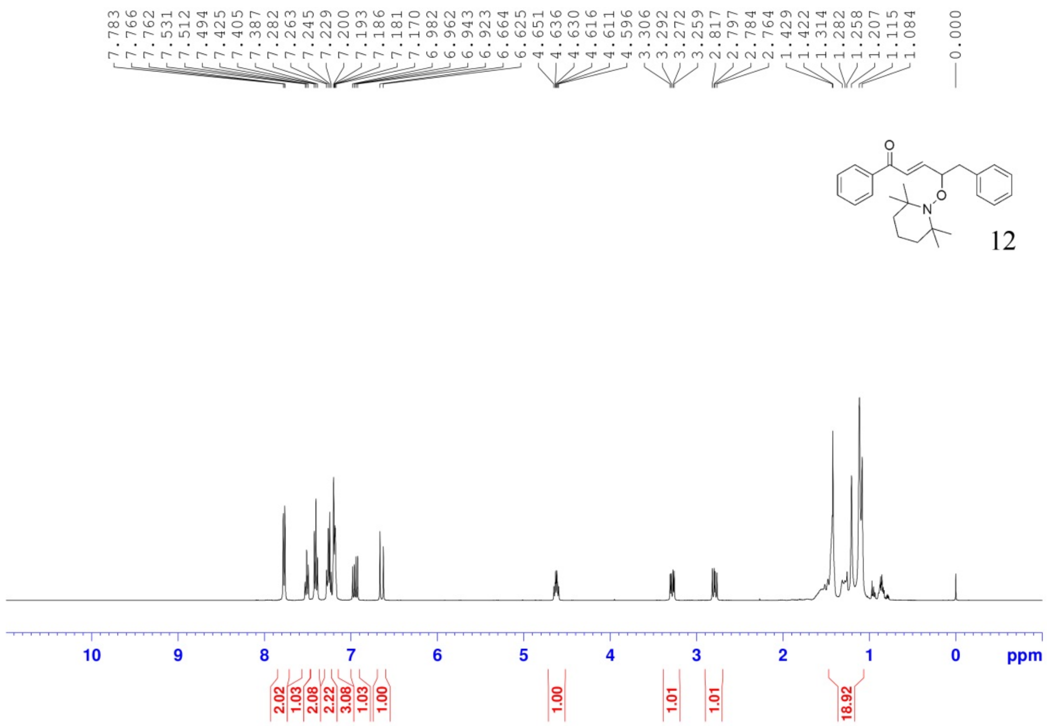

Supplementary Figure 55. ^{1}H NMR spectrum for γ-TEMPO-substituted enone intermediate **12**.

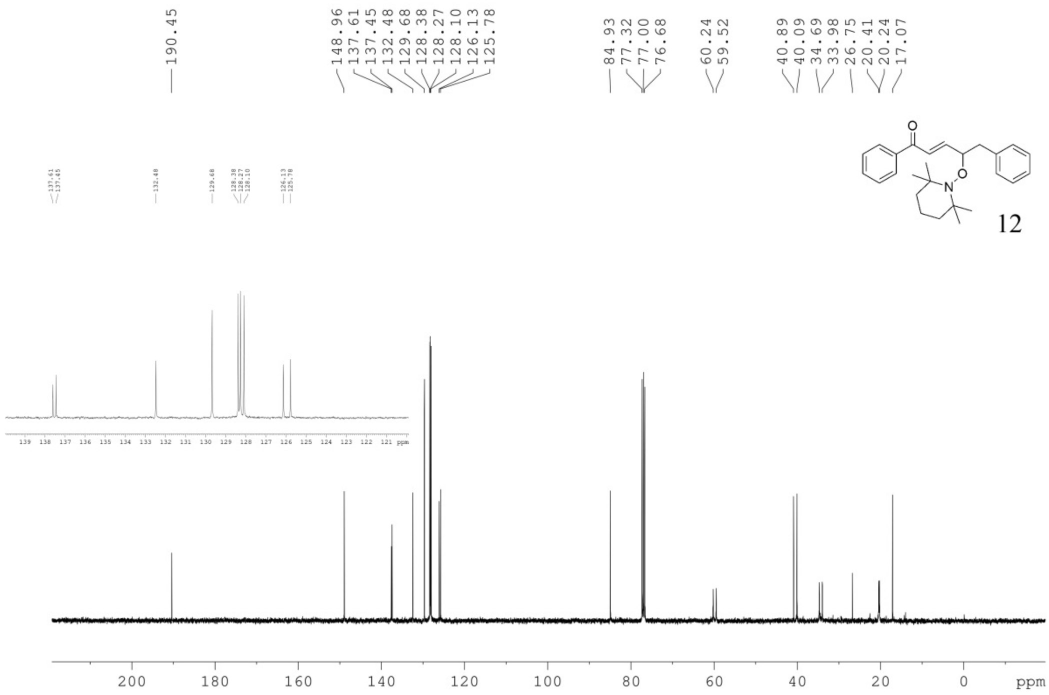

Supplementary Figure 56. ^{13}C NMR spectrum for γ-TEMPO-substituted enone intermediate **12**.

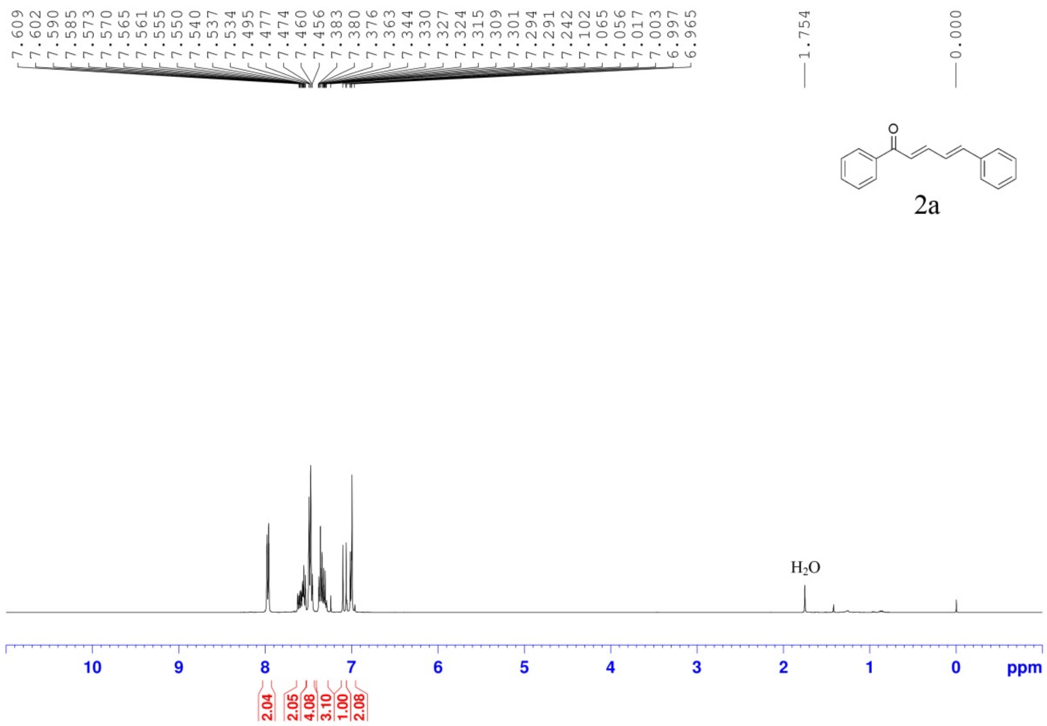

Supplementary Figure 57. ^{1}H NMR spectrum for (2*E*,4*E*)-1,5-diphenylpenta-2,4-dien-1-one **(2a)**.

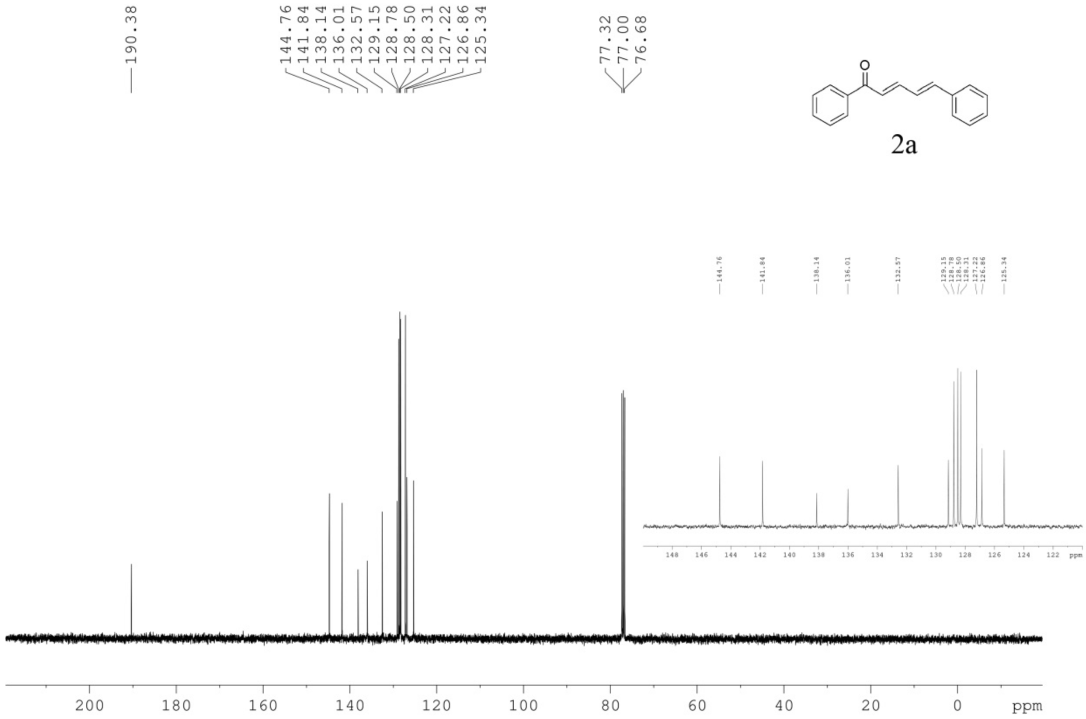

Supplementary Figure 58. ^{13}C NMR spectrum for (2*E*,4*E*)-1,5-diphenylpenta-2,4-dien-1-one **(2a)**.

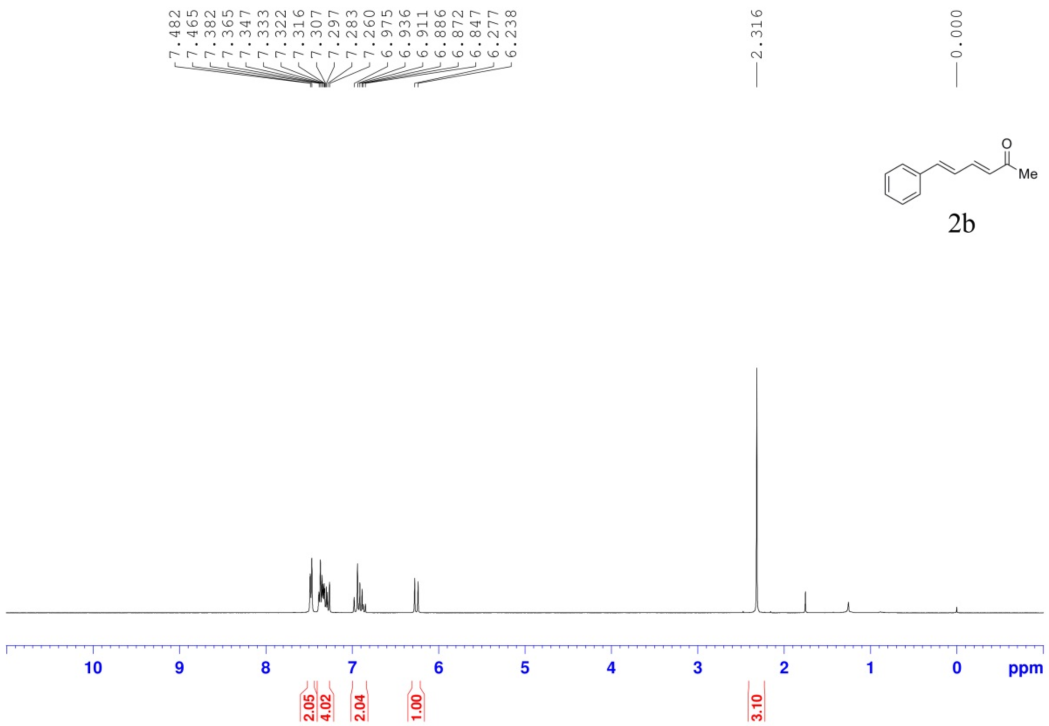

Supplementary Figure 59. ^{1}H NMR spectrum for (3*E*,5*E*)-6-phenylhexa-3,5-dien-2-one **(2b)**.

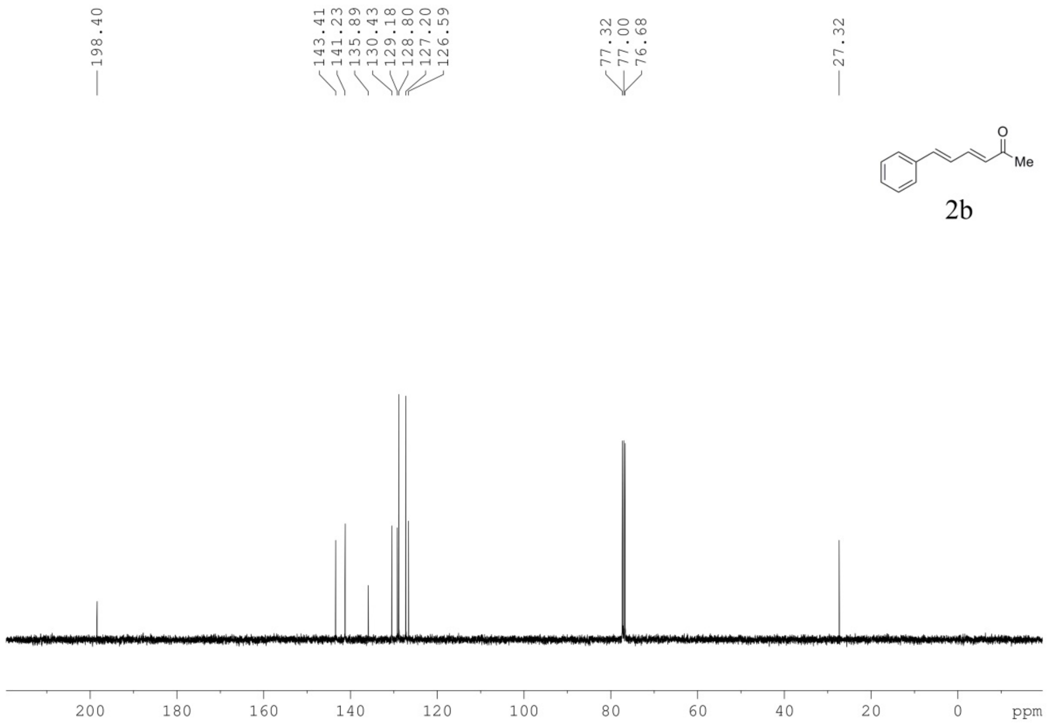

Supplementary Figure 60. ^{13}C NMR spectrum for (3*E*,5*E*)-6-phenylhexa-3,5-dien-2-one **(2b)**.

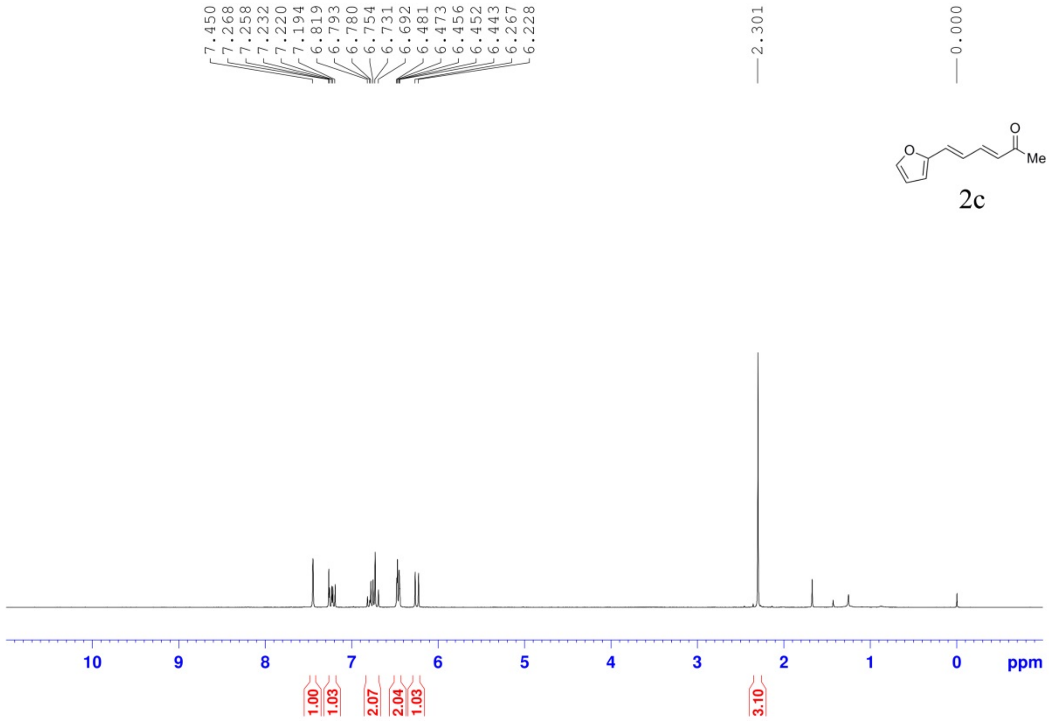

Supplementary Figure 61. ^{1}H NMR spectrum for (3*E*,5*E*)-6-(furan-2-yl)hexa-3,5-dien-2-one **(2c)**.

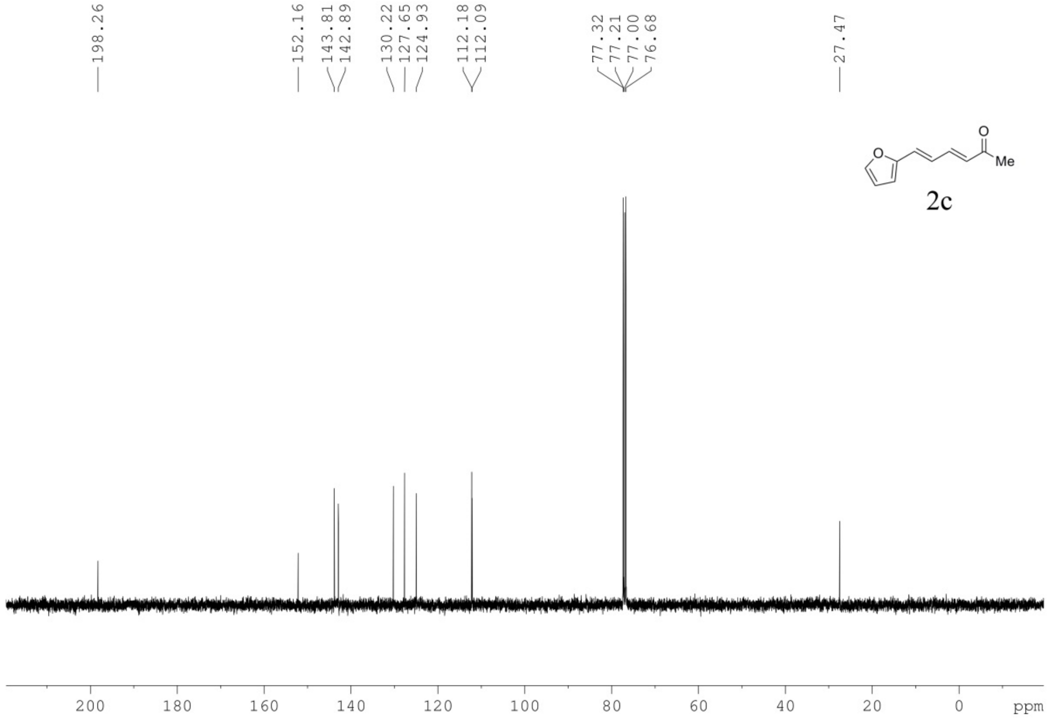

Supplementary Figure 62. ^{13}C NMR spectrum for (3*E*,5*E*)-6-(furan-2-yl)hexa-3,5-dien-2-one **(2c)**.

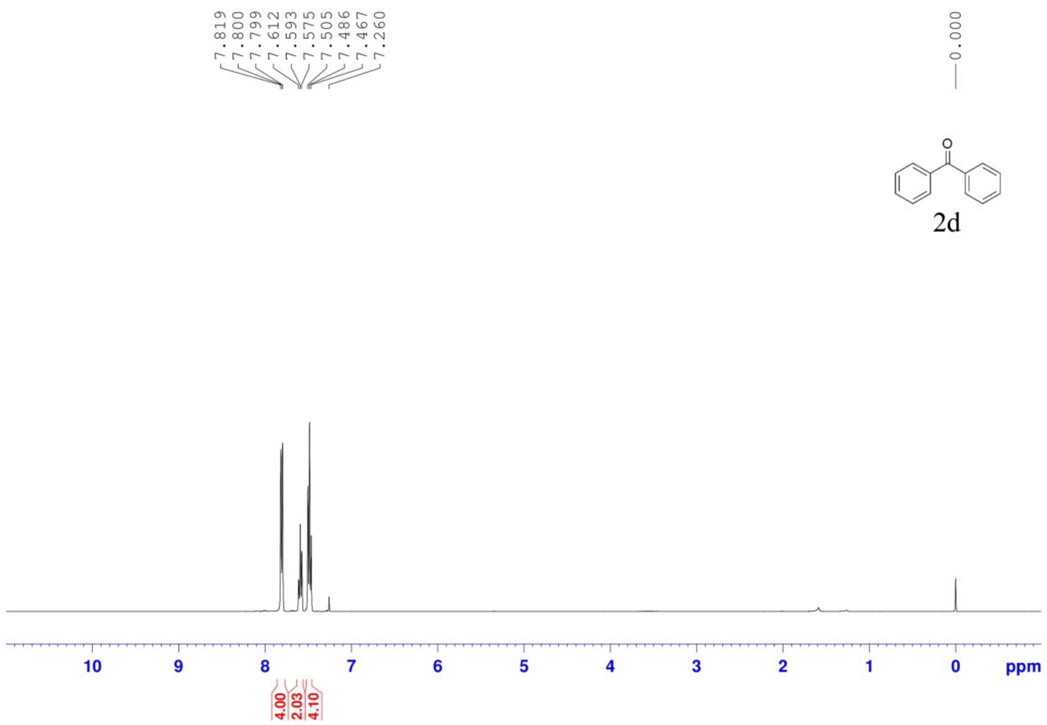

Supplementary Figure 63. ^{1}H NMR spectrum for benzophenone **(2d)**.

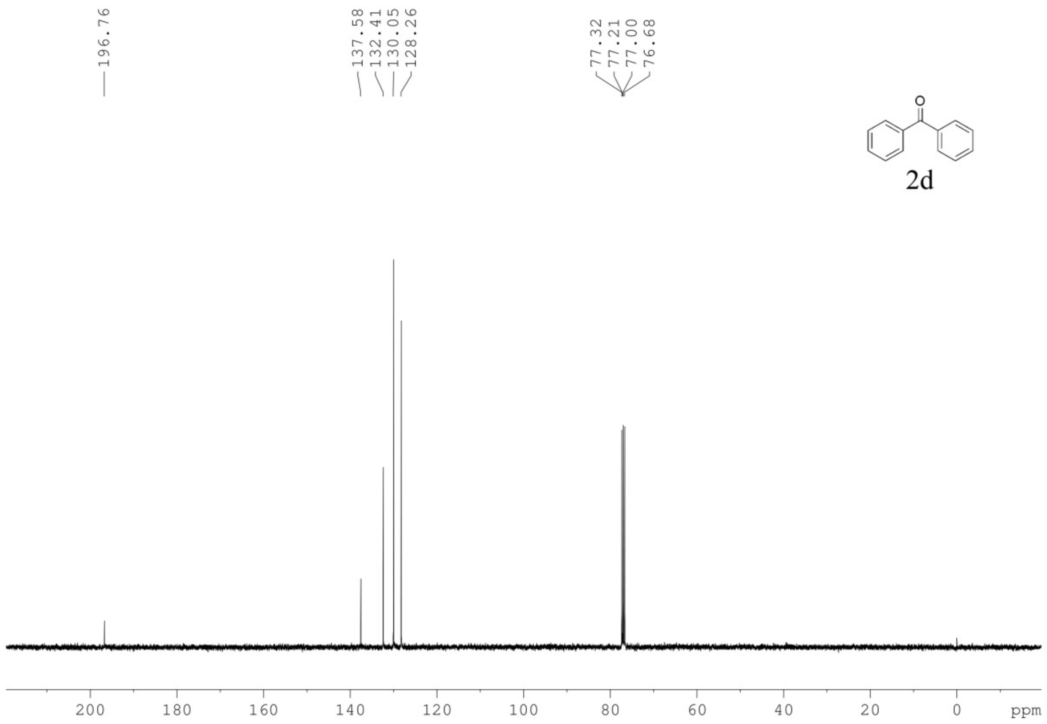

Supplementary Figure 64. ^{13}C NMR spectrum for benzophenone **(2d)**.

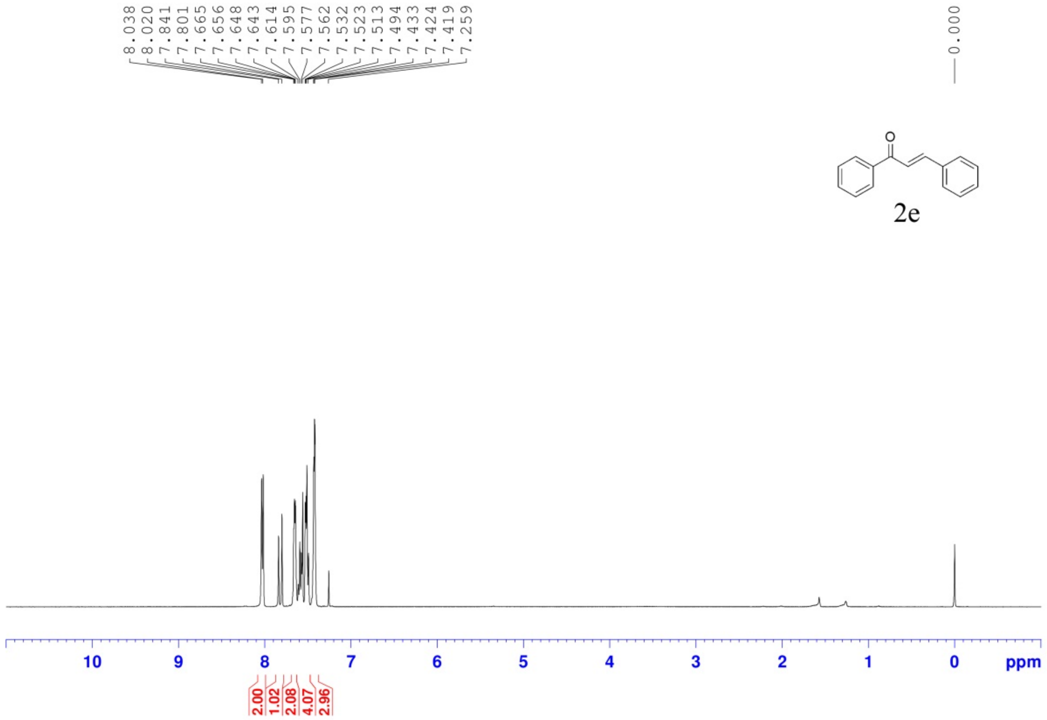

Supplementary Figure 65. ^{1}H NMR spectrum for (*E*)-chalcone **(2e)**.

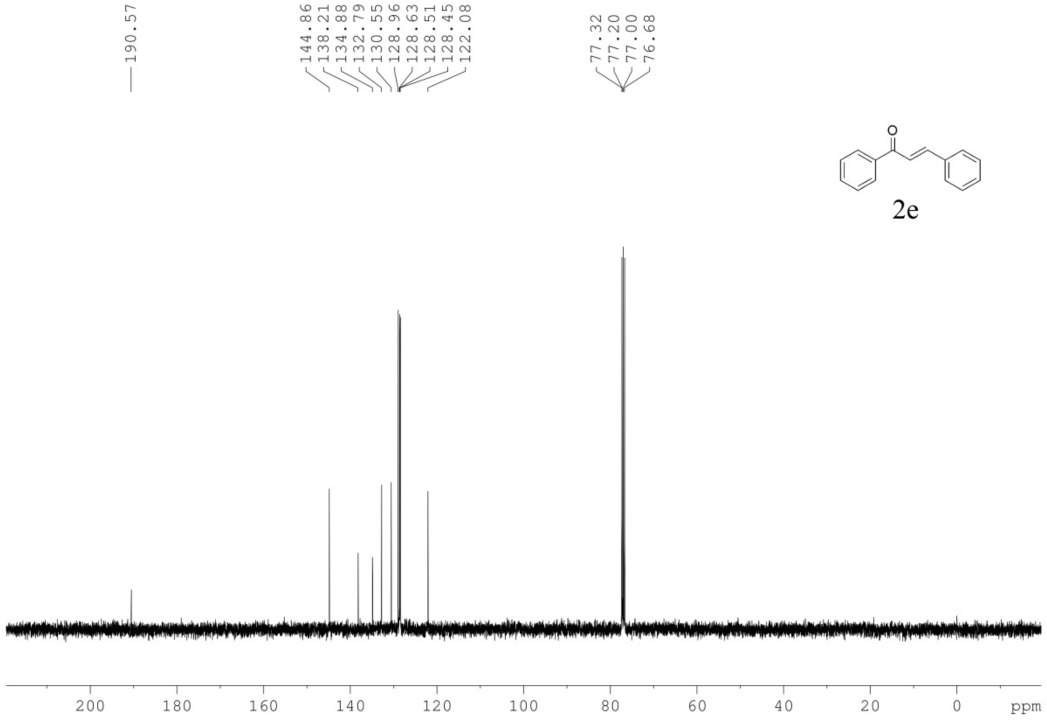

Supplementary Figure 66. ^{13}C NMR spectrum for (*E*)-chalcone **(2e)**.

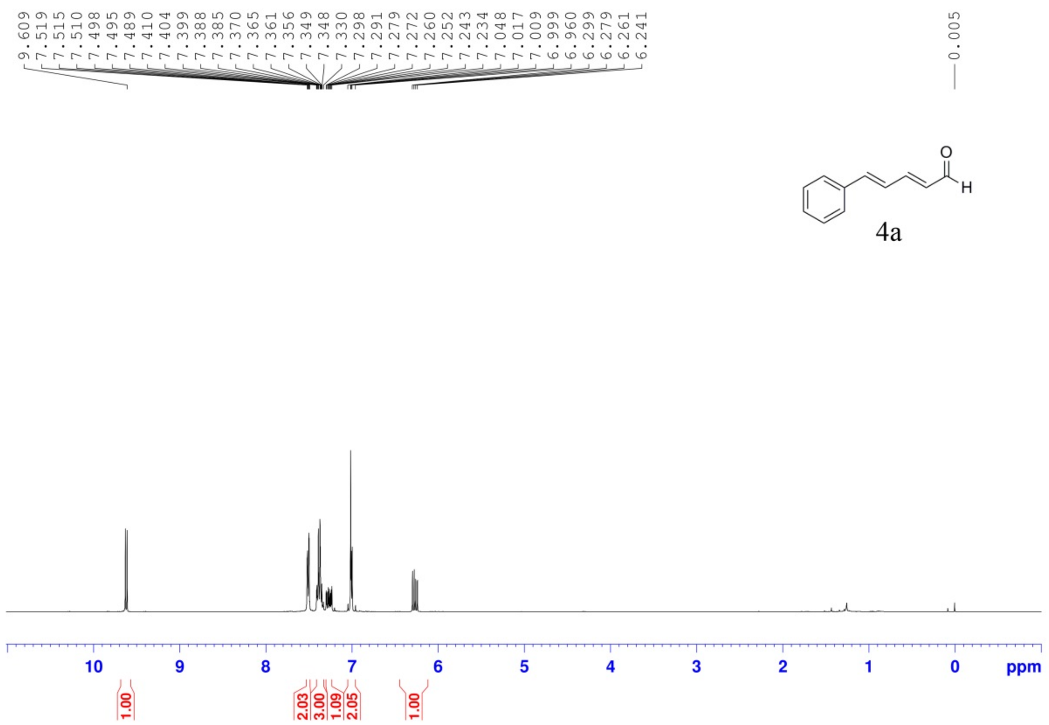

Supplementary Figure 67. ^{1}H NMR spectrum for (2*E*,4*E*)-5-phenylpenta-2,4-dienal **(4a)**.

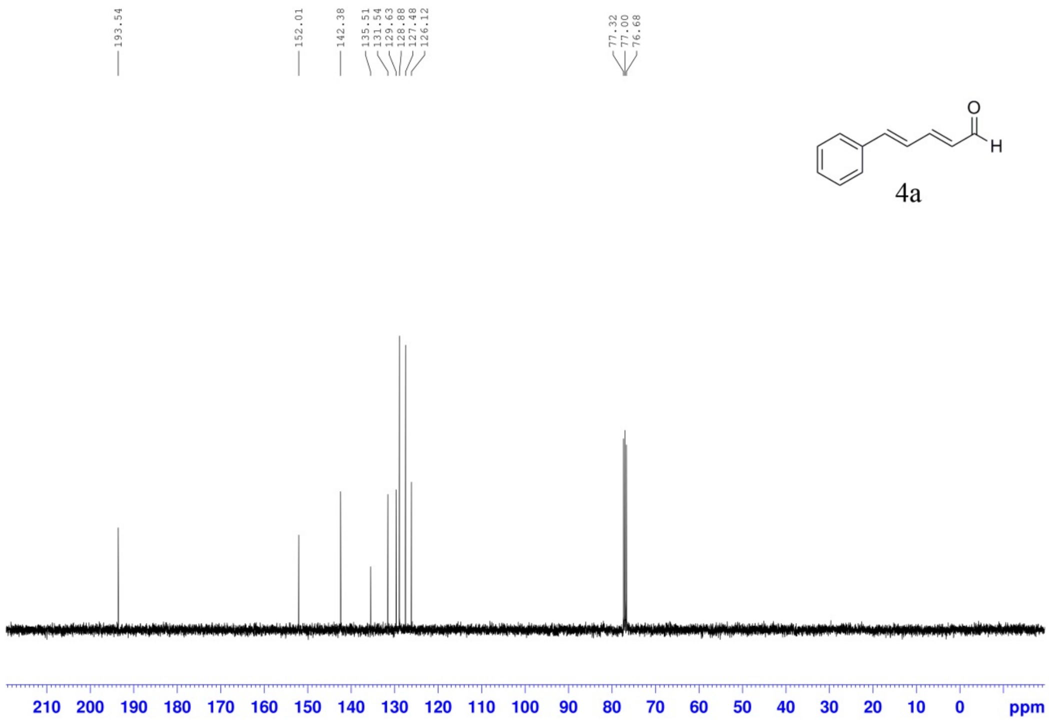

Supplementary Figure 68. ^{13}C NMR spectrum for (2*E*,4*E*)-5-phenylpenta-2,4-dienal **(4a)**.

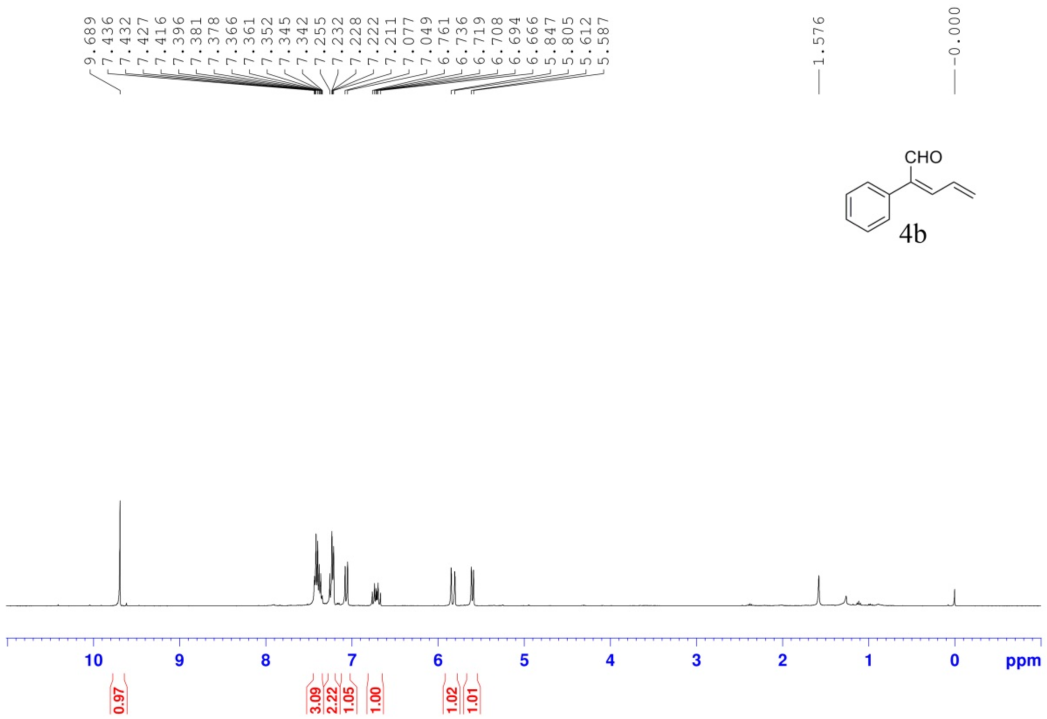

Supplementary Figure 69. ^{1}H NMR spectrum for (*Z*)-2-phenylpenta-2,4-dienal **(4b)**.

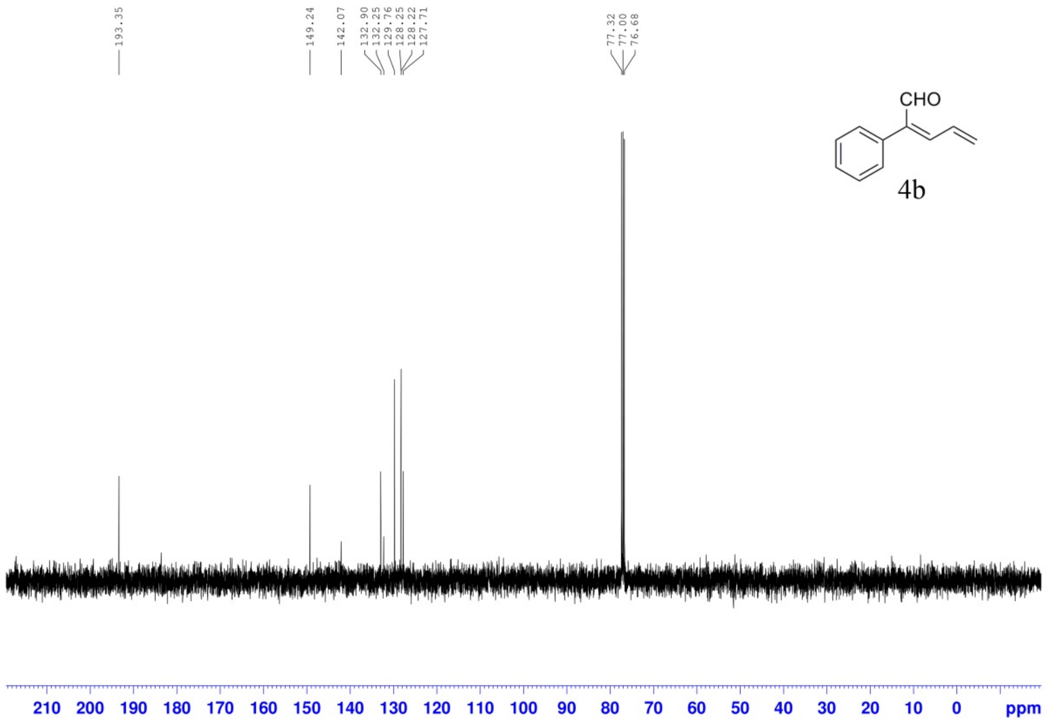

Supplementary Figure 70. ^{13}C NMR spectrum for (*Z*)-2-phenylpenta-2,4-dienal **(4b)**.

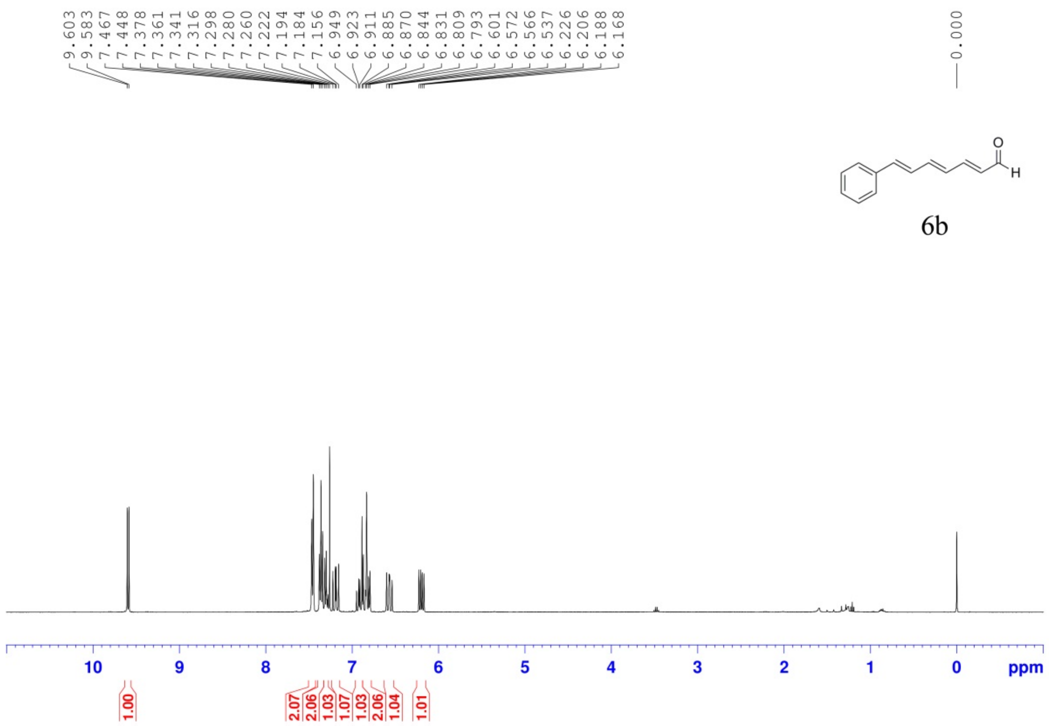

6b

Supplementary Figure 71. ^{1}H NMR spectrum for (2*E*,4*E*,6*E*)-7-phenylhepta-2,4,6-trienal **(6b)**.

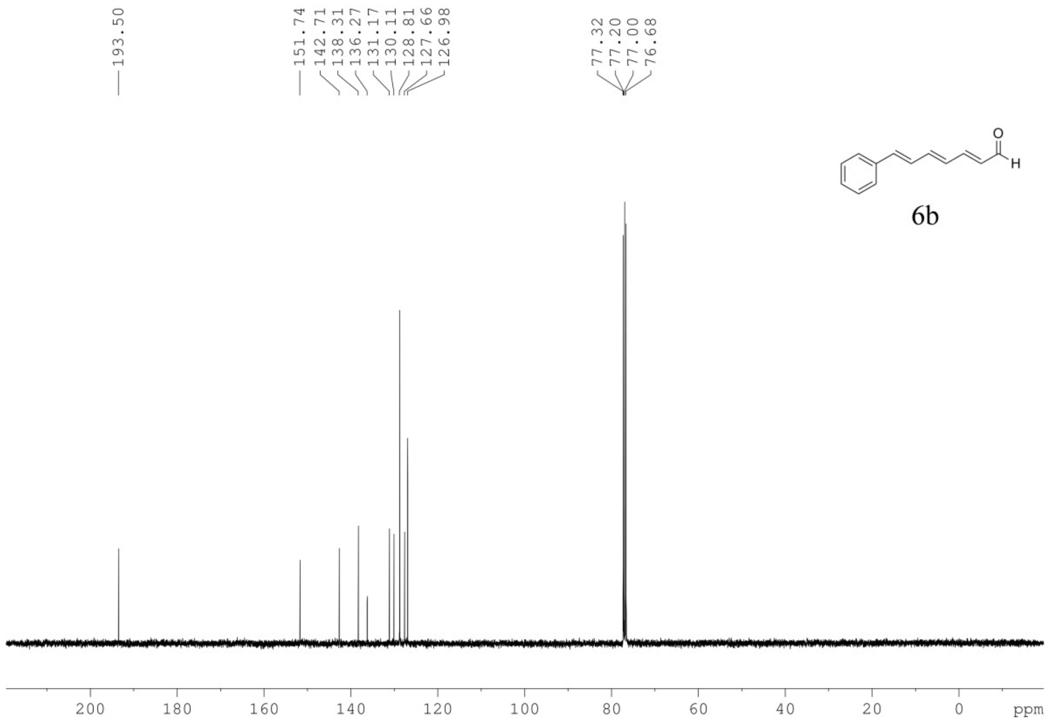

6b

Supplementary Figure 72. ^{13}C NMR spectrum for (2*E*,4*E*,6*E*)-7-phenylhepta-2,4,6-trienal **(6b)**.

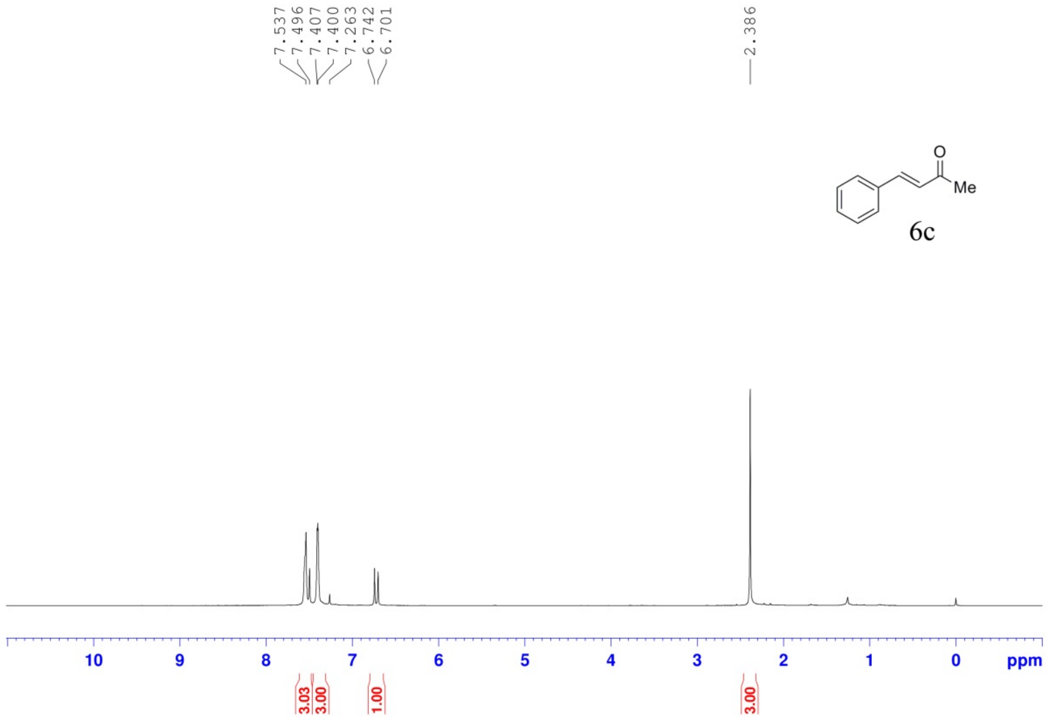

Supplementary Figure 73. ^{1}H NMR spectrum for (*E*)-4-phenylbut-3-en-2-one **(6c)**.

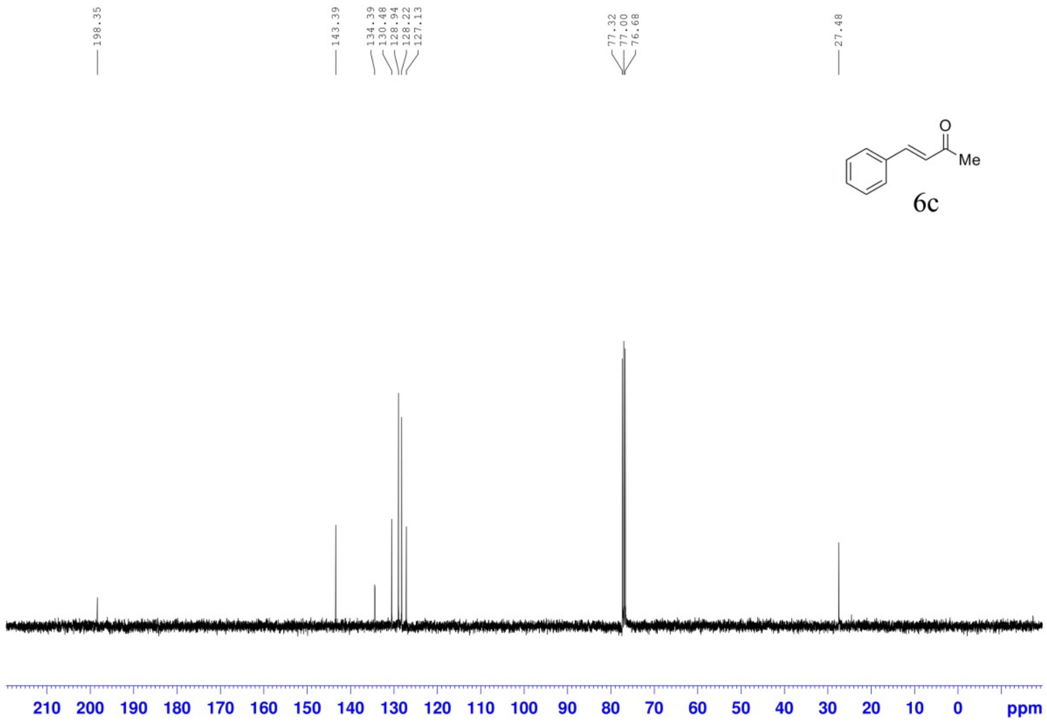

Supplementary Figure 74. ^{13}C NMR spectrum for (*E*)-4-phenylbut-3-en-2-one **(6c)**.

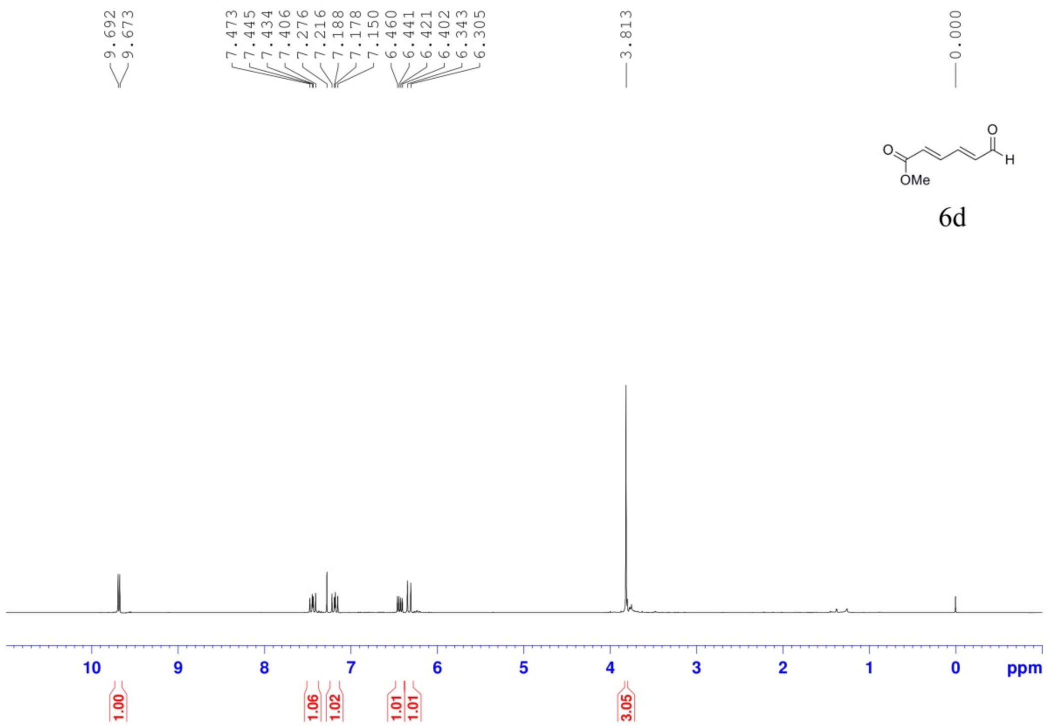

Supplementary Figure 75. ^{1}H NMR spectrum for methyl (2*E*,4*E*)-6-oxohexa-2,4-dienoate **(6d)**.

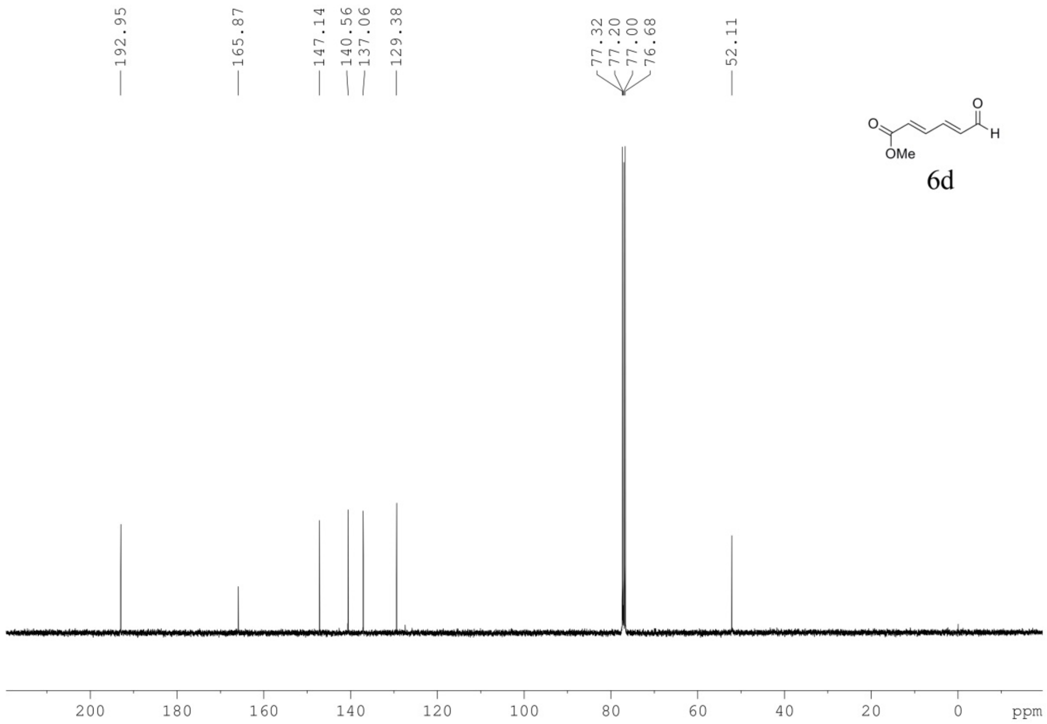

Supplementary Figure 76. ^{13}C NMR spectrum for methyl (2*E*,4*E*)-6-oxohexa-2,4-dienoate **(6d)**.

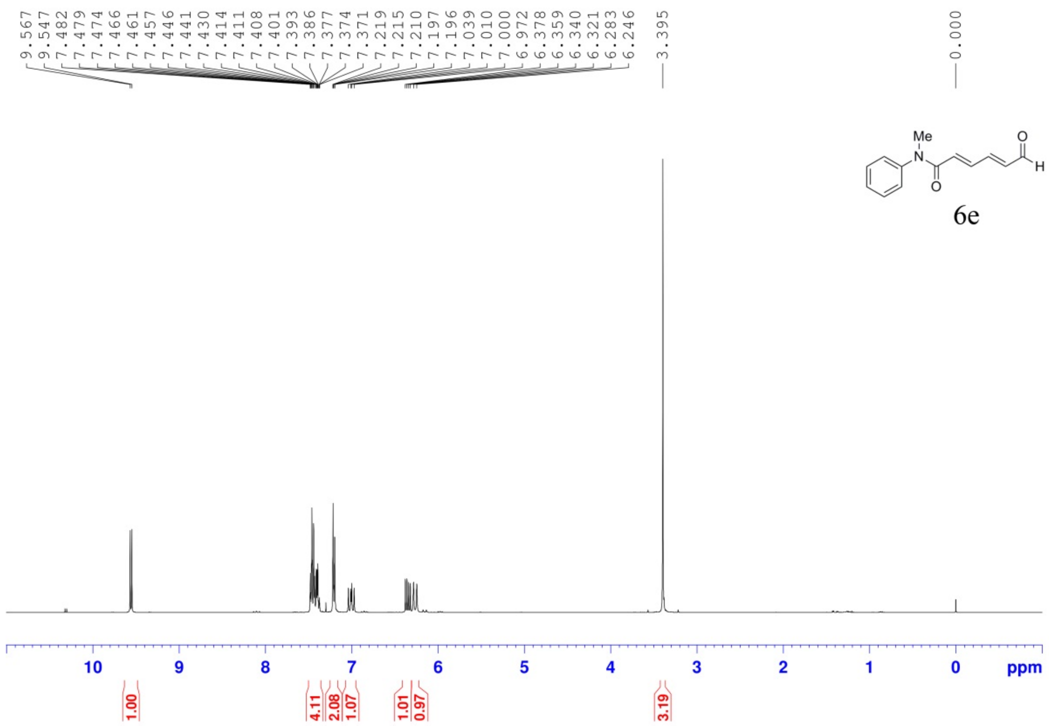

Supplementary Figure 77. ^{1}H NMR spectrum for (2*E*,4*E*)-*N*-methyl-6-oxo-*N*-phenylhexa-2,4-dienamide **(6e)**.

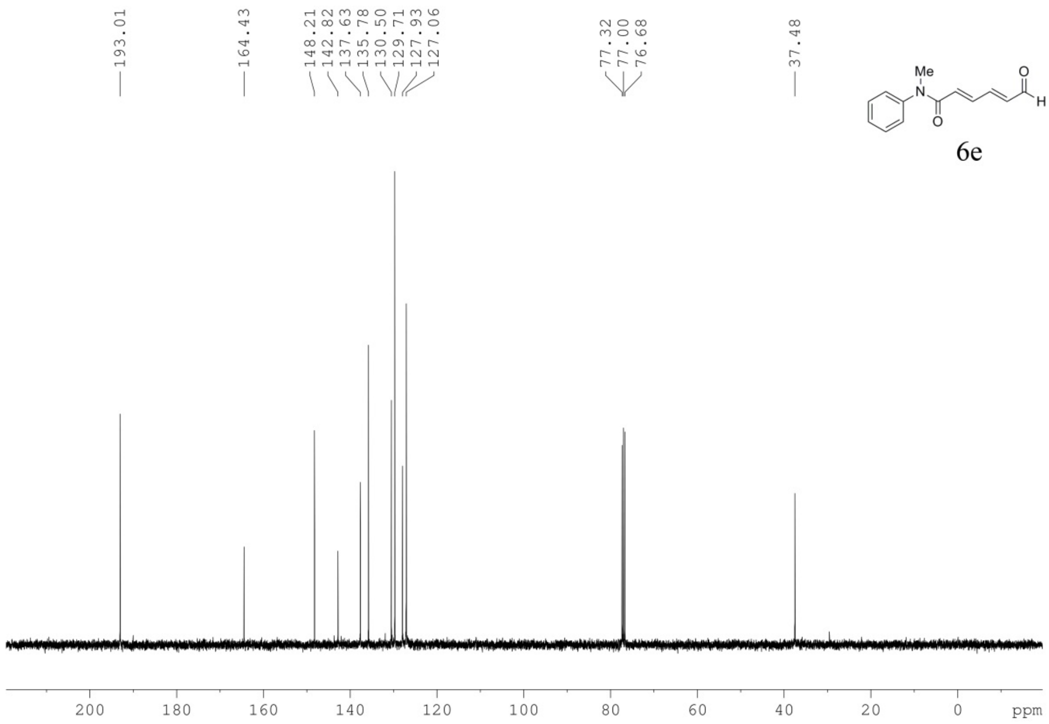

Supplementary Figure 78. ^{13}C NMR spectrum for (2*E*,4*E*)-*N*-methyl-6-oxo-*N*-phenylhexa-2,4-dienamide **(6e)**.

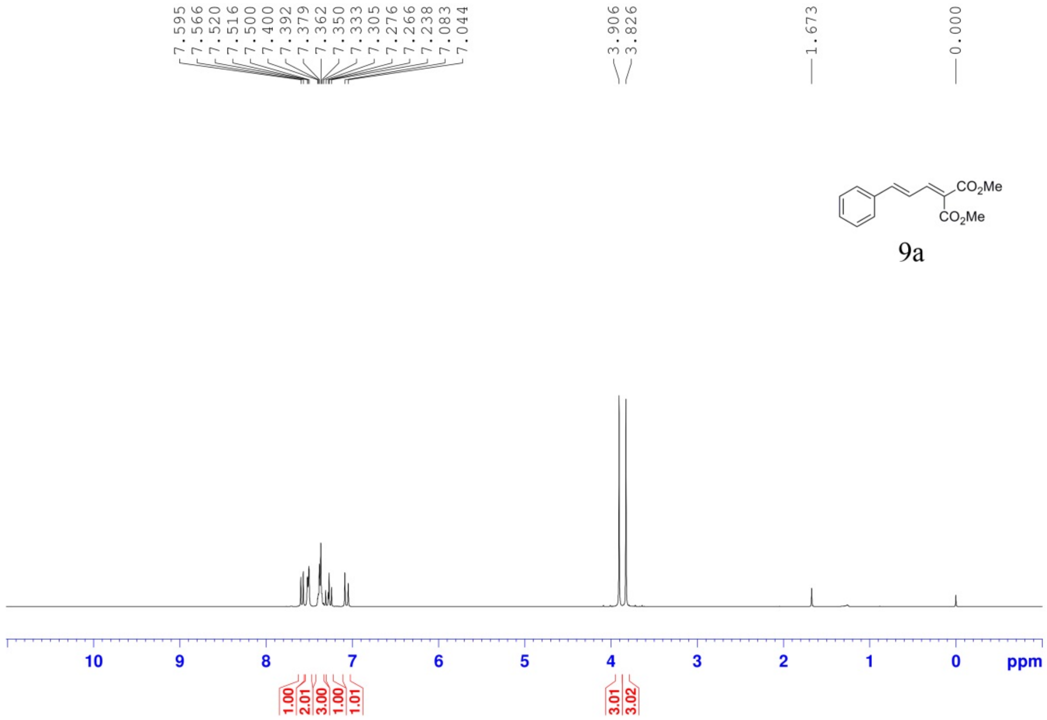

Supplementary Figure 79. ^{1}H NMR spectrum for dimethyl (*E*)-2-(3-phenylallylidene)malonate **(9a)**.

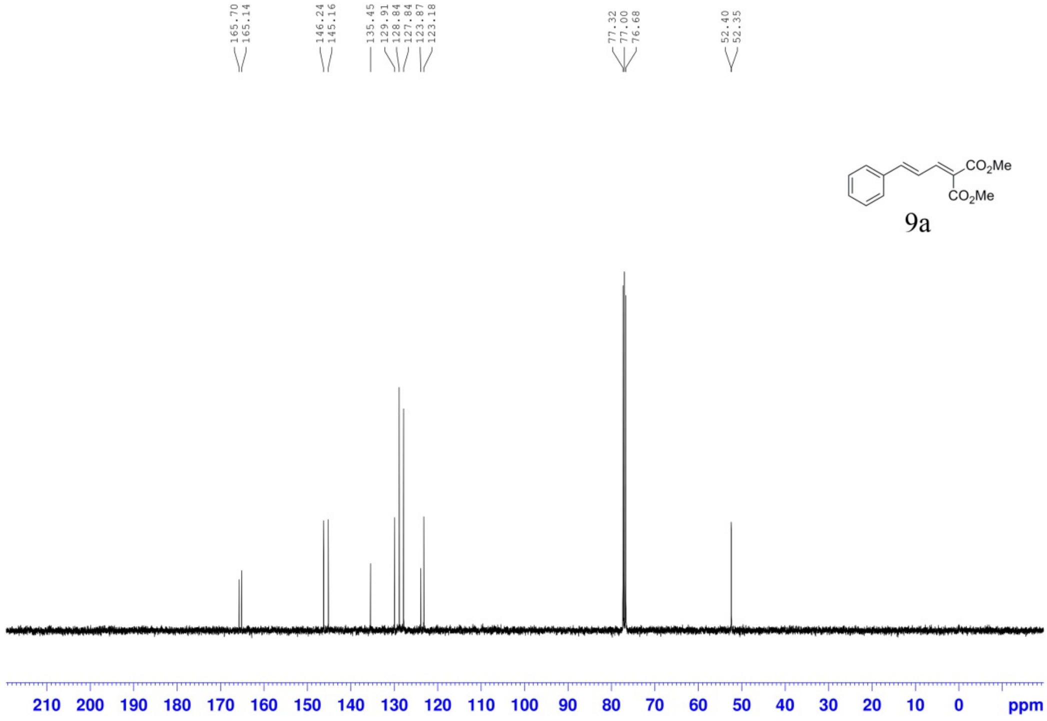

Supplementary Figure 80. ^{13}C NMR spectrum for dimethyl (*E*)-2-(3-phenylallylidene)malonate **(9a)**.

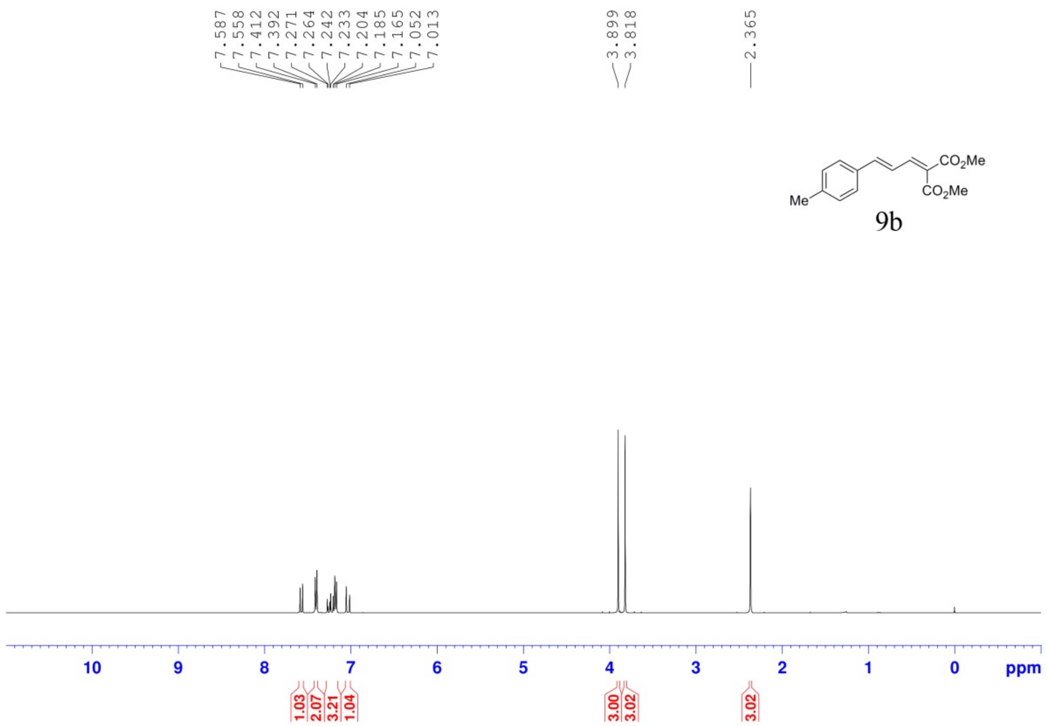

Supplementary Figure 81. ^{1}H NMR spectrum for dimethyl (*E*)-2-(3-(p-tolyl)allylidene)malonate **(9b)**.

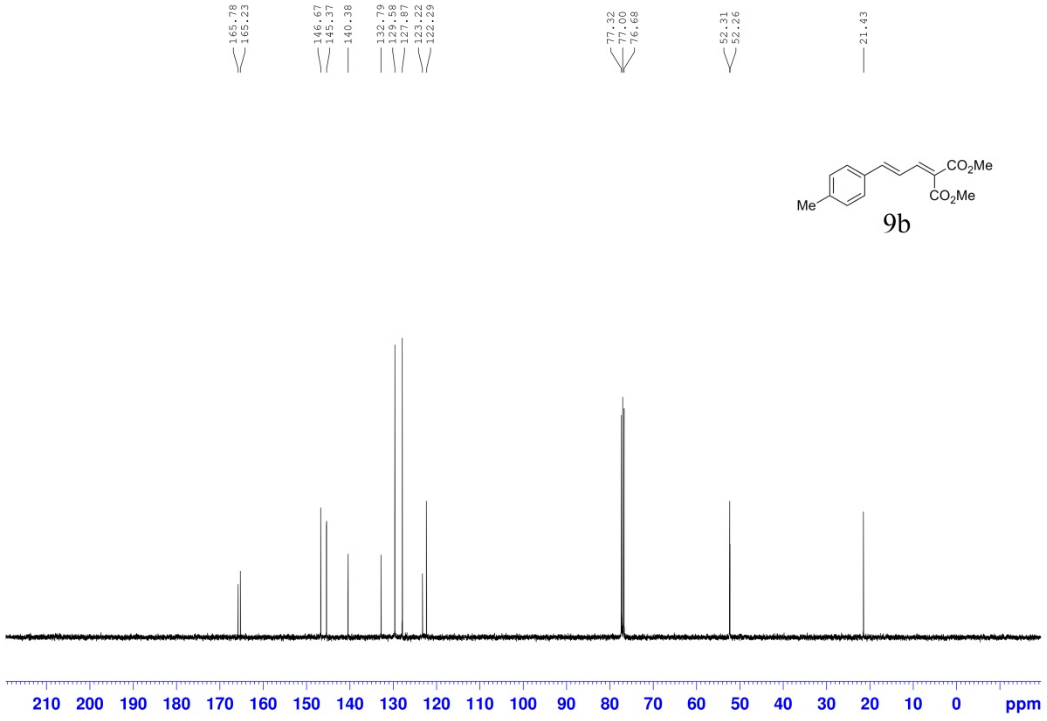

Supplementary Figure 82. ^{13}C NMR spectrum for dimethyl (*E*)-2-(3-(p-tolyl)allylidene)malonate **(9b)**.

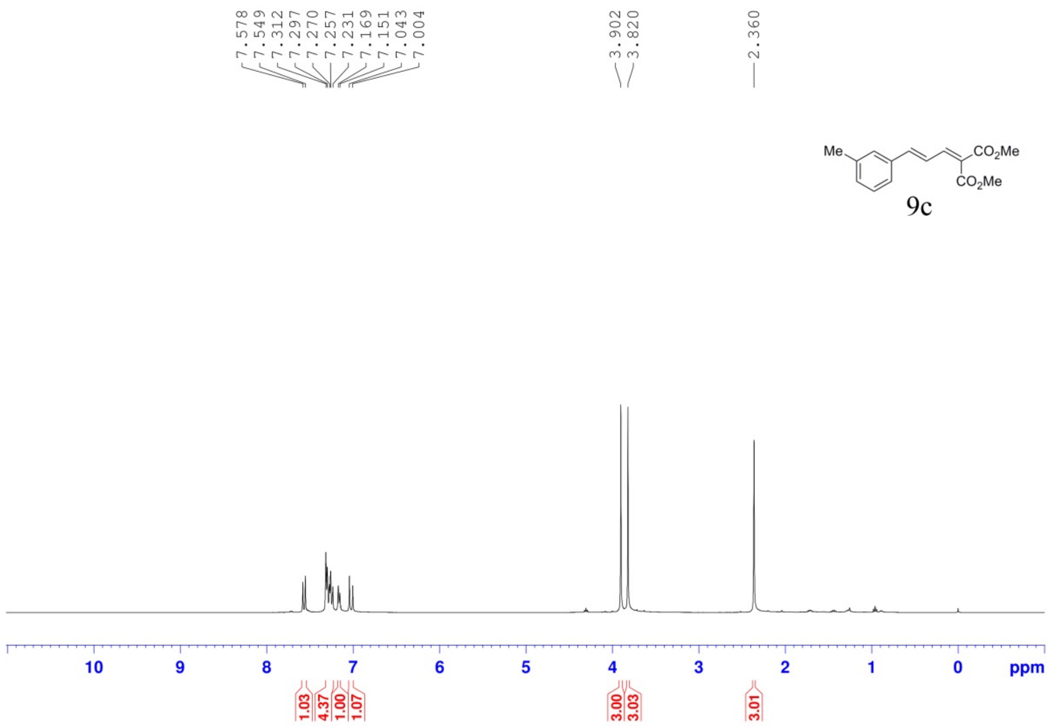

Supplementary Figure 83. ^{1}H NMR spectrum for dimethyl (*E*)-2-(3-(m-tolyl)allylidene)malonate **(9c)**.

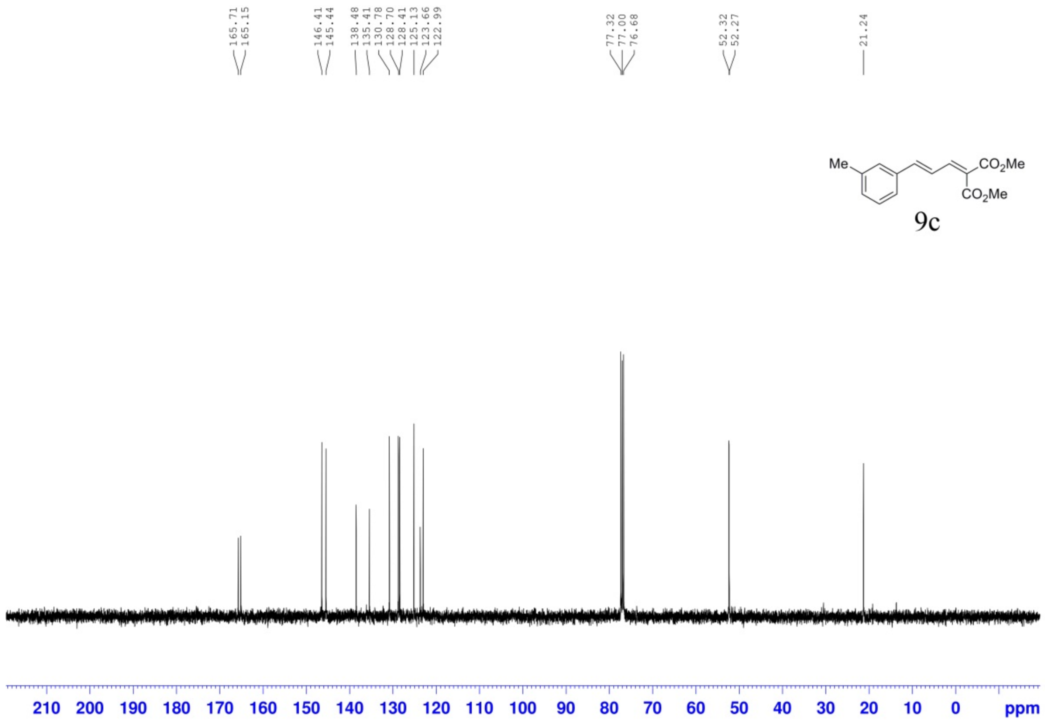

Supplementary Figure 84. ^{13}C NMR spectrum for dimethyl (*E*)-2-(3-(m-tolyl)allylidene)malonate **(9c)**.

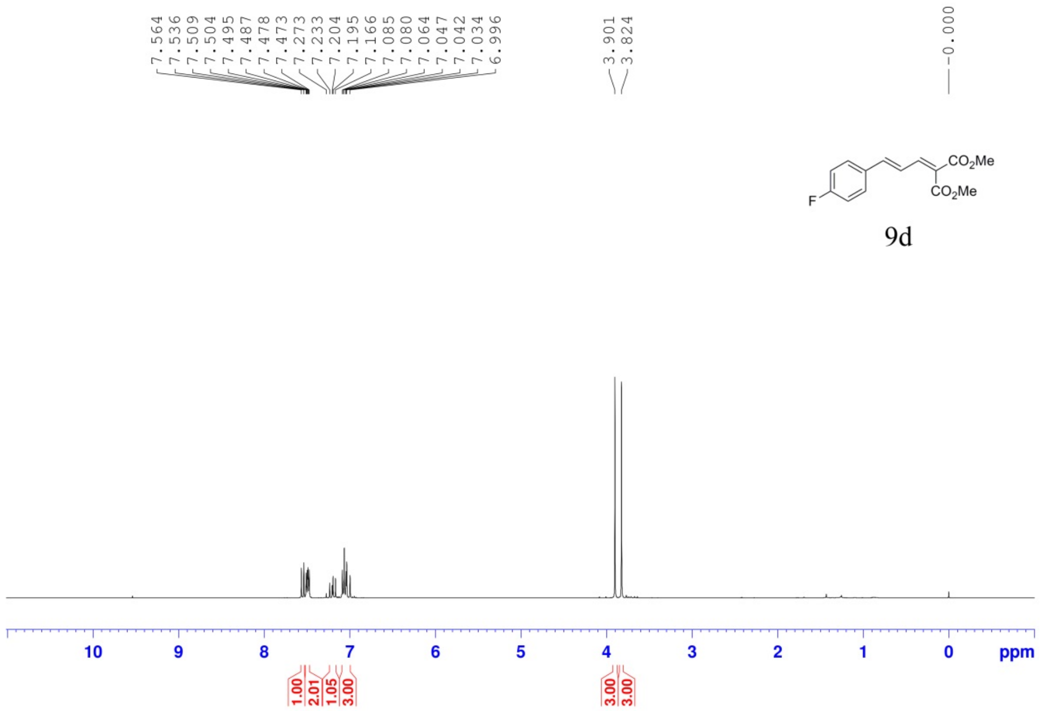

Supplementary Figure 85. ^{1}H NMR spectrum for dimethyl (*E*)-2-(3-(4-fluorophenyl)allylidene)malonate **(9d)**.

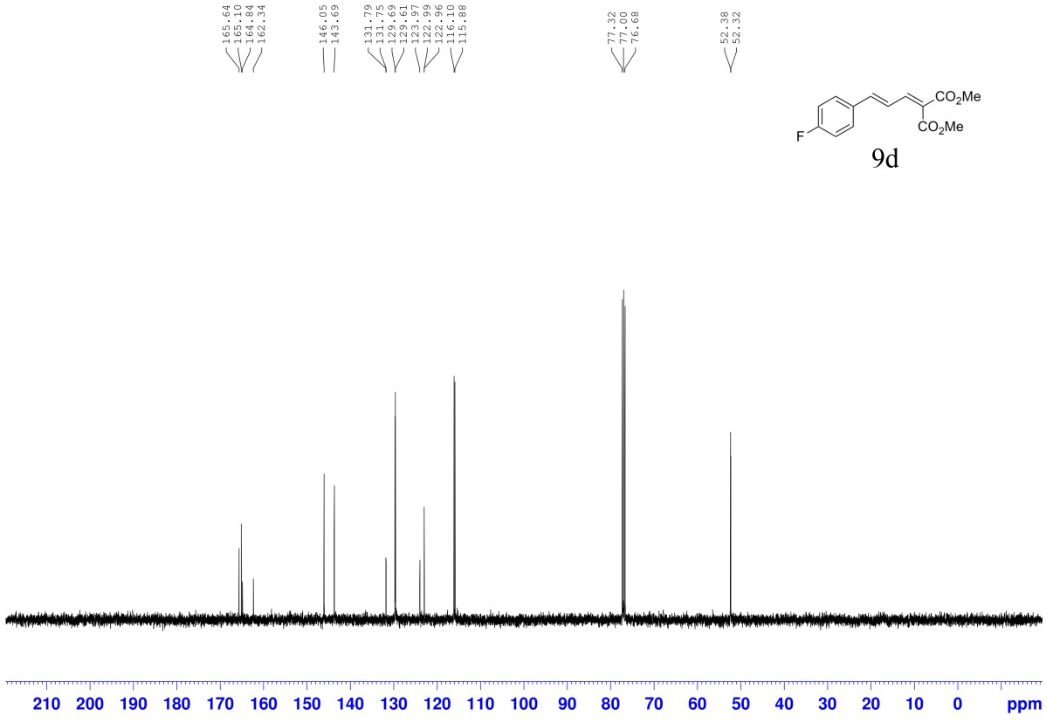

Supplementary Figure 86. ^{13}C NMR spectrum for dimethyl (*E*)-2-(3-(4-fluorophenyl)allylidene)malonate **(9d)**.

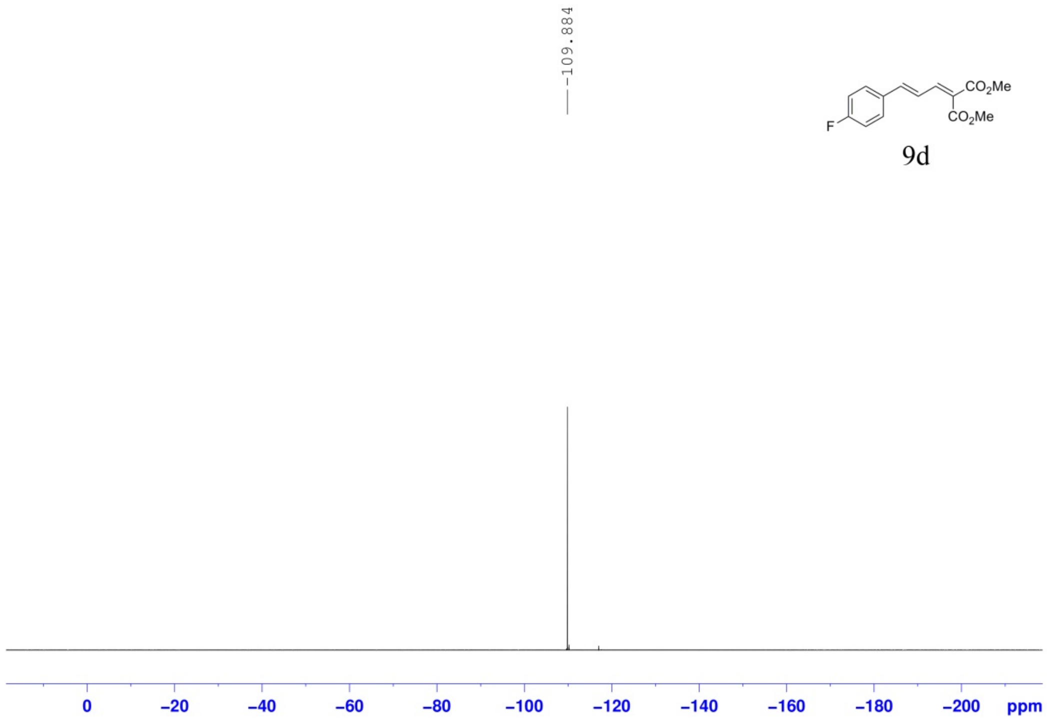

Supplementary Figure 87. ^{19}F NMR spectrum for dimethyl (*E*)-2-(3-(4-fluorophenyl)allylidene)malonate **(9d)**.

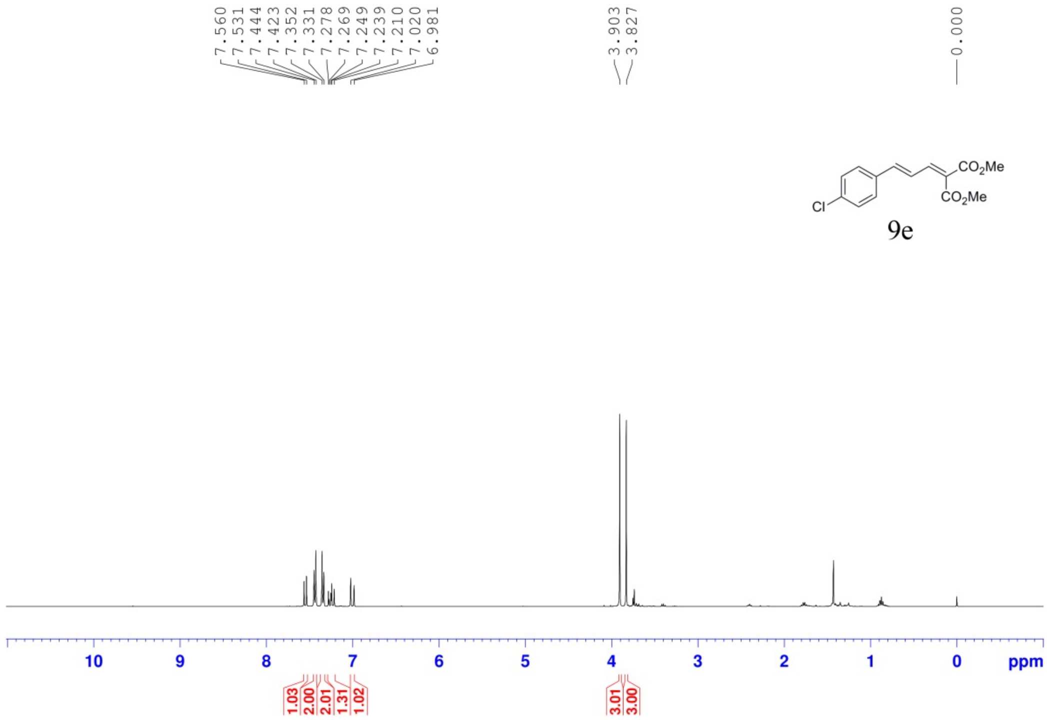

Supplementary Figure 88. ^{1}H NMR spectrum for dimethyl (*E*)-2-(3-(4-chlorophenyl)allylidene)malonate **(9e)**.

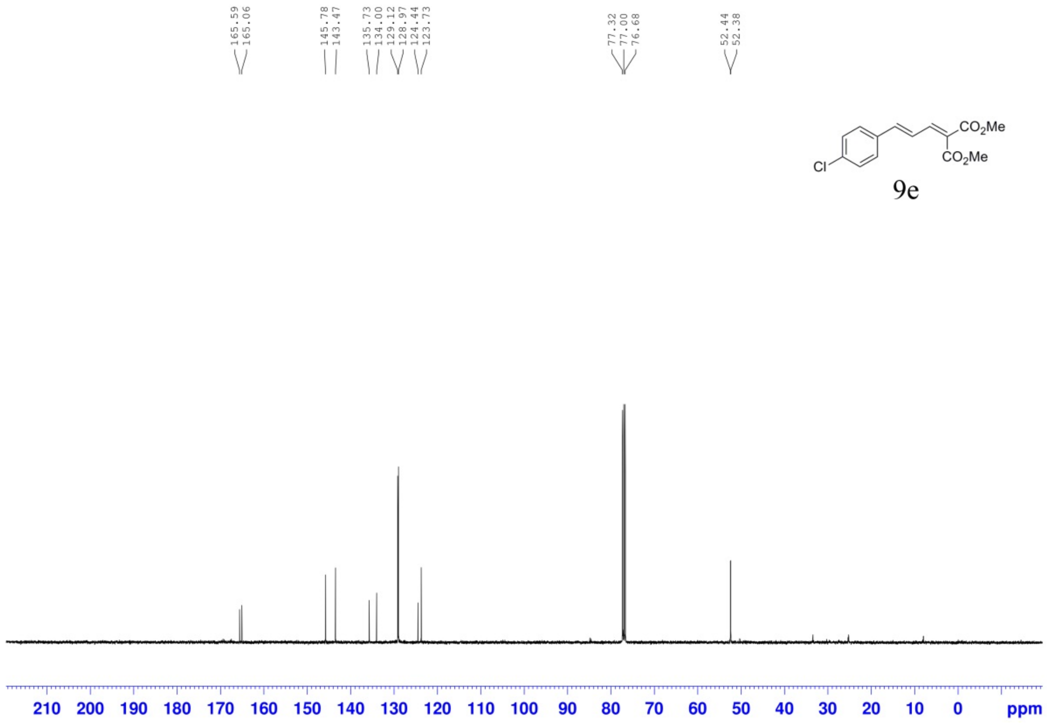

Supplementary Figure 89. ^{13}C NMR spectrum for dimethyl (*E*)-2-(3-(4-chlorophenyl)allylidene)malonate **(9e)**.

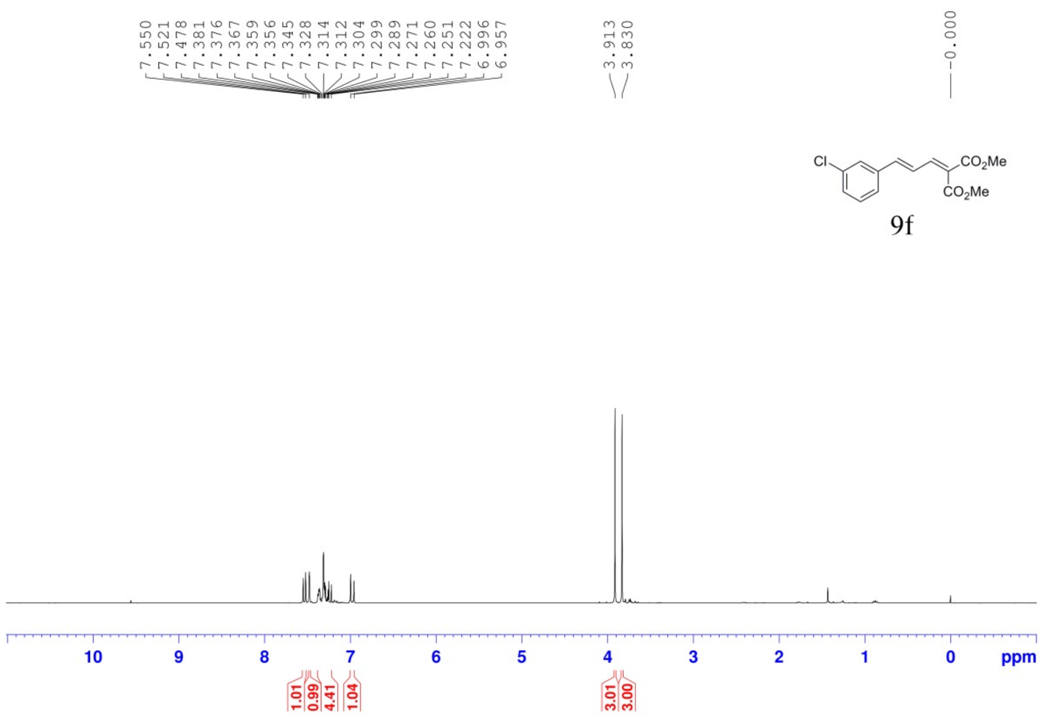

Supplementary Figure 90. ^{1}H NMR spectrum for dimethyl (*E*)-2-(3-(3-chlorophenyl)allylidene)malonate **(9f)**.

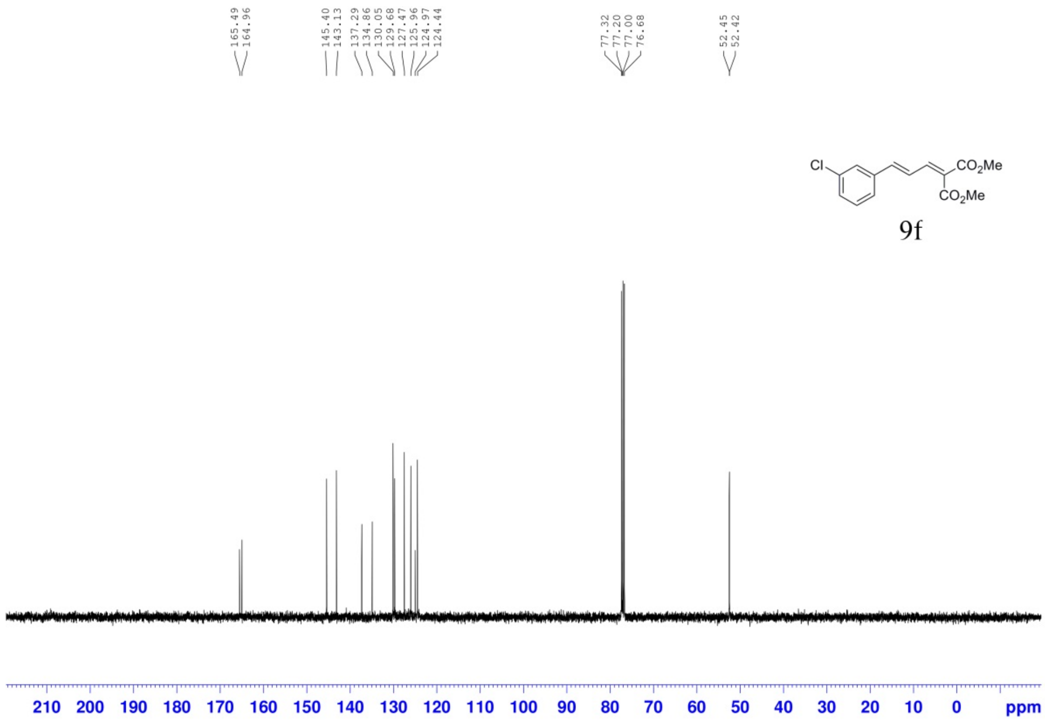

Supplementary Figure 91. ^{13}C NMR spectrum for dimethyl (*E*)-2-(3-(3-chlorophenyl)allylidene)malonate **(9f)**.

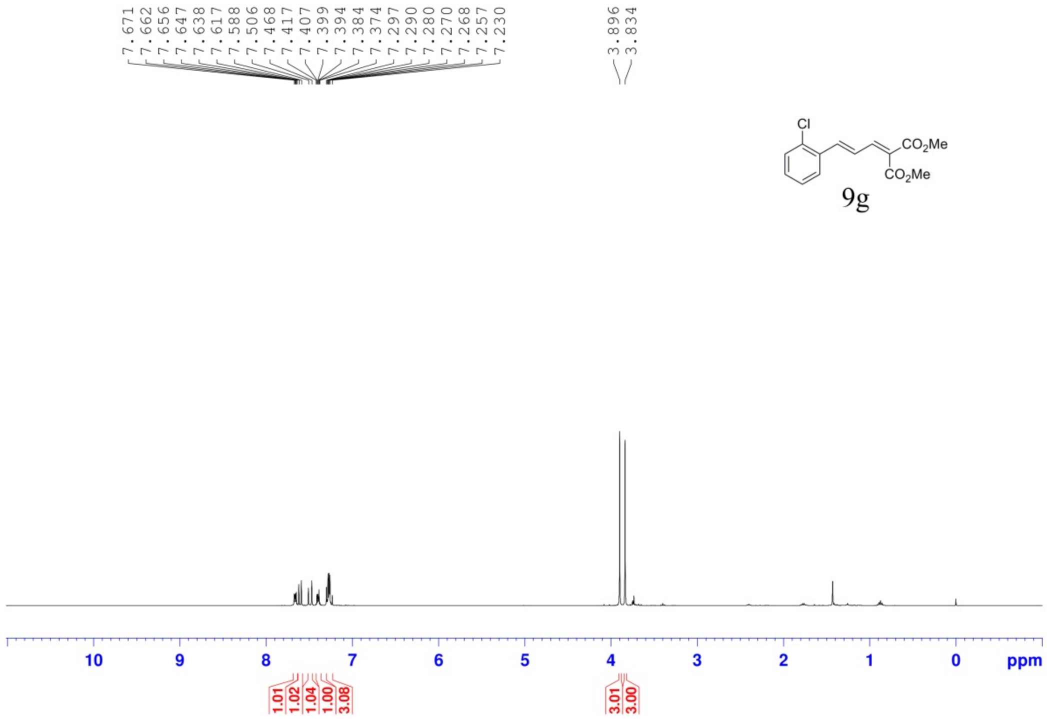

Supplementary Figure 92. ^{1}H NMR spectrum for dimethyl (*E*)-2-(3-(2-chlorophenyl)allylidene)malonate **(9g)**.

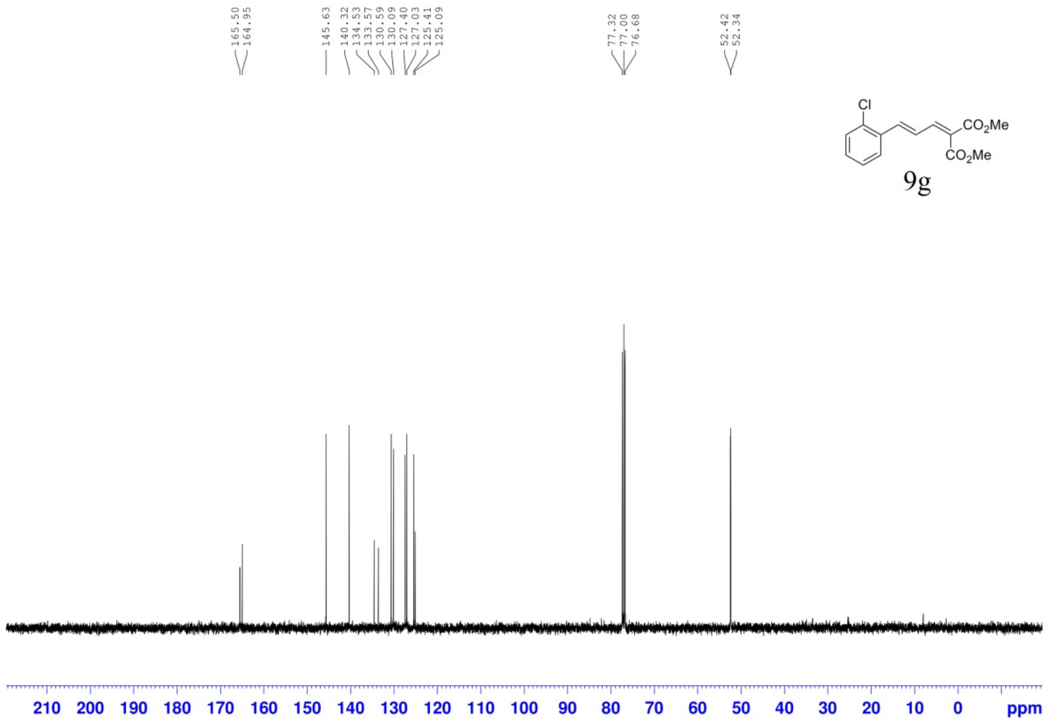

Supplementary Figure 93. ^{13}C NMR spectrum for dimethyl (*E*)-2-(3-(2-chlorophenyl)allylidene)malonate **(9g)**.

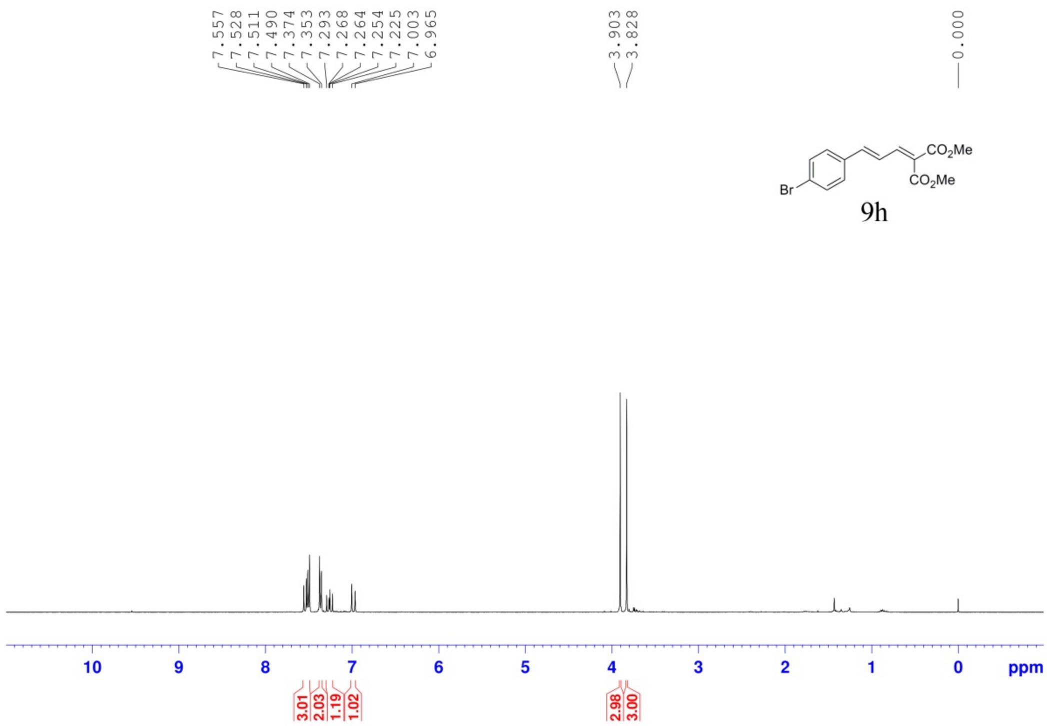

Supplementary Figure 94. ^{1}H NMR spectrum for dimethyl (*E*)-2-(3-(4-bromophenyl)allylidene)malonate **(9h)**.

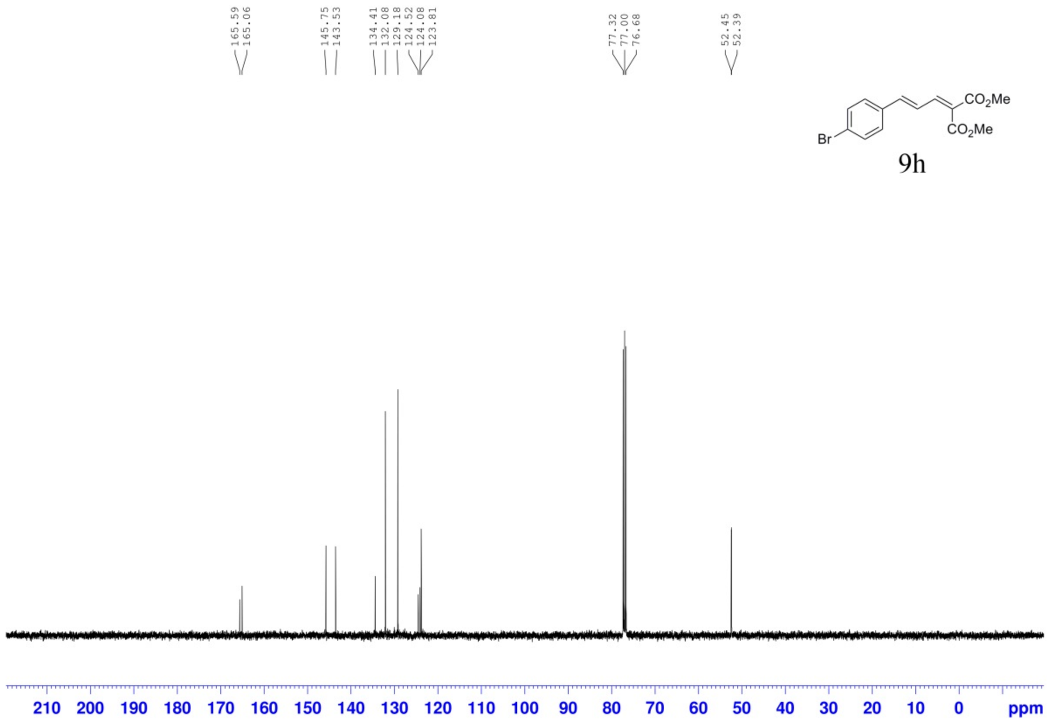

Supplementary Figure 95. ^{13}C NMR spectrum for dimethyl (*E*)-2-(3-(4-bromophenyl)allylidene)malonate **(9h)**.

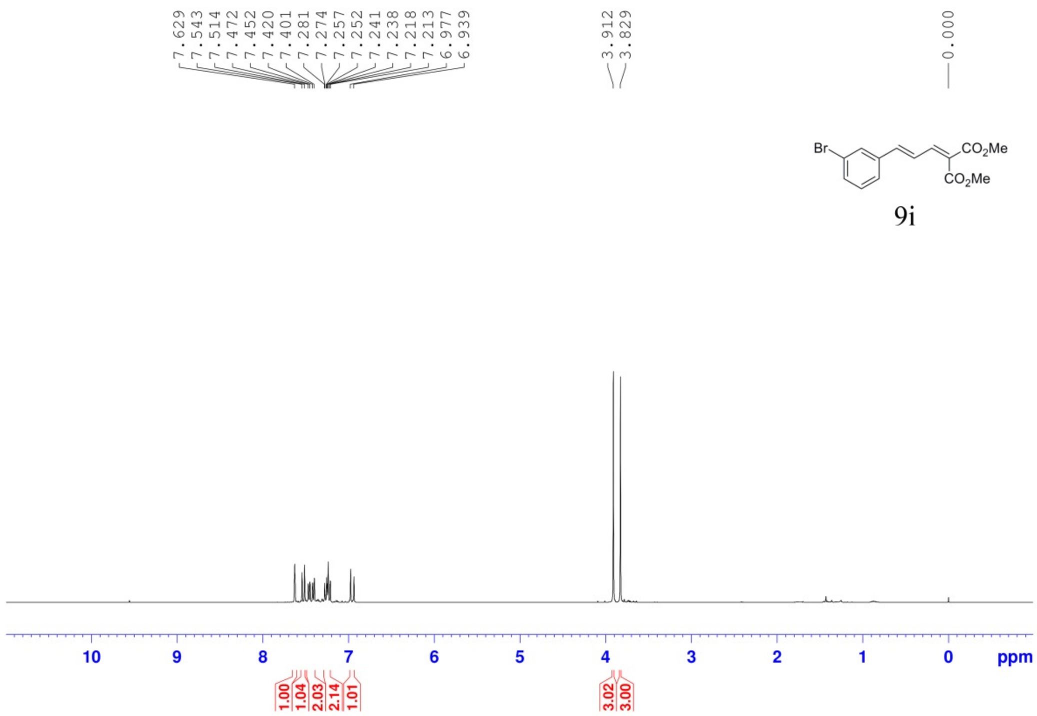

Supplementary Figure 96. ^{1}H NMR spectrum for dimethyl (*E*)-2-(3-(3-bromophenyl)allylidene)malonate **(9i)**.

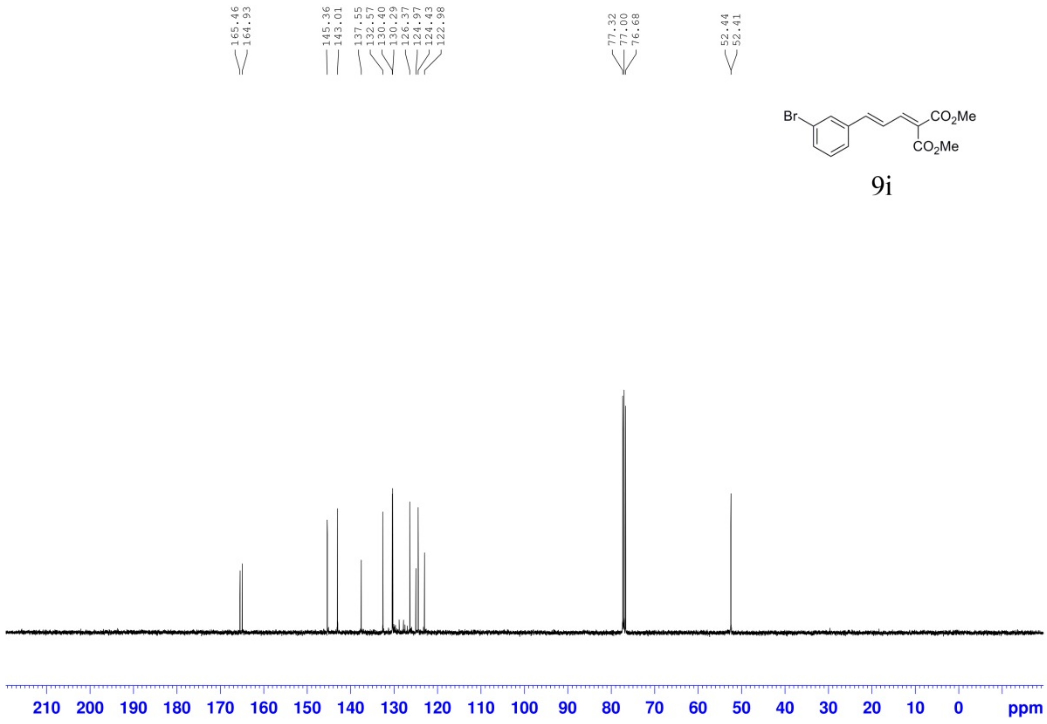

Supplementary Figure 97. ^{13}C NMR spectrum for dimethyl (*E*)-2-(3-(3-bromophenyl)allylidene)malonate **(9i)**.

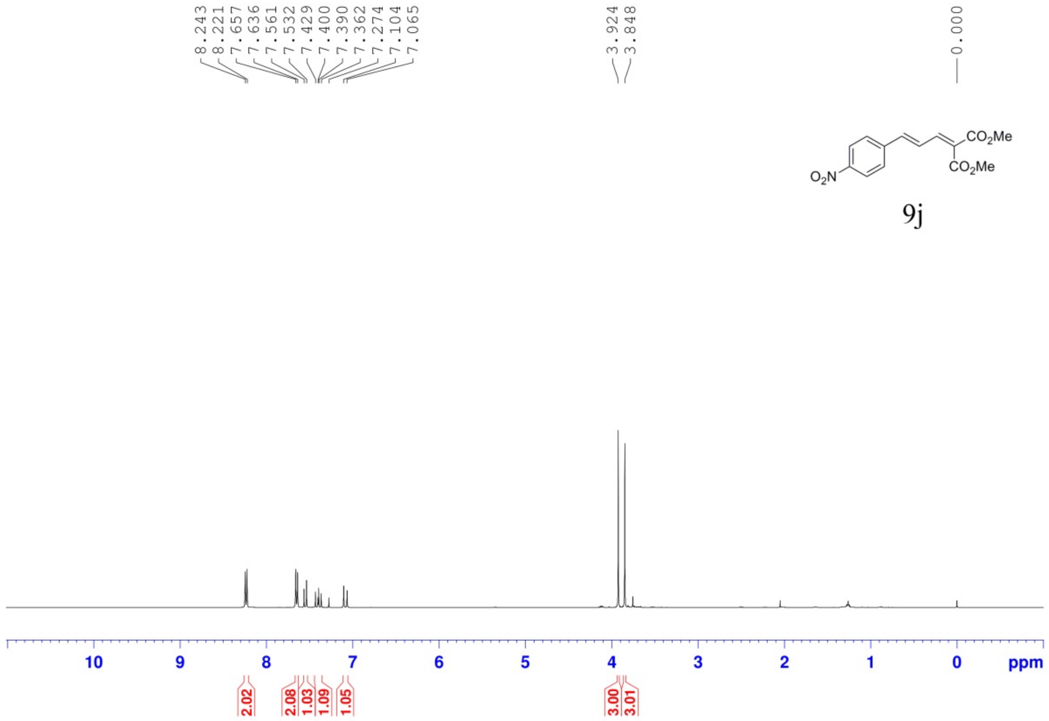

Supplementary Figure 98. ^{1}H NMR spectrum for dimethyl (*E*)-2-(3-(4-nitrophenyl)allylidene)malonate **(9j)**.

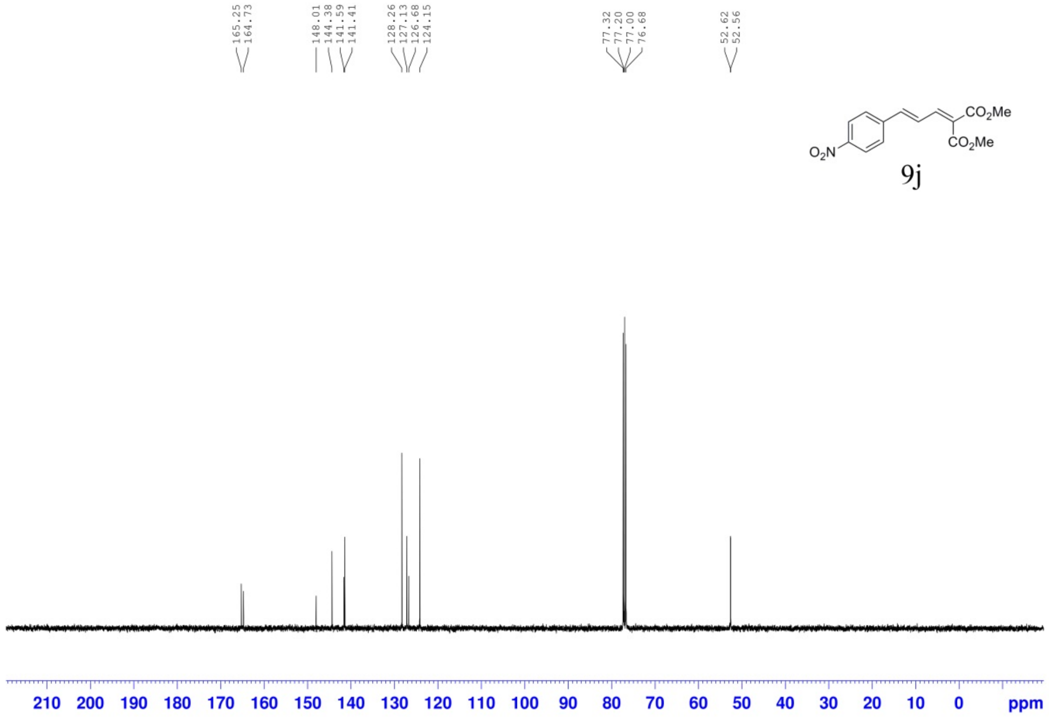

Supplementary Figure 99. ^{13}C NMR spectrum for dimethyl (*E*)-2-(3-(4-nitrophenyl)allylidene)malonate **(9j)**.

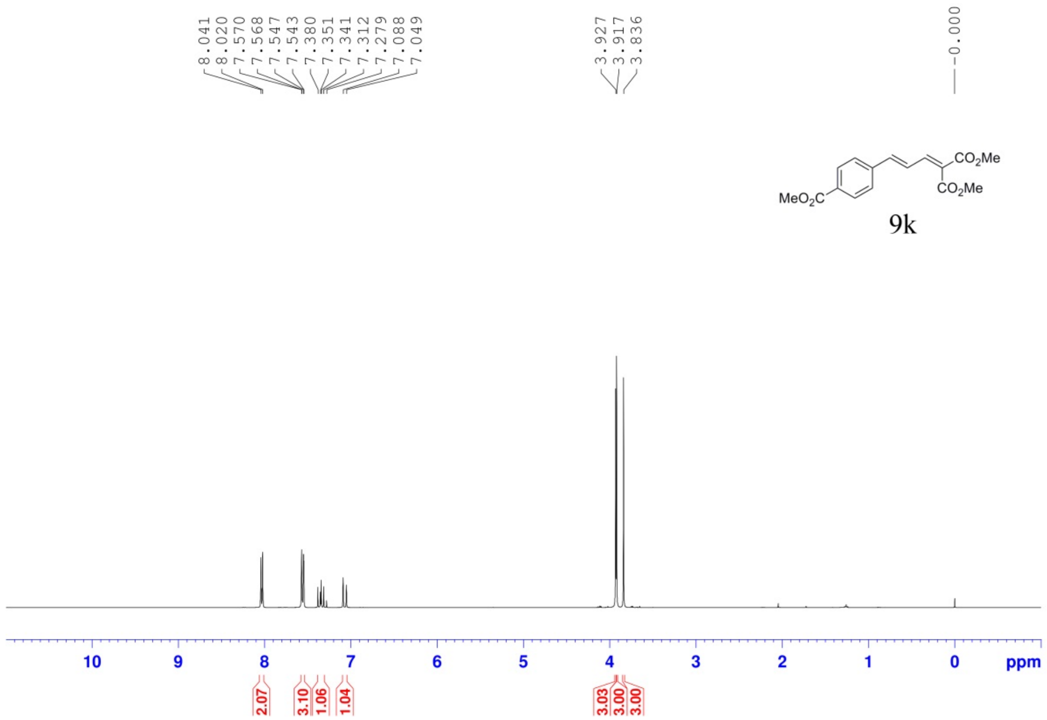

Supplementary Figure 100. ^{1}H NMR spectrum for dimethyl (*E*)-2-(3-(4-(methoxycarbonyl)phenyl)allylidene)malonate **(9k)**.

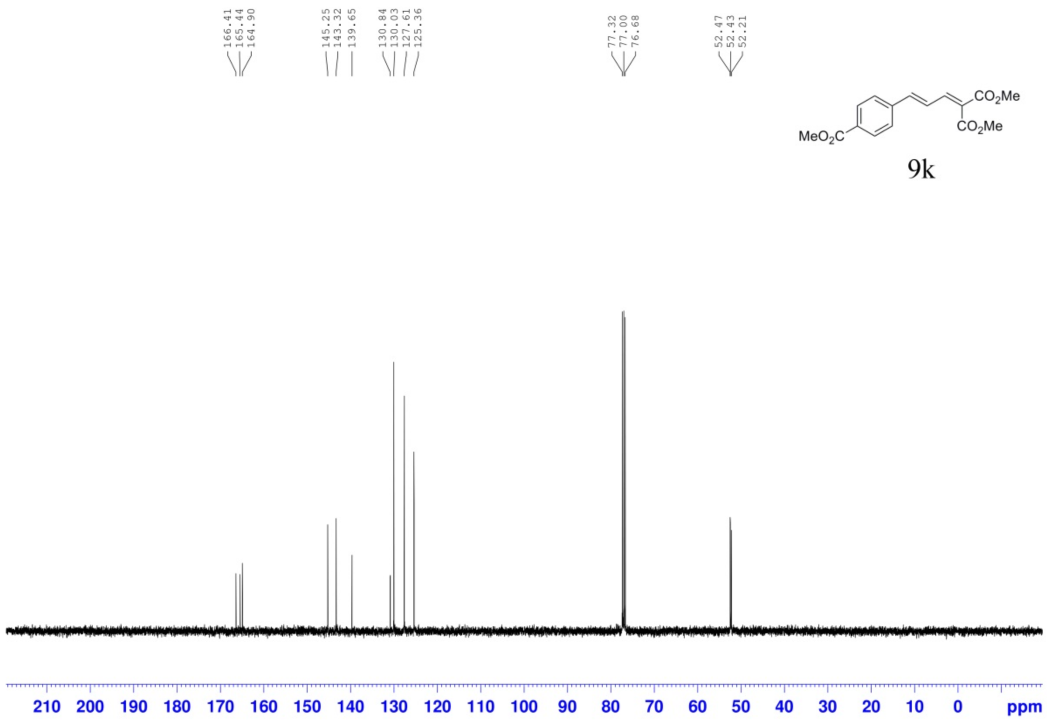

Supplementary Figure 101. ^{13}C NMR spectrum for dimethyl (*E*)-2-(3-(4-(methoxycarbonyl)phenyl)allylidene)malonate **(9k)**.

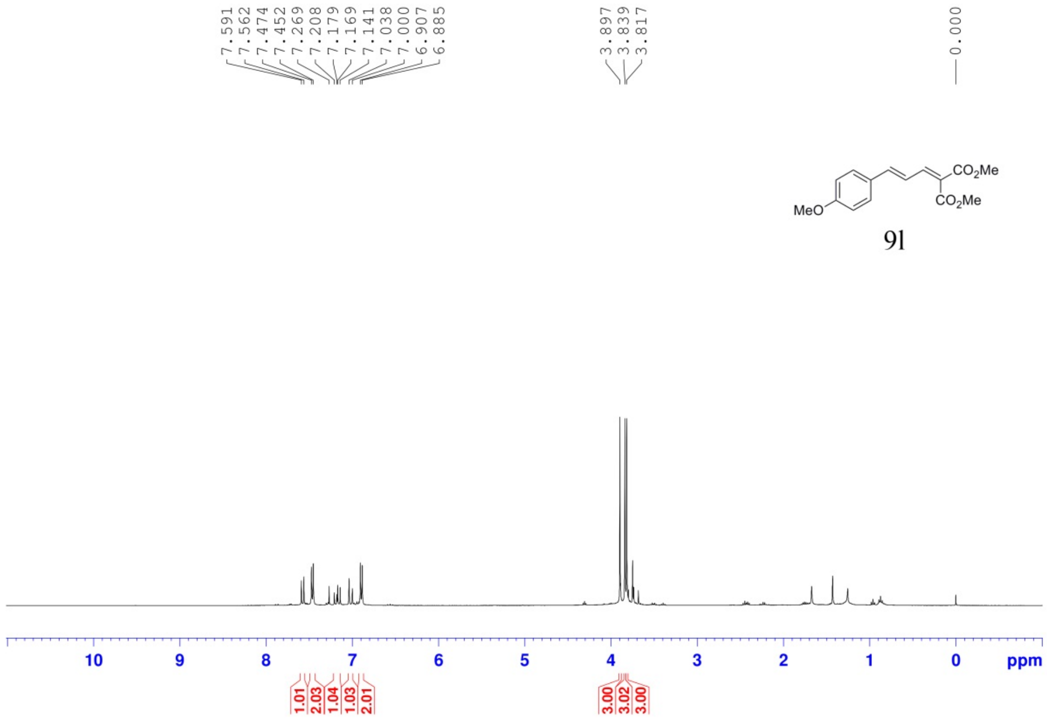

Supplementary Figure 102. ^{1}H NMR spectrum for dimethyl (*E*)-2-(3-(4-methoxyphenyl)allylidene)malonate **(9l)**.

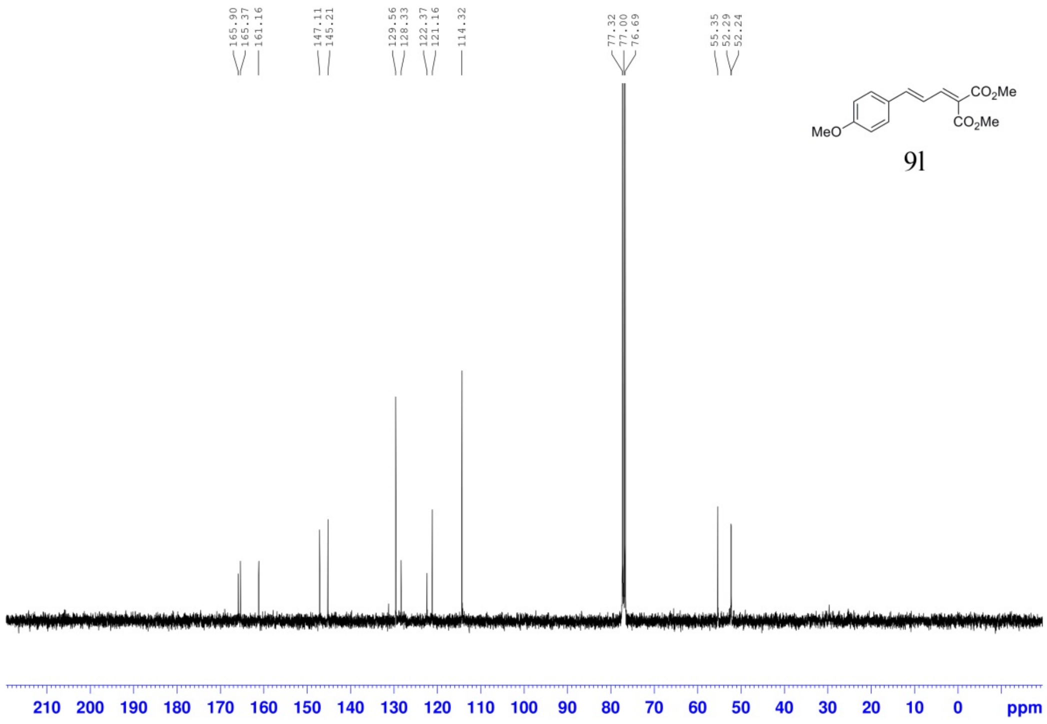

Supplementary Figure 103. ^{13}C NMR spectrum for dimethyl (*E*)-2-(3-(4-methoxyphenyl)allylidene)malonate **(9l)**.

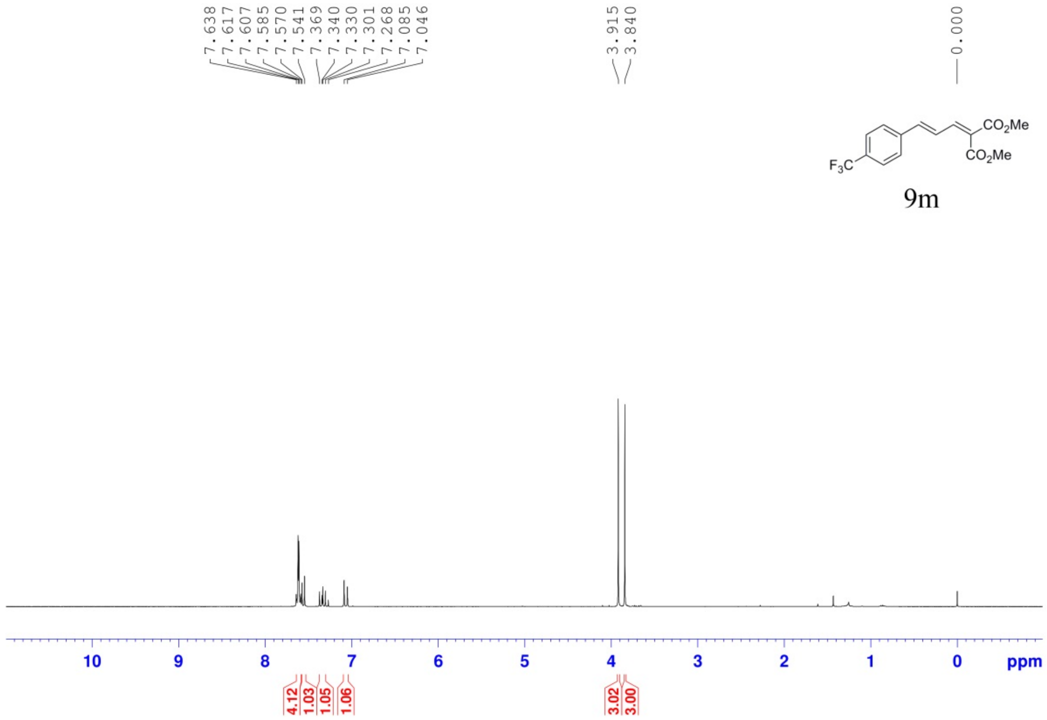

Supplementary Figure 104. ^{1}H NMR spectrum for dimethyl (*E*)-2-(3-(4-(trifluoromethyl)phenyl)allylidene)malonate **(9m)**.

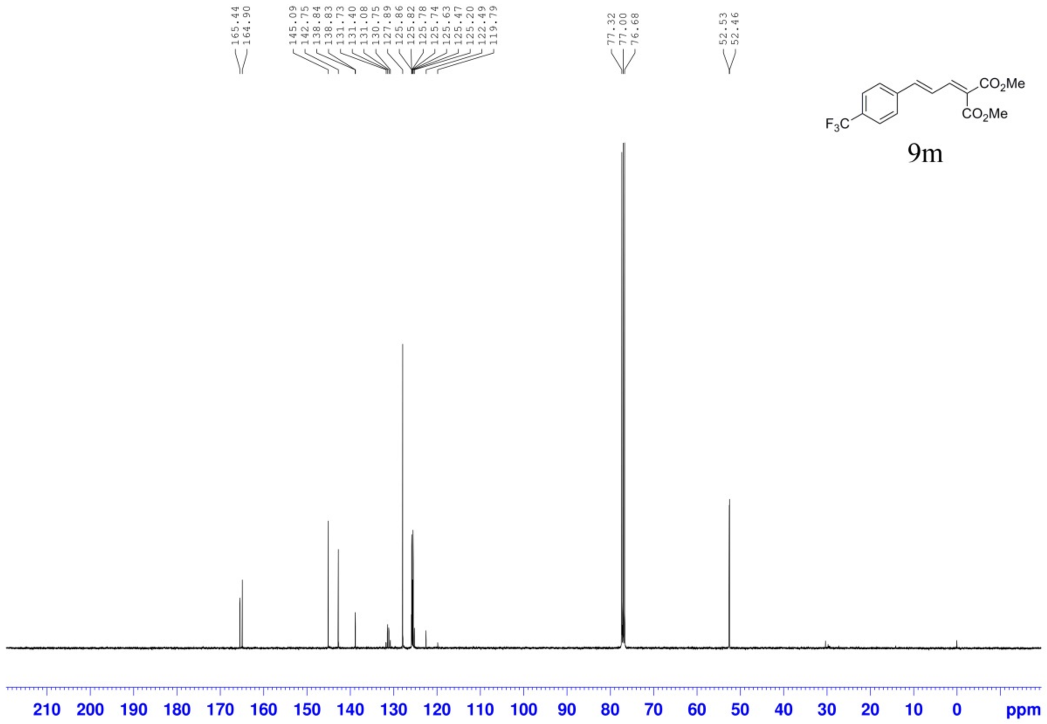

Supplementary Figure 105. ^{13}C NMR spectrum for dimethyl (*E*)-2-(3-(4-(trifluoromethyl)phenyl)allylidene)malonate **(9m)**.

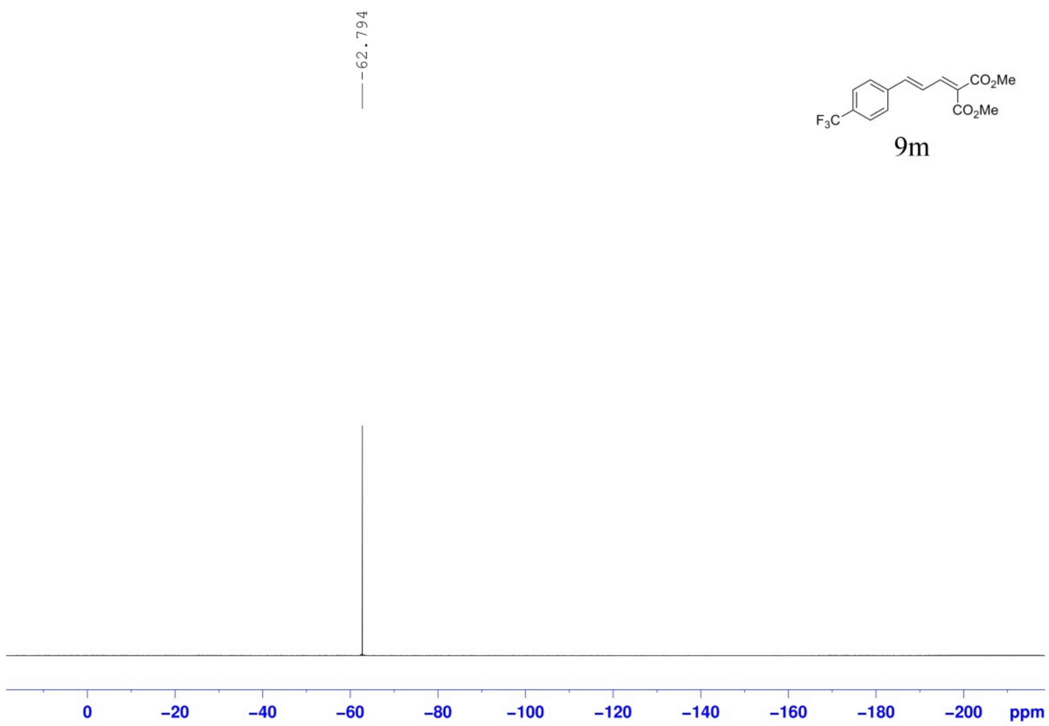

Supplementary Figure 106. ^{19}F NMR spectrum for dimethyl (*E*)-2-(3-(4-(trifluoromethyl)phenyl)allylidene)malonate **(9m)**.

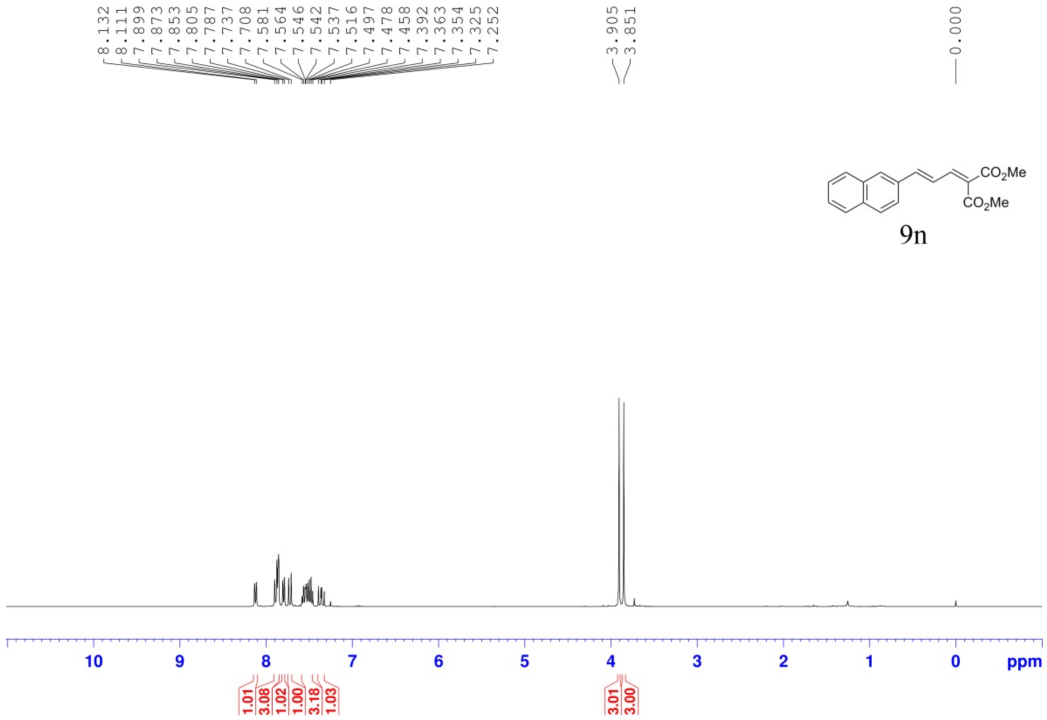

Supplementary Figure 107. ^1H NMR spectrum for dimethyl (*E*)-2-(3-(naphthalen-2-yl)allylidene)malonate **(9n)**.

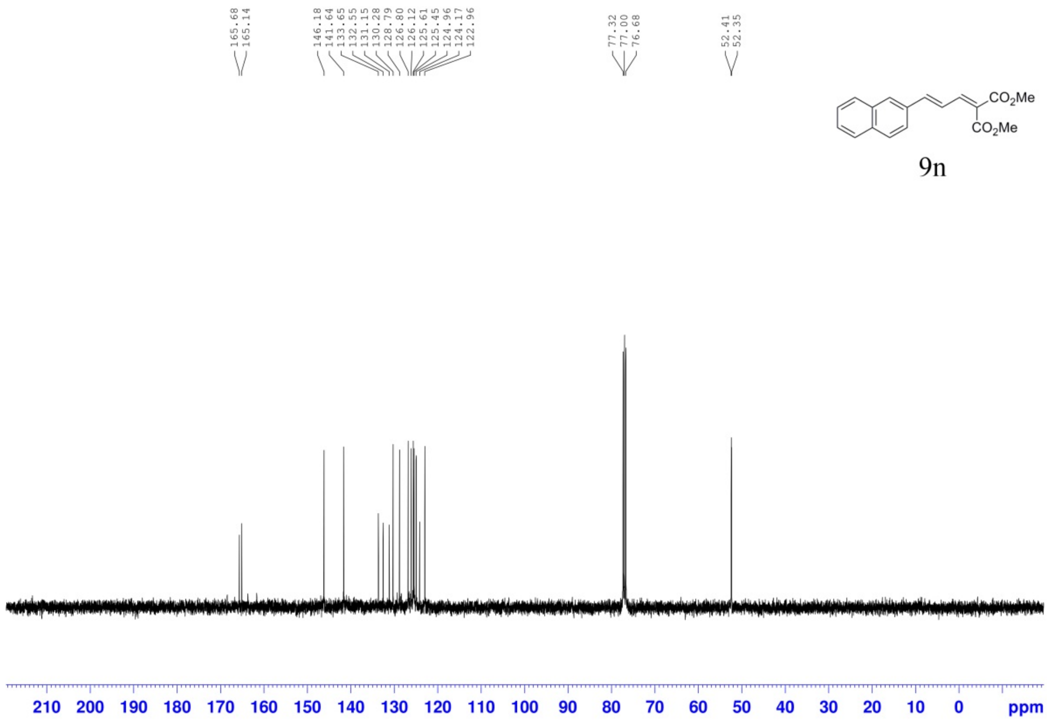

Supplementary Figure 108. ^{13}C NMR spectrum for dimethyl (*E*)-2-(3-(naphthalen-2-yl)allylidene)malonate **(9n)**.

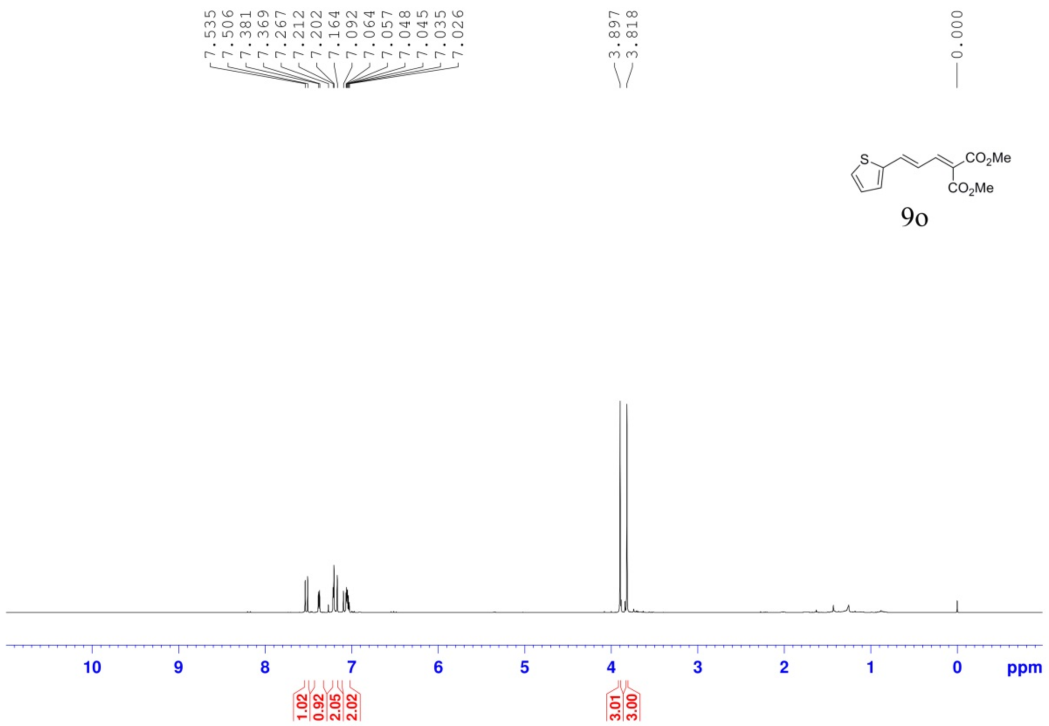

Supplementary Figure 109. ^{1}H NMR spectrum for dimethyl (*E*)-2-(3-(thiophen-2-yl)allylidene)malonate **(9o)**.

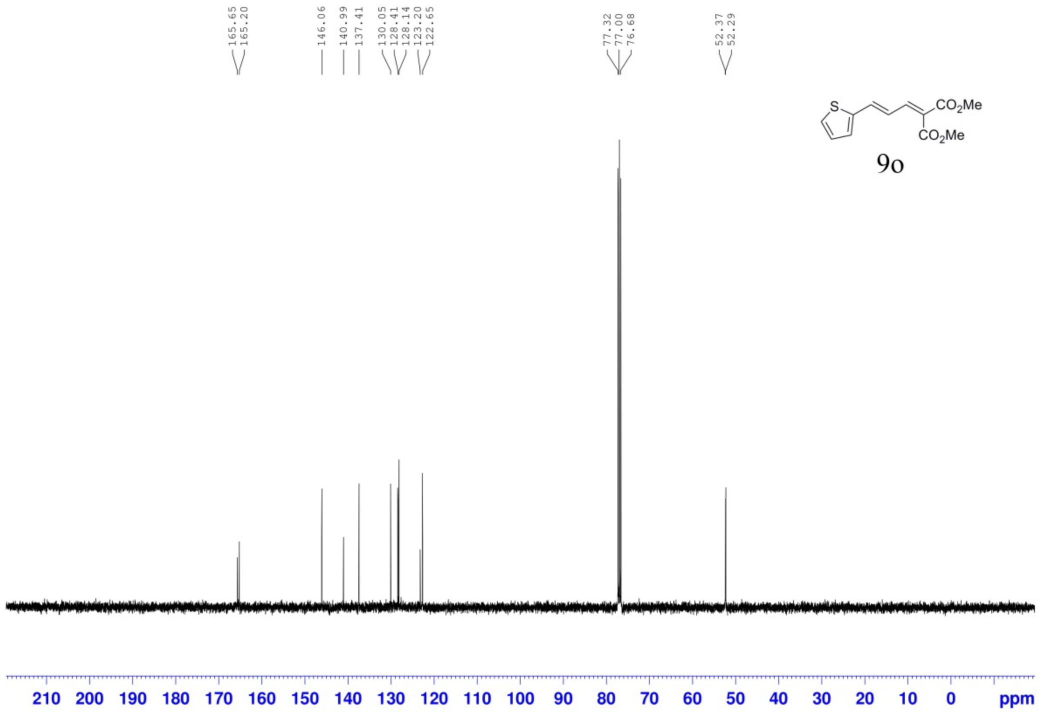

Supplementary Figure 110. ^{13}C NMR spectrum for dimethyl (*E*)-2-(3-(thiophen-2-yl)allylidene)malonate **(9o)**.

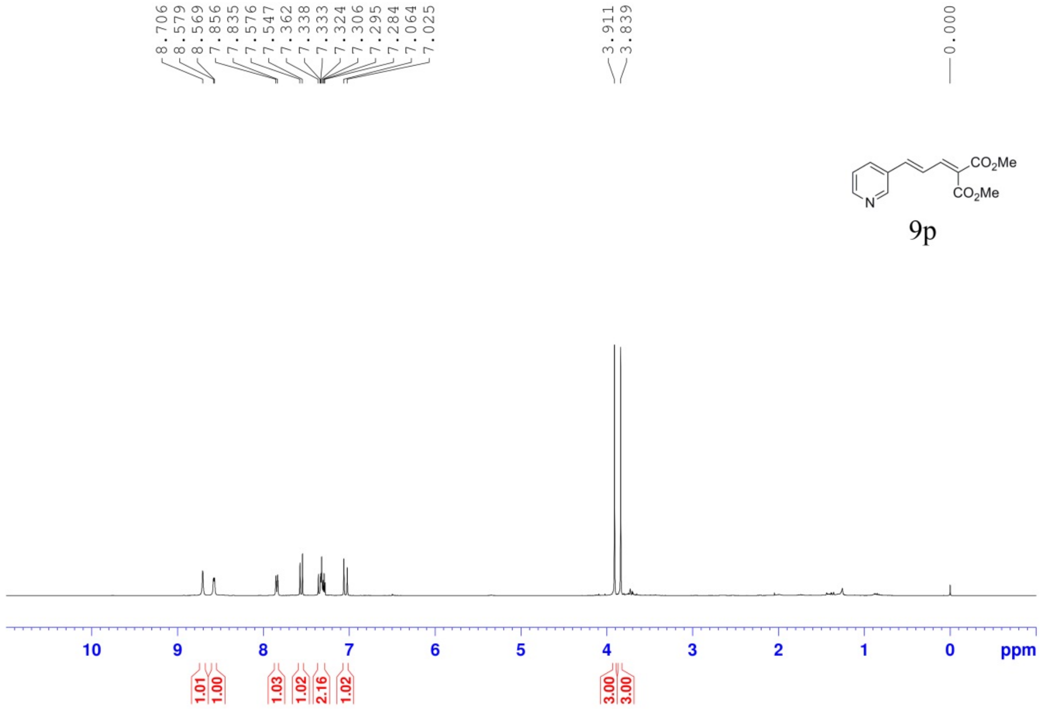

Supplementary Figure 111. ^{1}H NMR spectrum for dimethyl (*E*)-2-(3-(pyridin-3-yl)allylidene)malonate **(9p)**.

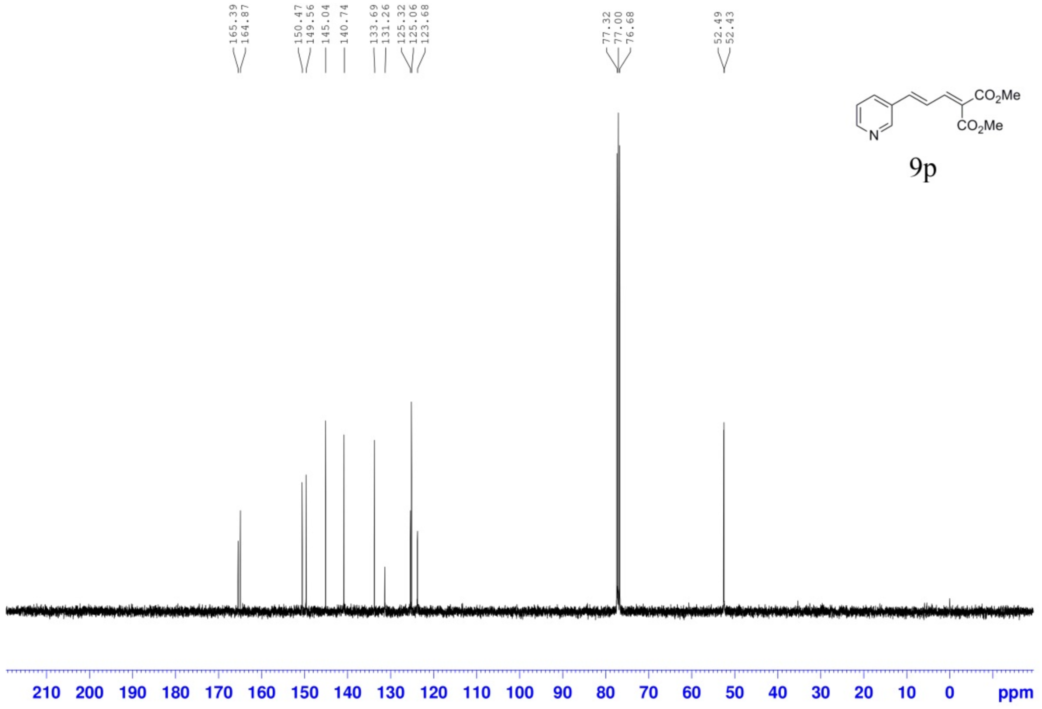

Supplementary Figure 112. ^{13}C NMR spectrum for dimethyl (*E*)-2-(3-(pyridin-3-yl)allylidene)malonate **(9p)**.

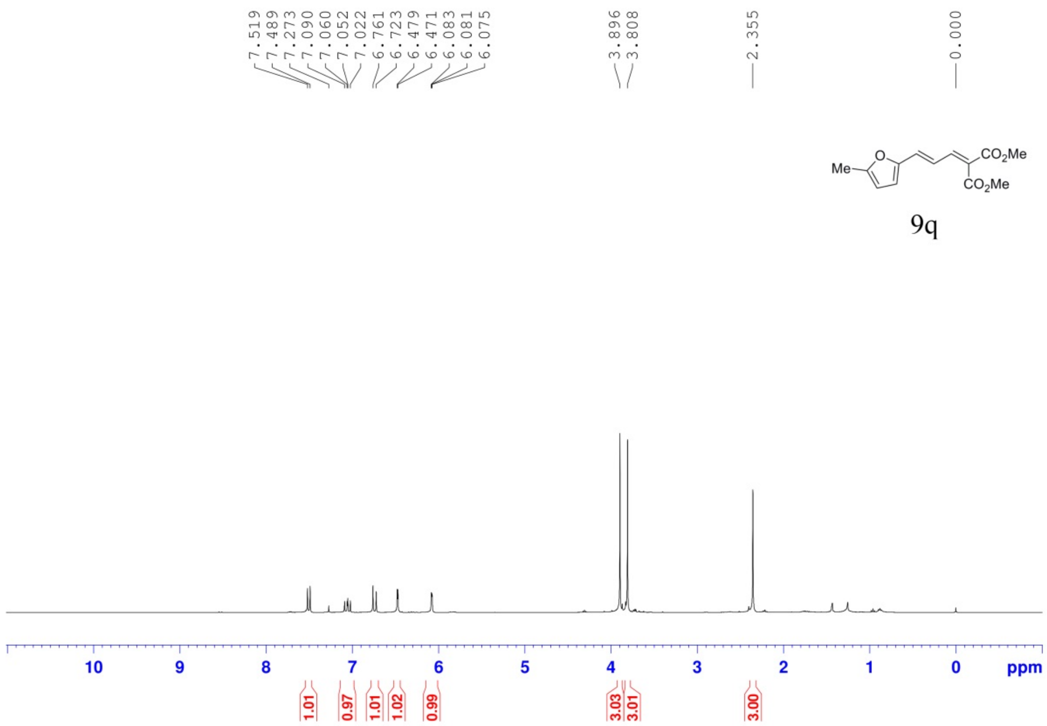

Supplementary Figure 113. ^{1}H NMR spectrum for dimethyl (*E*)-2-(3-(5-methylfuran-2-yl)allylidene)malonate **(9q)**.

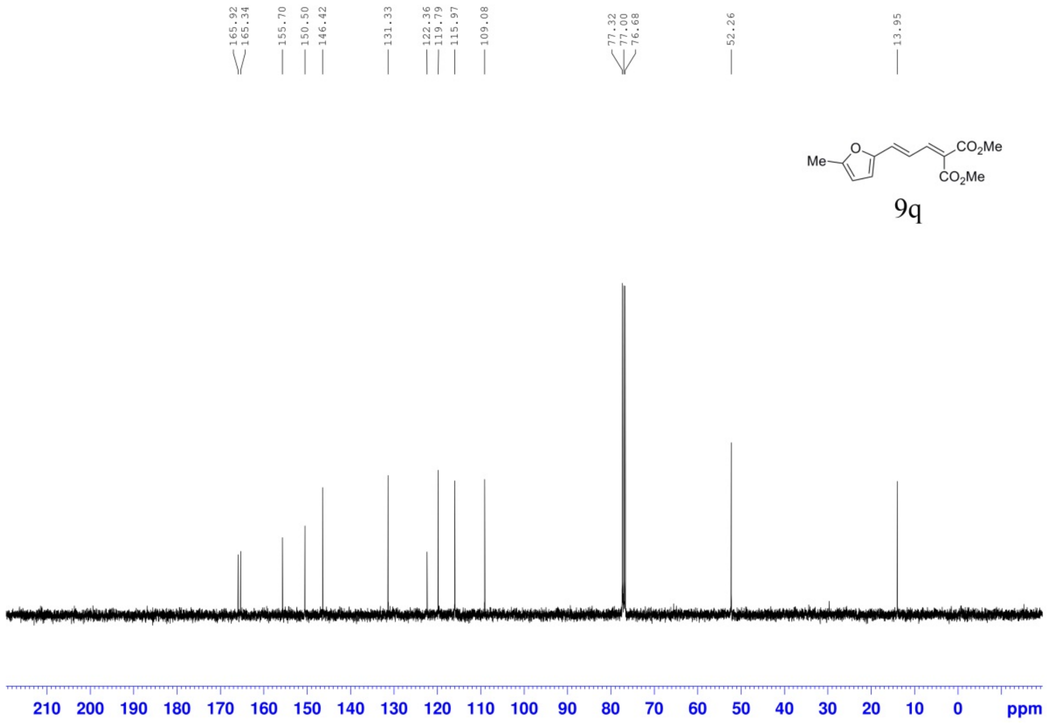

Supplementary Figure 114. ^{13}C NMR spectrum for dimethyl (*E*)-2-(3-(5-methylfuran-2-yl)allylidene)malonate **(9q)**.

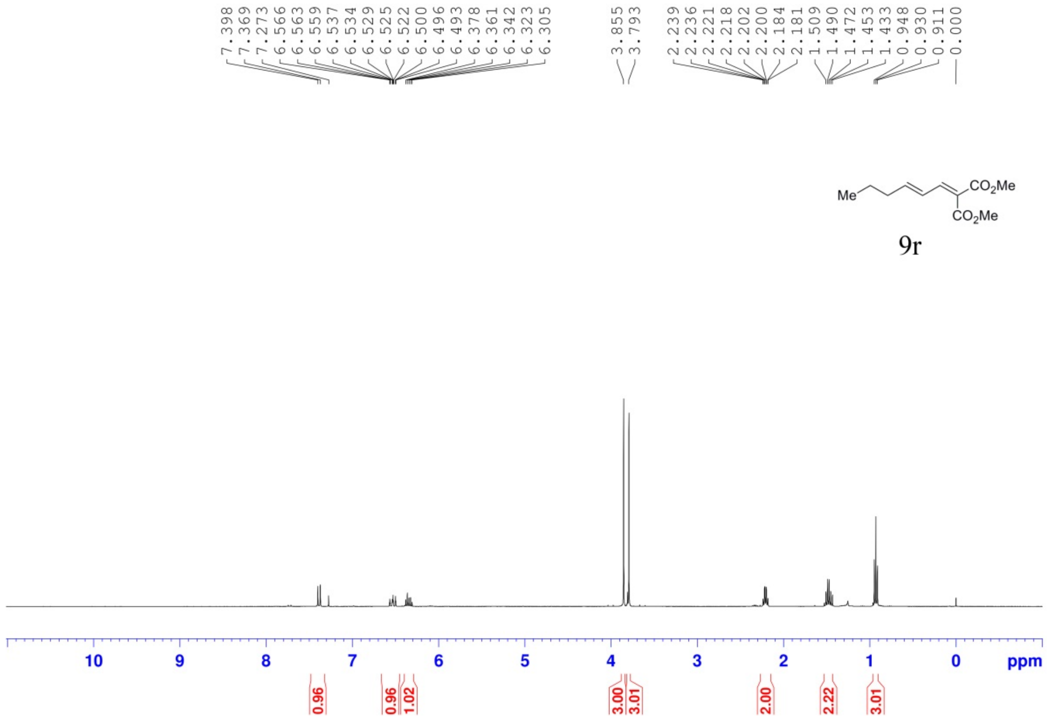

Supplementary Figure 115. ^{1}H NMR spectrum for dimethyl (*E*)-2-(hex-2-en-1-ylidene)malonate **(9r)**.

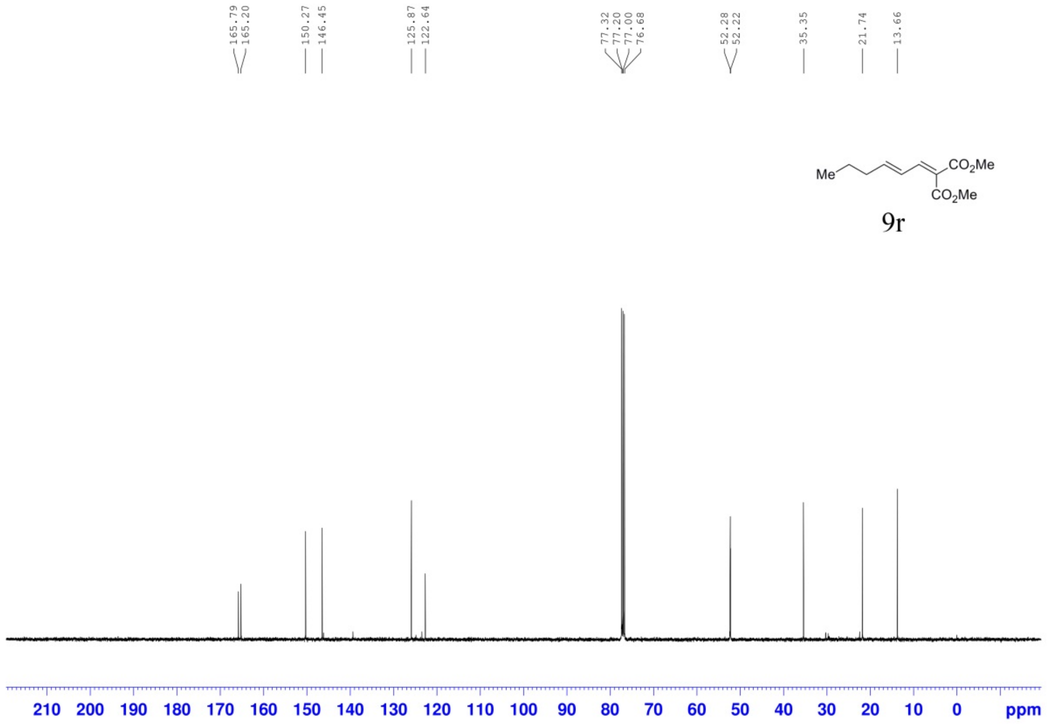

Supplementary Figure 116. ^{13}C NMR spectrum for dimethyl (*E*)-2-(hex-2-en-1-ylidene)malonate **(9r)**.

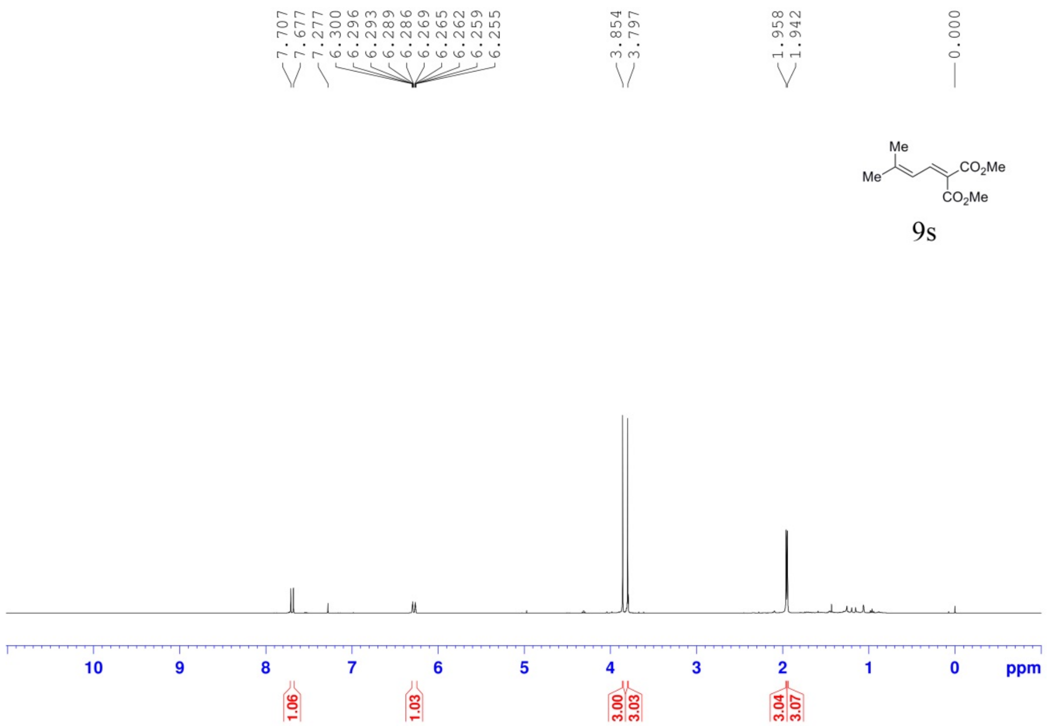

Supplementary Figure 117. ^{1}H NMR spectrum for dimethyl 2-(3-methylbut-2-en-1-ylidene)malonate **(9s)**.

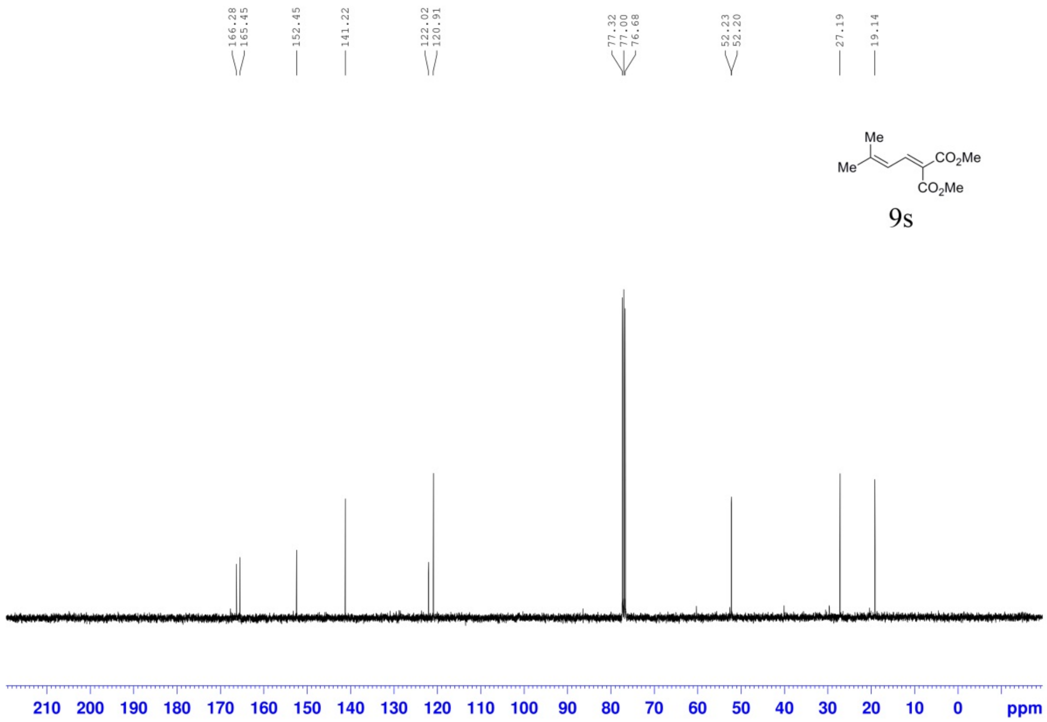

Supplementary Figure 118. ^{13}C NMR spectrum for dimethyl 2-(3-methylbut-2-en-1-ylidene)malonate **(9s)**.

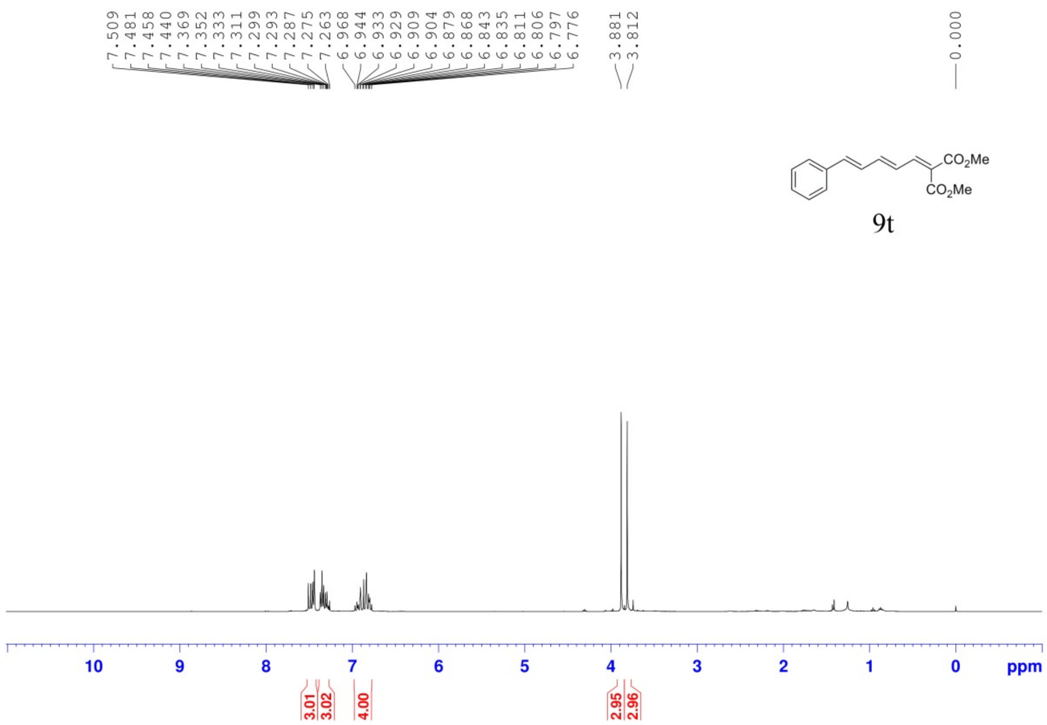

Supplementary Figure 119. ^{1}H NMR spectrum for dimethyl 2-((2E,4E)-5-phenylpenta-2,4-dien-1-ylidene)malonate **(9t)**.

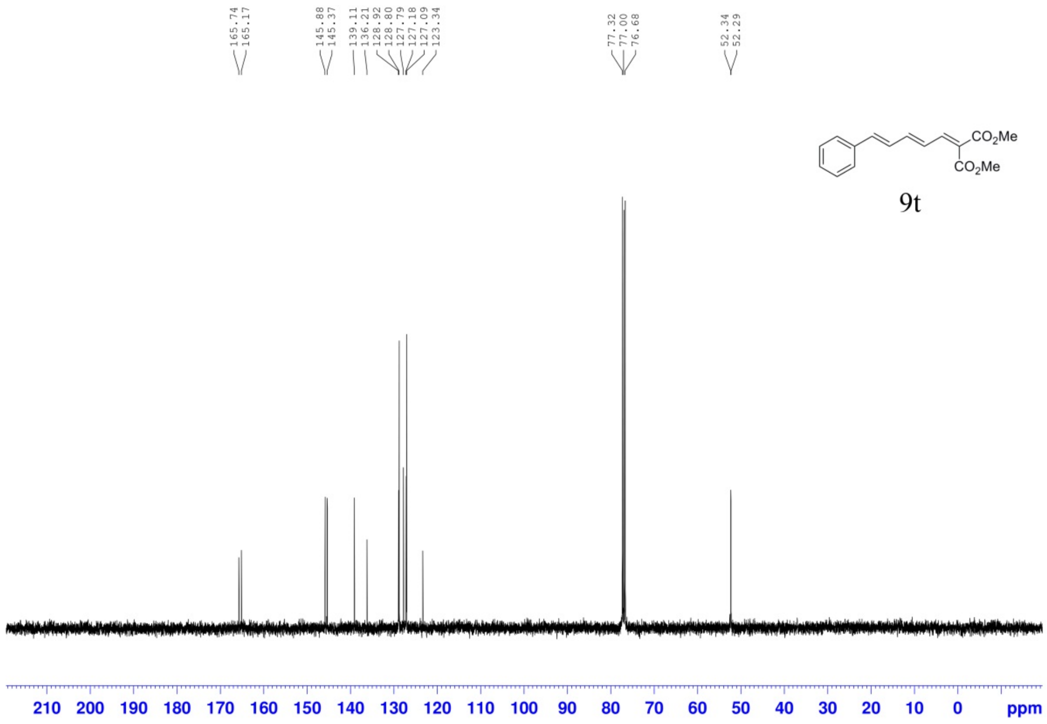

Supplementary Figure 120. ^{13}C NMR spectrum for dimethyl 2-((2E,4E)-5-phenylpenta-2,4-dien-1-ylidene)malonate **(9t)**.

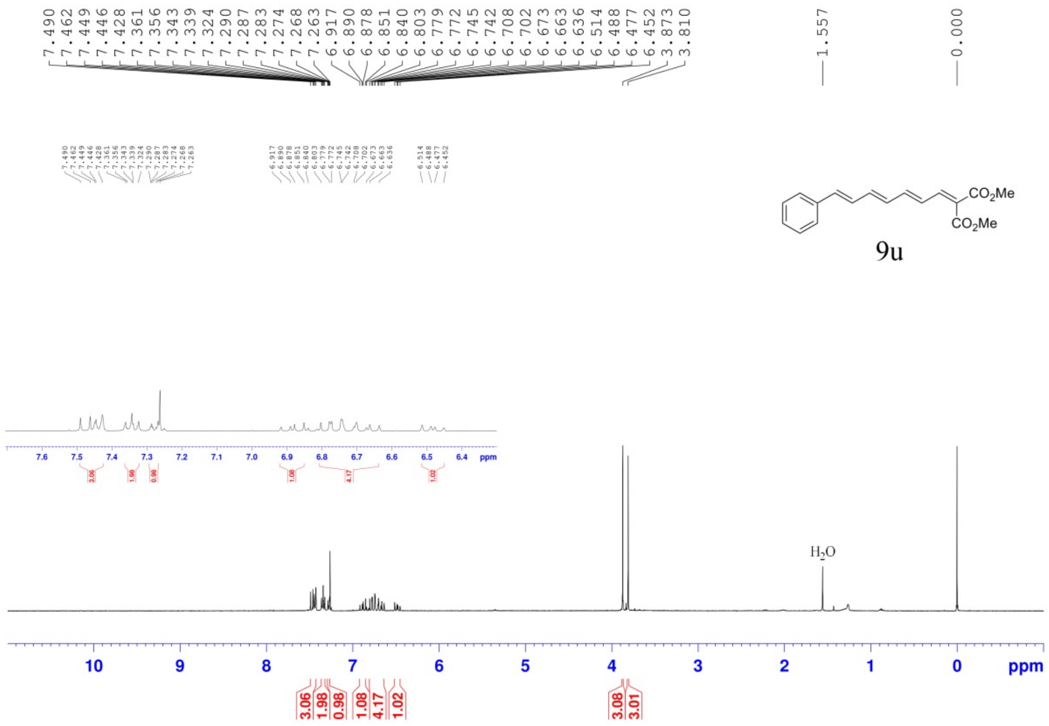

Supplementary Figure 121. ^{1}H NMR spectrum for dimethyl 2-((2*E*,4*E*,6*E*)-7-phenylhepta-2,4,6-trien-1-ylidene) malonate **(9u)**.

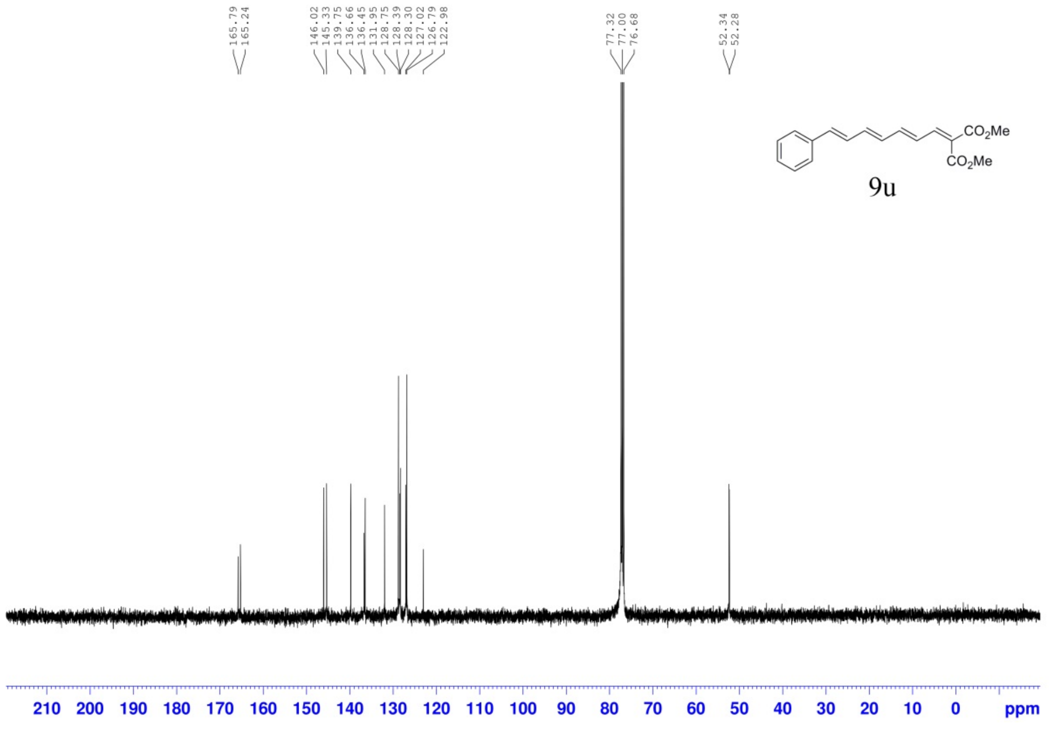

Supplementary Figure 122. ^{13}C NMR spectrum for dimethyl 2-((2*E*,4*E*,6*E*)-7-phenylhepta-2,4,6-trien-1-ylidene) malonate **(9u)**.

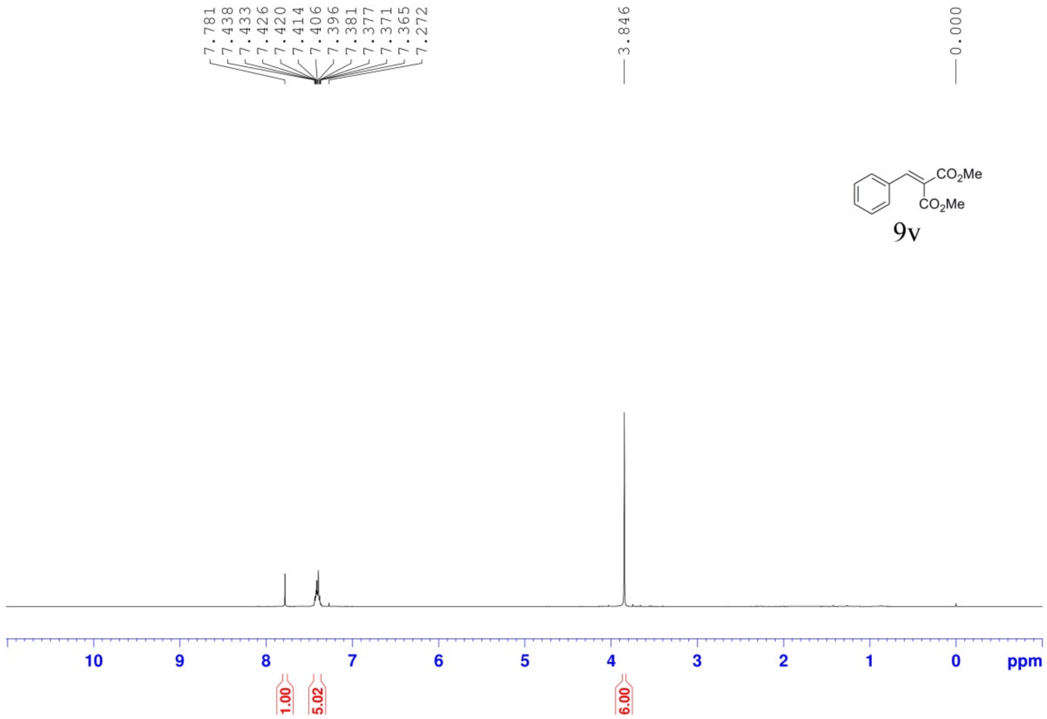

Supplementary Figure 123. ^{1}H NMR spectrum for dimethyl 2-benzylidenemalonate **(9v)**.

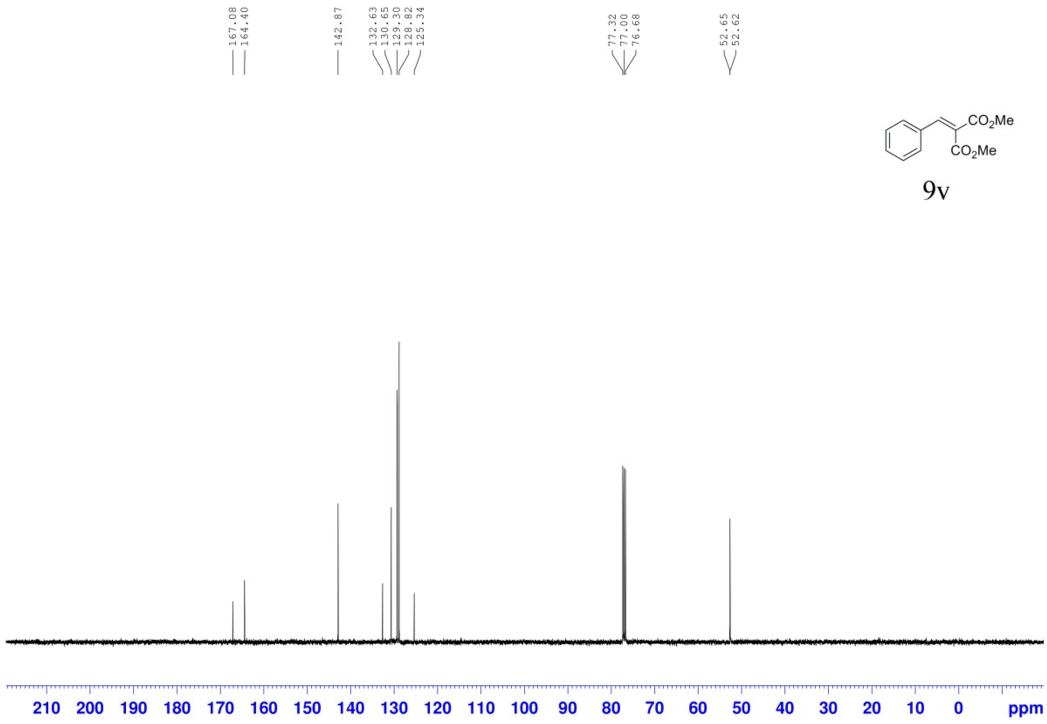

Supplementary Figure 124. ^{13}C NMR spectrum for dimethyl 2-benzylidenemalonate **(9v)**.

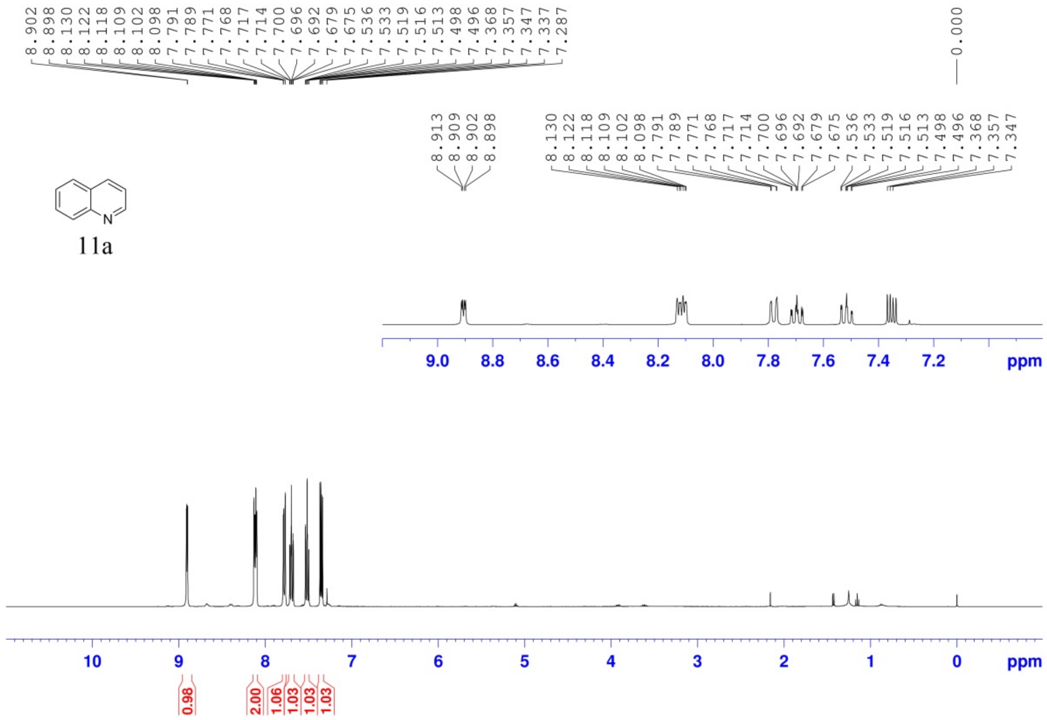

Supplementary Figure 125. ^{1}H NMR spectrum for quinolone (11a).

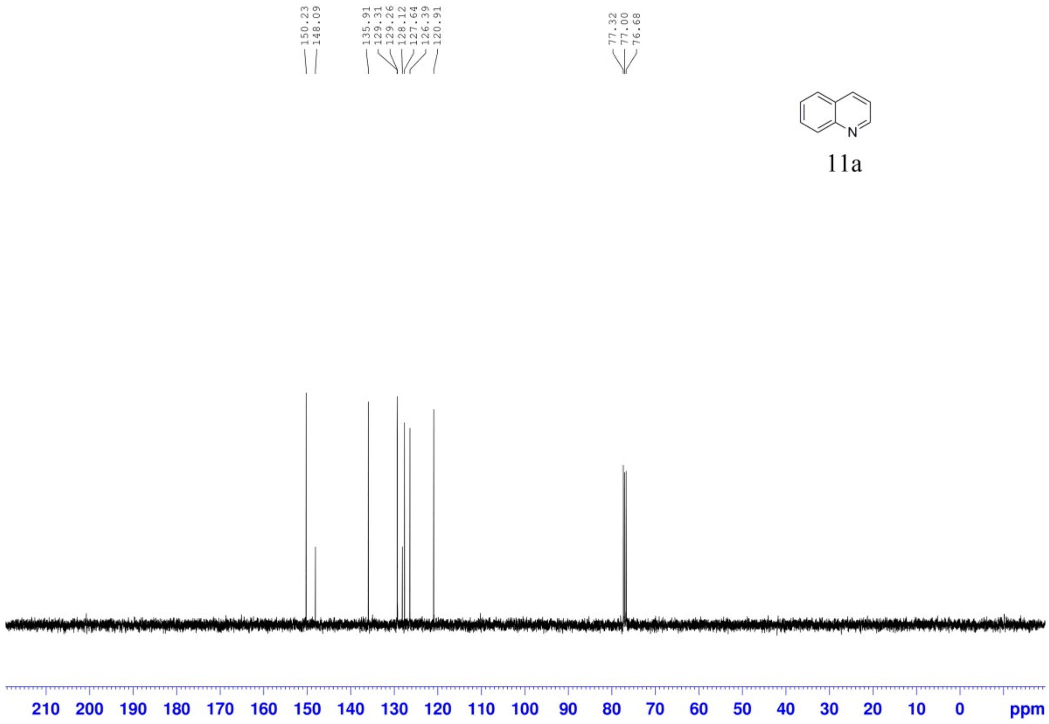

Supplementary Figure 126. ^{13}C NMR spectrum for quinolone (11a).

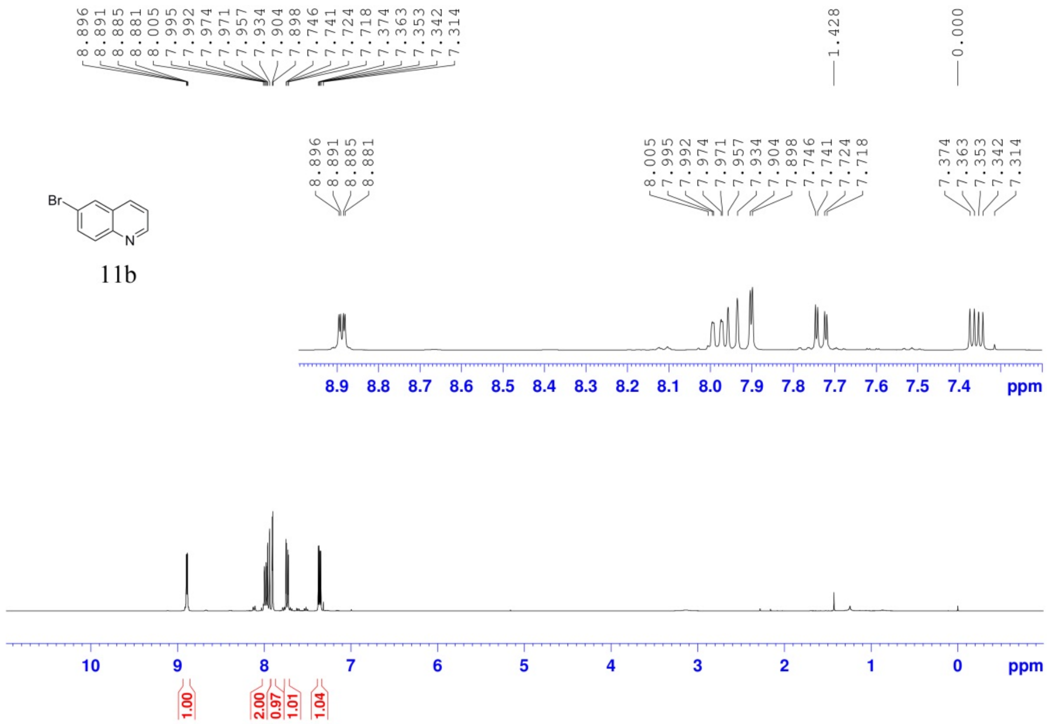

Supplementary Figure 127. ^{1}H NMR spectrum for 6-bromoquinoline **(11b)**.

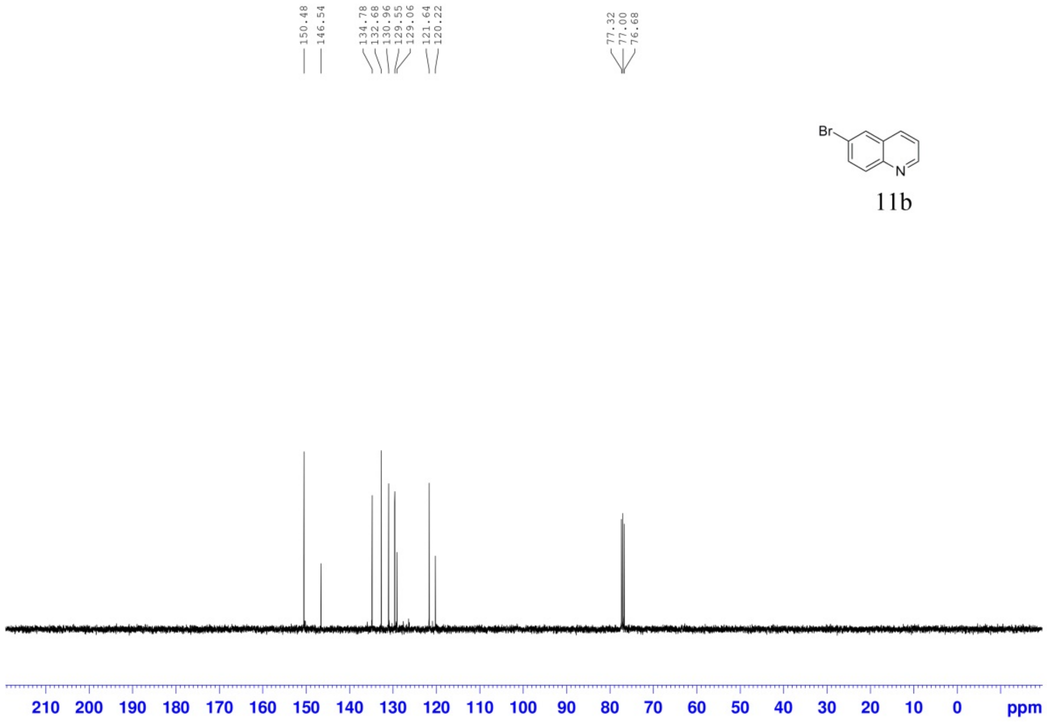

Supplementary Figure 128. ^{13}C NMR spectrum for 6-bromoquinoline **(11b)**.

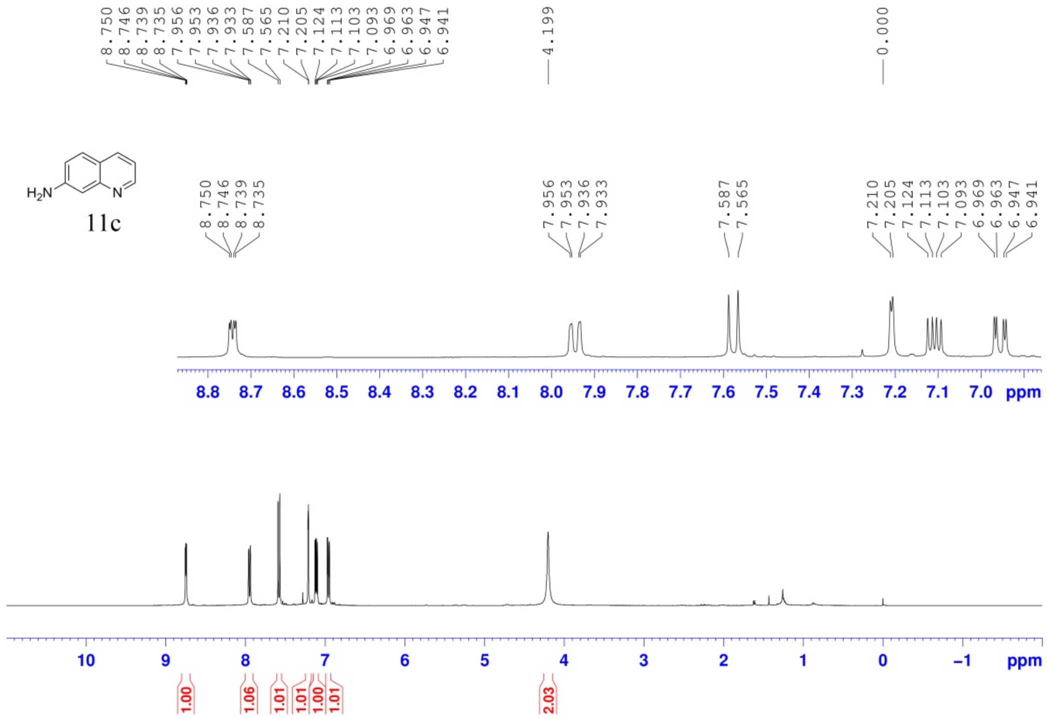

Supplementary Figure 129. ^{1}H NMR spectrum for quinolin-7-amine **(11c)**.

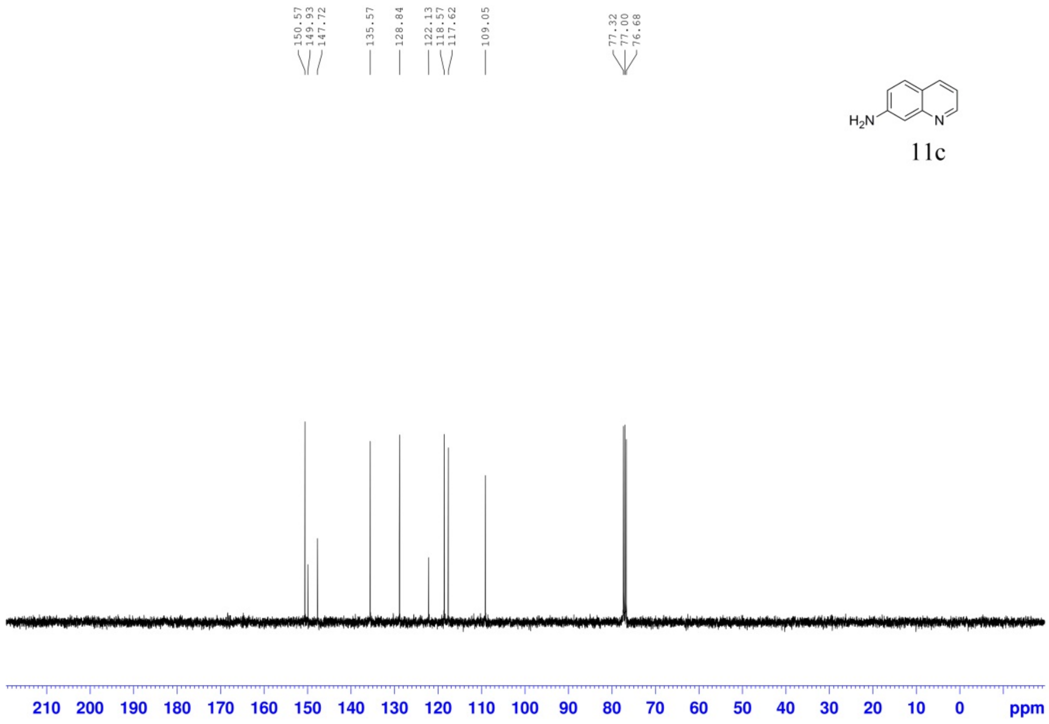

Supplementary Figure 130. ^{13}C NMR spectrum for quinolin-7-amine **(11c)**.

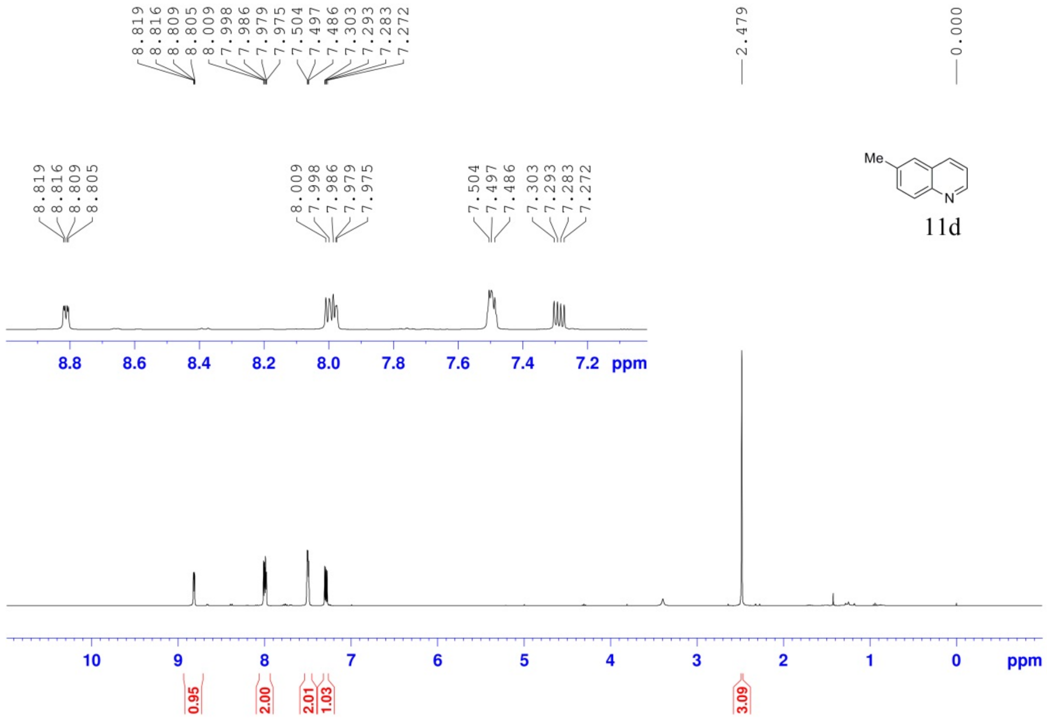

Supplementary Figure 131. ^{1}H NMR spectrum for 6-methylquinoline **(11d)**.

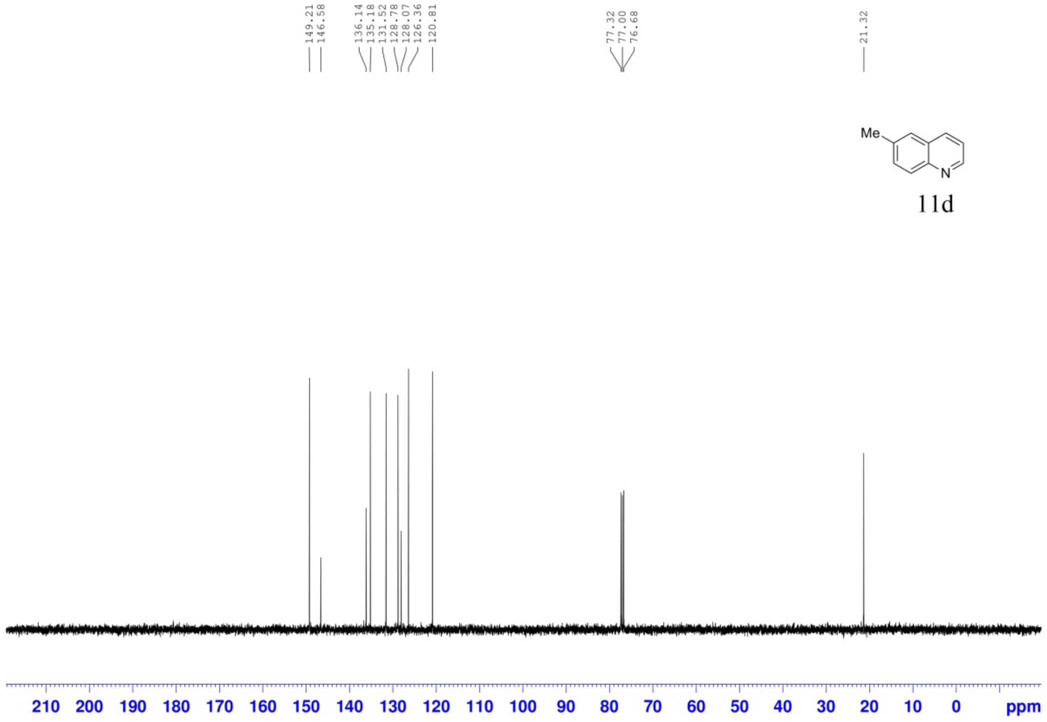

Supplementary Figure 132. ^{13}C NMR spectrum for 6-methylquinoline **(11d)**.

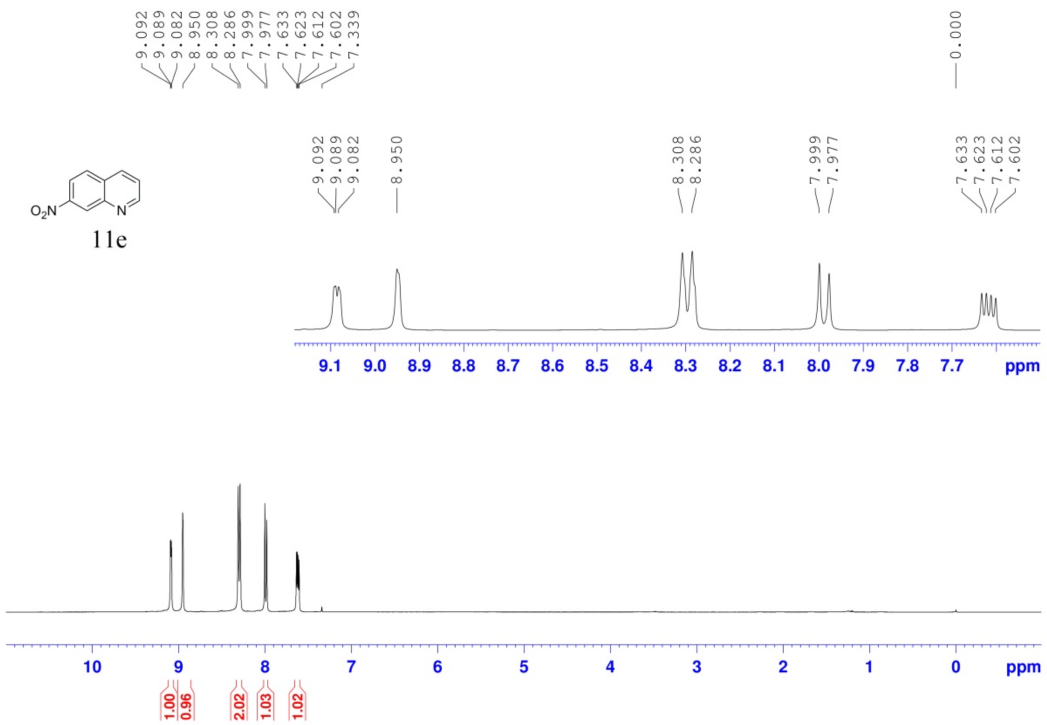

Supplementary Figure 133. ^{1}H NMR spectrum for 7-nitroquinoline **(11e)**.

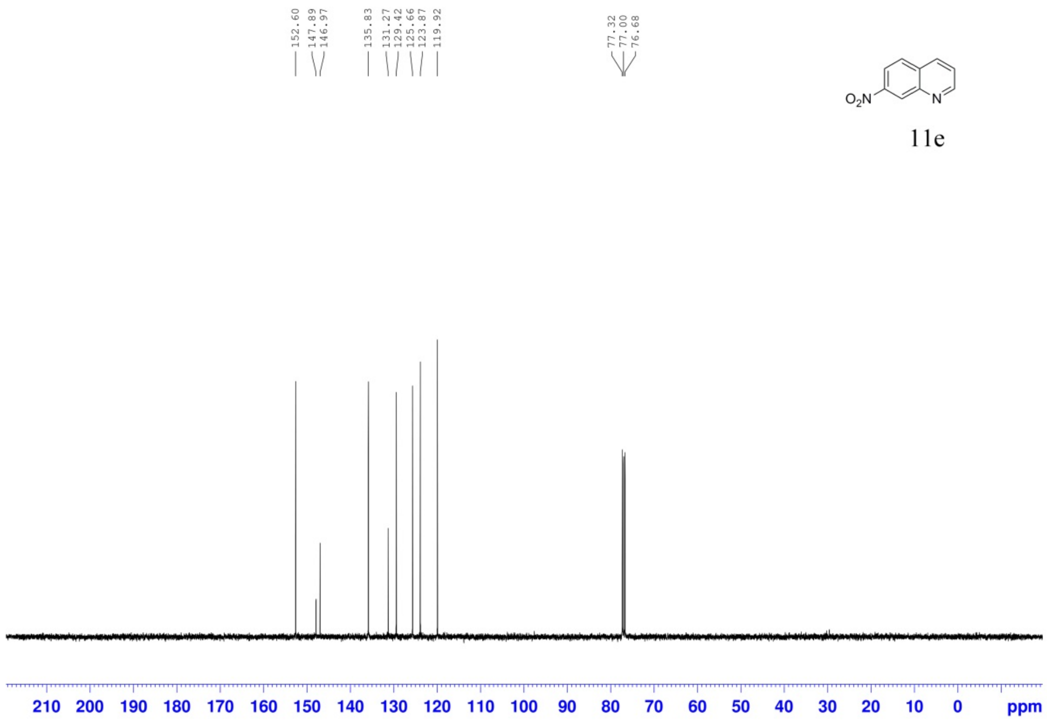

Supplementary Figure 134. ^{13}C NMR spectrum for 7-nitroquinoline **(11e)**.

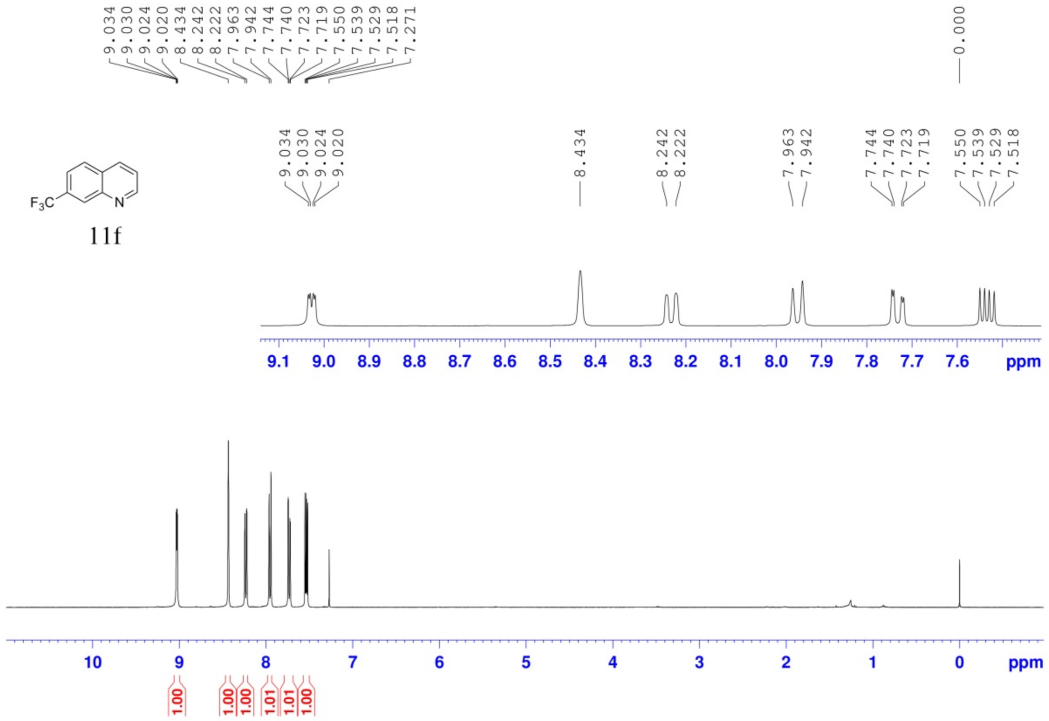

Supplementary Figure 135. ^{1}H NMR spectrum for 7-(trifluoromethyl)quinolone **(11f)**.

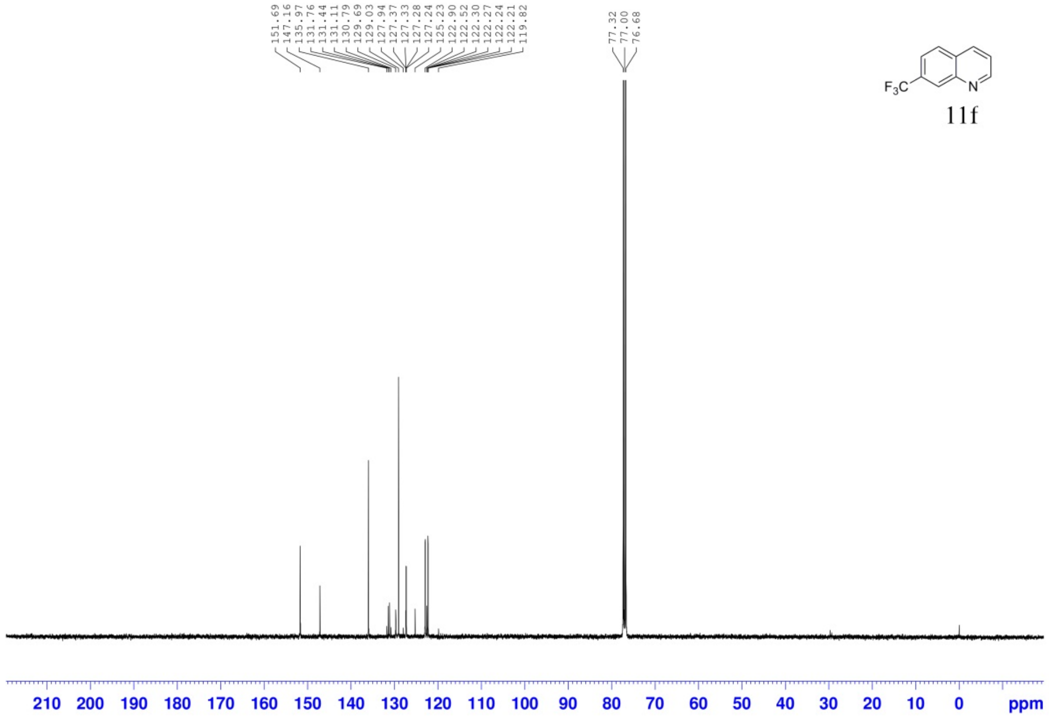

Supplementary Figure 136. ^{13}C NMR spectrum for 7-(trifluoromethyl)quinolone **(11f)**.

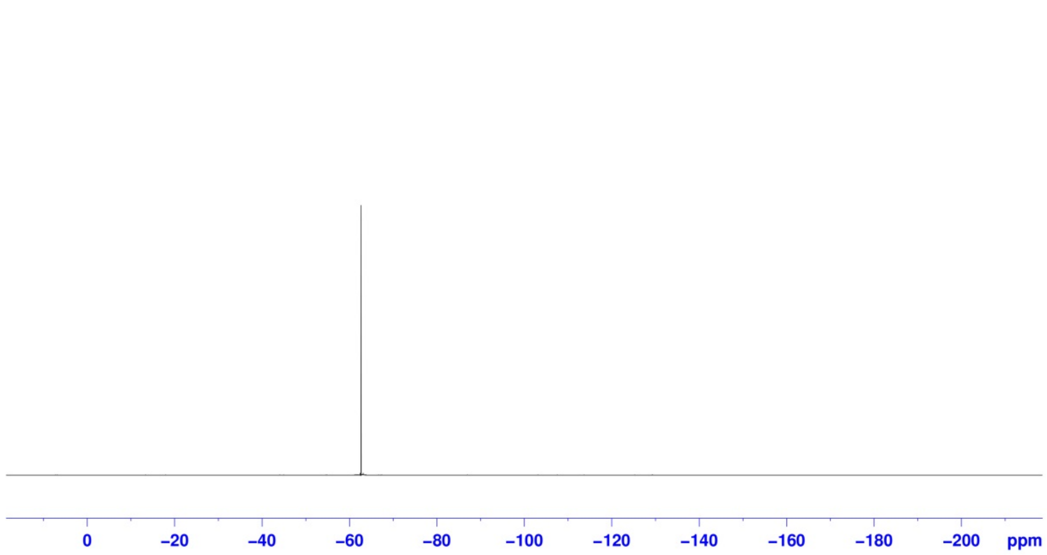

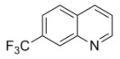

11f

Supplementary Figure 137. ^{19}F NMR spectrum for 7-(trifluoromethyl)quinolone **(11f)**.

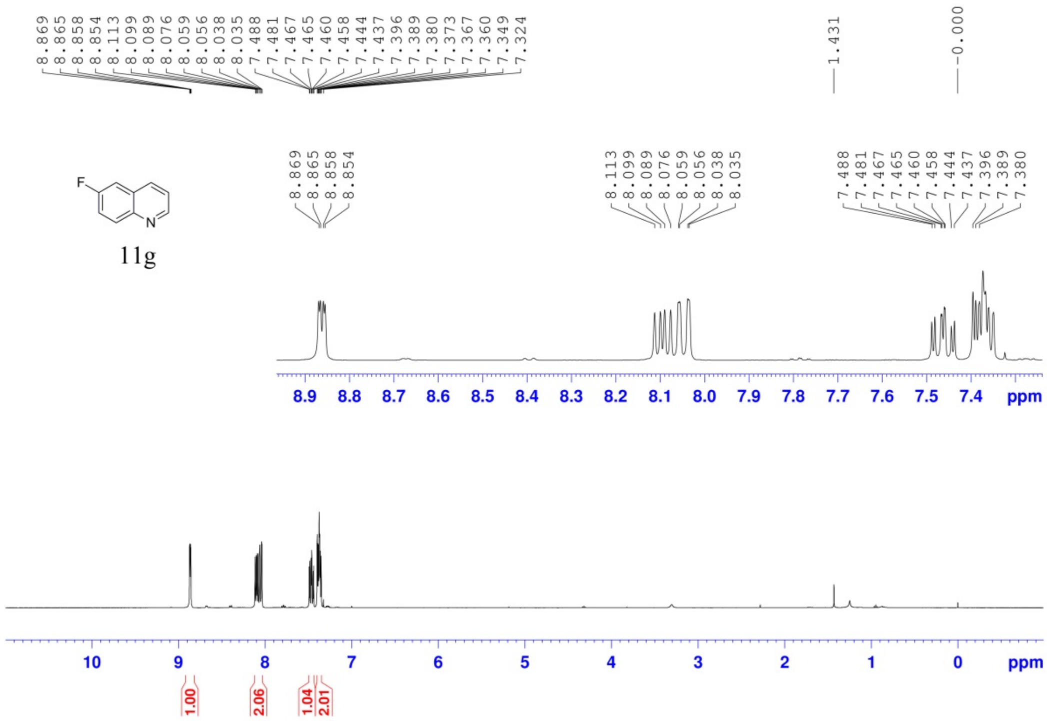

Supplementary Figure 138. ^{1}H NMR spectrum for 6-fluoroquinoline **(11g)**.

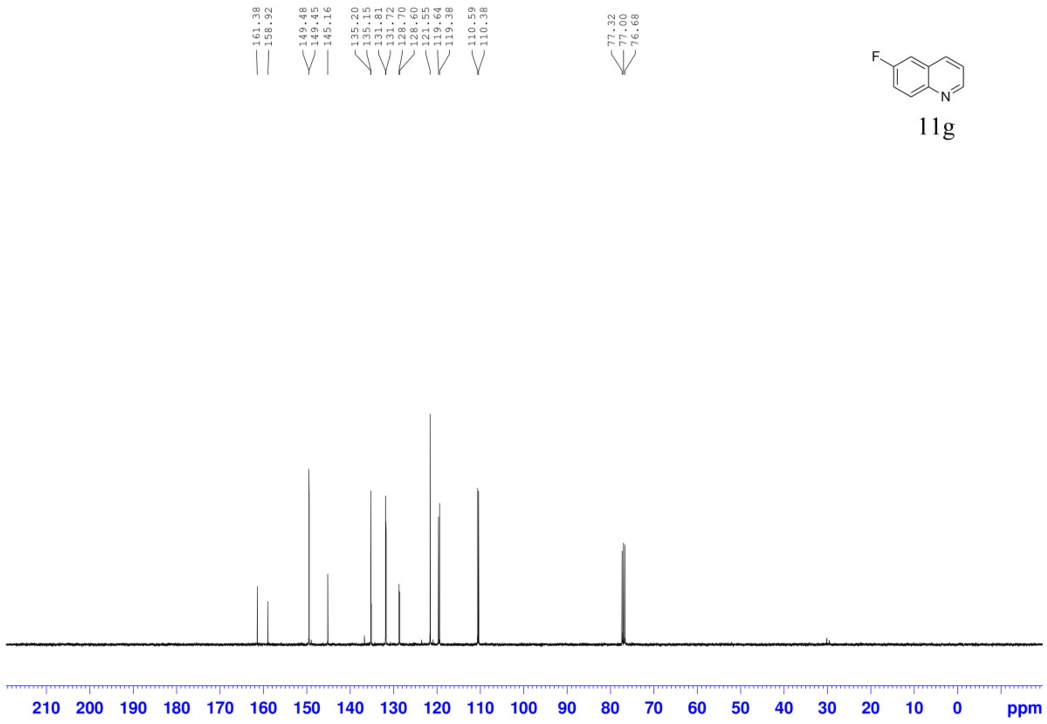

Supplementary Figure 139. ^{13}C NMR spectrum for 6-fluoroquinoline **(11g)**.

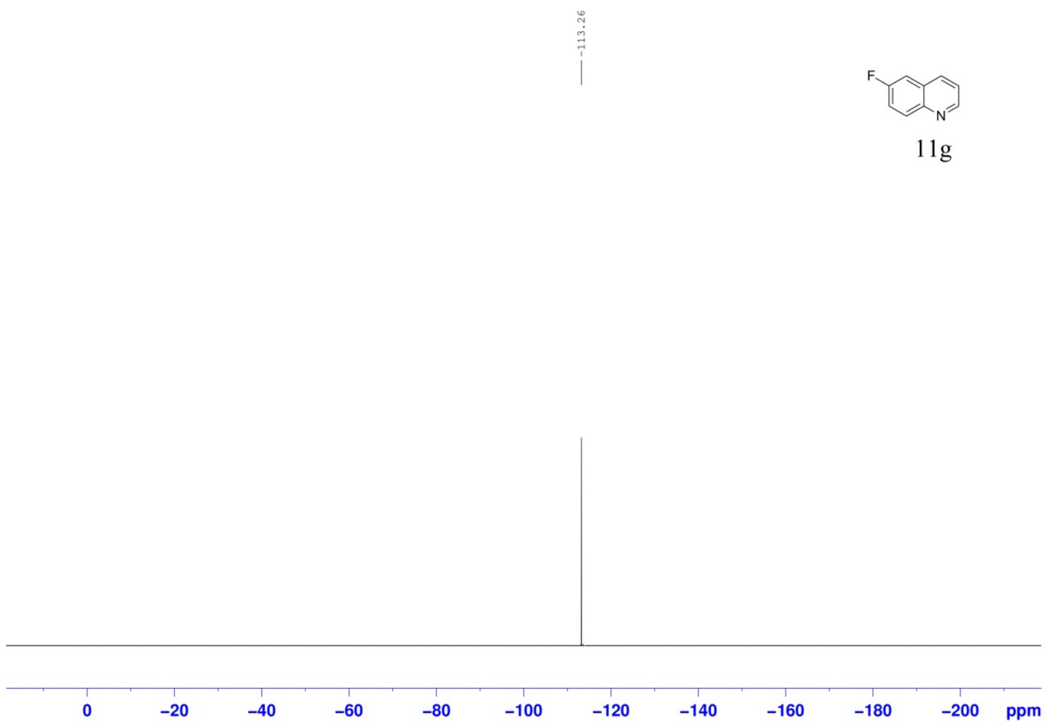

11g

Supplementary Figure 140. ^{19}F NMR spectrum for 6-fluoroquinoline **(11g)**.

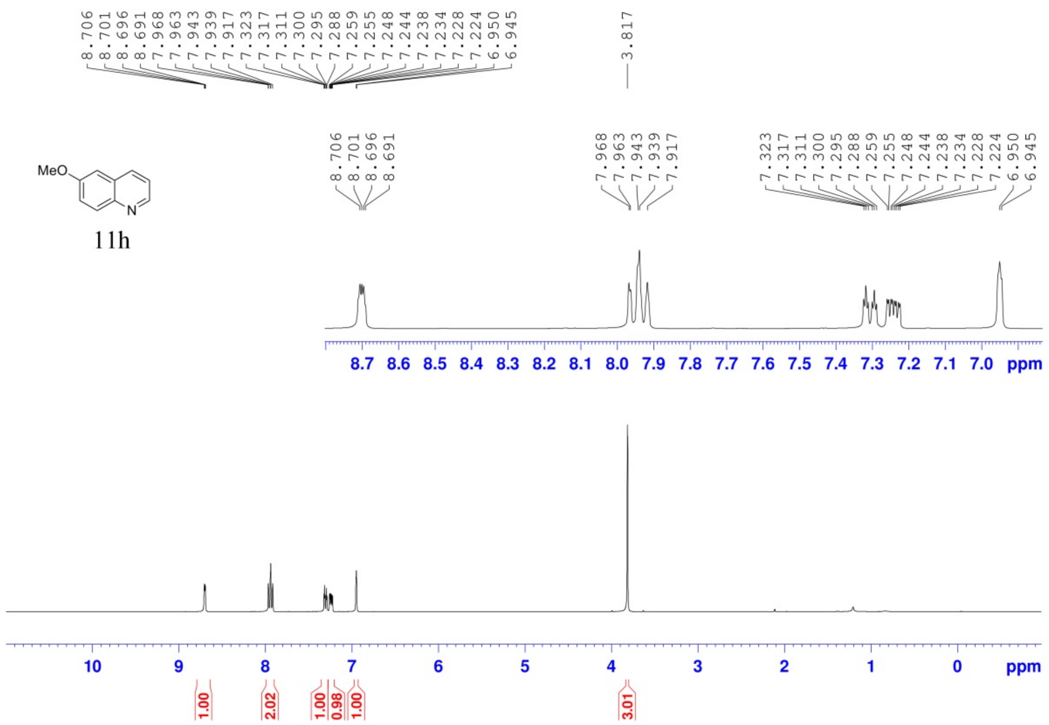

Supplementary Figure 141. ^{1}H NMR spectrum for 6-methoxyquinoline **(11h)**.

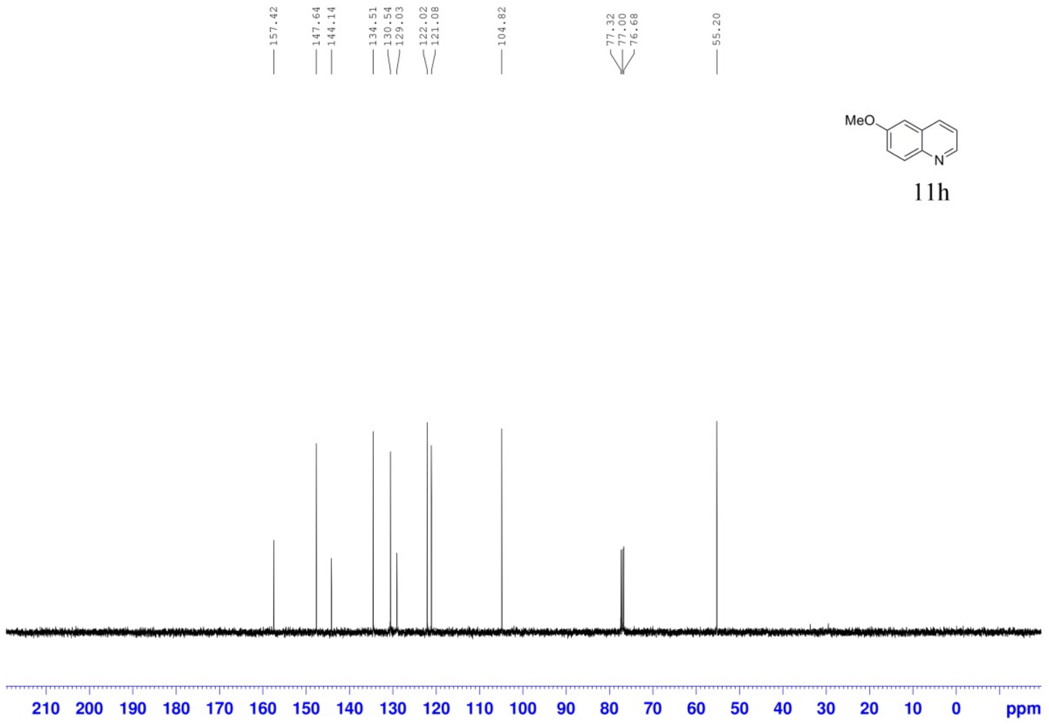

Supplementary Figure 142. ^{13}C NMR spectrum for 6-methoxyquinoline **(11h)**.

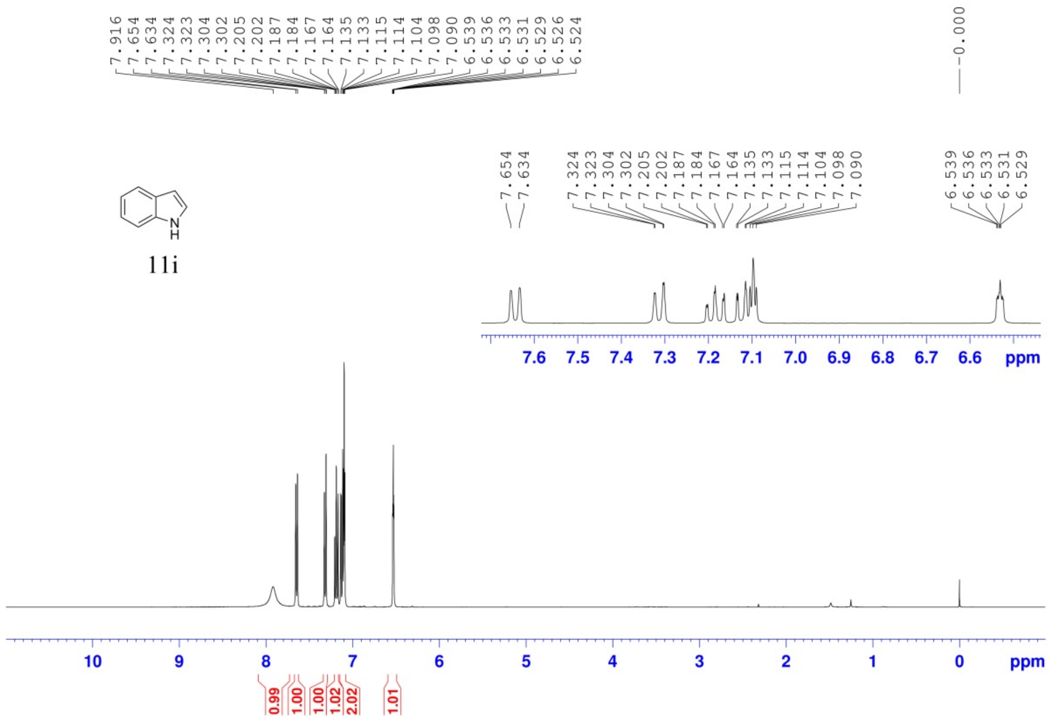

Supplementary Figure 143. ^{1}H NMR spectrum for 1H-indole **(11i)**.

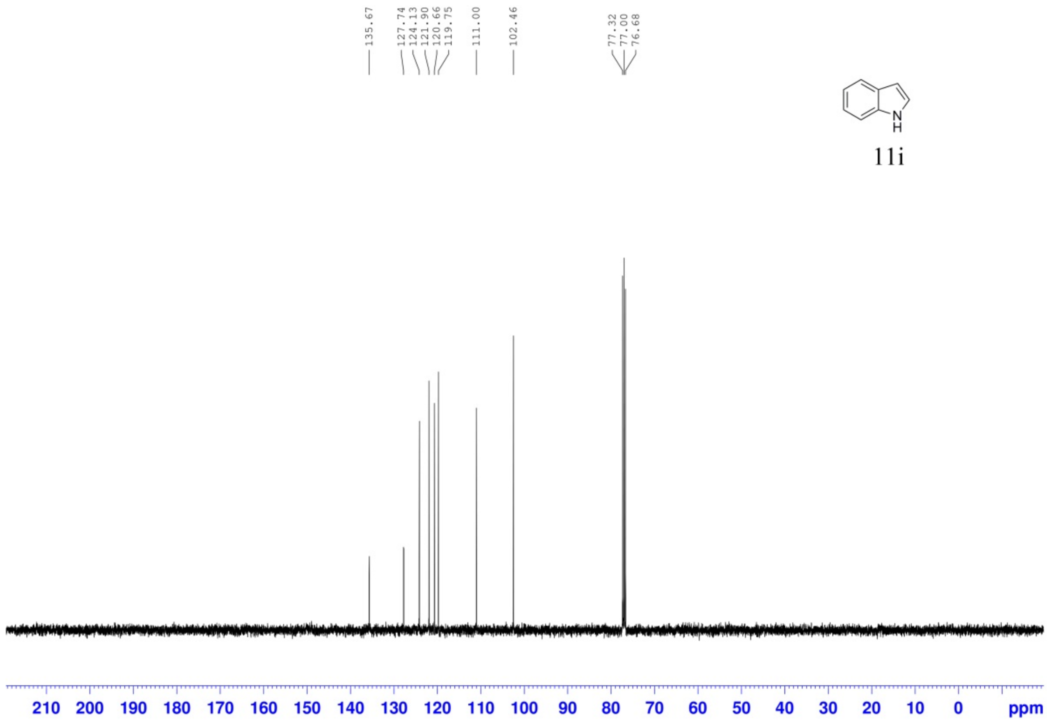

Supplementary Figure 144. ^{13}C NMR spectrum for 1H-indole **(11i)**.

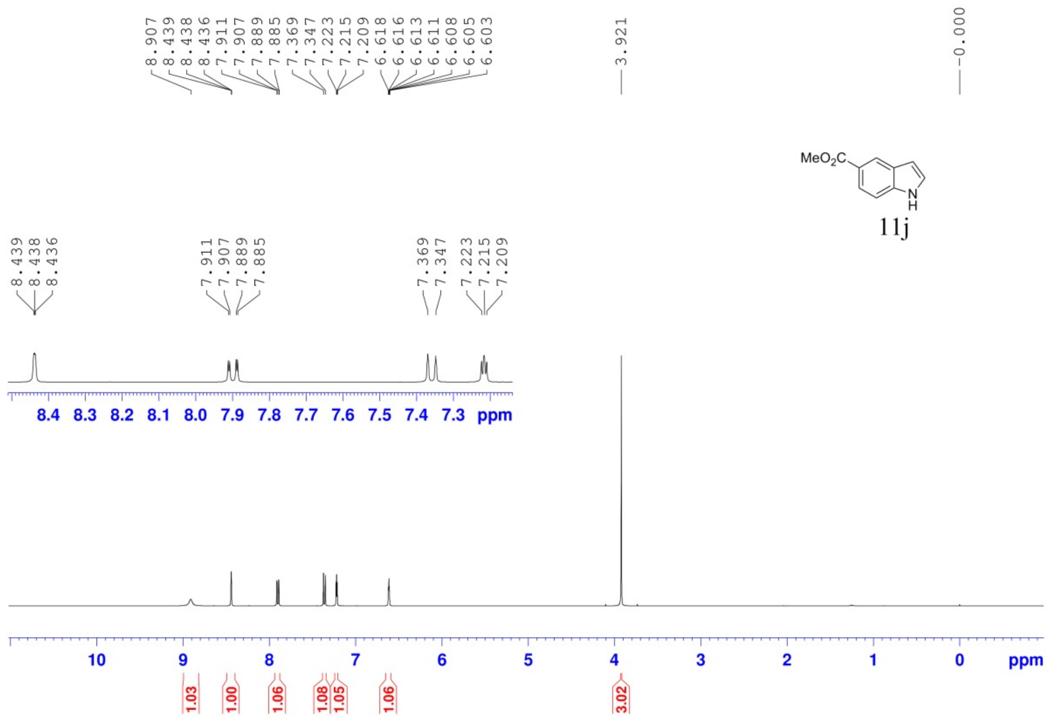

Supplementary Figure 145. ^{1}H NMR spectrum for methyl 1H-indole-5-carboxylate **(11j)**.

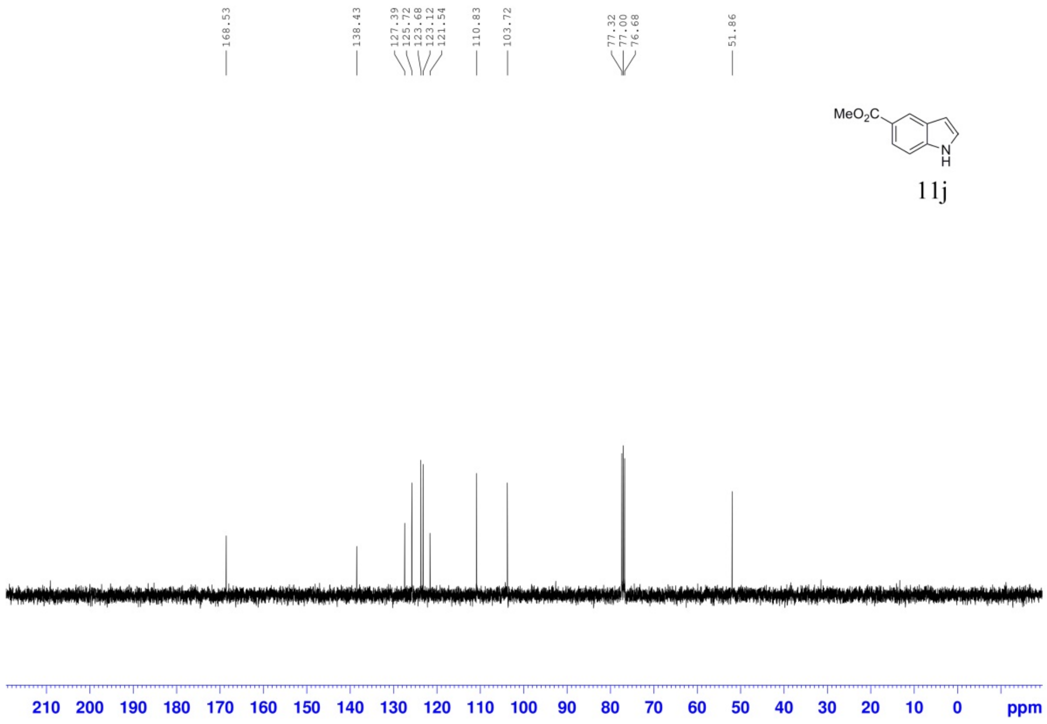

Supplementary Figure 146. ^{13}C NMR spectrum for methyl 1H-indole-5-carboxylate **(11j)**.

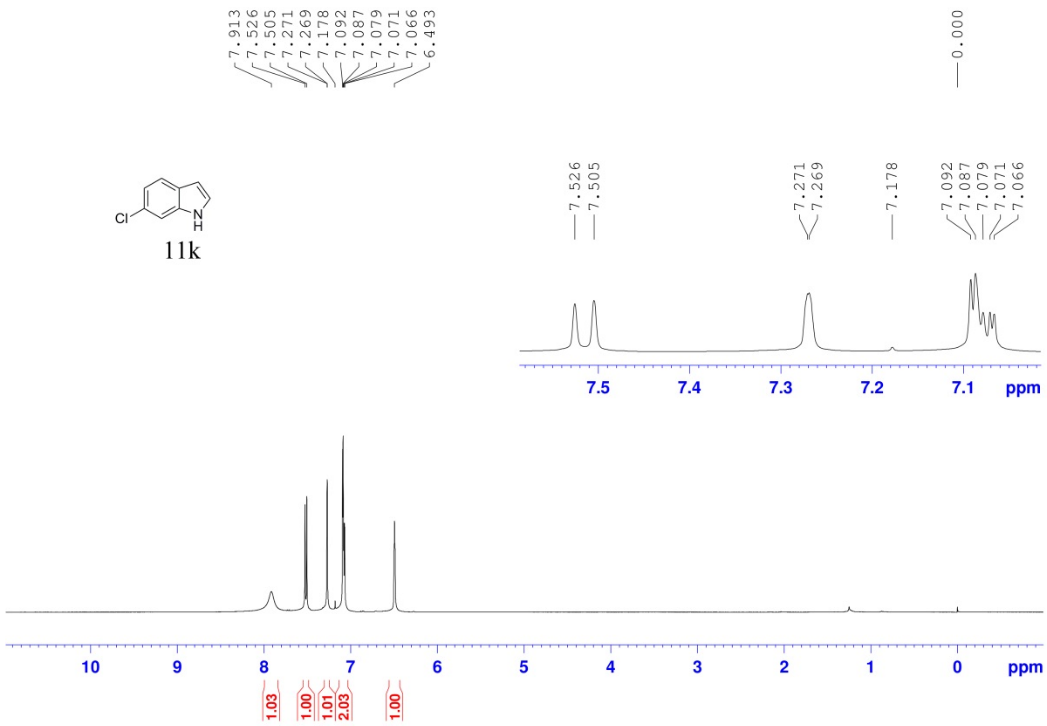

Supplementary Figure 147. ^{1}H NMR spectrum for 6-chloro-1H-indole **(11k)**.

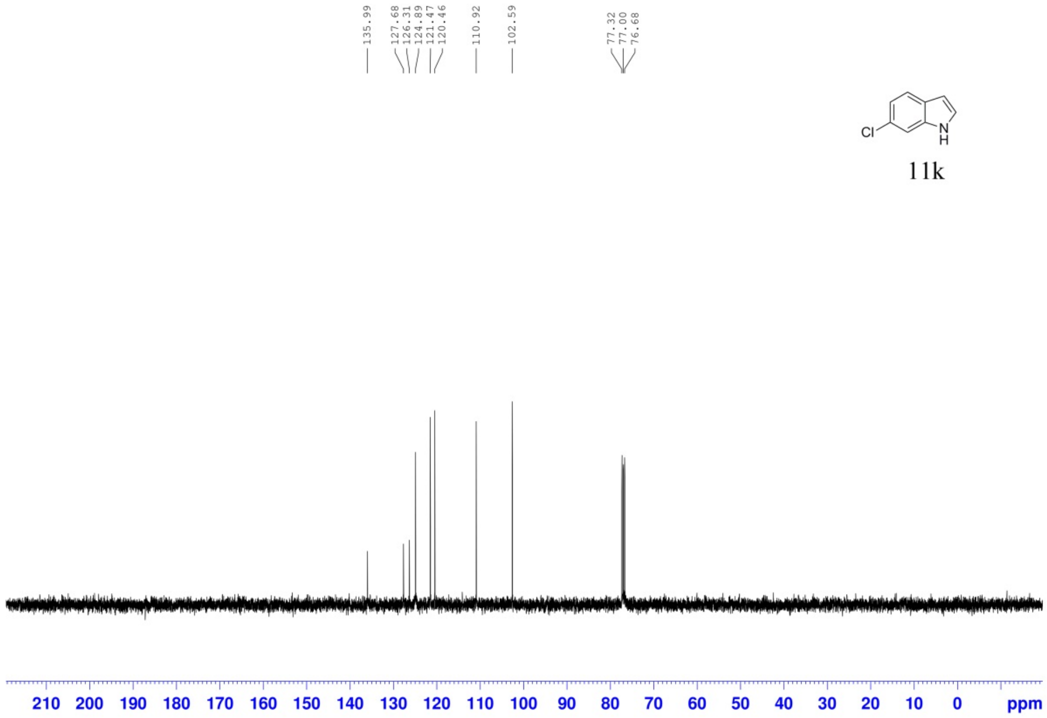

Supplementary Figure 148. ^{13}C NMR spectrum for 6-chloro-1H-indole **(11k)**.

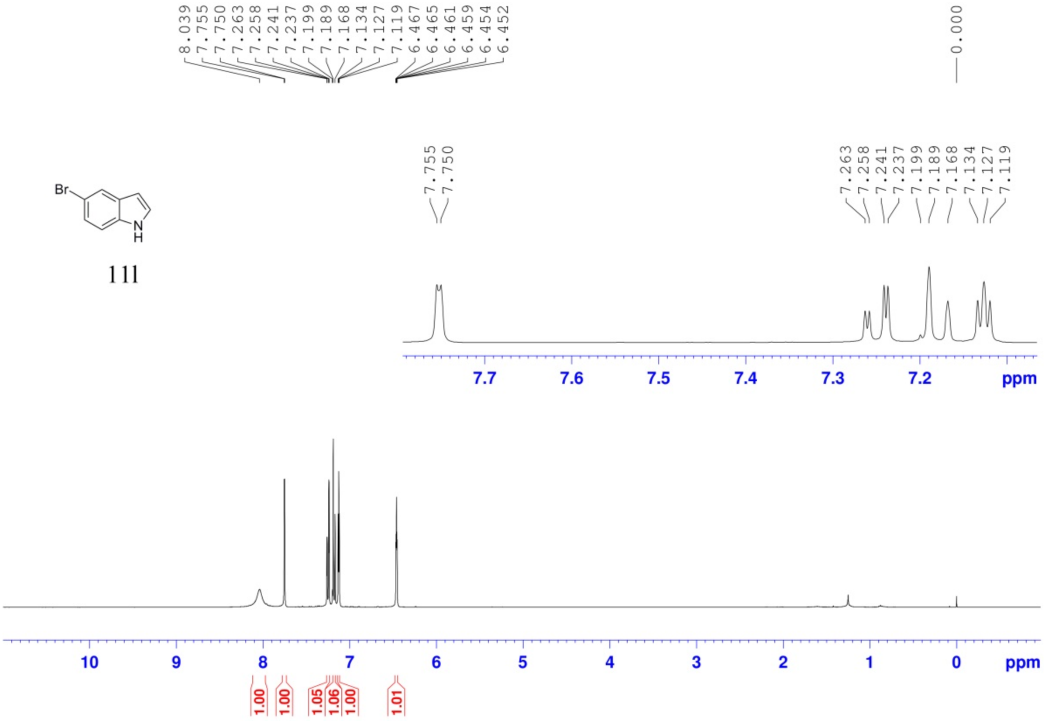

11l

Supplementary Figure 149. ^{1}H NMR spectrum for 5-bromo-1H-indole **(11l)**.

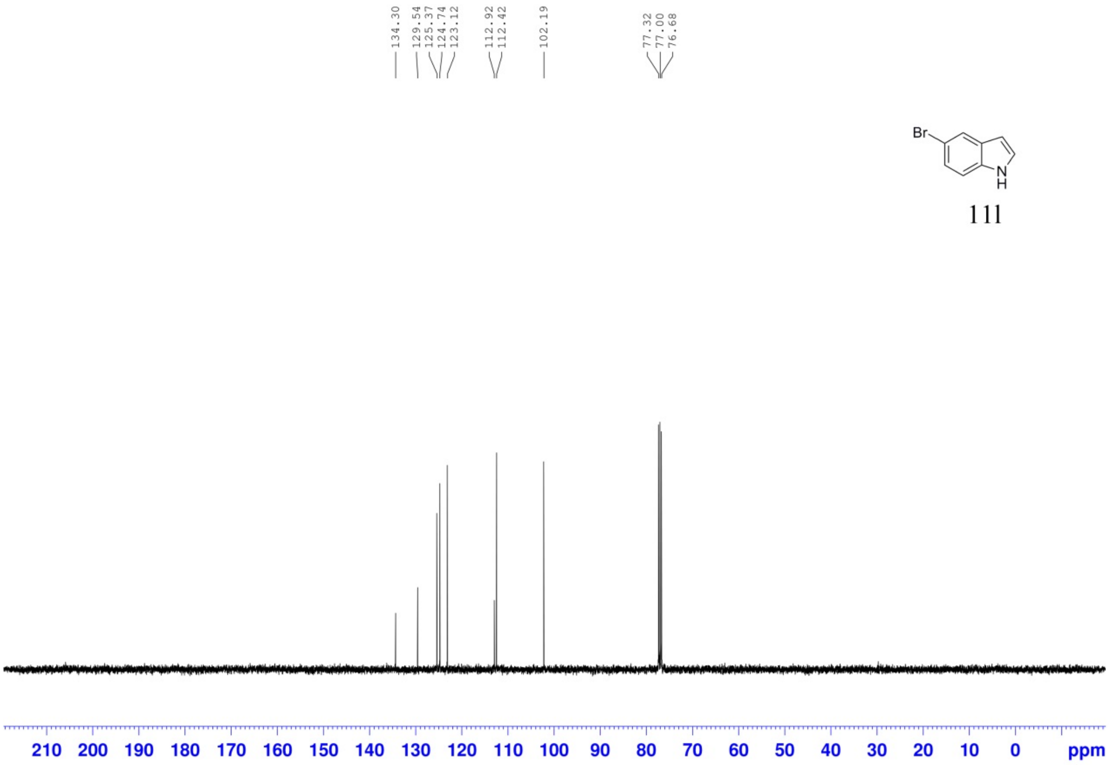

11l

Supplementary Figure 150. ^{13}C NMR spectrum for 5-bromo-1H-indole **(11l)**.

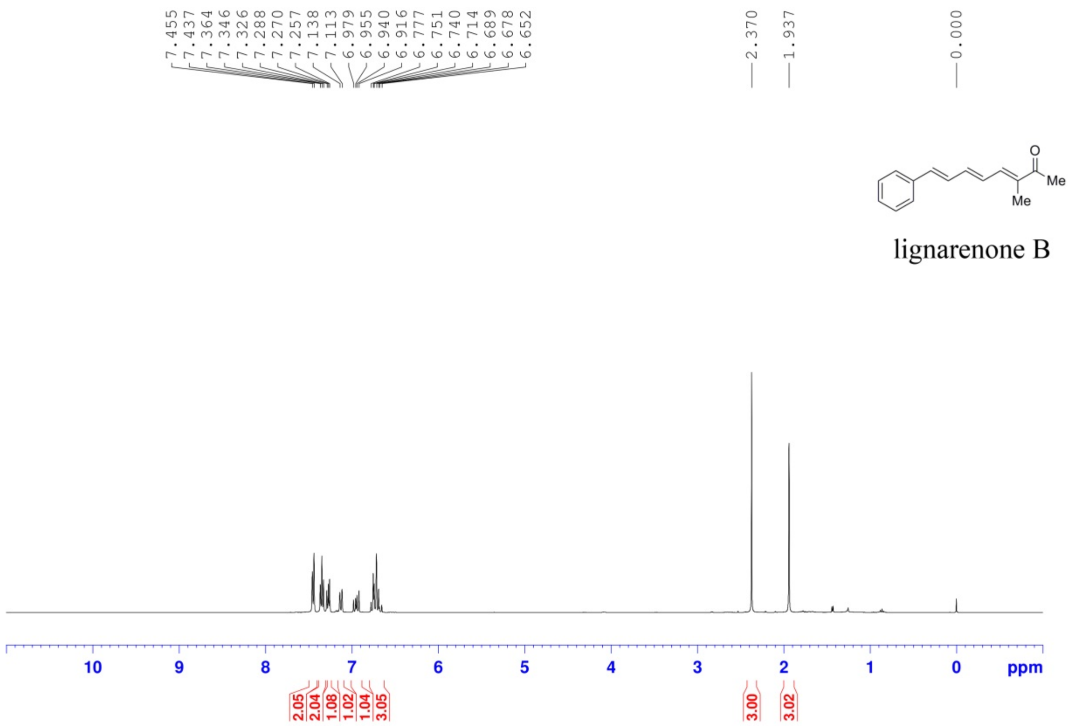

Supplementary Figure 151. ^{1}H NMR spectrum for lignarenone B.

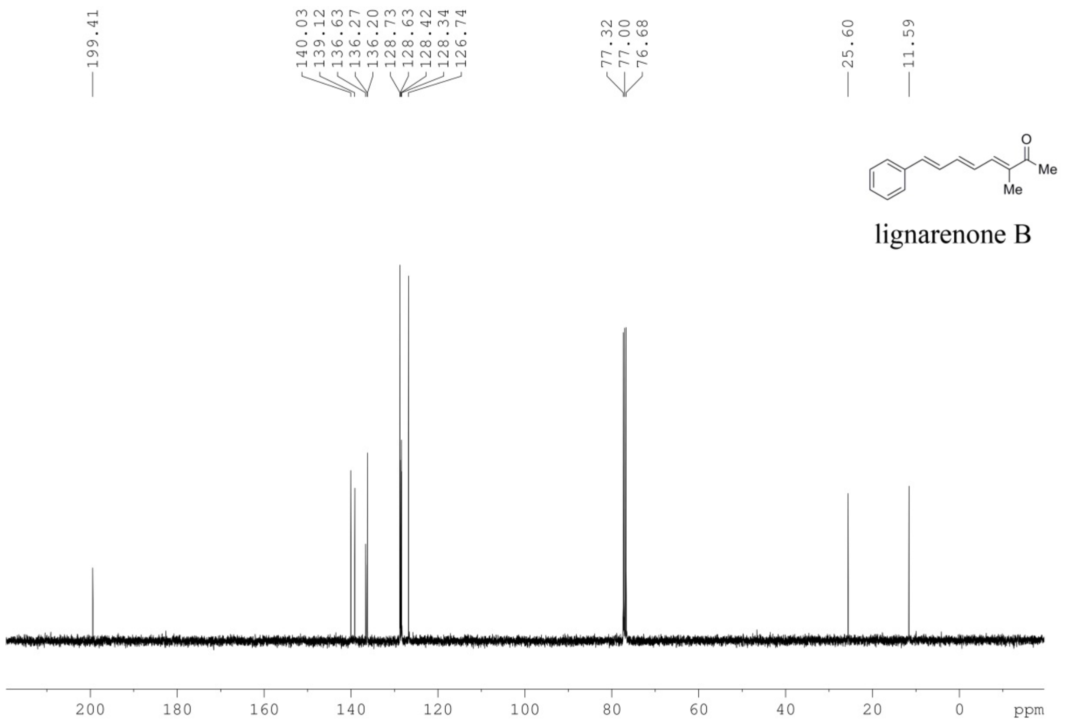

Supplementary Figure 152. ^{13}C NMR spectrum for lignarenone B.

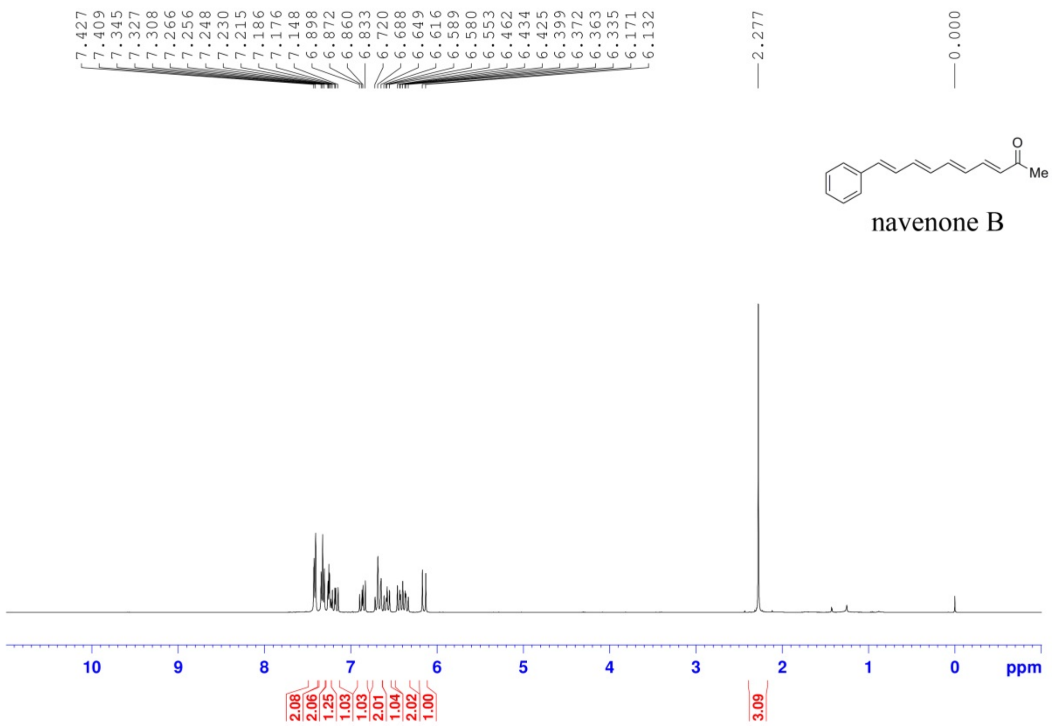

Supplementary Figure 153. ^{1}H NMR spectrum for navenone B.

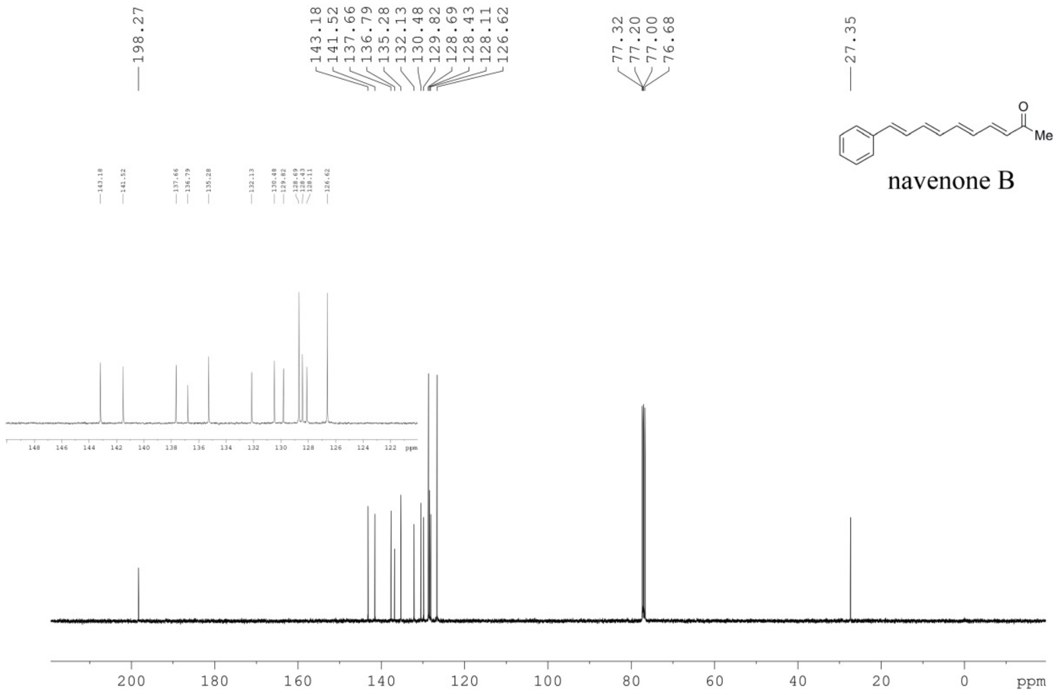

Supplementary Figure 154. ^{13}C NMR spectrum for navenone B.

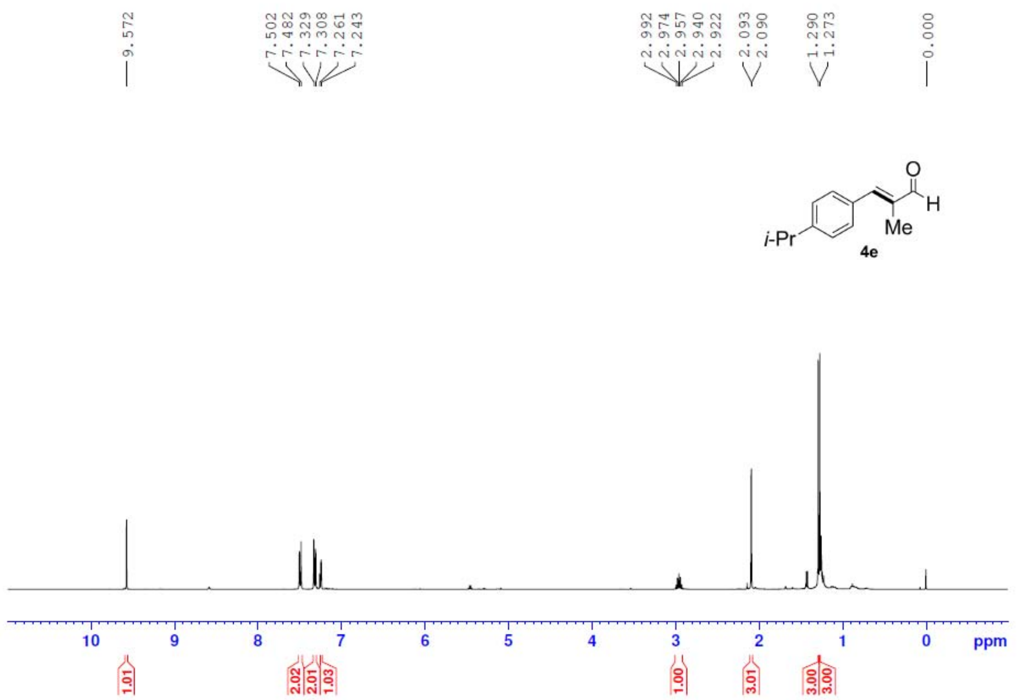

Supplementary Figure 155. ^{1}H NMR spectrum for (*E*)-3-(4-isopropylphenyl)-2-Methylacrylaldehyde.

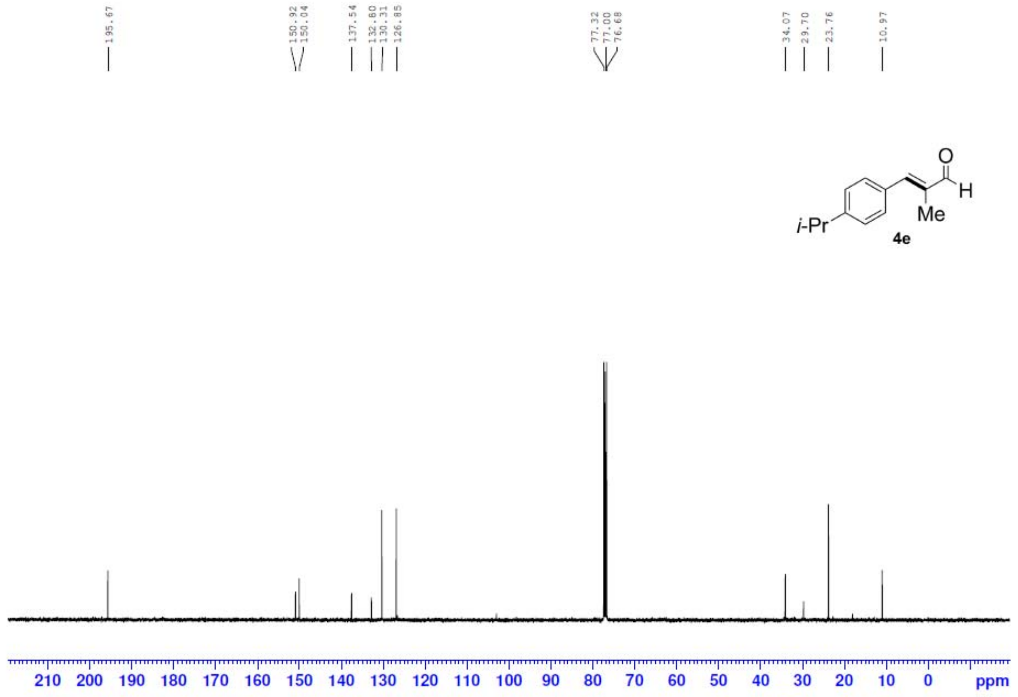

Supplementary Figure 156. ^{13}C NMR spectrum for (*E*)-3-(4-isopropylphenyl)-2-Methylacrylaldehyde.

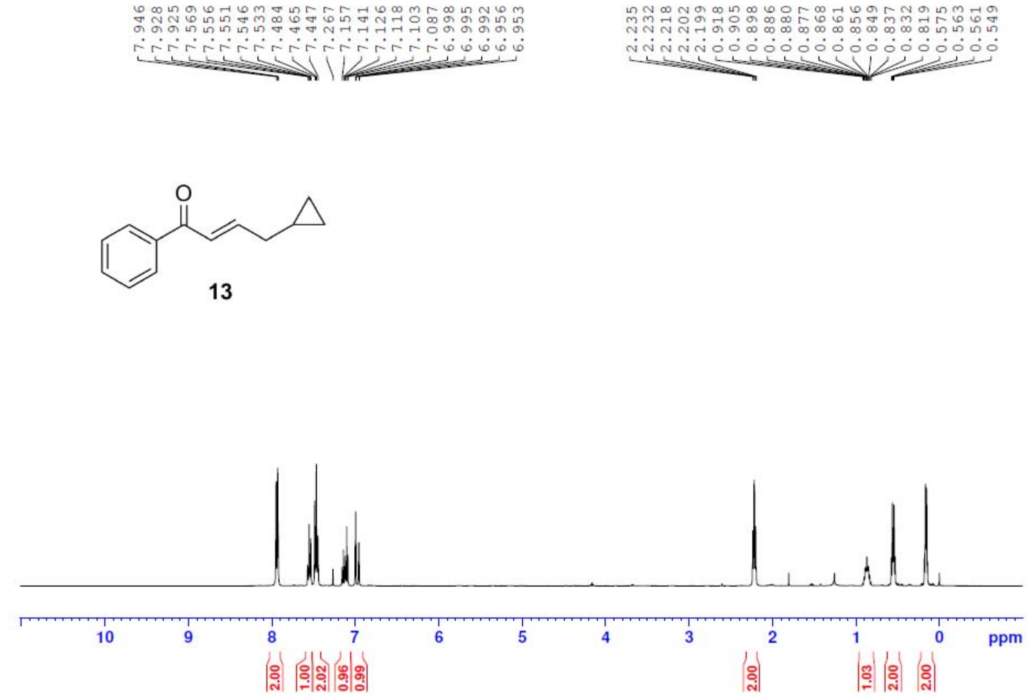

Supplementary Figure 157. ^{1}H NMR spectrum for (*E*)-4-cyclopropyl-1-phenylbut-2-en-1-one.

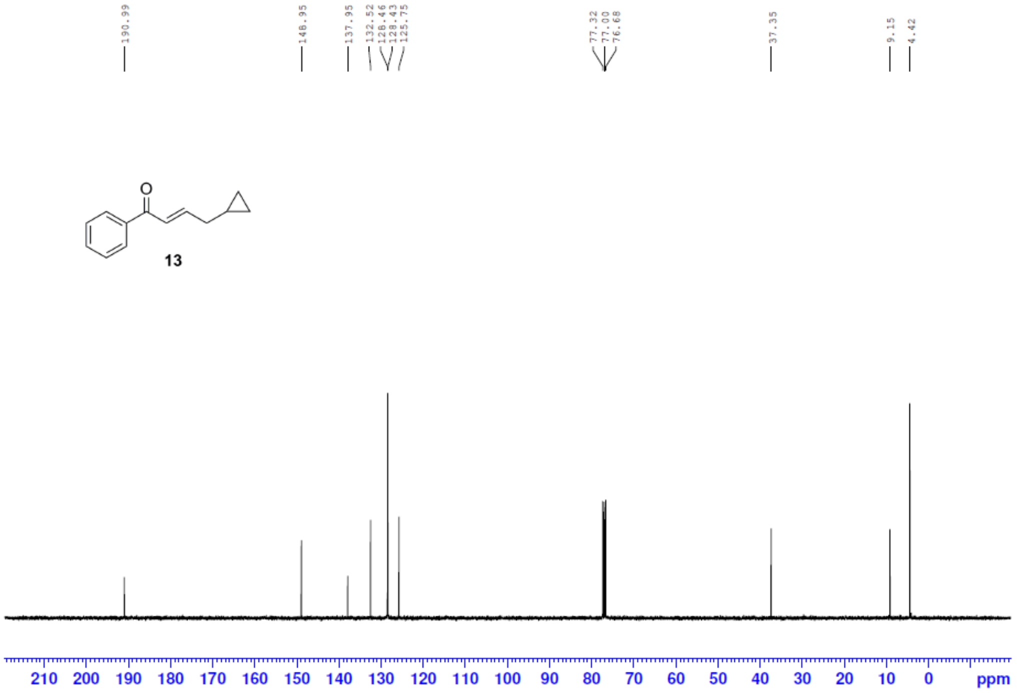

Supplementary Figure 158. ^{13}C NMR spectrum for (*E*)-4-cyclopropyl-1-phenylbut-2-en-1-one.

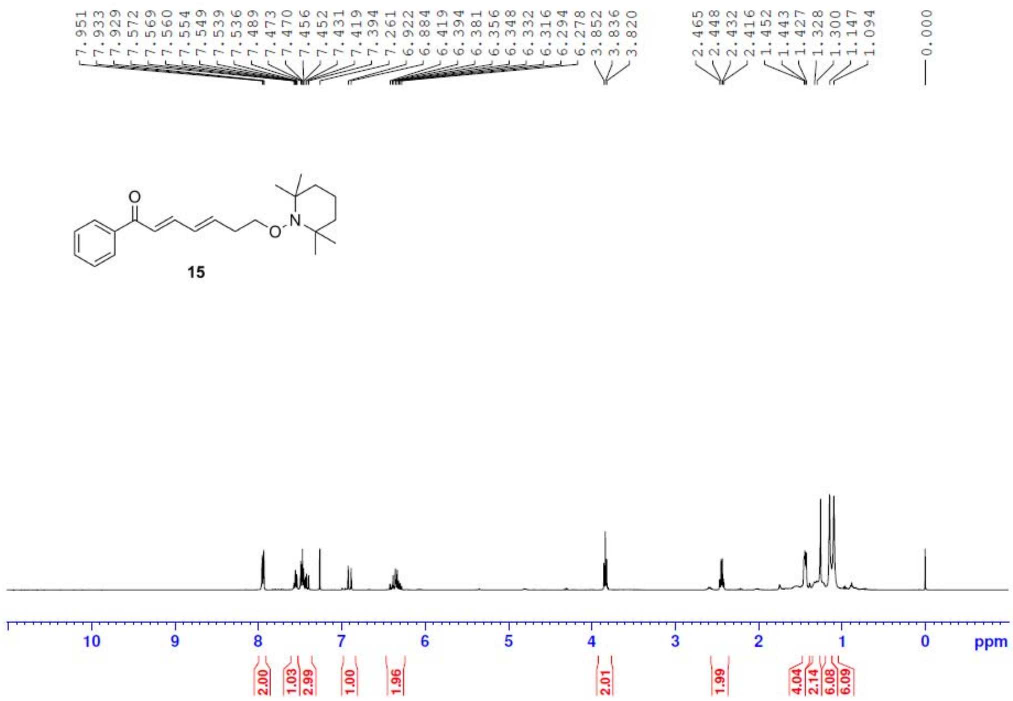

Supplementary Figure 159. ^{1}H NMR spectrum for **15.**

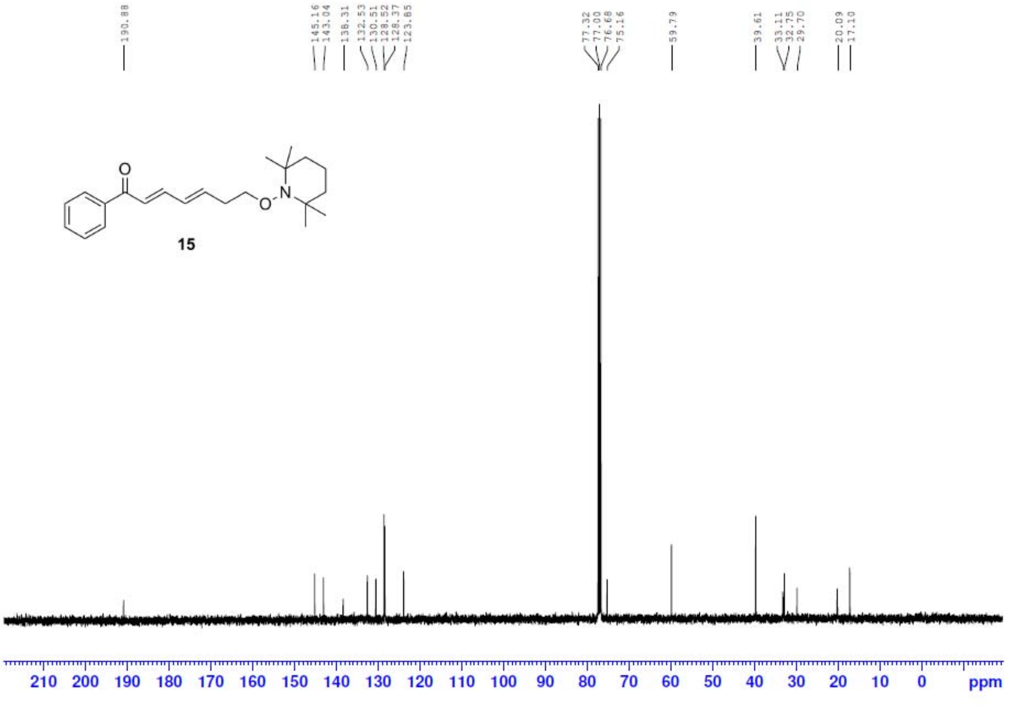

Supplementary Figure 160. ^{13}C NMR spectrum for **15.**

Supplementary Table 1. Optimization of the successive dehydrogenation reaction for 1,5-diphenylpentan-1-one (1a)[a]

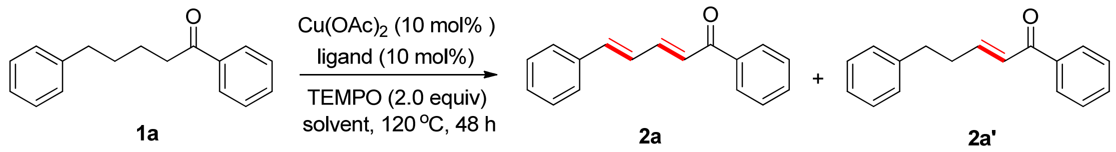

entry	ligand	solvent (1.0 mL)	conversion (%)	yield (%)[b]	
				**2a** (%)	**2a'**(%)
1	L1	1,2-dichlorobenzene	98	66	<5
2	L1	1,4-dioxane	80	54	<5
3	L1	DMF	79	61	<5
4	L1	toluene	85	47	<5
5	L1	*t*-AmylOH	93	61	<5
6	L2	1,2-dichlorobenzene	99	76	<5
**7**	L3	1,2-dichlorobenzene	94	75	<5
8	L4	1,2-dichlorobenzene	40	30	<5
9	L5	1,2-dichlorobenzene	87	68	<5
**10**	**L6**	**1,2-dichlorobenzene**	**99**	**85 (76)[c]**	**<5**
11	L7	1,2-dichlorobenzene	92	52	10
12	L8	1,2-dichlorobenzene	68	42	8
13	L9	1,2-dichlorobenzene	90	55	11
14	L10	1,2-dichlorobenzene	70	32	9
15	L11	1,2-dichlorobenzene	98	49	10
16	L12	1,2-dichlorobenzene	98	61	<5
17	L13	1,2-dichlorobenzene	96	56	<5
18	L14	1,2-dichlorobenzene	89	52	<5
19	L15	1,2-dichlorobenzene	93	51	10
20	L16	1,2-dichlorobenzene	98	61	8
21	L6 (20 mol%)	1,2-dichlorobenzene	95	70	<5
22	-	1,2-dichlorobenzene	43	29	<5

[a]Standard coditions : **1a** (0.2 mmol), $Cu(OAc)_2$ (10 mol%), Ligand (10 mol%), TEMPO (2.0 equiv), solvent (1.0 mL), 120 °C, N_2 atmosphere, 48 h. [b]Determined by GC using dodecane as an internal standard. [c]Isolated yield.

Supplementary Table 2. Optimization of the successive dehydrogenation reaction for in situ generated α,β-unsaturated diesters[a]

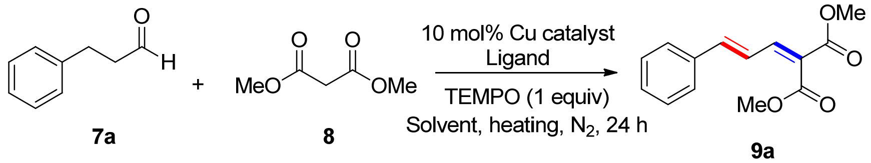

entry	Cu Source (10 mol%)	Ligand (L:Cu)	Solvent	Temp (°C)	Yield[a]
1	$Cu(OAc)_2$	L1 (1.5:1)	1,2-dichlorobenzene	100	59%
2	$Cu(OAc)_2$	L1 (1.5:1)	DME	100	48%
3	$Cu(OAc)_2$	L1 (1.5:1)	DMF	100	58%
4	$Cu(OAc)_2$	L1 (1.5:1)	*t*-Amyl alcohol	100	66%
5	$Cu(OAc)_2$	L1 (1.5:1)	toluene	100	49%
6	$Cu(OAc)_2$	L1 (1.5:1)	1,4-dioxane	100	32%
7	$Cu(OAc)_2$	L1 (1.5:1)	HFIP	100	0%
8	$Cu(OAc)_2$	L2 (1.5:1)	*t*-Amyl alcohol	100	77%
9	$Cu(OAc)_2$	L3 (1.5:1)	*t*-Amyl alcohol	100	63%
10	$Cu(OAc)_2$	L4 (1.5:1)	*t*-Amyl alcohol	100	27%
11	$Cu(OAc)_2$	L5 (1.5:1)	*t*-Amyl alcohol	100	72%
12	$Cu(OAc)_2$	L6 (1.5:1)	*t*-Amyl alcohol	100	50%
13	$Cu(OAc)_2$	L17 (1.5:1)	*t*-Amyl alcohol	100	72%

14	$Cu(OAc)_2$	L18 (1.5:1)	*t*-Amyl alcohol	100	46%
15	$Cu(OAc)_2$	L19 (1.5:1)	*t*-Amyl alcohol	100	45%
16	$Cu(OAc)_2$	L2 (1.5:1)	*t*-Amyl alcohol	120	81%[b]
**17**	**$Cu(OAc)_2$**	**L2 (2:1)**	***t*-Amyl alcohol**	**120**	**87%[b]**
18	$Cu(TFA)_2$	L2 (2:1)	*t*-Amyl alcohol	120	70%
19	$Cu(SO_2OCF_3)_2$	L2 (2:1)	*t*-Amyl alcohol	120	32%
20	CuF_2	L2 (2:1)	*t*-Amyl alcohol	120	56%
21	$CuBr_2$	L2 (2:1)	*t*-Amyl alcohol	120	trace
22	$CuCl_2$	L2 (2:1)	*t*-Amyl alcohol	120	trace
23	$CuSO_4$	L2 (2:1)	*t*-Amyl alcohol	120	34%
24	CuBr	L2 (2:1)	*t*-Amyl alcohol	120	trace
25	CuCl	L2 (2:1)	*t*-Amyl alcohol	120	trace
26	$Cu(OAc)_2$	L2 (2:1)	*t*-Amyl alcohol	120	trace[c]
27	$Cu(OAc)_2$	L2 (2:1)	*t*-Amyl alcohol	120	68[d]
28	$Cu(OAc)_2$	L2 (2:1)	*t*-Amyl alcohol	120	trace[e]
29	$Cu(OAc)_2$	L2 (2:1)	*t*-Amyl alcohol	120	60[f]
30	$Cu(OAc)_2$	L2 (2:1)	*t*-Amyl alcohol	120	71[b,g]

[a]Conditions: **7a** (0.2 mmol), **8** (0.5 mmol), Cu catalyst (10 mol%), Ligand, TEMPO (1.0 equiv), solvent (1.0 mL), N_2 atmosphere, 24 h. Yields were determined by GC analysis using dodecane as an internal standard. [b]Isolated yields. [c]1 atm O_2. [d]1 atm air; [e]20% TEMPO was used; [f]50% TEMPO was used; [g]The reaction was carried out for 14 h.

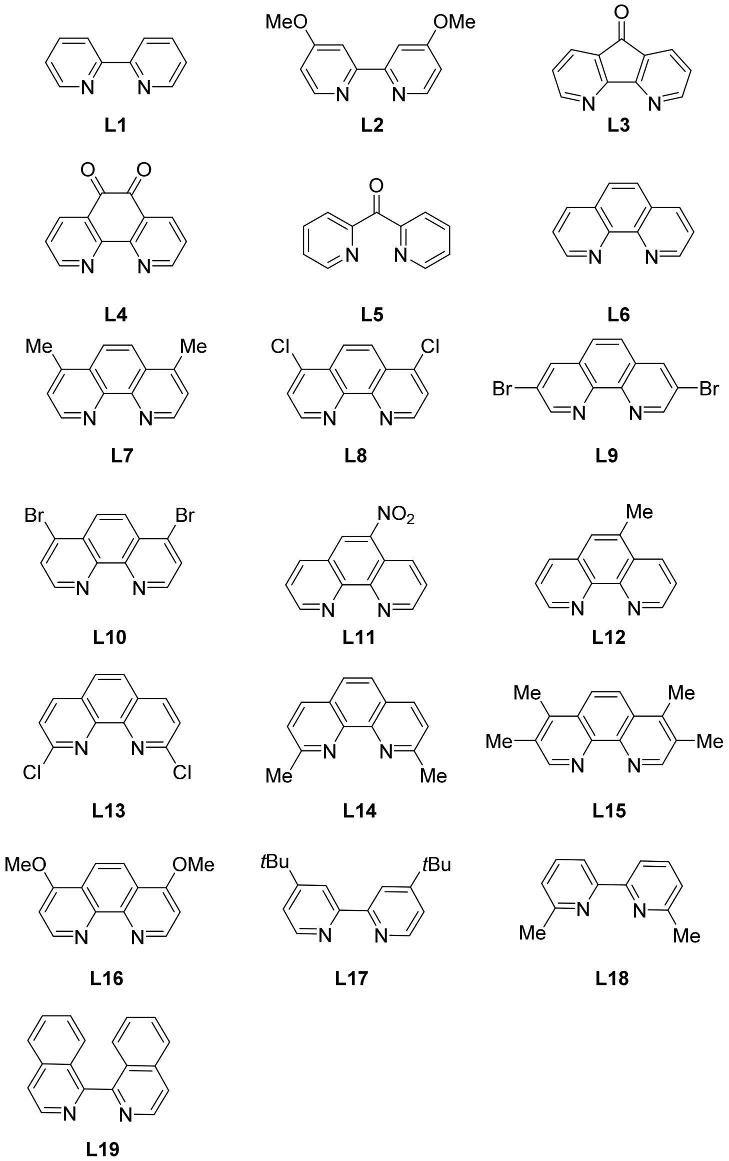

Supplementary Figure 161. Bidentate ligand screening

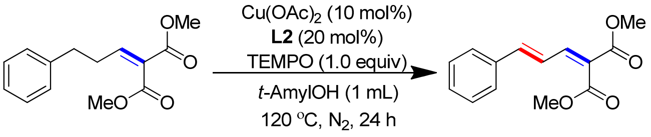

9a 82% isolated yield

The independently prepared **dimethyl 2-(3-phenylpropylidene)malonate** could be used as starting material to afford **9a** in 82% isolated yield under standard conditions. This result is in accordance with our proposed Knoevenagel condensation-dehydrogenation sequence.

Supplementary Methods

General Considerations. All reactions were conducted under a nitrogen atmosphere with dry solvents. Unless otherwise noted, materials were purchased from Sigma-Aldrich, Acros, Alfa Aesar, TCI and other commercial suppliers and used directly without further purification. Anhydrous $Cu(OAc)_2$ was purchased from Acros. 1,2-Dichlorobenzene, DMF and *tert*-amyl alcohol were distilled over CaH_2 and stored under nitrogen. Toluene, 1,4-dioxane and DME were distilled from Na and stored under nitrogen. ^{1}H NMR (400 MHz), ^{13}C NMR (100 MHz) and ^{19}F NMR (377 MHz) spectra were recorded in $CDCl_3$ solutions using a Bruker AVANCE 400 spectrometer. The chemical shift values (δ) were calibrated using TMS (0.00 ppm for ^{1}H) and residual undeuterated solvent $CHCl_3$ (77.0 ppm for ^{13}C). HRMS (High-resolution mass spectra) were performed by the Shanghai Mass Spectrometry Center in Shanghai Institute of Organic Chemistry, Chinese Academic of Sciences (Instrument: Thermo Fisher Scientific LTQ FT Ultra, Operation Mode: DART Positive).

Synthesis of 1,5-diphenylpentan-1-one (1a).[1]

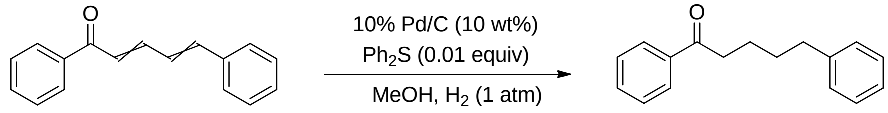

1a

To a 250 mL round bottom flask equipped with a stir bar was charged with cinnamylideneacetophenone (1.1715g, 5 mmol), 10% Pd/C (0.1172g, 10wt % of cinnamylideneacetophenone), and diphenylsulfide (8.2 μL, 0.01 equiv) in MeOH (10.0 mL). After three vacuum/H_2 cycles to replace air inside the flask with hydrogen, the round bottom flask was purged with a balloon pressure of hydrogen. The reaction mixture was stirred at room temperature for 48 h. After the reaction was finished, the mixture was filtered through a pad of silica gel and washed with 50.0 mL of ethyl acetate. The solvent was removed under reduced pressure, and the residue was purified by flash chromatography on silica gel (petroleum ether/diethyl ether = 100:5). The product **1a** was obtained in 90% yield.

Physical state: colorless oil;

HRMS (*m/z*): calculated for $C_{17}H_{18}OH^+$ $[M+H]^+$, 239.1430; found, 239.1430;

^{1}H NMR (400 MHz, $CDCl_3$): δ 7.92 (d, J = 7.4 Hz, 2H), 7.52 (t, J = 7.3 Hz, 1H), 7.44-7.40 (m, 2H), 7.28-7.24 (m, 2H), 7.20-7.14 (m, 3H), 2.97-2.93 (m, 2H), 2.67-2.63 (m, 2H), 1.82-1.66 (m, 4H);

^{13}C NMR (100 MHz, $CDCl_3$): δ 200.2, 142.2, 137.0, 132.8, 128.5, 128.3, 128.2, 128.0, 125.7, 38.3, 35.7, 31.0, 23.9.

Synthesis of 6-phenylhexan-2-one (1b) [1, 2]

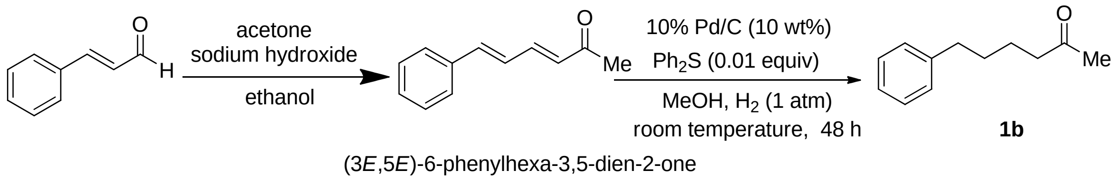

(1)To a well-stirred solution of *trans*-cinnamaldehyde (0.6608g, 5.0 mmol**)** and acetone (0.5808g, 10.0 mmol) in ethanol (50.0 mL) in a round bottom flask (250 mL) was added dropwise NaOH solution (5.0 mL, 6mol/L). The reaction mixture was stirred at room temperature for 6 h and quenched by 10% HCl solution (10.0 mL). After extracted by ethyl acetate (20.0 mL×3) and dried by Na_2SO_4, the combined organic layers were concentrated under reduced pressure and the crude (3*E*,5*E*)-6-phenylhexa-3,5-dien-2-one was obtained; (2) A 250 mL round bottom flask equipped with a stir bar was charged with the crude (3*E*,5*E*)-6-phenylhexa-3,5-dien-2-one (0.8610g, 5.0 mmol), 10% Pd/C (0.0861g, 10wt % of 3*E*,5*E*)-6-phenylhexa-3,5-dien-2-one) and diphenylsulfide (8.2 μL, 0.01 equiv) in MeOH (10.0 mL). After three vacuum/H_2 cycles to replace air inside the flask with hydrogen, the round bottom flask was purged with a balloon pressure of hydrogen. The reaction mixture was stirred at room temperature for 48 h. After the reaction was finished, the mixture was filtered through a pad of silica gel and washed with 50.0 mL of ethyl acetate. The solvent was removed under reduced pressure, and the residue was purified by flash chromatography on silica gel (petroleum ether/diethyl ether = 100:5), the product **1b** was obtained in 72% yield.

Physical state: colorless oil;

HRMS (*m/z*): calculated for $C_{12}H_{16}OH^+$ $[M+H]^+$, 177.1274; found, 177.1275;

^{1}H NMR (400 MHz, $CDCl_3$): δ 7.27-7.24 (m, 2H), 7.17-7.14 (m, 3H), 2.60 (t, *J* = 6.8 Hz, 2H), 2.41 (t, *J* = 6.6 Hz, 2H), 2.09 (s, 3H), 1.62-1.58 (m, 4H);

^{13}C NMR (100 MHz, $CDCl_3$): δ 208.7, 142.0, 128.2, 128.1, 125.6, 43.4, 35.6, 30.8, 29.7, 23.3.

Synthesis of 6-(furan-2-yl)hexan-2-one (1c) [1, 2]

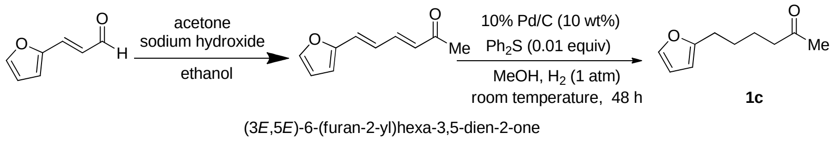

(1)To a well-stirred solution of 3-(2-furyl)acrolein (0.6106g, 5.0 mmol) and acetone (0.5808g, 10.0 mmol) in ethanol (50.0 mL) in a round bottom flask (250 mL) was added dropwise NaOH solution (5.0 mL, 6mol/L). The reaction mixture was stirred at room temperature for 6 h and quenched by 10% HCl solution (10.0 mL). After extracted by ethyl acetate (20.0 mL×3) and dried by Na_2SO_4, the combined organic layers were concentrated under reduced pressure and the crude (3*E*,5*E*)-6-(furan-2-yl)hexa-3,5-dien-2-one was obtained; (2) A 250 mL round bottom flask equipped with a stir bar was charged with the crude (3*E*,5*E*)-6-(furan-2-yl)hexa-3,5-dien-2-one (0.8109g, 5.0 mmol), 10% Pd/C (0.0811g, 10wt % of (3*E*,5*E*)-6-(furan-2-yl)hexa-3,5-dien-2-one) and diphenylsulfide (8.2 μL, 0.01 equiv) in MeOH (10.0 mL). After three vacuum/H_2 cycles to replace air inside the flask with hydrogen, the round bottom flask was purged with a balloon pressure of hydrogen. The reaction mixture was stirred at room temperature for 48 h. After the reaction was finished, the mixture was filtered through a pad of silica gel and washed with 50.0 mL of ethyl acetate. The solvent was removed under reduced pressure, and the residue was purified by flash chromatography on silica gel (petroleum ether/diethyl ether = 100:5), the product **1c** was obtained in 70% yield.

Physical state: colorless oil;

HRMS (*m/z*): calculated for $C_{10}H_{14}O_2H^+$ $[M+H]^+$,167.1067; found, 167.1067;

^{1}H NMR (400 MHz, $CDCl_3$): δ 7.284-7.280 (m, 1H), 6.26 (dd, *J* = 3.0, 1.9 Hz, 1H), 5.98 (dd, *J* = 3.0, 0.6 Hz, 1H), 2.63 (t, *J* = 6.8 Hz, 2H), 2.44 (t, *J* = 6.8 Hz, 2H), 2.12 (s, 3H), 1.65-1.62 (m, 4H);

^{13}C NMR (100 MHz, $CDCl_3$): δ 208.7, 155.7, 140.7, 110.0, 104.8, 43.2, 29.8, 27.6, 27.4, 23.1.

Synthesis of 3-cyclohexyl-1-phenylpropan-1-one (1e)[3]

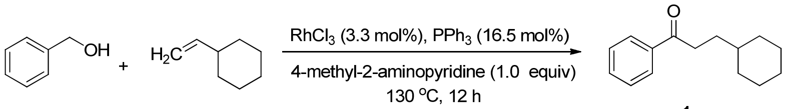

To a 100 mL Schlenk tube equipped with a stir bar was charged with benzyl alcohol (0.1550 g, 1.44mmol), vinylcyclohexane (1.5869g, 10.0 equiv), 4-methyl-2-aminopyridine (0.1560g, 1.0 equiv) and PPh_3 (0.0626g, 0.238 mmol, 16.5 mol%). After the reaction mixture was stirred for several minutes, the catalyst $RhCl_3$ (0.0100g, 0.048 mmol, 3.3 mol%) was added, and then the septum was replaced with a Teflon screwcap under nitrogen flow. The reaction mixture was stirred at 130 °C for 12 h. Upon cooling to room temperature, the reaction mixture was filtrated through a pad of silica gel, washed with 50 mL of ethyl acetate and concentrated under reduced pressure. The residue was purified by flash chromatography on silica gel (petroleum ether/ether = 100:10) to provide the product **1e** in 76% yield.

Physical state: white solid;

^{1}H NMR (400 MHz, $CDCl_3$): δ7.97-7.94 (m, 2H), 7.56-7.52 (m, 1H), 7.47-7.43 (m, 2H), 2.99-2.95 (m, 2H), 1.77-1.60 (m, 7H), 1.35-1.10 (m, 4H), 0.99-0.90 (m, 2H);

^{13}C NMR (100 MHz, $CDCl_3$): δ 200.7, 137.0, 132.8, 128.5, 128.0, 37.3, 36.1, 33.1, 31.7, 26.5, 26.2.

Synthesis of 5-phenylpentanal (3a).[4]

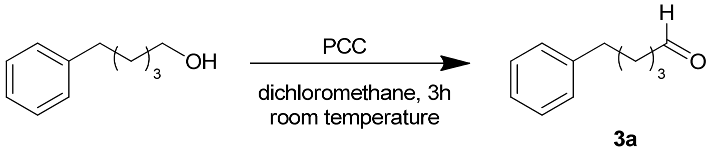

To a well-stirred solution of 5-phenyl-1-pentanol (0.8213g, 5mmol) in dry dichloromethane (15.0 mL) in a 25 mL reaction tube was added Celite (Celite : alcohol = 1:1, wt/wt) and PCC (pyridinium chlorochromate, 2.6945g, 2.5 equiv) , and then the reaction mixture was stirred at room temperature for 3h. After the reaction was finished, the mixture was filtered through a pad of silica gel and washed with 50.0 mL of ethyl acetate. The solvent was removed under reduced pressure, and the residue was purified by flash chromatography on silica gel (petroleum ether/ethyl ether = 100:10), the product **3a** was obtained in 65% yield.

Physical state: colorless oil;

^{1}H NMR (400 MHz, $CDCl_3$): δ 9.75 (t, *J* = 1.7 Hz, 1H), 7.30-7.26 (m, 2H), 7.20-7.16 (m, 3H), 2.66-2.62 (m, 2H), 2.47-2.43 (m, 2H), 1.69-1.65 (m, 4H);

^{13}C NMR (100 MHz, $CDCl_3$): δ 202.6, 141.9, 128.4, 128.3, 125.8, 43.7, 35.6, 30.9, 21.6.

Synthesis of 2-phenylpentanal (3b).[1]

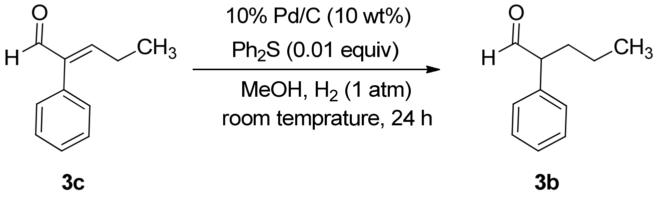

To a 250 mL round bottom flask equipped with a stir bar was charged with 2-phenyl-2-pentenal **3c** (5 mmol, 0.8011g), 10% Pd/C (0.0801g, 10wt % of **3c**), and diphenylsulfide (8.2 μL, 0.01 equiv) in MeOH (10.0 mL). After three vacuum/H_2 cycles to replace air inside the flask with hydrogen, the round bottom flask was purged with a balloon pressure of hydrogen. The reaction mixture was stirred at room temperature for 48 h. After the reaction was finished, the mixture was filtered through a pad of silica gel and washed with 50.0 mL of ethyl acetate. The solvent was removed

under reduced pressure, and the residue was purified by flash chromatography on silica gel (petroleum ether/diethyl ether = 100:5). The product **3b** was obtained in 55% yield. This compound is known.[5] The ^{1}H NMR spectra data is in good agreement with the literature data.

Physical state: colorless oil;

HRMS (*m*/*z*): calculated for $C_{11}H_{14}OH^+$ $[M+H]^+$, 163.0754; found, 163.0755;

^{1}H NMR (400 MHz, $CDCl_3$): δ 9.66 (s, 1H), 7.39-7.29 (m, 3H), 7.19 (d, *J* = 7.7 Hz, 2H), 3.50 (t, *J* = 7.3 Hz, 1H), 2.09-2.00 (m, 1H), 1.77-1.67 (m, 1H), 1.32-1.26 (m, 2H), 0.92 (t, *J* = 7.3 Hz, 3H).

Synthesis of methyl 6-hydroxyhexanoate (5d).[6]

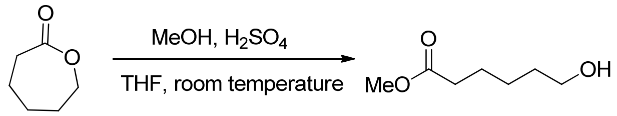

5d

To a well-stirred solution of ε-caprolactone (1.1414, 10 mmol) in MeOH (10.0 mL) was added dropwise concentrated aqueous sulfuric acid (adjusting to pH to 6 using pH paper). The reaction mixture was stirred for 20 min. After the reaction was finished, the reaction mixture was diluted with ethyl acetate (25.0 mL) and washed with brine (20.0 mL). The aqueous layer was extracted with ethyl acetate (20.0 mL) three times. Then, the combined organic layers were dried over anhydrous Na_2SO_4, and concentrated under reduced pressure. The residue was purified by flash chromatography on silica gel (petroleum ether/ethyl acetate = 100:10), the product **5d** was obtained in 75% yield.

Physical state: colorless oil;

HRMS (*m*/*z*): calculated for $C_7H_{14}O_3NH_4^+$ $[M+NH_4]^+$, 164.1281; found,164.1282;

^{1}H NMR (400 MHz, $CDCl_3$): δ 3.67-3.63 (m, 5H), 2.33 (t, J = 7.5 Hz, 2H), 1.73-1.55 (m, 4H), 1.49-1.37 (m, 3H);

^{13}C NMR (100 MHz, $CDCl_3$): δ 174.3, 62.1, 51.4, 33.8, 32.0, 25.1, 24.5.

Synthesis of 6-hydroxy-*N*-methyl-*N*-phenylhexanamide (5e).[7]

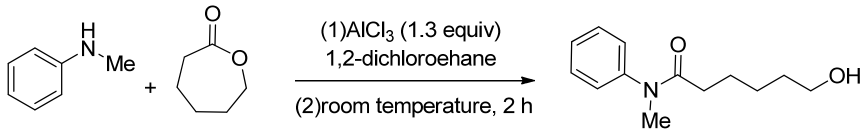

5e

To a well-stirred suspension of $AlCl_3$ (1.7334, 13 mmol) in dry 1,2-dichloroethane (10.0 mL) in the 100 mL round bottom flask was added dropwise *N*-methylaniline (2.6790g, 25.0 mmol) under the ice bath condition and then the achieved solution was stirred at room temperature for 10 minutes. Then ε-caprolactone (1.1414g, 10 mmol) was added dropwise to this solution and the mixture was stirred at room temperature for 2 h. After the reaction was finished, the reaction mixture was quenched with a mixture of ice and water (20.0 mL) and stirred for a further 0.5 h. Followed by filtration through a pad of silica gel, the organic layer was separated and the aqueous layer was extracted with 1,2-dichloroethane (20.0 mL) for three times. The combined organic layers were washed with brine and dried by Na_2SO_4. After concentration under reduced pressure, the residue was purified by flash chromatography on silica gel (petroleum ether/ethyl acetate = 100:15), the product **5e** was obtained in 61% yield.

Physical state: yellow oil;

HRMS (*m*/*z*): calculated for $C_{13}H_{19}NO_2H^+$ $[M+H]^+$, 222.1489; found, 222.1488;

^{1}H NMR (400 MHz, $CDCl_3$): δ 7.44-7.40 (m, 2H), 7.34 (t, *J* = 7.2 Hz, 1H), 7.17 (d, *J* = 7.5 Hz, 2H), 3.58 (t, *J* = 6.4 Hz, 2H), 3.26 (s, 3H), 2.21 (brs, 1H), 2.08 (t, *J* = 7.3 Hz, 2H), 1.63-1.55 (m, 2H), 1.52-1.45 (m, 2H), 1.31-1.23 (m, 2H);

^{13}C NMR (100 MHz, $CDCl_3$): δ 173.1, 144.0, 129.7, 127.7, 127.2, 62.3, 37.2, 33.9, 32.2, 25.2, 24.9.

Synthesis of 3-phenylpropanal compounds (7b-7p)[8]

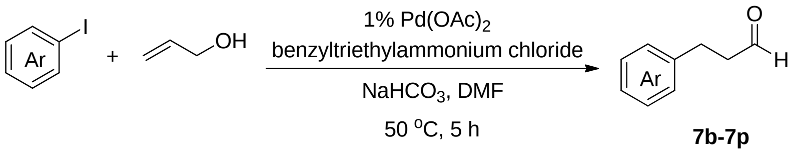

7b-7p

General procedure: In a nitrogen-filled glovebox, a 100 mL Schlenk tube equipped with a stir bar was charged with substituted iodobenzene (5.0 mmol), $Pd(OAc)_2$ (0.0113g, 0.1 mmol, 1 mol%), benzyltriethylammonium chloride (1.1389g, 1.0 equiv) and $NaHCO_3$ (1.050g, 2.5 equiv). The tube was fitted with a rubber septum and moved out of the glovebox. Then allyl alcohol (0.4356g, 1.5 equiv) and DMF (20.0 mL) were added in turn to the Schlenk tube through the rubber septum using syringes, and then the septum was replaced with a Teflon screwcap under nitrogen flow. The reaction mixture was stirred at 50 °C for 5 h. Upon cooling to room temperature, the reaction mixture was filtrated through a pad of silica gel, washed with 50 mL of ethyl acetate and washed twice with water (20.0 mL). The organic layer was dried over anhydrous Na_2SO_4 and concentrated under reduced pressure. The residue was purified by flash chromatography on silica gel to provide the corresponding product in 50 – 90 % yields.

Synthesis of 3-(*p*-tolyl)propanal (7b)

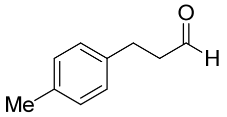

7b

This substrate was synthesized following the *general procedure*. The Schlenk tube was charged with 4-iodotoluene (1.0902g, 5mmol), $Pd(OAc)_2$ (0.0113g, 0.1 mmol, 1 mol%), benzyltriethylammonium chloride (1.1389g, 1.0 equiv), $NaHCO_3$ (1.050g, 2.5 equiv) and allyl alcohol (0.4356g, 1.5 equiv) in DMF (20.0 mL) and the reaction mixture was stirred at 50 °C for 5 h. After concentration and purification by flash chromatography on silica gel (petroleum ether/diethyl ether = 100:5), the product **7b** was obtained.

Physical state: colorless oil;

^{1}H NMR (400 MHz, $CDCl_3$): δ 9.77 (s, 1H), 7.10-7.05 (m, 4H), 2.89 (t, J = 7.5 Hz, 2H), 2.72 (t, J = 7.3 Hz, 2H), 2.30 (s, 3H);

^{13}C NMR (100 MHz, $CDCl_3$): δ 201.6, 137.1, 135.6, 129.1, 128.0, 45.2, 27.6, 20.8.

Synthesis of 3-(m-tolyl)propanal (7c)

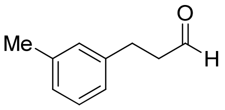

7c

This substrate was synthesized following the *general procedure*. The Schlenk tube was charged with 3-iodotoluene (1.0902g, 5mmol), $Pd(OAc)_2$ (0.0113g, 0.1 mmol, 1 mol%), benzyltriethylammonium chloride (1.1389g, 1.0 equiv), $NaHCO_3$ (1.050g, 2.5 equiv) and allyl alcohol (0.4356g, 1.5 equiv) in DMF (20.0 mL) and the reaction mixture was stirred at 50 ^{o}C for 5 h. After concentration and purification by flash chromatography on silica gel (petroleum ether/diethyl ether = 100:5), the product **7c** was obtained.

Physical state: colorless oil;

^{1}H NMR (400 MHz, $CDCl_3$): δ 9.78 (s, 1H), 7.21-7.15 (m, 1H), 7.02-6.97 (m, 3H), 2.90 (t, J = 7.5 Hz. 2H), 2.73 (t, J = 7.5 Hz, 2H), 2.31 (s, 3H);

^{13}C NMR (100 MHz, $CDCl_3$): δ 201.6, 140.2, 138.1, 129.0, 128.4, 126.9, 125.2, 45.2, 27.9, 21.3.

Synthesis of 3-(4-fluorophenyl)propanal (7d)

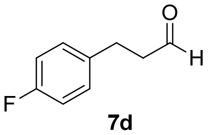

7d

This substrate was synthesized following the *general procedure*. The Schlenk tube was charged with 1-fluoro-4-iodobenzene (1.1100g, 5mmol), $Pd(OAc)_2$ (0.0113g, 0.1 mmol, 1 mol%), benzyltriethylammonium chloride (1.1389g, 1.0 equiv), $NaHCO_3$ (1.050g, 2.5 equiv) and allyl alcohol (0.4356g, 1.5 equiv) in DMF (20.0 mL) and the reaction mixture was stirred at 50 ^{o}C for 5 h. After concentration and purification by

flash chromatography on silica gel (petroleum ether/diethyl ether = 100:5), the product **7d** was obtained.

Physical state: colorless oil;

^{1}H NMR (400 MHz, $CDCl_3$): δ 9.80 (d, *J* = 1.1 Hz, 1H), 7.17-7.12 (m, 2H), 7.00-6.94 (m, 2H), 2.92 (t, *J* = 7.4 Hz, 2H), 2.76 (t, *J* = 7.4 Hz, 2H);

^{13}C NMR (100 MHz, $CDCl_3$): δ 201.2, 161.3 (d, *J* = 244.2 Hz), 135.9 (d, *J* = 3.3 Hz), 129.6 (d, *J* = 7.9 Hz), 115.2 (d, *J* = 21.2 Hz), 45.2, 27.1.

^{19}F NMR (377 MHz): δ -117.0.

Synthesis of 3-(4-chlorophenyl)propanal (7e)

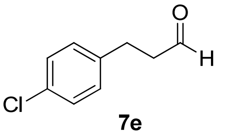

7e

This substrate was synthesized following the *general procedure*. The Schlenk tube was charged with 1-chloro-4-iodobenzene (1.1922g, 5mmol), $Pd(OAc)_2$ (0.0113g, 0.1 mmol, 1 mol%), benzyltriethylammonium chloride (1.1389g, 1.0 equiv), $NaHCO_3$ (1.050g, 2.5 equiv) and allyl alcohol (0.4356g, 1.5 equiv) in DMF (20.0 mL) and the reaction mixture was stirred at 50 °C for 5 h. After concentration and purification by flash chromatography on silica gel (petroleum ether/diethyl ether = 100:5), the product **7e** was obtained.

Physical state: colorless oil;

^{1}H NMR (400 MHz, $CDCl_3$): δ 9.8 (s, 1H), 7.27-7.24 (m, 2H), 7.12 (d, *J* = 8.3 Hz, 2H), 2.92 (t, *J* = 7.4 Hz, 2H), 2.77 (t, *J* = 7.4 Hz, 2H);

^{13}C NMR (100 MHz, $CDCl_3$): δ 201.0, 138.8, 132.0, 129.6, 128.6, 45.1, 27.4.

Synthesis of 3-(3-chlorophenyl)propanal (7f)

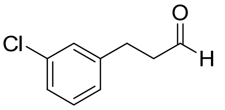

7f

This substrate was synthesized following the *general procedure*. The Schlenk tube was charged with 1-chloro-3-iodobenzene (1.1922g, 5mmol), $Pd(OAc)_2$ (0.0113g, 0.1 mmol, 1 mol%), benzyltriethylammonium chloride (1.1389g, 1.0 equiv), $NaHCO_3$ (1.050g, 2.5 equiv) and allyl alcohol (0.4356g, 1.5 equiv) in DMF (20.0 mL) and the reaction mixture was stirred at 50 °C for 5 h. After concentration and purification by flash chromatography on silica gel (petroleum ether/diethyl ether = 100:5), the product **7f** was obtained.

Physical state: colorless oil;

^{1}H NMR (400 MHz, $CDCl_3$): δ 9.81 (s, 1H), 7.24-7.17(m, 3H), 7.07 (d, *J* = 7.2 Hz, 1H), 2.93 (t, *J* = 7.4 Hz, 2H), 2.78 (t, *J* = 7.4 Hz, 2H);

^{13}C NMR (100 MHz, $CDCl_3$): δ 200.9, 142.3, 134.2, 129.8, 128.4, 126.5, 126.4, 44.9, 27.6.

Synthesis of 3-(2-chlorophenyl)propanal (7g)

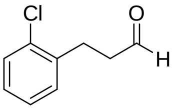

7g

This substrate was synthesized following the *general procedure*. The Schlenk tube was charged with 1-chloro-2-iodobenzene (1.1922g, 5mmol), $Pd(OAc)_2$ (0.0113g, 0.1 mmol, 1 mol%), benzyltriethylammonium chloride (1.1389g, 1.0 equiv), $NaHCO_3$ (1.050g, 2.5 equiv) and allyl alcohol (0.4356g, 1.5 equiv) in DMF (20.0 mL) and the reaction mixture was stirred at 50 °C for 5 h. After concentration and purification by flash chromatography on silica gel (petroleum ether/diethyl ether = 100:5), the product **7g** was obtained.

Physical state: colorless oil;

^{1}**H NMR** (400 MHz, $CDCl_3$): δ 9.82 (s, 1H), 7.36-7.33 (m, 1H), 7.25-7.14 (m, 3H), 3.06 (t, J = 7.4 Hz, 2H), 2.82-2.78 (m, 2H);

^{13}**C NMR** (100 MHz, $CDCl_3$): δ 201.2, 137.9, 133.8, 130.5, 129.6, 127.8, 126.9, 43.4, 26.1.

Synthesis of 3-(4-bromophenyl)propanal (7h)

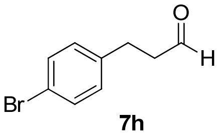

7h

This substrate was synthesized following the *general procedure*. The Schlenk tube was charged with 1-bromo-4-iodobenzene (1.4146g, 5mmol), $Pd(OAc)_2$ (0.0113g, 0.1 mmol, 1 mol%), benzyltriethylammonium chloride (1.1389g, 1.0 equiv), $NaHCO_3$ (1.050g, 2.5 equiv) and allyl alcohol (0.4356g, 1.5 equiv) in DMF (20.0 mL) and the reaction mixture was stirred at 50 °C for 5 h. After concentration and purification by flash chromatography on silica gel (petroleum ether/diethyl ether = 100:5), the product **7h** was obtained.

Physical state: pale yellow oil;

^{1}**H NMR** (400 MHz, $CDCl_3$): δ 9.76 (d, J = 1.3 Hz, 1H), 7.38 (d, J = 8.3 Hz, 2H), 7.05 (d, J = 8.3 Hz, 2H), 2.88 (t, J = 7.4 Hz, 2H), 2.76-2.72 (m, 2H);

^{13}**C NMR** (100 MHz, $CDCl_3$): δ 201.0, 139.3, 131.6, 130.1, 120.0, 45.0, 27.4.

Synthesis of 3-(3-bromophenyl)propanal (7i)

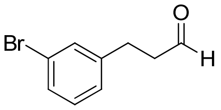

7i

This substrate was synthesized following the *general procedure*. The Schlenk tube was charged with 1-bromo-3-iodobenzene (1.4146g, 5mmol), $Pd(OAc)_2$ (0.0113g, 0.1 mmol, 1 mol%), benzyltriethylammonium chloride (1.1389g, 1.0 equiv), $NaHCO_3$ (1.050g, 2.5 equiv) and allyl alcohol (0.4356g, 1.5 equiv) in DMF (20.0 mL) and the reaction mixture was stirred at 50 °C for 5 h. After concentration and purification by

flash chromatography on silica gel (petroleum ether/diethyl ether = 100:5), the product **7i** was obtained.

Physical state: pale yellow oil;

^{1}H NMR (400 MHz, $CDCl_3$): δ 9.80 (s, 1H), 7.34-7.32 (m, 2H), 7.18-7.11 (m, 2H), 2.92 (t, J = 7.4 Hz, 2H), 2.78 (t, J = 7.4 Hz, 2H);

^{13}C NMR (100 MHz, $CDCl_3$): δ 200.8, 142.7, 131.3, 130.1, 129.4, 127.0, 122.5, 44.9, 27.6.

Synthesis of 3-(4-nitrophenyl)propanal (7j)

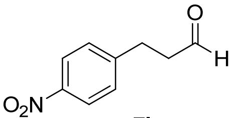

7j

This substrate was synthesized following the *general procedure*. The Schlenk tube was charged with 1-iodo-4-nitrobenzene (1.2451g, 5mmol), $Pd(OAc)_2$ (0.0113g, 0.1 mmol, 1 mol%), benzyltriethylammonium chloride (1.1389g, 1.0 equiv), $NaHCO_3$ (1.050g, 2.5 equiv) and allyl alcohol (0.4356g, 1.5 equiv) in DMF (20.0 mL) and the reaction mixture was stirred at 50 ^{o}C for 5 h. After concentration and purification by flash chromatography on silica gel (petroleum ether/diethyl ether = 100:10), the product **7j** was obtained.

Physical state: yellow solid;

^{1}H NMR (400 MHz, $CDCl_3$): δ 9.84 (s, 1H), 8.15 (d, J = 8.6 Hz, 2H), 7.38 (d, J = 8.6 Hz, 2H), 3.07 (t, J = 7.3 Hz, 2H), 2.88 (t, J = 7.3 Hz, 2H);

^{13}C NMR (100 MHz, $CDCl_3$): δ 200.1, 148.2, 146.5, 129.2, 123.7, 44.4, 27.7.

Synthesis of methyl 4-(3-oxopropyl)benzoate (7k)

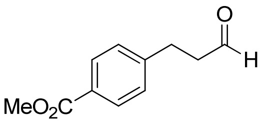

7k

This substrate was synthesized following the *general procedure*. The Schlenk tube was charged with methyl 4-iodobenzoate (1.3103g, 5mmol), $Pd(OAc)_2$ (0.0113g, 0.1 mmol,

1 mol%), benzyltriethylammonium chloride (1.1389g, 1.0 equiv), $NaHCO_3$ (1.050g, 2.5 equiv) and allyl alcohol (0.4356g, 1.5 equiv) in DMF (20.0 mL) and the reaction mixture was stirred at 50 °C for 5 h. After concentration and purification by flash chromatography on silica gel (petroleum ether/diethyl ether = 100:15), the product **7k** was obtained.

Physical state: white solid;

^{1}H NMR (400 MHz, $CDCl_3$): δ 9.80 (t, *J* = 1.1 Hz, 1H), 7.96-7.94 (m, 2H), 7.26 (d, *J* = 8.3 Hz, 2H), 3.88 (s, 3H), 2.99 (t, *J* = 7.5 Hz, 2H), 2.82-2.78 (m, 2H);

^{13}C NMR (100 MHz, $CDCl_3$): δ 200.7, 166.7, 145.7, 129.6, 128.1, 128.0, 51.8, 44.5, 27.7.

Synthesis of 3-(4-methoxyphenyl)propanal (7l)

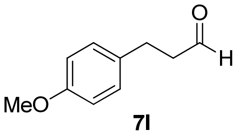

7l

This substrate was synthesized following the *general procedure*. The Schlenk tube was charged with methyl 4-iodoanisole (1.1702g, 5mmol), $Pd(OAc)_2$ (0.0113g, 0.1 mmol, 1 mol%), benzyltriethylammonium chloride (1.1389g, 1.0 equiv), $NaHCO_3$ (1.050g, 2.5 equiv) and allyl alcohol (0.4356g, 1.5 equiv) in DMF (20.0 mL) and the reaction mixture was stirred at 50 °C for 5 h. After concentration and purification by flash chromatography on silica gel (petroleum ether/diethyl ether = 100:10), the product **7l** was obtained.

Physical state: colorless oil;

^{1}H NMR (400 MHz, $CDCl_3$): δ 9.78 (t, *J* = 1.1 Hz, 1H), 7.11-7.09 (m, 2H), 6.84-6.81 (m, 2H), 3.76 (s, 3H), 2.89 (t, *J* = 7.5 Hz, 2H), 2.72 (t, *J* = 7.5 Hz, 2H);

^{13}C NMR (100 MHz, $CDCl_3$): δ 201.7, 157.9, 132.2, 129.1, 113.8, 55.1, 45.4, 27.1.

Synthesis of 3-(4-(trifluoromethyl)phenyl)propanal (7m)

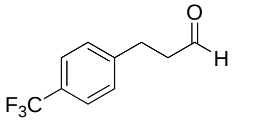

7m

This substrate was synthesized following the *general procedure*. The Schlenk tube was charged with 4-iodobenzotrifluoride (1.3601g, 5mmol), $Pd(OAc)_2$ (0.0113g, 0.1 mmol, 1 mol%), benzyltriethylammonium chloride (1.1389g, 1.0 equiv), $NaHCO_3$ (1.050g, 2.5 equiv) and allyl alcohol (0.4356g, 1.5 equiv) in DMF (20.0 mL) and the reaction mixture was stirred at 50 ^oC for 5 h. After concentration and purification by flash chromatography on silica gel (petroleum ether/diethyl ether = 100:5), the product **7m** was obtained.

Physical state: colorless oil;

^{1}H NMR (400 MHz, $CDCl_3$): δ 9.78 (t, *J* = 1.1 Hz, 1H), 7.53 (d, *J* = 7.9 Hz, 2H), 7.30 (d, *J* = 7.9 Hz, 2H), 2.99 (t, *J* = 7.2 Hz, 2H), 2.81-2.77 (m, 2H);

^{13}C NMR (100 MHz, $CDCl_3$): δ 200.6, 144.6, 128.4 (q, *J* = 32.4 Hz), 127.9, 125.3 (q, *J* = 3.6Hz), 124.2 (q, *J* = 271.7 Hz), 44.6, 27.6;

^{19}F NMR (377 MHz): δ -62.41.

Synthesis of 3-(naphthalen-2-yl)propanal (7n)

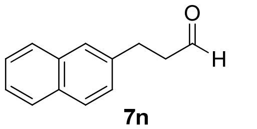

7n

This substrate was synthesized following the *general procedure*. The Schlenk tube was charged with 2-iodonaphthalene (1.2704g, 5mmol), $Pd(OAc)_2$ (0.0113g, 0.1 mmol, 1 mol%), benzyltriethylammonium chloride (1.1389g, 1.0 equiv), $NaHCO_3$ (1.050g, 2.5 equiv) and allyl alcohol (0.4356g, 1.5 equiv) in DMF (20.0 mL) and the reaction mixture was stirred at 50 ^oC for 5 h. After concentration and purification by flash chromatography on silica gel (petroleum ether/diethyl ether = 100:5), the product **7n** was obtained.

Physical state: pale yellow oil;

^{1}H NMR (400 MHz, $CDCl_3$): δ 9.79 (s, 1H), 7.93 (d, J = 8.2 Hz, 1H), 7.84-7.81 (m, 1H), 7.7 (d, J = 8.2 Hz, 1H), 7.51-7.43 (m, 2H), 7.38-7.34 (m, 1H), 7.29-7.27 (d, J = 1H), 3.35 (t, J = 7.7 Hz, 2H), 2.84-2.80 (m, 2H).
^{13}C NMR (100 MHz, $CDCl_3$): δ 201.4, 136.2, 133.8, 131.4, 128.8, 127.1, 126.0, 125.8, 125.6, 125.5, 123.2, 44.3, 25.0.

Synthesis of 3-(thiophen-2-yl)propanal (7o)

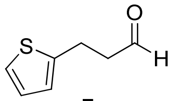

7o

This substrate was synthesized following the *general procedure*. The Schlenk tube was charged with 2-iodothiophene (1.0502g, 5mmol), $Pd(OAc)_2$ (0.0113g, 0.1 mmol, 1 mol%), benzyltriethylammonium chloride (1.1389g, 1.0 equiv), $NaHCO_3$ (1.050g, 2.5 equiv) and allyl alcohol (0.4356g, 1.5 equiv) in DMF (20.0 mL) and the reaction mixture was stirred at 50 ^{o}C for 5 h. After concentration and purification by flash chromatography on silica gel (petroleum ether/diethyl ether = 100:6), the product **7o** was obtained.
Physical state: pale yellow oil;
^{1}H NMR (400 MHz, $CDCl_3$): δ 9.80 (s, 1H), 7.13-7.11 (m, 1H), 6.92-6.90 (m, 1H), 6.81-6.80 (m, 1H), 3.16 (t, J = 7.3 Hz, 2H), 2.84-2.80 (m, 2H);
^{13}C NMR (100 MHz, $CDCl_3$): δ 200.8, 142.8, 126.8, 124.6, 123.5, 45.2, 22.2.

Synthesis of 3-(pyridin-3-yl)propanal (7p)

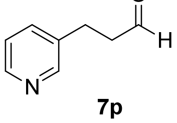

7p

This substrate was synthesized following the *general procedure*. The Schlenk tube was charged with 3-iodopyridine (1.0250g, 5mmol), $Pd(OAc)_2$ (0.0113g, 0.1 mmol, 1 mol%), benzyltriethylammonium chloride (1.1389g, 1.0 equiv), $NaHCO_3$ (1.050g, 2.5 equiv) and allyl alcohol (0.4356g, 1.5 equiv) in DMF (20.0 mL) and the reaction mixture was stirred at 50 ^{o}C for 5 h. After concentration and purification by flash

chromatography on silica gel (petroleum ether/ethyl acetate = 100:15), the product **7p** was obtained.

Physical state: colorless oil;

^{1}H NMR (400 MHz, $CDCl_3$): δ 9.82 (s, 1H), 8.48-8.45 (m, 2H), 7.54 (d, J = 7.8 Hz, 1H), 7.22 (dd, J_1 = 7.7 Hz, J_2 = 4.8 Hz, 1H), 2.96 (t, J = 7.3 Hz, 2H), 2.82 (t, J = 7.3 Hz, 2H);

^{13}C NMR (100 MHz, $CDCl_3$): δ 200.4, 149.5, 147.5, 135.7, 135.6, 123.2, 44.5, 24.9.

Synthesis of 7-phenylheptanal (7u).[4]

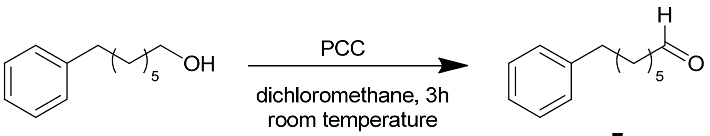

7u

To a well-stirred solution of 7-phenyl-1-heptanol (0.9615g, 5 mmol) in dry dichloromethane (15.0 mL) in a 25 mL reaction tube was added Celite (Celite : alcohol = 1:1, wt/wt) and PCC (pyridinium chlorochromate, 2.6945g, 2.5 equiv) , and then the reaction mixture was stirred at room temperature for 3h. After the reaction was finished, the mixture was filtered through a pad of silica gel and washed with 50.0 mL of ethyl acetate. The solvent was removed under reduced pressure, and the residue was purified by flash chromatography on silica gel (petroleum ether/ethyl ether = 100:10), the product **7u** was obtained in 68% yield.

Physical state: colorless oil;

^{1}H NMR (400 MHz, $CDCl_3$): δ 9.74 (t, J = 1.8 Hz, 1H), 7.29-7.24 (m, 2H), 7.18-7.15 (m. 3H), 2.60 (t, J = 7.7 Hz, 2H), 2.42-2.38 (m, 2H), 1.65-1.58 (m, 4H), 1.39-1.26 (m, 4H).

^{13}C NMR (100 MHz, $CDCl_3$): δ 202.7, 142.6, 128.3, 128.2, 125.6, 43.8, 35.8, 31.2, 29.0, 28.9, 21.9.

Synthesis of (*E*)-1,5-diphenylpent-2-en-1-one (2a')

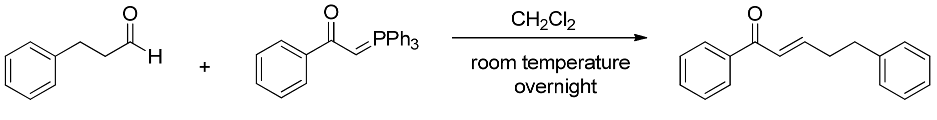

To a solution of (benzoylmethylene)triphenyl phosphorane (1.7118g, 4.5 mmol) in CH_2Cl_2 (6.0 mL) was added 3-phenylpropionaldehyde (0.4025g, 3.0 mmol). The reaction mixture was stirred at 80°C for 30 h. After the reaction was finished, the reaction mixture was concentrated under reduced pressure, and then purified by flash chromatography on silica gel (petroleum ether/ethyl ether = 100:5), the product **2a'** was obtained in 80% yield.

Physical state: pale yellow oil;

HRMS (*m/z*): calculated for $C_{17}H_{16}OH^+$ $[M+H]^+$, 237.1274; found, 237.1274;

^{1}H NMR (400 MHz, $CDCl_3$): δ 7.88-7.86 (m, 2H), 7.54 (t, *J* = 7.4 Hz, 1H), 7.46-7.42 (m, 2H), 7.32-7.28 (m, 2H), 7.24-7.19 (m, 3H), 7.07 (td, *J* = 15.4, 6.9 Hz, 1H), 6.88-6.84 (m, 1H), 2.86-2.82 (m, 2H), 2.66-2.61 (m, 2H);

^{13}C NMR (100 MHz, $CDCl_3$): δ 190.8, 148.4, 140.8, 137.8, 132.6, 128.51, 128.48, 128.46, 128.37, 126.5, 126.2, 34.49, 34.46.

Synthesis of 3-(triphenylphosphoranylidene)butan-2-one [9]

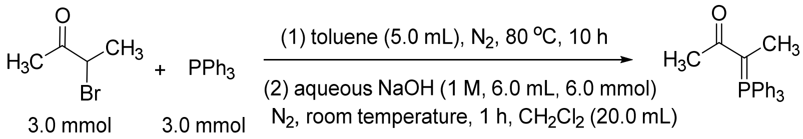

(1) To a solution of 3-bromo-2-butanone (0.4530g, 3.0 mmol) in toluene (5.0 mL) under nitrogen was added triphenylphosphine (0.7860g, 3.0 mmol). The reaction mixture was stirred at 80 ° C for 10 h. After the reaction mixture was cooled to room temperature, the resulting precipitate was filtered by vacuum filtration, washed with ethyl acetate (3×10 mL), and concentrated under vacuum to give the corresponding phosphonium salt; (2) the corresponding phosphonium salt was dissolved in dichloromethane (20 mL) under nitrogen , and then aqueous sodium hydroxide (1 M, 6.0 mL, 6.0 mmol) was added. The mixture was stirred at room temperature for 1 h. After the reaction was finished, the reaction mixture was extracted with

dichloromethane (3×10.0 mL). The combined organic fractions were washed with saturated brine (2×20.0 mL), and dried by anhydrous Na_2SO_4. The organic layer was concentrated under vacuum to give the corresponding stabilized phosphonium ylides [3-(triphenylphosphoranylidene)butan-2-one] in 70% yield.

Physical state: white solid;

HRMS (*m/z*): calculated for $C_{22}H_{21}OPH^+$ $[M+H]^+$, 333.1403; found, 333.1402;

^{1}H NMR (400 MHz, $CDCl_3$): δ 7.60-7.55 (m, 6H), 7.52-7.48 (m, 3H), 7.45-7.41 (m, 6H), 2.13 (s, 3H), 1.66 (d, J_{P-H} = 15.5 Hz, 3H);

^{13}C NMR (100 MHz, $CDCl_3$): δ 187.8, 133.2 (d, *J* = 9.6 Hz), 131.07 (d, *J* = 2.6 Hz), 128.2 (d, *J* = 12.0 Hz), 127.4 (d, *J* = 90.0 Hz), 55.8 (d, *J* = 107.1 Hz), 25.1 (d, *J* = 10.7 Hz), 14.0 (d, *J* = 14.5 Hz); **^{31}P NMR** (162 MHz, $CDCl_3$) : δ 17.62.

Synthesis the authentic γ-TEMPO-substituted enone intermediate 12[10]

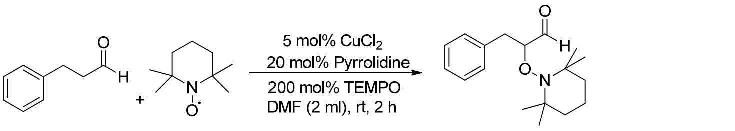

(eq.1)

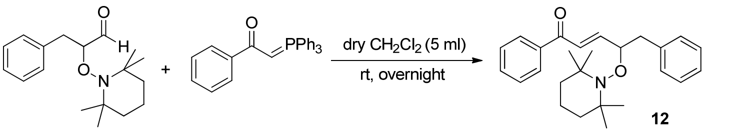

(eq.2)

General procedure: (Eq.1) In a nitrogen-filled glovebox, a 100 mL Schlenk tube equipped with a stir bar was charged with 3-phenylpropionaldehyde (0.4025g, 3.0 mmol), $CuCl_2$ (0.0256g, 0.15 mmol, 5 mol%), pyrrodidine (0.0427g, 0.6 mmol, 20 mol%) and TEMPO (0.9360g, 2.0 equiv). The tube was fitted with a rubber septum and moved out of the glovebox. Then DMF (2.0 mL) was added to the Schlenk tube through the rubber septum using syringes, and then the septum was replaced with a Teflon screwcap under nitrogen flow. The reaction mixture was stirred at room temperature for 2 h. After the reaction was finished, the reaction mixture was filtrated through a pad of silica gel, extracted with 50 mL of ethyl acetate and washed twice with water (20.0 mL). The organic layer was dried over anhydrous Na_2SO_4 and concentrated under reduced pressure. The residue was directly used in the next step reaction without further purification.

(Eq. 2) The above-mentioned residue was added to a solution of (benzoylmethylene)triphenyl phosphorane (0.3804g, 1.0 mmol) in CH_2Cl_2 (5.0 mL). The reaction mixture was stirred at room temperature overnight. After the reaction was finished, the reaction mixture was concentrated under reduced pressure, and then purified by flash chromatography on silica gel (petroleum ether/ether = 100:5), the product **12** was obtained in overall 12% yield.

Physical state: slight red oil;

HRMS (*m/z*): calculated for $C_{26}H_{33}O_2NH^+$ $[M+H]^+$, 392.2584; found, 392.2584;

^{1}H NMR (400 MHz, $CDCl_3$): δ 7.78-7.76 (m, 2H), 7.51 (t, *J* = 7.4 Hz, 1H), 7.42-7.39 (m, 2H), 7.28-7.23 (m, 2H), 7.20-7.17 (m, 3H), 6.95 (dd, *J* = 15.6, 8.2 Hz, 1H), 6.64 (d, *J* = 15.6 Hz, 1H), 4.65-4.60 (m, 1H), 3.28 (dd, *J* = 13.3, 5.5 Hz, 1H), 2.79 (dd, *J* = 13.3, 8.3 Hz, 1H), 1.43-1.08 (m, 18H);

^{13}C NMR (100 MHz, $CDCl_3$): δ 190.4, 149.0, 137.6, 137.5, 132.5, 129.7, 128.4, 128.3, 128.1, 126.1, 125.8, 84.9, 60.2, 59.5, 40.9, 40.1, 34.7, 34.0, 26.8, 20.4, 20.2, 17.1

Synthesis the (*E*)-4-cyclopropyl-1-phenylbut-2-en-1-one (13)

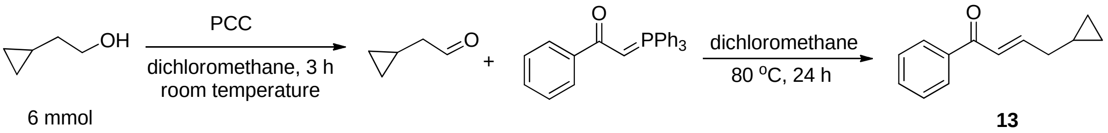

(1) To a well-stirred solution of 2-cyclopropylethanol (0.5168g, 6.0 mmol) in dry dichloromethane (20.0 mL) in a 25 mL reaction tube was added Celite (Celite : alcohol = 1:1, wt/wt) and PCC (pyridinium chlorochromate, 3.2334 g, 2.5 equiv) , and then the reaction mixture was stirred at room temperature for 3h. After the reaction was finished, the mixture was filtered through a pad of silica gel and washed with 30.0 mL of dichloromethane. The collected organic layer was directly used in the next step reaction without further purification.

(2) The compound 2-(triphenylphosphoranylidene)acetophenone (2.2826g, 6.0 mmol) was added to the solution of collected organic layer in CH_2Cl_2 at 80 °C for 30 h. After the reaction was finished, the reaction mixture was concentrated under reduced

pressure and purified by flash chromatography on silica gel (petroleum ether/diethyl ether = 100 : 2), the product Synthesis of (*E*)-4-cyclopropyl-1-phenylbut-2-en-1-one **13** was obtained in 51% overall yield.

Physical state: yellow oil;

HRMS (*m/z*): calculated for $C_{13}H_{14}OH^+$ $[M+H]^+$, 187.1117; found, 187.1116;

^{1}H NMR (400 MHz, $CDCl_3$): 7.95-7.93 (m, 2H), 7.57-7.53 (m, 1H), 7.48-7.45 (m, 2H), 7.16-7.09 (m, 1H), 7.00-6.95 (m. 1H), 2.23-2.20 (m, 2H), 0.92-0.82 (m, 1H), 0.58-0.53, (m, 2H), 0.18-0.14 (m, 2H);

^{13}C NMR (100 MHz, $CDCl_3$): 191.0, 148.9, 137.9, 132.5, 128.5, 128.4, 125.7, 37.4, 9.1, 4.4.

Observation of the Successive Dehydrogenation Sequence

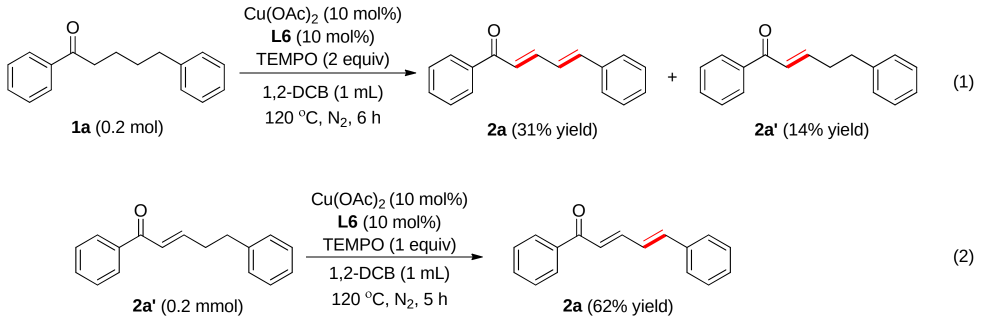

In the Supplementary Equation 1, the reaction was conducted with **1a** (0.0477g, 0.2 mmol), $Cu(OAc)_2$ (0.0036g, 0.02 mmol, 10 mol%), 1,10-phenanthroline (0.0036g, 0.02 mmol, 10 mol%) and TEMPO (0.0630g, 0.4 mmol) in1,2-dichlorobenzene (1.0 mL) at 120 °C for 6 h. Then the reaction was analyzed by GC using dodecane as an internal standard. GC yields (**2a and 2a'**) were reported in the Supplementary Equation 1.

In the Supplementary Equation 2, the reaction was conducted with **2a’** (0.0473, 0.2 mmol), $Cu(OAc)_2$ (0.0036g, 0.02 mmol, 10 mol%), 1,10-phenanthroline (0.0036g, 0.02 mmol, 10 mol%) and TEMPO (0.0315g, 0.2 mmol) in1,2-dichlorobenzene (1.0 mL) at 120 °C for 5 h. Then the reaction was analyzed by GC using dodecane as an internal standard. GC yield of **2a** was reported in the Supplementary Equation 2.

Identification of γ-TEMPO-substituted Enone Intermediate by NMR

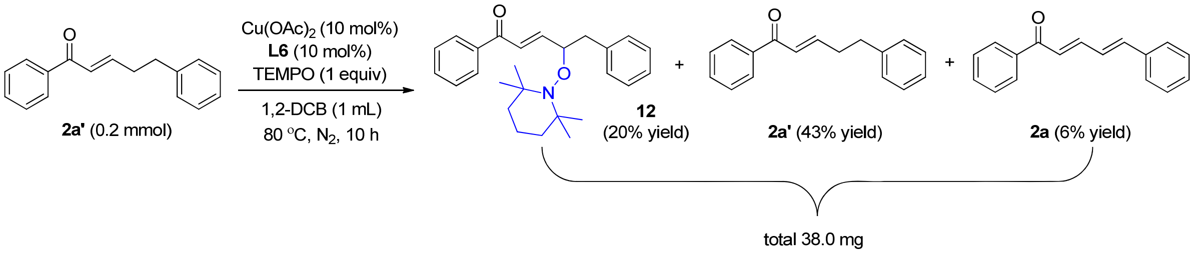

(3)

In order to directly observe the formation of γ-TEMPO-substituted enone intermediate **12**, the reaction was conducted with **2a’** (0.0473g, 0.2 mmol), $Cu(OAc)_2$ (0.0036g, 0.02 mmol, 10 mol%), 1,10-phenanthroline (0.0036g, 0.02 mmol, 10 mol%) and TEMPO (0.0315g, 0.2 mmol) in 1,2-dichlorobenzene (1.0 mL) at 80 °C for 10 h (At higher temperature the β-TEMPO elimination is fast, which will make it difficult to observe the formation **12**). After cooling to room temperature, the reaction was concentrated and purified by flash chromatography on silica gel (petroleum ether/ethyl ether = 100:5) to obtained 38.0 mg of isolated product. After ^{1}H NMR analysis, the isolated product was identified as a mixture of **2a’**, **12** and **2a** (inseparable by column chromatography). The ^{1}H NMR spectra of this mixture is illustrated in Supplementary Figure 162D (red line). The ^{1}H NMR spectra of independently prepared **2a’** (Supplementary Figure 162A, purple line), **2a** (Supplementary Figure 162B, blue line) and **12** (Supplementary Figure162C, green line) was also illustrated in Supplementary Figure 162. For a clear and convenient comparison, the enlarged spectra at different range are illustrated in Supplementary Figure 163 and Supplementary Figure 164. The yields of **12**, **2a** and **2a’** were determined by the analysis of relative ratio (mol/mol) in ^{1}H NMR spectra (as shown in Supplementary Figure 165).

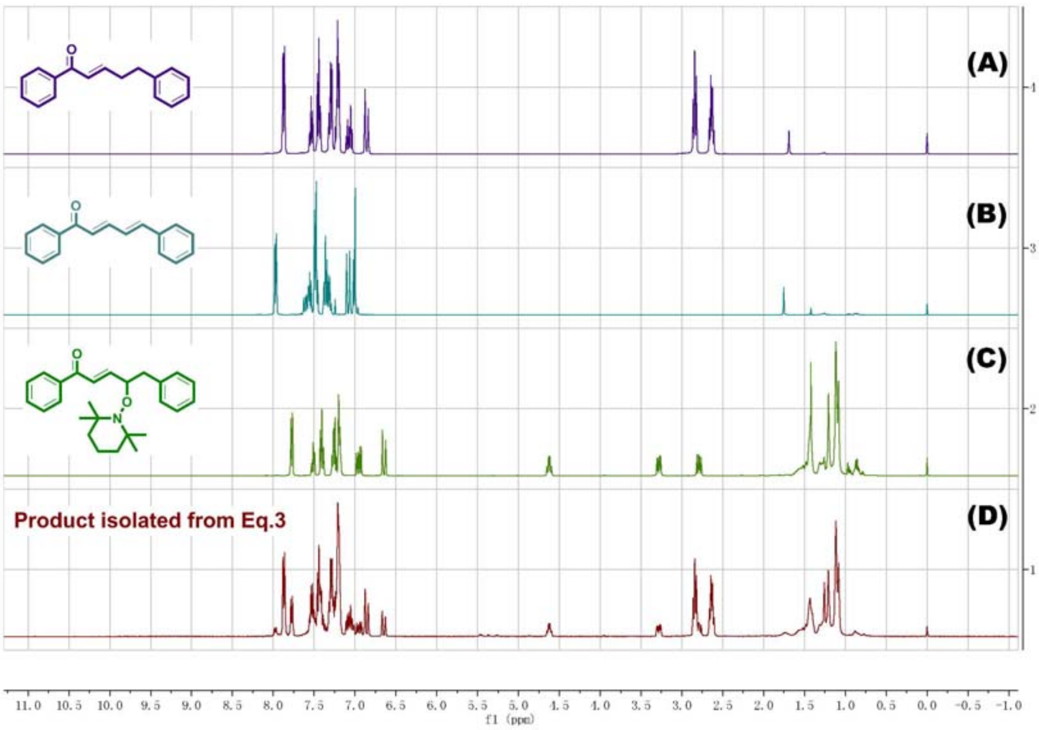

Supplementary Figure 162. (A) ^{1}H NMR spectra of 2a'; (B) ^{1}H NMR spectra of 2a; (C) ^{1}H NMR spectra of 12; (D) ^{1}H NMR spectra of isolated product from Eq. 3.

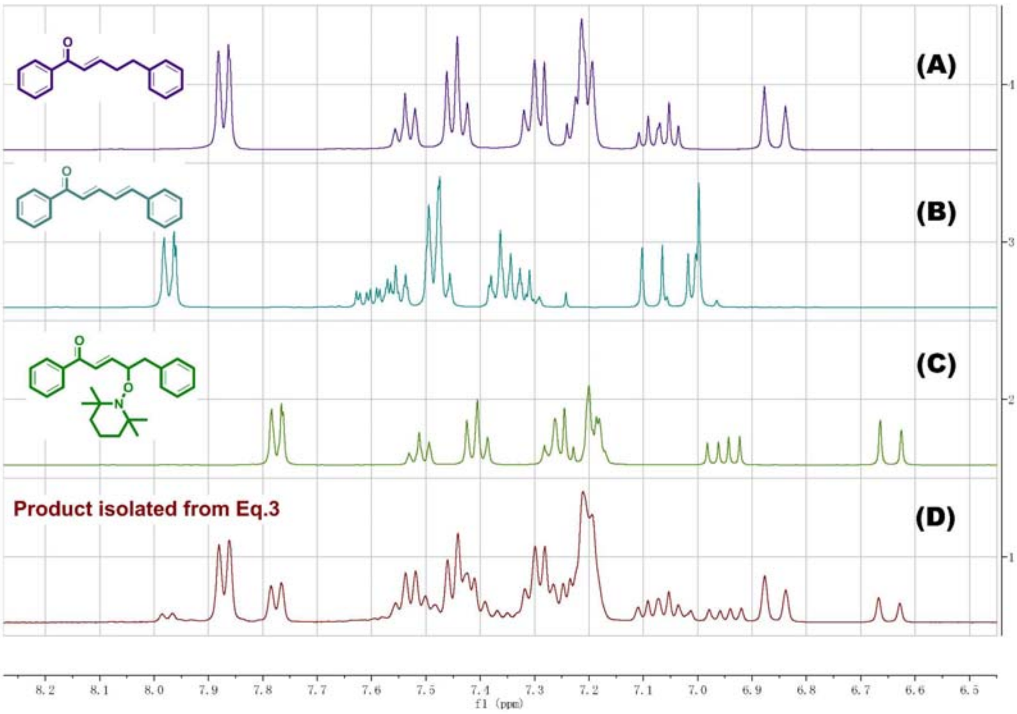

Supplementary Figure 163. Enlarged ^{1}H NMR spectra in Fig. S1 in the range between 6.45ppm-8.25 ppm

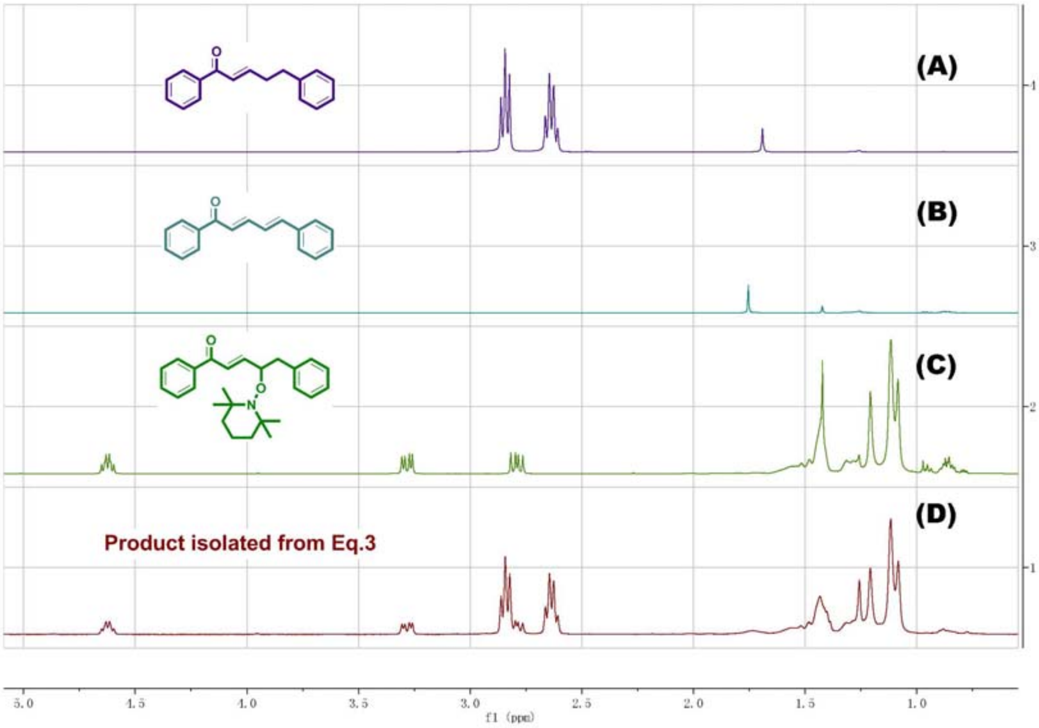

Supplementary Figure 164. Enlarged ^{1}H NMR spectra in Fig. S1 in the range between 0.00ppm-5.00 ppm

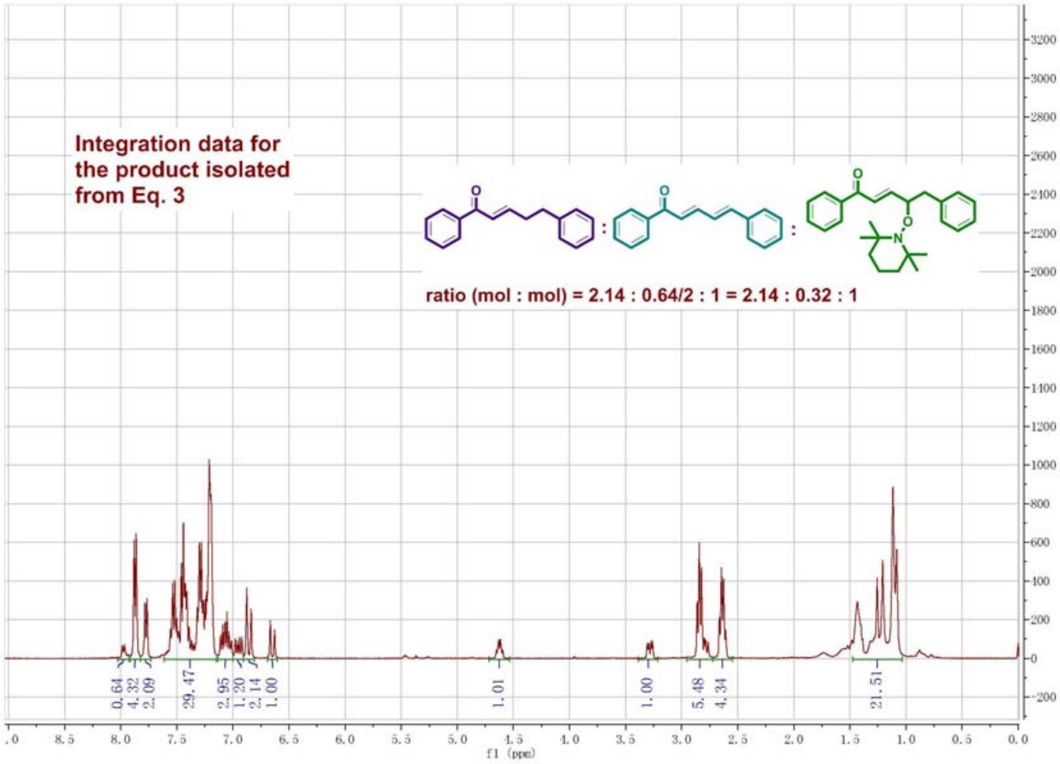

Supplementary Figure 165. Integration data for the product isolated from Eq. 3.

The Evidence of Successive Dehydrogenation Pathway via γ-TEMPO Substituted Enone Intermediate

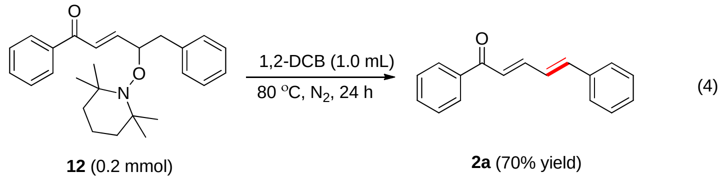

In order to provide the successive dehydrogenation through β-eliminatin of the γ-TEMPO-substituted enone intermediate **12**, the reaction was conducted with **12** (0.0783g, 0.2 mmol) in1, 2-dichlorobenzene (1.0 mL) at 80 °C for 24 h. After cooling to room temperature, the reaction mixture was concentrated and purified by flash chromatography on silica gel (petroleum ether/ethyl ether = 100:5). The product **2a** was obtained in 70% yield.

The Radical Probe Experiment to Identify the γ-enone Radical Intermediate

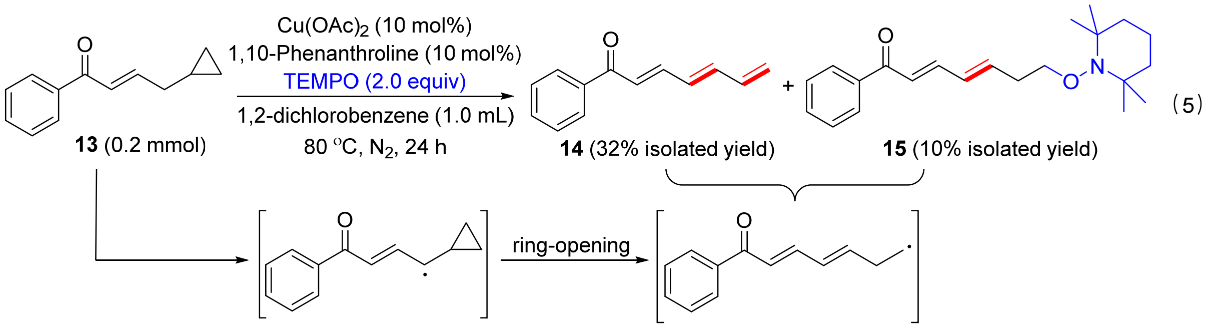

In order to identify the successive dehydrogenation through the γ-enone radical intermediate, the radical clock reaction was conducted with **13** (0.0372g, 0.2 mmol) 1,10-phenanthroline (0.0036g, 0.02 mmol, 10 mol%) and TEMPO (0.0630g, 0.4 mmol) in1, 2-dichlorobenzene (1.0 mL) at 80 °C for 24 h. After cooling to room temperature, the reaction mixture was concentrated and purified by flash chromatography on silica gel (petroleum ether/ethyl ether = 100:5). The trienone product **14** was obtained in 32% yield (This compound is known.[11]) and the terminal TEMPO substituent product **15** was obtained in 10% yield.

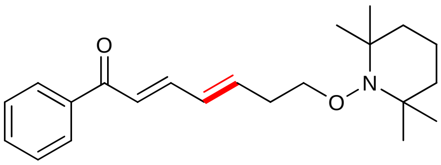

15

Physical state: slight yellow oil;

HRMS (*m/z*): calculated for $C_{22}H_{31}NO_2H^+$ $[M+H]^+$, 342.2428; found, 342.2429;

^{1}H NMR (400 MHz, $CDCl_3$): δ 7.95-7.93 (m, 2H), 7.57-7.54 (m, 1H), 7.49-7.39 (m, 3H), 6.90 (d, J = 15.1 Hz, 1H), 6.42-6.28 (m, 2H), 3.84 (t, J = 6.6 Hz, 2H), 2.46-2.42 (m, 2H), 1.45-1.43 (m, 4H), 1.33-1.30 (m, 2H), 1.15 (s, 6H), 1.09 (s, 6H);

^{13}C NMR (100 MHz, $CDCl_3$): δ 190.9, 145.2, 143.0, 138.3, 132.5, 130.5, 128.5, 128.4, 123.9, 75.2, 59.8, 39.6, 33.1, 32.7, 29.7, 20.1, 17.1.

Kinetic Time Course Experiments of the Successive Dehydrogenation of 1a

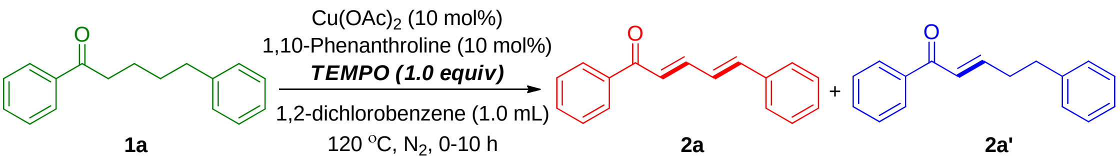

(6)

Supplementary Table 3. The Time Course Data for the Successive Dehydrogenation Reaction of 1a

Time (h)	**[2a]**	**[2a']**	**[1a]**
0	0	0	100
1	0	4	80
2	0	4	78
4	11	5	66
6	14	3	65
10	23	6	56

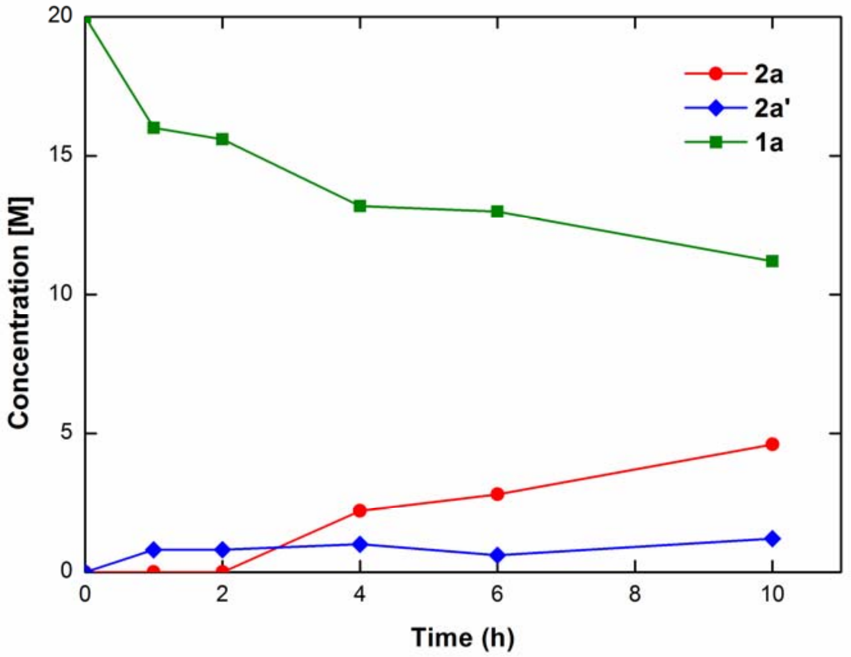

Supplementary Figure 166. Kinetic time course of the dehydrogenation of **1a** (green) to **2a** (red) and **2a'** (blue) using lower amount of TEMPO.

In the Supplementary Equation 6, the time course of the successive dehydrogenation reactions of **1a** were conducted with **1a** (0.0477g, 0.2 mmol), $Cu(OAc)_2$ (0.0036g, 0.02 mmol, 10 mol%), 1,10-phenanthroline (0.0036g, 0.02 mmol, 10 mol%) and TEMPO (0.0315g, 0.2 mmol) in1,2-dichlorobenzene (1.0 mL) at 120 ºC for specific time. Then the reaction's GC yield (**2a ,2a' and 1a'**) was analyzed by GC using dodecane as an internal standard.

Observation of 2,2,6,6-Tetramethylpiperidine in the Successive Dehydrogenation of 1a

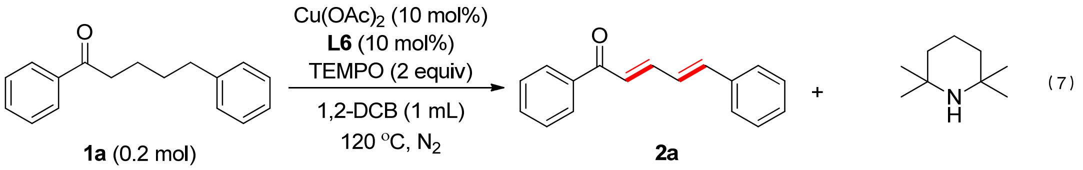

	2a	
24 h	50%	56%
48 h	85%	79%

In the Supplementary Equation 7, the successive dehydrogenation reactions was conducted with **1a** (0.0477g, 0.2 mmol), $Cu(OAc)_2$ (0.0036g, 0.02 mmol, 10 mol%), 1,10-phenanthroline (0.0036g, 0.02 mmol, 10 mol%) and TEMPO (0.0630g, 0.4 mmol) in1,2-dichlorobenzene (1.0 mL) at 120 °C for specific time. After the reaction was finished, the 2,2,6,6-tetramethylpiperidine was analyzed by GC using dodecane as an internal standard.

Competition Reaction Between *N*-heterocycle and alcohol

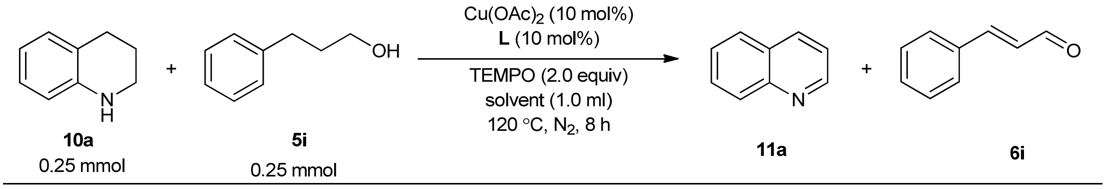

(8)

L1/TsOH(10 mol%)/1,2-dichlorobenzene	31%	15%
L2/TsOH(10 mol%)/1,2-dichlorobenzene	24%	19%
L1/LiOAc(1.0 equiv)/1,2-dichlorobenzene	30%	7%
L2/LiOAc(1.0 equiv)/1,2-dichlorobenzene	27%	17%
L1/TsOH(10 mol%)/*t*-AmylOH	30%	19%
L2/TsOH(10 mol%)/*t*-AmylOH	27%	20%
L1/LiOAc(1.0 equiv)/ *t*-AmylOH	33%	<5%
L2/LiOAc(1.0 equiv)/ *t*-AmylOH	31%	11%

Kinetic Dependence of Successive Dehydrogenative Reaction Components by Initial Rate Methods Using (*E*)-4-cyclopropyl-1-phenylbut-2-en-1-one (1a’) as Model Substrate

General Methods: The reactions were conducted for specific times and then immediately quenched by immersing vessels into ice-cold water. When the reaction tube was cool down, the dodecane (0.0170g, 0.1mmol) as an internal standard was added to the reaction mixture and then the mixture was analyzed by GC.

Kinetic Dependence on $Cu(OAc)_2$/1,10-phenanthroline: Reactions were performed with **1a’** (0.0237 g, 0.1 mmol), $Cu(OAc)_2$ (0.0009-0.0144 g, 0.005-0.08 mmol), 1,10-phenanthroline (0.0009-0.0144 g, 0.005-0.08 mmol) and TEMPO (0.0156 g, 0.1 mmol) in 1,2-dichlorobenzene (0.5 mL). The mixture was kept stirring (at 270 rpm) at 120 ° C using an aluminium-heating block. After 10-60 minutes, the mixture was added with dodecane (22 μL) as an internal standard using a microliter syringe and then analyzed by GC.

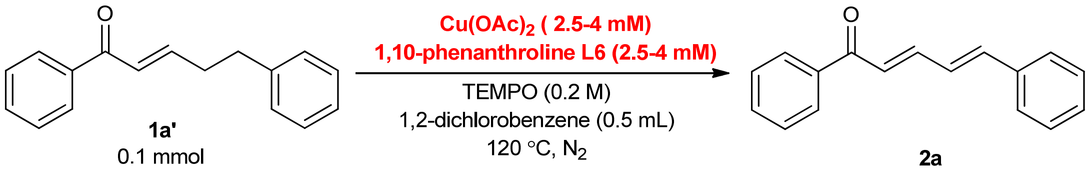

[$Cu(OAc)_2$/1,10-Phen] (mM)	time (min)	[2a] (M)	initial rate (M/min)
0.01	15	0.01786	0.00108
	30	0.02652	
	45	0.05377	
	60	0.06275	

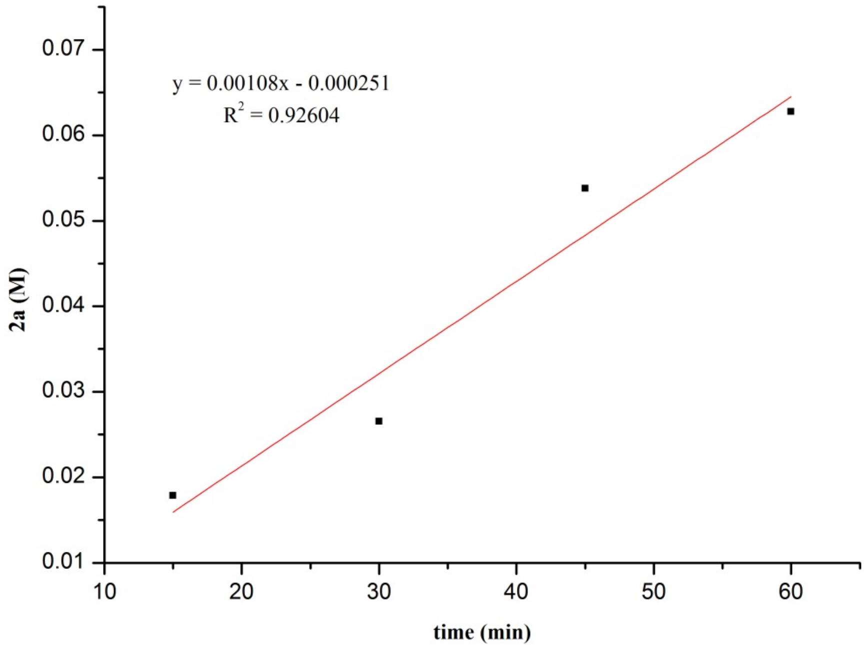

Supplementary Figure 167. Initial rate date for the successive dehydrogenation of **1a'** to **2a** at [Cu(OAc)$_2$/1,10-phen] (0.01 M).

[Cu(OAc)$_2$/1,10-Phen] (M)	time (min)	[2a] (M)	initial rate (M/min)
0.02	10	0.02468	0.00109
	20	0.03572	
	30	0.04374	
	40	0.05839	

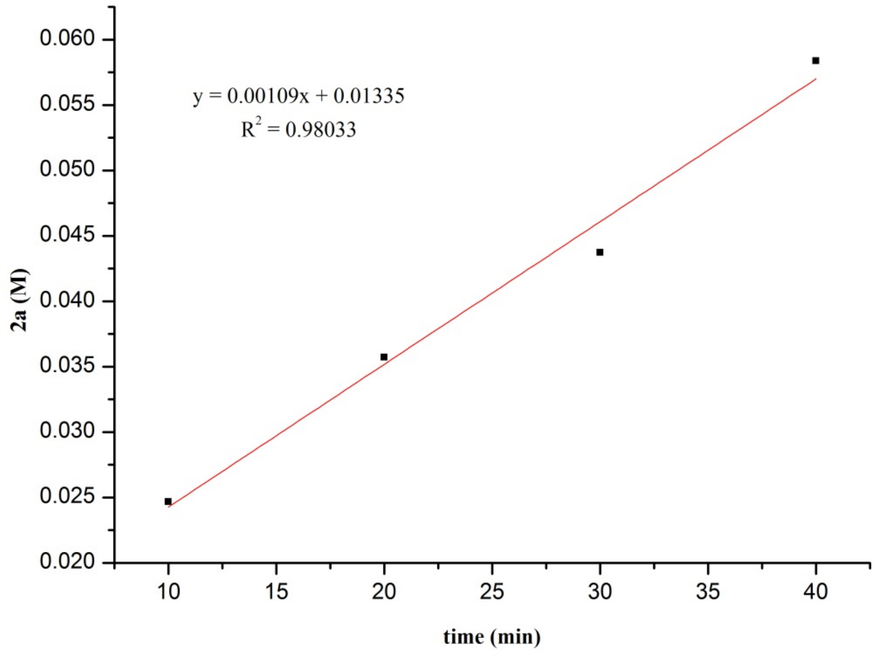

Supplementary Figure 168. Initial rate date for the successive dehydrogenation of **1a'** to **2a** at [Cu(OAc)$_2$/1,10-phen] (0.02 M).

[Cu(OAc)$_2$/1,10-Phen] (mM)	time (min)	[2a] (M)	initial rate (M/min)
0.04	10	0.03426	0.00133
	20	0.05493	
	30	0.06118	
	40	0.07666	

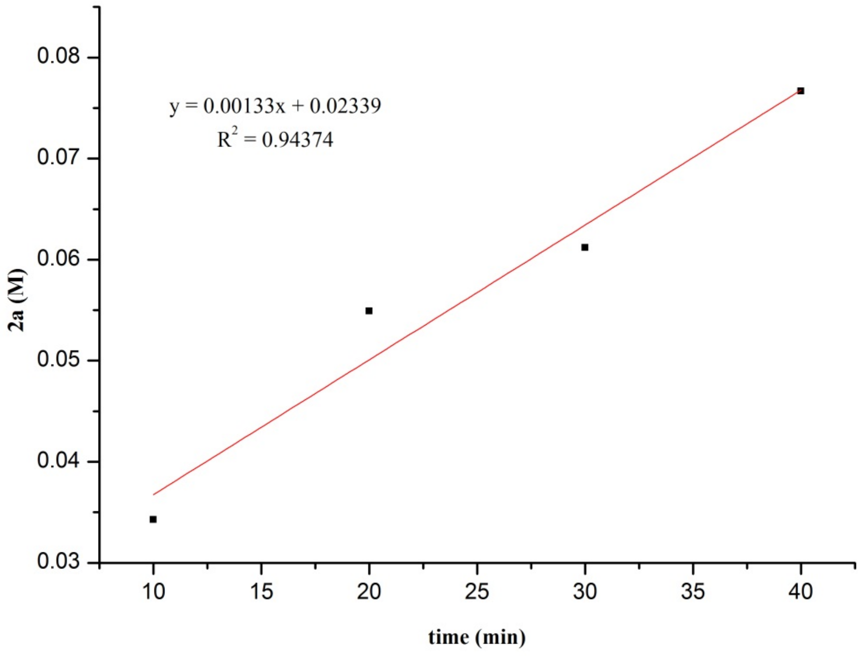

Supplementary Figure 169. Initial rate date for the successive dehydrogenation of **1a'** to **2a** at [Cu(OAc)$_2$/1,10-phen] (0.04 M).

[Cu(OAc)$_2$/1,10-Phen] (mM)	time (min)	[2a] (M)	initial rate (M/min)
0.08	10	0.04502	0.00140
	20	0.06488	
	30	0.07696	
	40	0.08778	

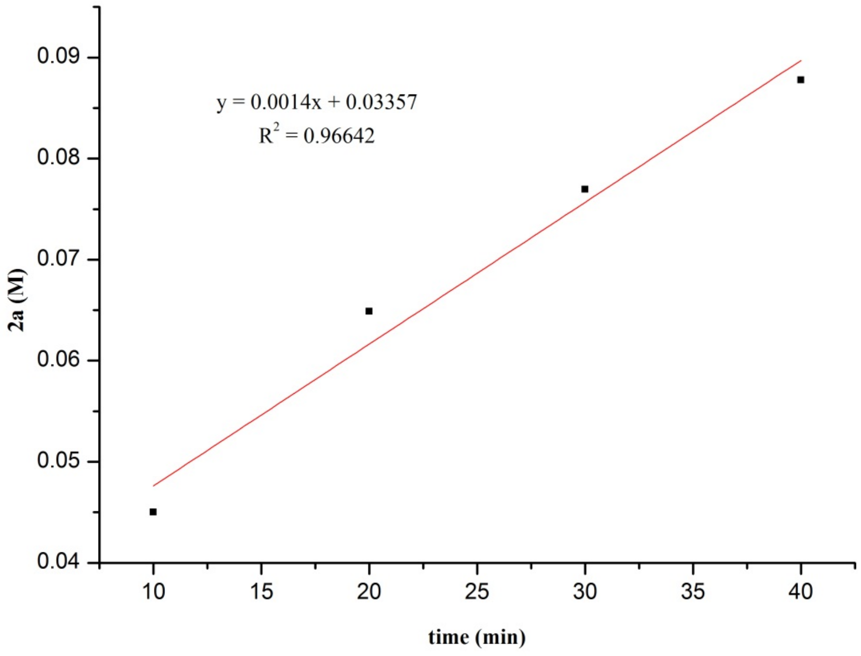

Supplementary Figure 170. Initial rate date for the successive dehydrogenation of **1a'** to **2a** at [Cu(OAc)2/1,10-phen] (0.08 M).

[Cu(OAc)2/1,10-Phen] (mM)	time (min)	[2a] (M)	initial rate (M/min)
0.16	10	0.04962	0.00188
	20	0.07258	
	30	0.09153	
	40	0.10598	

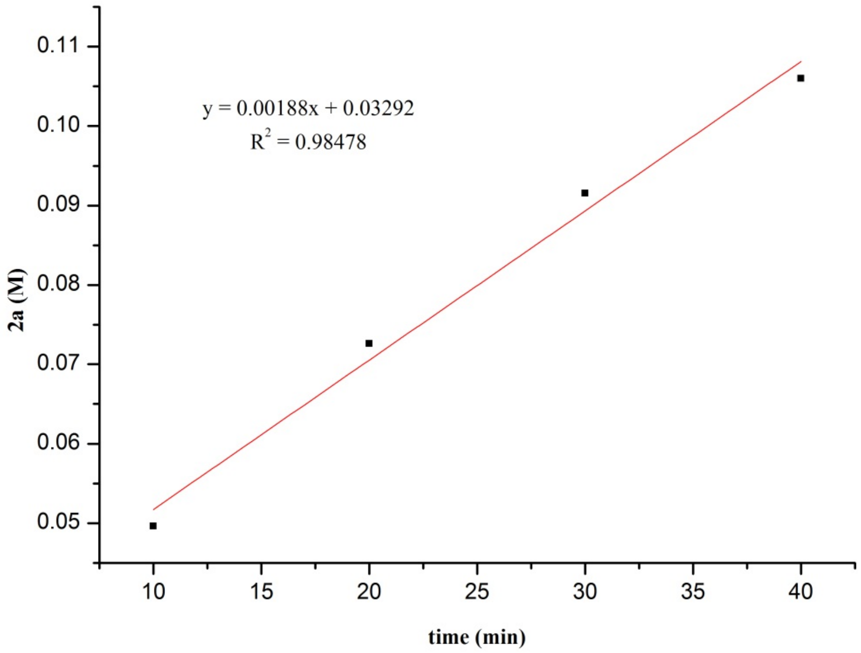

Supplementary Figure 171. Initial rate date for the successive dehydrogenation of **1a'** to **2a** at [Cu(OAc)$_2$/1,10-phen] (0.16 M).

[Cu(OAc)$_2$/1,10-phen] (mM)	Initial rate (M/min)
0.01	0.00108
0.02	0.00109
0.04	0.00133
0.08	0.00140
0.16	0.00188

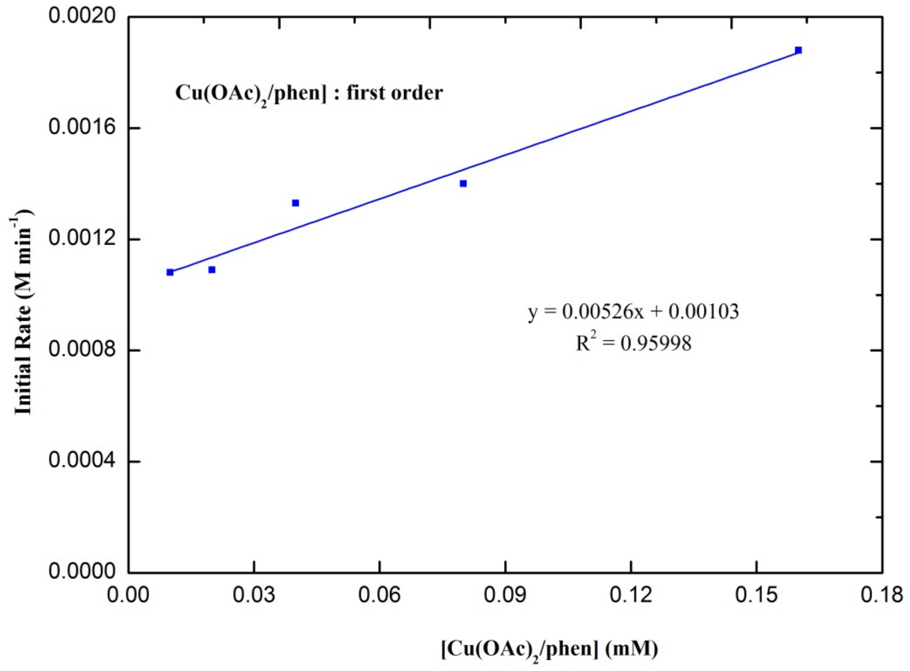

Supplementary Figure 172. Kinetic data from the successive dehydrogenation of **1a'** at varying concentrations of [Cu(OAc)$_2$/1,10-phen].

Kinetic Dependence on 1a': Reactions were performed with **1a'** (0.0071-0.0356 g, 0.03-0.15 mmol), Cu(OAc)$_2$ (0.0018 g, 0.01 mmol), 1,10-phenanthroline (0.0018 g, 0.01 mmol) and TEMPO (0.0156 g, 0.1 mmol) in 1,2-dichlorobenzene (0.5 mL). The mixture was kept stirring (at 270 rpm) at 120 º C using an aluminium-heating block. After 6-30 minutes, the mixture was added with dodecane (22 µL) as an internal standard using a microliter syringe and then analyzed by GC.

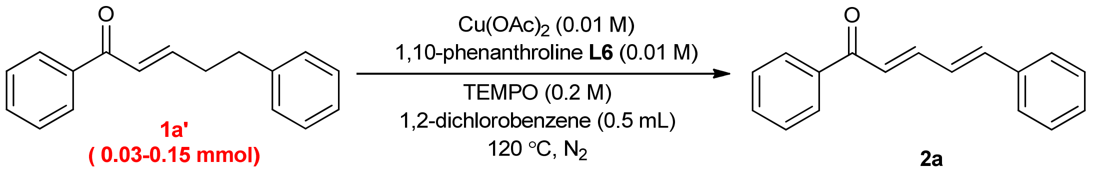

[1a'] (M)	time (min)	[2a] (M)	initial rate (M/min)
0.06	12	0.01543	0.0002541
	18	0.01759	
	24	0.01874	

	30	0.02013	

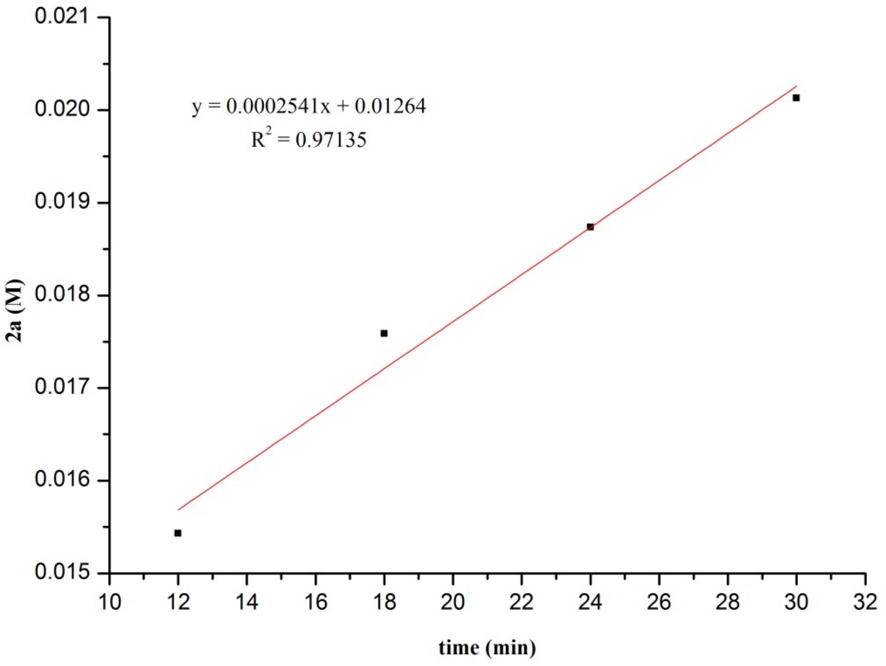

Supplementary Figure 173. Initial rate date for the successive dehydrogenation of 1a' to 2a at [1a'] (0.06 M).

[1a'] (M)	time (min)	[2a] (M)	initial rate (M/min)
0.1	6	0.00761	0.000643733
	12	0.00917	
	18	0.01342	
	24	0.01907	

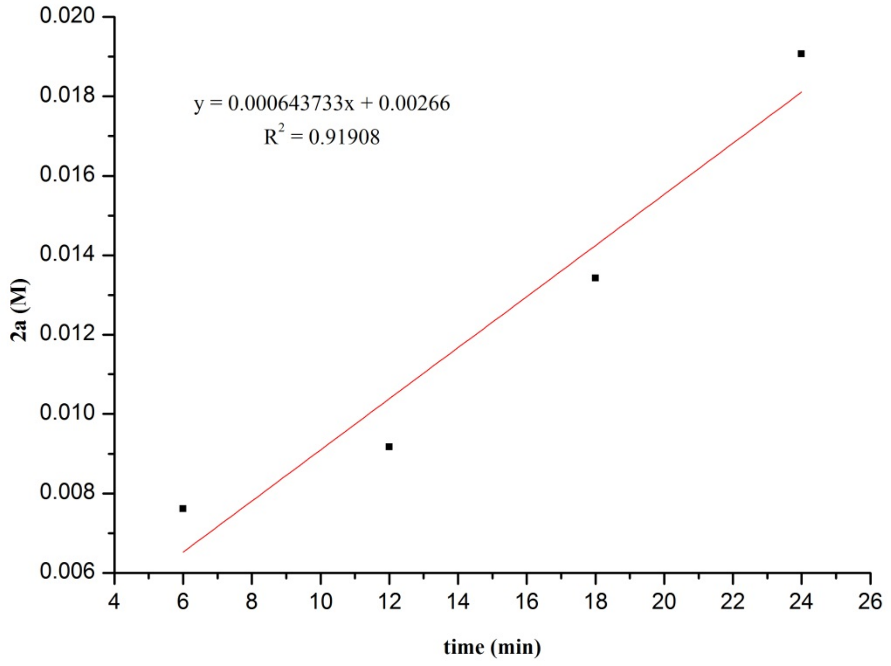

Supplementary Figure 174. Initial rate date for the successive dehydrogenation of 1a' to 2a at [1a'] (0.1 M).

[1a'] (M)	time (min)	[2a] (M)	initial rate (M/min)
0.15	6	0.02921	0.00119
	12	0.03405	
	18	0.04159	
	24	0.05041	

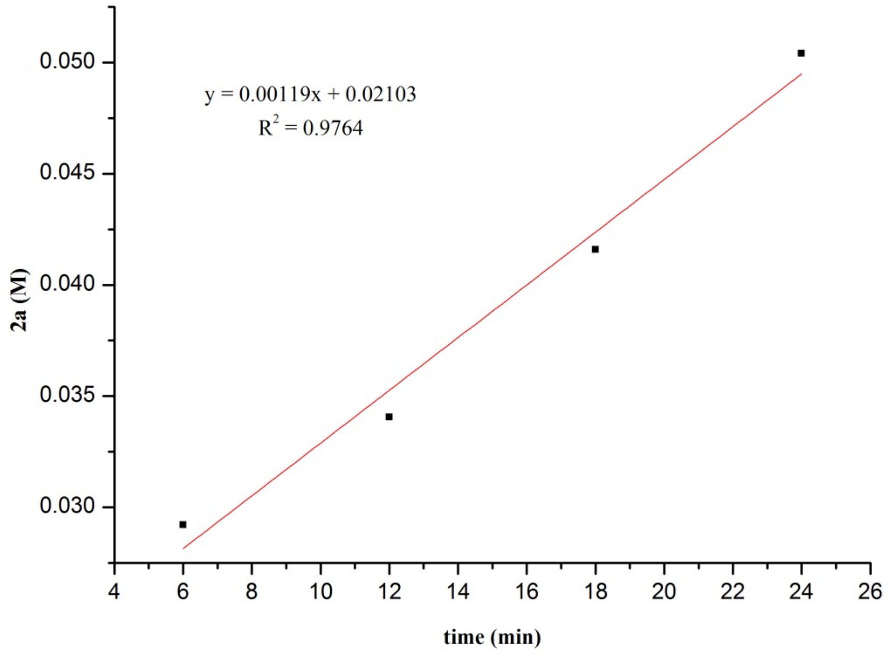

Supplementary Figure 175. Initial rate date for the successive dehydrogenation of 1a' to 2a at [1a'] (0.15 M).

[1a'] (M)	time (min)	[2a] (M)	initial rate (M/min)
0.2	6	0.02223	0.00172
	12	0.03212	
	18	0.04394	
	24	0.05279	

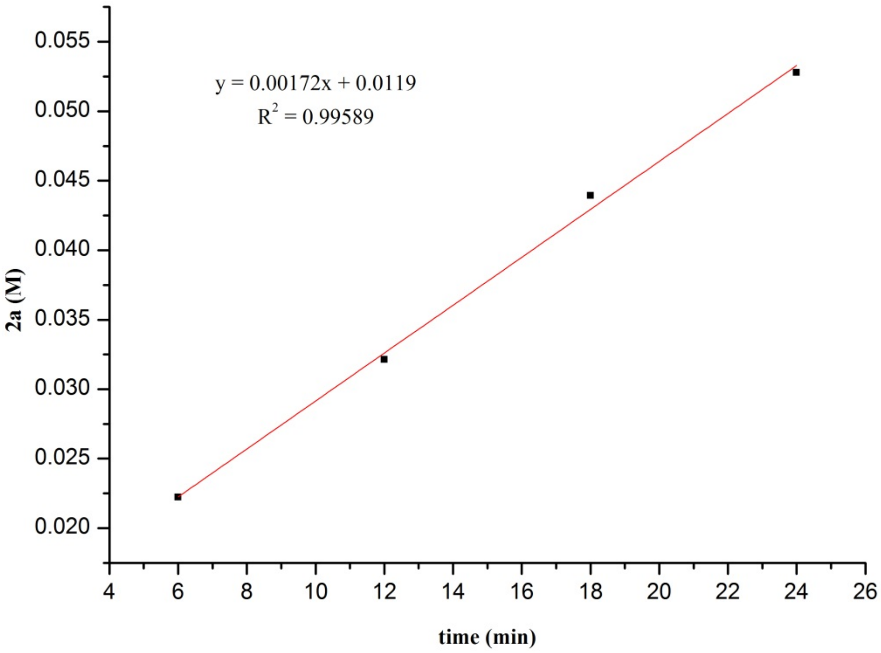

Supplementary Figure 176. Initial rate date for the successive dehydrogenation of 1a’ to 2a at [1a’] (0.2 M).

[1a’] (M)	time (min)	[2a] (M)	initial rate (M/min)
0.3	6	0.02130	0.0024
	12	0.03920	
	18	0.05377	
	24	0.0645	

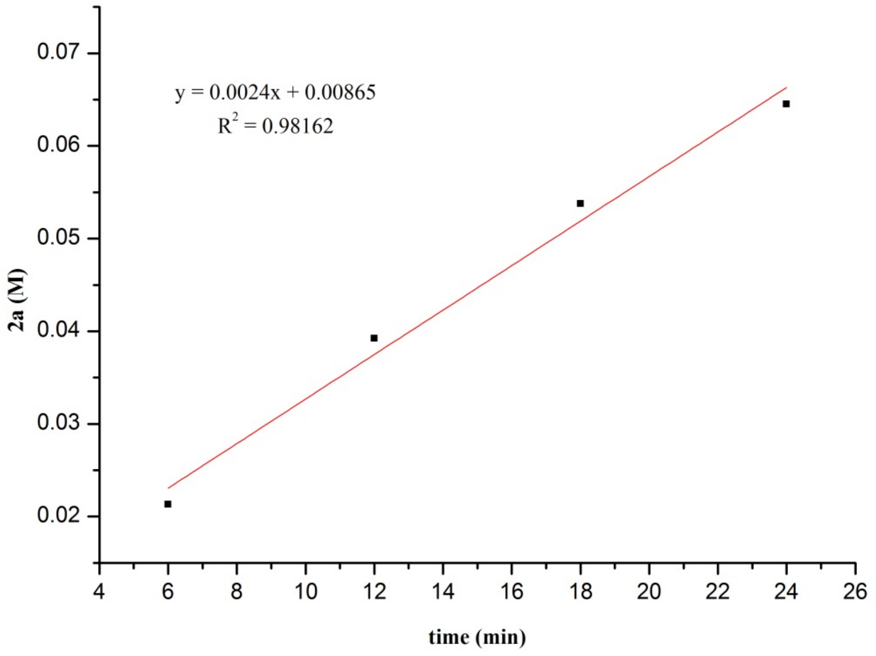

Supplementary Figure 177. Initial rate date for the successive dehydrogenation of 1a’ to 2a at [1a’] (0.3 M).

[1a’] (M)	initial rate (M/min)
0.06	0.0002541
0.1	0.0006437
0.15	0.00119
0.2	0.00172
0.3	0.00240

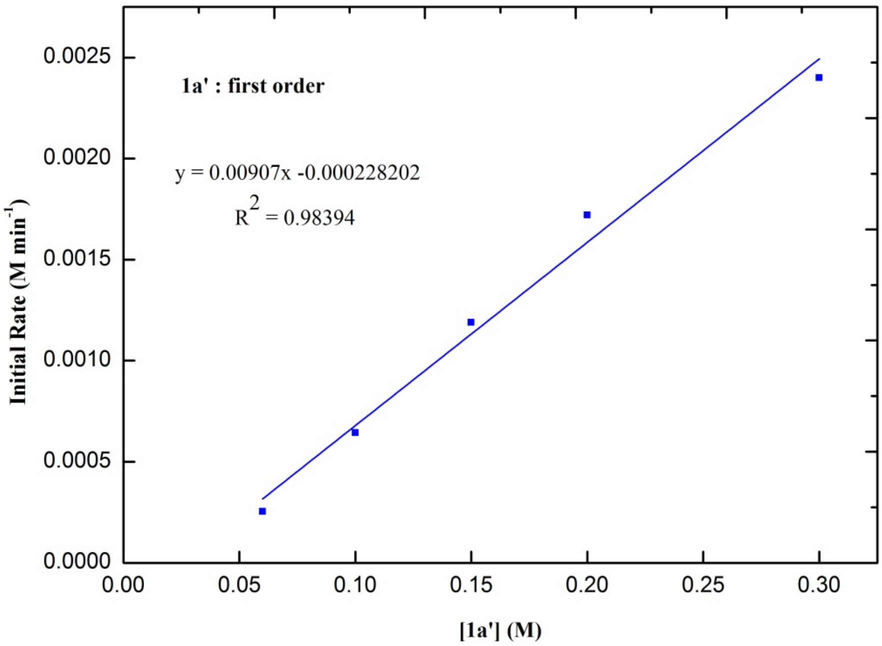

Supplementary Figure 178. Kinetic data from the successive dehydrogenation of **1a’** at varying concentrations of [1a’].

Kinetic Dependence on TEMPO: Reactions were performed with 1a’ (0.0237 g, 0.1 mmol), $Cu(OAc)_2$ (0.0018 g, 0.01 mmol), 1,10-phenanthroline (0.0018 g, 0.01 mmol) and TEMPO (0.0031-0.0624 g, 0.02-0.4 mmol) in 1,2-dichlorobenzene (0.5 mL). The mixture was kept stirring (at 270 rpm) at 120 °C using an aluminium-heating block. After 8-32 minutes, the mixture was added with dodecane (22 μL) as an internal standard using a microliter syringe and then analyzed by GC.

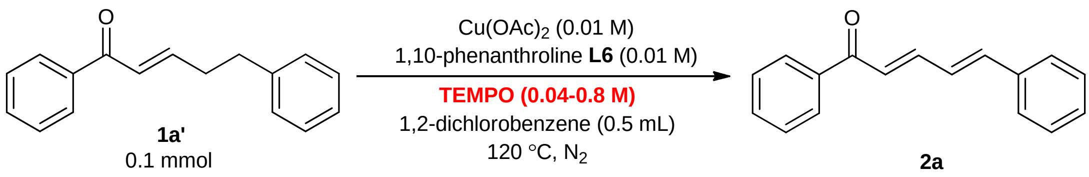

[TEMPO] (M)	time (min)	[2a] (M)	initial rate (M/min)
0.04	8	0.01625	0.000362025
	16	0.01945	
	24	0.02188	
	32	0.02509	

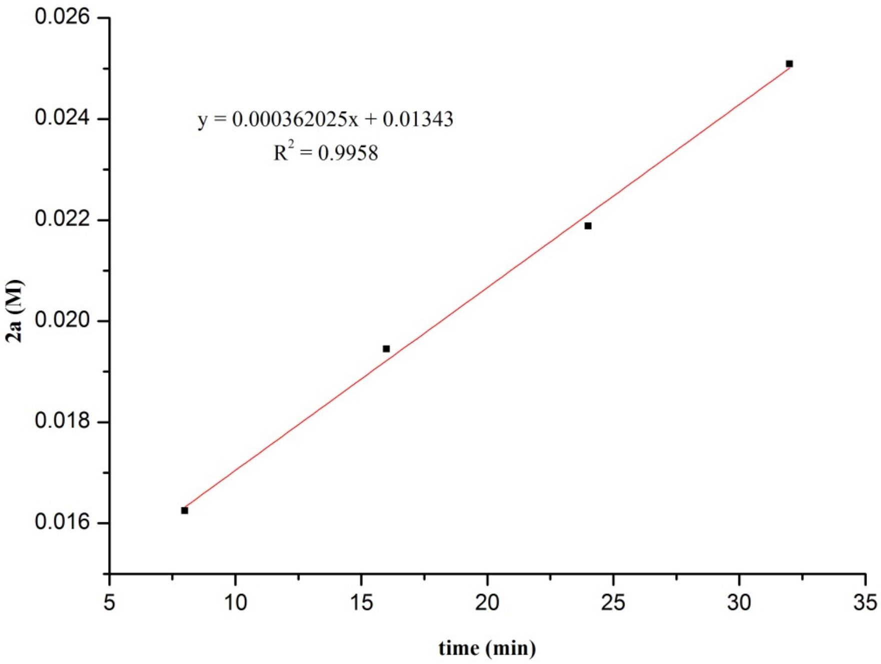

Supplementary Figure 179. Initial rate date for the successive dehydrogenation of 1a' to 2a at [TEMPO] (0.04 M).

[TEMPO] (M)	time (min)	[2a] (M)	initial rate (M/min)
0.08	8	0.01868	0.0007759
	16	0.02766	
	24	0.03233	
	32	0.03782	

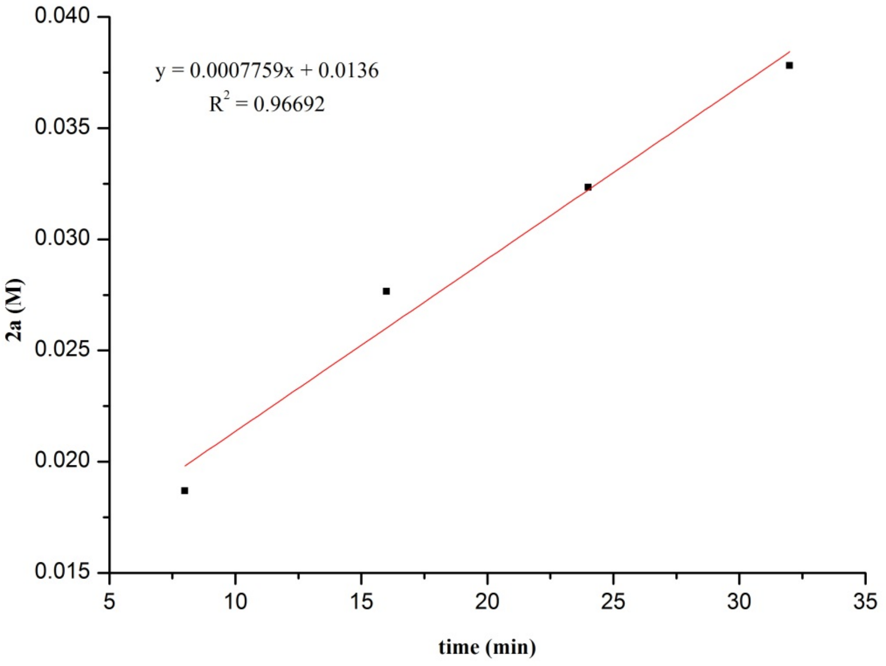

Supplementary Figure 180. Initial rate date for the successive dehydrogenation of 1a' to 2a at [TEMPO] (0.08 M).

[TEMPO] (M)	time (min)	[2a] (M)	initial rate (M/min)
0.12	8	0.02642	0.00152
	16	0.03260	
	24	0.05224	
	32	0.06047	

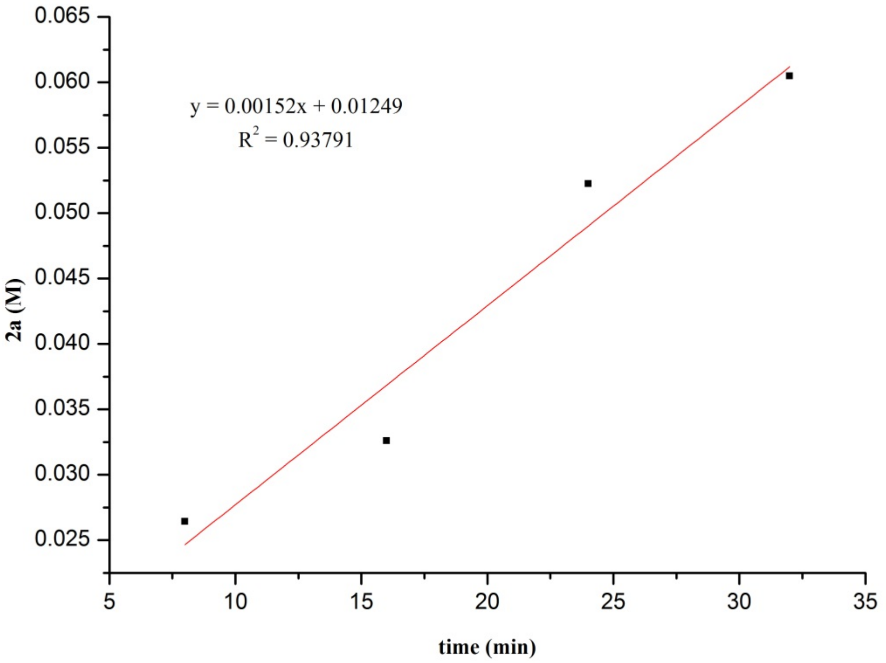

Supplementary Figure 181. Initial rate date for the successive dehydrogenation of 1a' to 2a at [TEMPO] (0.12 M).

[TEMPO] (M)	time (min)	[2a] (M)	initial rate (M/min)
0.2	8	0.02447	0.00193
	16	0.04207	
	24	0.05224	
	32	0.07247	

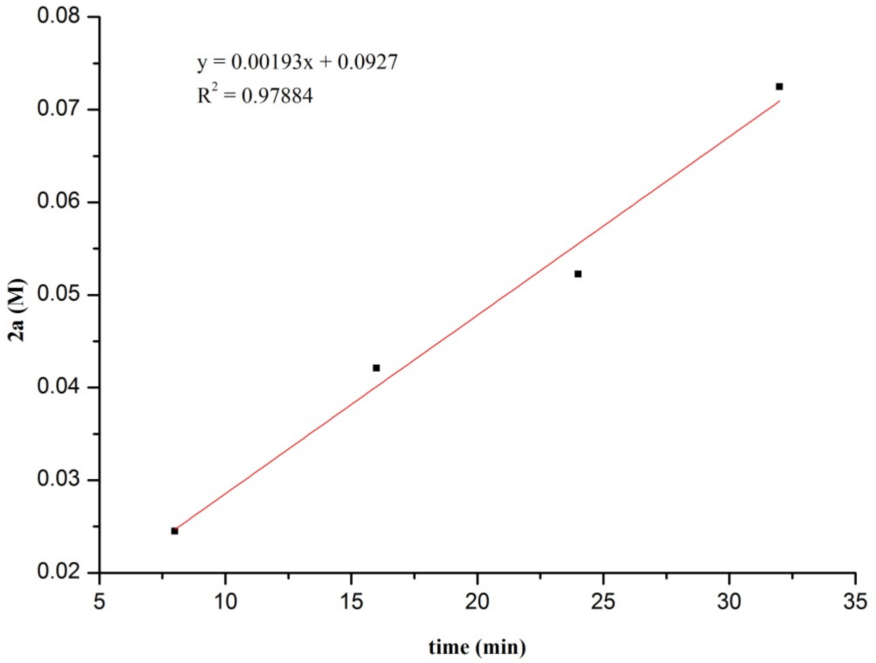

Supplementary Figure 182. Initial rate date for the successive dehydrogenation of 1a' to 2a at [TEMPO] (0.2 M).

[TEMPO] (M)	time (min)	[2a] (M)	initial rate (M/min)
0.4	8	0.02274	0.00165
	16	0.03013	
	24	0.04919	

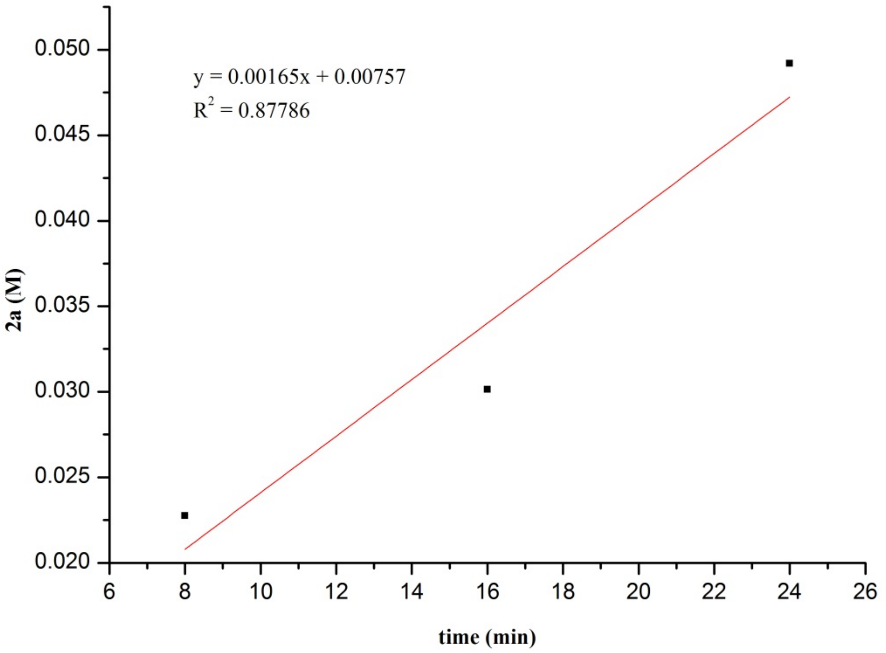

Supplementary Figure 183. Initial rate date for the successive dehydrogenation of 1a' to 2a at [TEMPO] (0.4 M).

[TEMPO] (M)	time (min)	[2a] (M)	initial rate (M/min)
0.6	8	0.02069	0.00155
	16	0.03703	
	24	0.04392	
	32	0.05966	

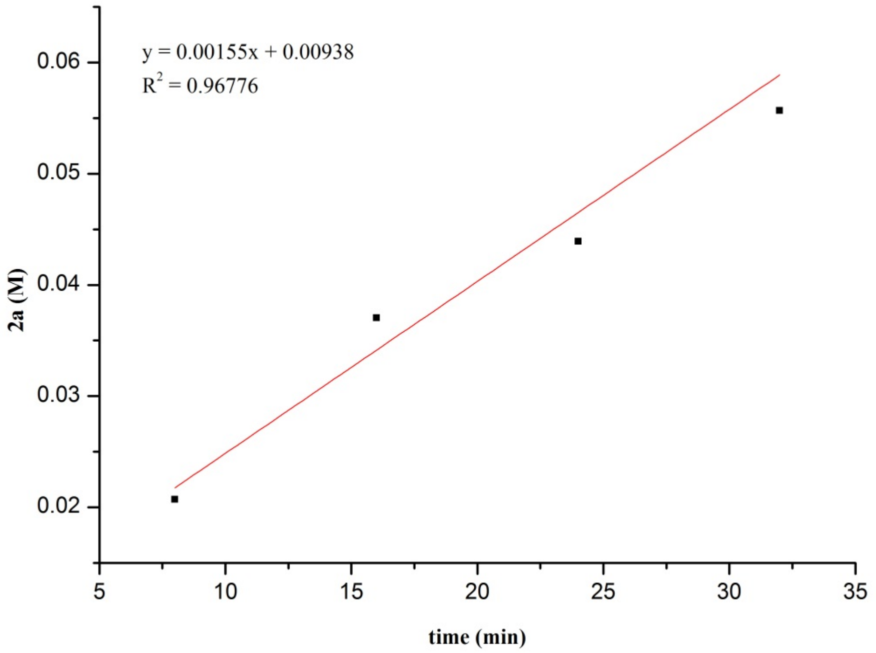

Supplementary Figure 184. Initial rate date for the successive dehydrogenation of 1a' to 2a at [TEMPO] (0.6 M).

[TEMPO] (M)	time (min)	[2a] (M)	initial rate (M/min)
0.8	8	0.01741	0.00195
	16	0.03612	
	24	0.04855	

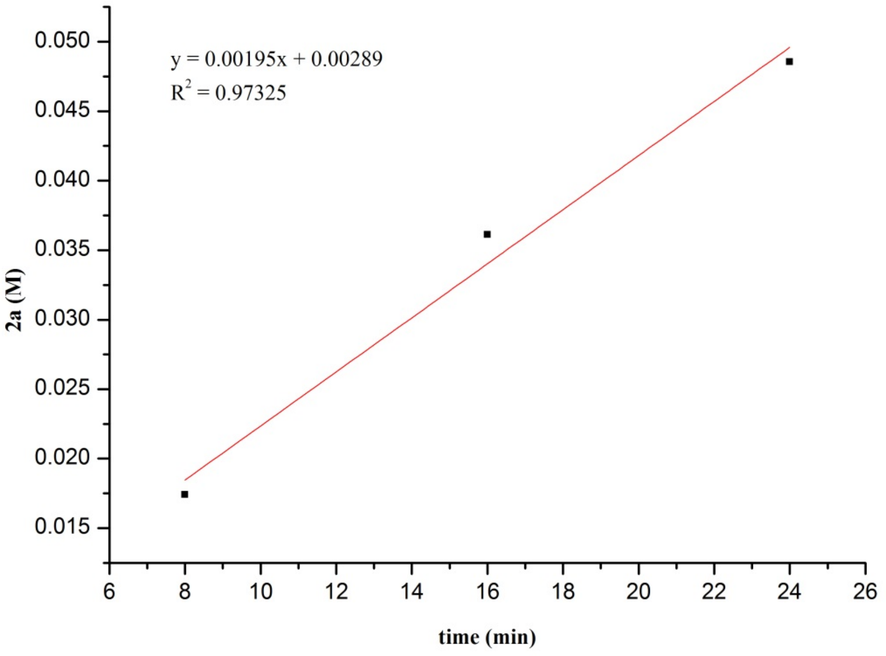

Supplementary Figure 185. Initial rate date for the successive dehydrogenation of 1a’ to 2a at [TEMPO] (0.8 M).

[TEMPO] (M)	initial rate (M/min)
0.04	0.0003620
0.08	0.0007759
0.12	0.00152
0.2	0.00193
0.4	0.00165
0.6	0.00155
0.8	0.00195

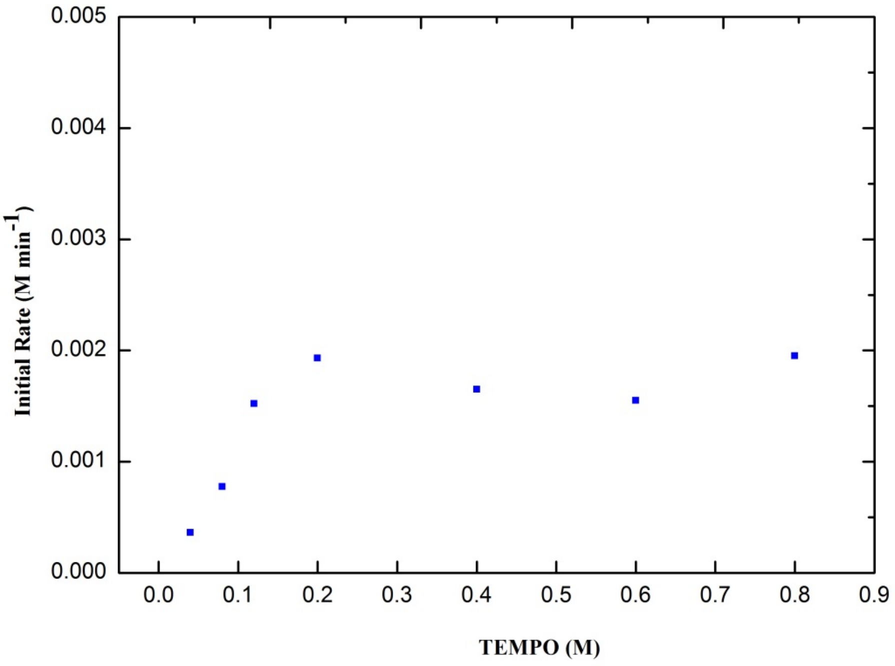

Supplementary Figure 186. Kinetic data from the successive dehydrogenation of **1a'** at varying concentrations of [TEMPO].

General Procedure for Copper-Catalyzed Successive Dehyrogenation (Fig. 3)

The substrates of copper-catalyzed successive dehydrogenation reaction included ketones, aldehydes, alcohols, α,β-unsaturated diesters and *N*-heterocycles. Thus, the different type of substrates was subject to different modified reaction conditions. The detailed conditions were performed by the following described procedures.

Method A: Catalytic $Cu(OAc)_2$/1,10-Phenanthroline System in 1,2-Dichlorobenzene for ketones.

In a nitrogen-filled glovebox, a 25 mL Schlenk tube equipped with a stir bar was charged with ketone (0.2 mmol), $Cu(OAc)_2$ (0.0036g, 0.02 mmol, 10 mol%), 1,10-phenanthroline (0.0036g, 0.02 mmol, 10 mol%) and TEMPO (0.0630g, 0.4 mmol or 0.0945g, 0.6 mmol). The tube was fitted with a rubber septum and moved out of the glove box. Then 1, 2-dichlorobenzene (1.0 mL) was added to the Schlenk tube through the rubber septum using syringes, and then the septum was replaced with a Teflon screwcap under nitrogen flow. The reaction mixture was stirred at 120 ^{o}C for 48 h. Upon cooling to room temperature, the reaction mixture was filtered through a pad of silica gel and washed with 10 mL of ethyl acetate. The filtrate was concentrated under reduced pressure and purified by flash chromatography on silica gel to provide the corresponding product.

Method B: Catalytic $Cu(OAc)_2$/4,4'-Dimethoxy-2,2'-bipyridine System in *tert*-Amyl Alcohol for aldehydes.

In a nitrogen-filled glovebox, a 25 mL Schlenk tube equipped with a stir bar was charged with aldehyde (0.2 mmol), $Cu(OAc)_2$ (0.0036g, 0.02 mmol, 10 mol%), 4,4'-dimethoxy-2,2'-bipyridine (0.0088, 0.04 mmol, 20 mol%) and TEMPO (0.0315g, 0.2 mmol or 0.0630g 0.4 mmol). The tube was fitted with a rubber septum and moved out of the glove box. Then *tert*-Amyl alcohol (1.0 mL) was added in turn to the Schlenk tube through the rubber septum using syringes, and then the septum was replaced with a Teflon screwcap under nitrogen flow. The reaction mixture was stirred at 120 ^{o}C or 100 ^{o}C for 48 h. Upon cooling to room temperature, the reaction mixture

was filtered through a pad of silica gel and washed with 10 mL of ethyl acetate. The filtrate was concentrated under reduced pressure and purified by flash chromatography on silica gel to provide the corresponding product.

Method C: Catalytic $Cu(OAc)_2$/4,4'-Dimethoxy-2,2'-bipyridine System in *tert*-Amyl Alcohol for alcohols

In a nitrogen-filled glovebox, a 25 mL Schlenk tube equipped with a stir bar was charged with alcohol (0.2 mmol), $Cu(OAc)_2$ (0.0036g, 0.02 mmol, 10 mol%), 4,4'-dimethoxy-2,2'-bipyridine (0.0088g, 0.04 mmol, 20 mol% or 0.0044g, 0.02 mmol, 10 mol%) TsOH (0.0035g, 0.02 mmol, 10 mol%) and TEMPO (0.0630g 0.4 mmol or 0.0945g, 0.6 mmol). The tube was fitted with a rubber septum and moved out of the glove box. Then *tert*-Amyl alcohol (1.0 mL) was added to the Schlenk tube through the rubber septum using syringes, and then the septum was replaced with a Teflon screwcap under nitrogen flow. The reaction mixture was stirred at 120 ^oC for 48 h. Upon cooling to room temperature, the reaction mixture was filtered through a pad of silica gel and washed with 10 mL of ethyl acetate. The filtrate was concentrated under reduced pressure and purified by flash chromatography on silica gel to provide the corresponding product.

Method D: Catalytic $Cu(OAc)_2$/4,4'-Dimethoxy-2,2'-bipyridine System in *t*ert-Amyl Alcohol for α,β-unsaturated diesters.

In a nitrogen-filled glovebox, a 25 mL Schlenk tube equipped with a stir bar was charged with 3-substituent propanal (0.2 mmol), $Cu(OAc)_2$ (0.0036g, 0.02 mmol, 10 mol%), 4,4'-dimethoxy-2,2'-bipyridine (0.0088, 0.04 mmol, 20 mol%) dimethyl malonate (0.0661g, 0.5mmol) and TEMPO (0.0315g 0.2 mmol). The tube was fitted with a rubber septum and moved out of the glove box. Then *tert*-Amyl alcohol (1.0 mL) was added in turn to the Schlenk tube through the rubber septum using syringes, and then the septum was replaced with a Teflon screwcap under nitrogen flow. The reaction mixture was stirred at 120 ^oC for 24 h. Upon cooling to room temperature, the reaction mixture was filtered through a pad of silica gel and washed with 10 mL of

ethyl acetate. The filtrate was concentrated under reduced pressure and purified by flash chromatography on silica gel to provide the corresponding product.

Method E: Catalytic $Cu(OAc)_2$/2,2'-Bipyridine System in 1,2-Dichlorobenzene for *N*-Heterocycles.

In a nitrogen-filled glovebox, a 25 mL Schlenk tube equipped with a stir bar was charged with *N*-heterocycle (0.5 mmol), $Cu(OAc)_2$ (0.0090g, 0.05 mmol, 10 mol%), 2,2'-bipyridine (0.0078, 0.05 mmol, 10 mol%) and TEMPO (0.0780g, 0.5 mmol, or 0.1563g 1.0 mmol). The tube was fitted with a rubber septum and moved out of the glove box. Then 1,2-dichlorobenzene (1.0 mL) was added in turn to the Schlenk tube through the rubber septum using syringes, and then the septum was replaced with a Teflon screwcap under nitrogen flow. The reaction mixture was stirred at 120 ºC for 24 h. Upon cooling to room temperature, the reaction mixture was filtered through a pad of silica gel and washed with 10 mL of ethyl acetate. The filtrate was concentrated under reduced pressure and purified by flash chromatography on silica gel to provide the corresponding product.

(2*E*,4*E*)-1,5-diphenylpenta-2,4-dien-1-one (2a)

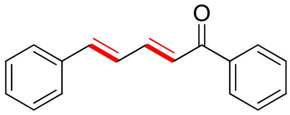

2a

2a (0.2 mmol scale) was synthesized following the *Method A*. The reaction was carried out with 1,5-diphenylpentan-1-one (0.0477g, 0.2 mmol), $Cu(OAc)_2$ (0.0036g, 0.02 mmol, 10 mol%), 1,10-phenanthroline (0.0036g, 0.02 mmol, 10 mol%) and TEMPO (0.0630g, 0.4 mmol) in 1, 2-dichlorobenzene (1.0 mL) at 120 ºC for 48 h. After concentration and purification by flash chromatography on silica gel (petroleum ether/diethyl ether = 100:6), the product **2a** was obtained in 76% yield.

Physical state: white solid;

HRMS (*m*/*z*): calculated for $C_{17}H_{14}OH^+$ $[M+H]^+$, 235.1117; found, 235.1117;

^{1}**H NMR** (400 MHz, $CDCl_3$): δ 7.98-7.96 (m, 2H), 7.63-7.53 (m, 2H), 7.49-7.46 (m, 4H), 7.38-7.29 (m, 3H), 7.08 (d, *J* = 14.9 Hz, 1H), 7.06-6.96 (m, 2H);

^{13}**C NMR** (100 MHz, $CDCl_3$): δ 190.4, 144.8, 141.8, 138.1, 136.0, 132.6, 129.1, 128.8, 128.5, 128.3, 127.2, 126.9, 125.3.

(3*E*,5*E*)-6-phenylhexa-3,5-dien-2-one (2b)

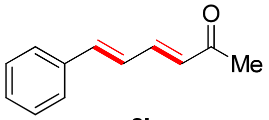

2b

2b (0.4 mmol scale) was synthesized following the *Method A*. The reaction was carried out with 6-phenylhexan-2-one (0.0704g, 0.4 mmol), $Cu(OAc)_2$ (0.0072g, 0.04 mmol, 10 mol%), 1,10-phenanthroline (0.0072g, 0.04 mmol, 10 mol%) and TEMPO (0.1260g, 0.8 mmol) in 1, 2-dichlorobenzene (2.0 mL) at 120 °C for 48 h. After concentration and purification by flash chromatography on silica gel (petroleum ether/diethyl ether = 100:6), the product **2b** was obtained in 60% yield.

Physical state: pale yellow solid;

HRMS (*m/z*): calculated for $C_{12}H_{12}OH^+$ $[M+H]^+$, 173.0961; found, 173.0961;

^{1}**H NMR** (400 MHz, $CDCl_3$): δ 7.47 (d, *J* = 7.0 Hz, 2H), 7.38-7.28 (m, 4H), 6.97-6.85 (m, 2H), 6.26 (d, *J* = 15.5 Hz, 1H), 2.32 (s, 3H);

^{13}**C NMR** (100 MHz, $CDCl_3$): δ 198.4, 143.4, 141.2, 135.9, 130.4, 129.2, 128.8, 127.2, 126.6, 27.3.

(3*E*,5*E*)-6-(furan-2-yl)hexa-3,5-dien-2-one (2c)

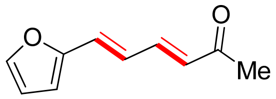

2c

2c (0.4 mmol scale) was synthesized following the *Method A*. The reaction was carried out with 6-(furan-2-yl)hexan-2-one (0.0664g, 0.4 mmol), $Cu(OAc)_2$ (0.0072g, 0.04 mmol, 10 mol%), 1,10-phenanthroline (0.0072g, 0.04 mmol, 10 mol%) and TEMPO (0.1260g, 0.8 mmol) in 1, 2-dichlorobenzene (2.0 mL) at 120 °C for 48 h. After

concentration and purification by flash chromatography on silica gel (petroleum ether/diethyl ether = 100:6), the product **2c** was obtained in 62% yield.

Physical state: yellow solid;

HRMS (*m*/*z*): calculated for $C_{10}H_{10}O_2H^+$ $[M+H]^+$, 163.0754; found, 163.0754;

^{1}H NMR (400 MHz, $CDCl_3$): δ7.45 (s, 1H), 7.26-7.19 (dd, *J* = 15.4, 10.4 Hz, 1H), 6.82-6.69 (m, 2H), 6.48-6.44 (m, 2H), 6.25 (d, *J* = 15.4 Hz, 1H), 2.30 (s, 3H);

^{13}C NMR (100 MHz, $CDCl_3$): δ 198.3, 152.2, 143.8, 142.9, 130.2, 127.6, 124.9, 112.2, 112.1, 27.5.

benzophenone (2d)

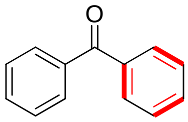

2d

2d (0.2 mmol scale) was synthesized following the *Method A* with slight modifications. The reaction was carried out with cyclohexyl phenyl ketone (0.0377g, 0.2 mmol), $Cu(OAc)_2$ (0.0072g, 0.04 mmol, 20 mol%), 1,10-phenanthroline (0.0072g, 0.04 mmol, 20 mol%) and TEMPO (0.0945g, 0.6 mmol) in 1, 2-dichlorobenzene (1.0 mL) at 130 °C for 48 h. After concentration and purification on flash chromatography on silica gel (petroleum ether/diethyl ether = 100:6), the product **2d** was obtained in 81% yield.

Physical state: white solid;

HRMS (*m*/*z*): calculated for $C_{13}H_{10}OH^+$ $[M+H]^+$, 183.084; found, 183.0804;

^{1}H NMR (400 MHz, $CDCl_3$): δ 7.82-7.80 (m, 4H), 7.61-7.58 (m, 2H), 7.51-7.47 (m, 4H);

^{13}C NMR (100 MHz, $CDCl_3$): δ 196.8, 137.6, 132.4, 130.1, 128.3.

(*E*)-chalcone (2e)

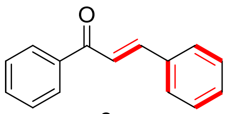

2e

2e (0.2 mmol scale) was synthesized following the *Method A* with slight modifications. The reaction was carried out with 3-cyclohexyl-1-phenylpropan-1-one (0.0432g, 0.2

mmol), $Cu(OAc)_2$ (0.0072g, 0.04 mmol, 20 mol%), 1,10-phenanthroline (0.0072g, 0.04 mmol, 20 mol%) and TEMPO (0.1260g, 0.8 mmol) in 1, 2-dichlorobenzene (1.0 mL) at 130 °C for 48 h. After concentration and purification by flash chromatography on silica gel (petroleum ether/diethyl ether = 100:6), the product **2e** was obtained in 65% yield.

Physical state: pale yellow solid;

HRMS (*m/z*): calculated for $C_{15}H_{12}OH^+$ $[M+H]^+$, 209.0961; found, 209.0961;

^{1}H NMR (400 MHz, $CDCl_3$): δ 8.03 (d, J = 7.3 Hz, 2H), 7.82 (d, J = 15.7 Hz, 1H), 7.66-7.64 (m, 2H), 7.61-7.49 (m, 4H), 7.43-7.42 (m, 3H);

^{13}C NMR (100 MHz, $CDCl_3$): δ 190.6, 144.9, 138.2, 134.9, 132.8, 130.6, 129.0, 128.6, 128.51, 128.45, 122.1.

Cyclohexenone (2f) and Phenol (2g)

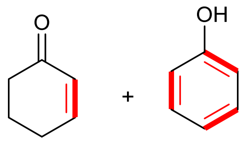

2f 2g

2f and 2g (0.4 mmol scale) were synthesized following the *Method A* with slight modifications. The reaction was carried out with cyclohexanone (0.0393g, 0.4 mmol), $Cu(OAc)_2$ (0.0072g, 0.04 mmol, 10 mol%), 1,10-phenanthroline (0.0072g, 0.04 mmol, 10 mol%), TsOH (0.0070g, 0.02 mmol, 10 mol%) and TEMPO (0.1248g, 0.8 mmol) in *tert*-Amyl alcohol (1.0 mL) at 120 °C for 48 h. After the reaction was finished, the product **2f** (68% GC yield) **and 2g** (16% GC yield) were analyzed by gas chromatography using dodecane as an internal standard.

(2*E*,4*E*)-5-phenylpenta-2,4-dienal (4a)

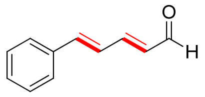

4a

4a (0.2 mmol scale) was synthesized following the *Method B.* The reaction was carried out with 5-phenylpentanal (0.0328g, 0.2mmol), $Cu(OAc)_2$ (0.0036g, 0.02 mmol, 10 mol%), 4,4'-dimethoxy-2,2'-bipyridine (0.0088g, 0.04 mmol, 20 mol%), TEMPO

(0.0945g, 0.6 mmol) in *tert*-Amyl alcohol (1.0 mL) at 120 °C for 48 h. in which was used. After concentration and purification by flash chromatography on silica gel (petroleum ether/diethyl ether, 100:6), the product **4a** was obtained in 39% yield.

Physical state: red oil;

HRMS (*m*/*z*): calculated for $C_{11}H_{10}OH^+$ $[M+H]^+$, 159.0804; found, 159.0804;

^{1}H NMR (400 MHz, $CDCl_3$): δ 9.62 (d. *J* = 8.0 Hz, 1H), 7.52-7.49 (m, 2H), 7.41-7.33 (m, 3H), 7.30-7.23 (m, 1H), 7.05-6.96 (m, 2H), 6.27 (dd, *J* = 15.2 Hz, 8.0 Hz, 1H);

^{13}C NMR (100 MHz, $CDCl_3$): δ 193.5, 152.0, 142.4, 135.5, 131.5, 129.6, 128.9, 127.5, 126.1.

(*Z*)-2-phenylpenta-2,4-dienal (4b)

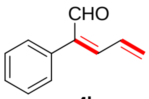

4b

4b (0.2 mmol scale) was synthesized following the *Method B* with slight modifications. The reaction was carried out with 2-phenylpentanal (0.0324g, 0.2 mmol), $Cu(OAc)_2$ (0.0036g, 0.02 mmol, 10 mol%), 4,4'-dimethoxy-2,2'-bipyridine (0.0088, 0.04 mmol, 20 mol%), TsOH (0.0035g, 0.02 mmol, 10 mol%) and TEMPO (0.0630g, 0.4 mmol) in 1,2-dichlorobenzene (1.0 mL) at 100 °C for 48 h. After concentration and purification by flash chromatography on silica gel (petroleum ether/diethyl ether = 100:6), the product **4b** was obtained in 35% yield.

Physical state: pale yellow oil;

HRMS (*m*/*z*): calculated for $C_{11}H_{10}OH^+$ $[M+H]^+$, 159.0804; found, 159.0805;

^{1}H NMR (400 MHz, $CDCl_3$): δ 9.69 (s, 1H), 7.44-7.34 (m, 3H), 7.25-7.21 (m, 2H), 7.06 (d, *J* = 11.3 Hz, 1H), 6.76-6.67 (m, 1H), 5.83 (d, *J* = 16.9 Hz, 1H), 5.60 (d, *J* = 10.0 Hz, 1H);

^{13}C NMR (100 MHz, $CDCl_3$): δ 193.3, 149.2, 142.1, 132.9, 132.2, 129.8, 128.3, 128.2, 127.7.

(*Z*)-2-phenylpenta-2,4-dienal (4c)

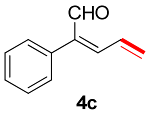

4c

4c (0.2 mmol scale) was synthesized following the *Method B* with slight modifications. The reaction was carried out with (*E*)-2-phenyl-2-pentenal (0.0320g, 0.2 mmol), $Cu(OAc)_2$ (0.0036g, 0.02 mmol, 10 mol%), 4,4'-dimethoxy-2,2'-bipyridine (0.0088, 0.04 mmol, 20 mol%), TsOH (0.0035g, 0.02 mmol, 10 mol%) and TEMPO (0.0315g, 0.2 mmol) in 1,2-dichlorobenzene (1.0 mL) at 100 °C for 24 h. After concentration and purification on flash chromatography on silica gel (petroleum ether/diethyl ether, 100:6), the product **4c** was obtained in 59% yield.

benzaldehyde (4d)

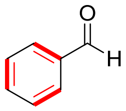

4d

4d (0.2 mmol scale) was synthesized following the *Method B* with slight modifications. The reaction was carried out with cyclohexanecarbaldehyde (0.0224g, 0.2 mmol), $Cu(OAc)_2$ (0.0072g, 0.04 mmol, 20 mol%), 4,4'-dimethoxy-2,2'-bipyridine (0.0176, 0.08 mmol, 40 mol%), TsOH (0.0035g, 0.02 mmol, 10 mol%) , piperidine (4 μL, 0.04 mmol, 20 mol%) and TEMPO (0.0945g, 0.6 mmol) in *tert*-Amyl alcohol (1.0 mL) at 120 °C for 24 h. After the reaction was finished, the product **4d** was analyzed by gas chromatography using dodecane as an internal standard (76% GC yield).

(*E*)-3-(4-isopropylphenyl)-2-methylacrylaldehyde (4e)

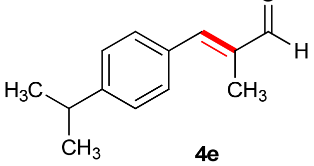

4e

4e (0.2 mmol scale) was synthesized following the *Method B* with slight modifications. The reaction was carried out with 3-(4-isopropylphenyl)-2-methylpropanal (0.0381g, 0.2 mmol), $Cu(OAc)_2$ (0.0036g, 0.02 mmol, 10 mol%), 4,4'-dimethoxy-2,2'-bipyridine (0.0044, 0.02 mmol, 10 mol%), TsOH (0.0035g, 0.02 mmol, 10 mol%) and TEMPO

(0.0315g, 0.2 mmol) in *tert*-Amyl alcohol (1.0 mL) at 120 °C for 24 h. After concentration and purification on flash chromatography on silica gel (petroleum ether/diethyl ether, 100:6), the product **4e** was obtained in 32% yield.

Physical state: pale yellow oil;

HRMS (*m*/*z*): calculated for $C_{13}H_{16}OH^+$ $[M+H]^+$, 189.1274; found, 189.1275;

^{1}H NMR (400 MHz, $CDCl_3$): δ 9.57 (s, 1H), 7.49 (d, *J* = 8.2 Hz, 2H), 7.32 (d, *J* = 8.2 Hz, 2H), 7.24 (s, 1H), 2.99-2.92 (m, 1H), 2.09 (d, *J* = 1.2 Hz, 3H), 1.29 (s, 3H), 1.27 (s, 3H);

^{13}C NMR (100 MHz, $CDCl_3$): δ.195.7, 150.9, 150.0, 137.5, 132.8, 130.3, 126.9, 34.1, 29.7, 23.8, 11.0.

(2*E*,4*E*)-5-phenylpenta-2,4-dienal (6a)

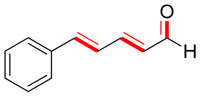

6a

6a (0.4 mmol scale) was synthesized following the *Method C* with slight modifications. The reaction was carried out with 5-phenyl-1-pentanol (0.0657g, 0.4 mmol), $Cu(OAc)_2$ (0.0072g, 0.04 mmol, 10 mol%), 4,4'-dimethoxy-2,2'-bipyridine (0.0088, 0.04 mmol, 10 mol%), TsOH (0.0070g, 0.04 mmol, 10 mol%) and TEMPO (0.1872g, 1.2 mmol) in *tert*-Amyl alcohol (3.0 mL) at 120 °C for 48 h. After concentration and purification by flash chromatography on silica gel (petroleum ether/diethyl ether = 100:6), the product **6a** was obtained in 55% yield.

(2*E*,4*E*,6*E*)-7-phenylhepta-2,4,6-trienal (6b)

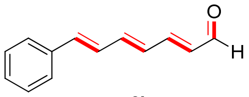

6b

6b (0.4 mmol scale) was synthesized following the *Method C* with slight modifications. The reaction was carried out with 7-phenylheptan-1-ol (0.0769g, 0.4 mmol), $Cu(OAc)_2$ (0.0072g, 0.04 mmol, 10 mol%), 4,4'-dimethoxy-2,2'-bipyridine (0.0088, 0.04 mmol, 10 mol%), TsOH (0.0070g, 0.04 mmol, 10 mol%) and TEMPO (0.2496g, 1.6 mmol) in *tert*-Amyl alcohol (3.0 mL) at 120 °C for 48 h. After concentration and purification by

flash chromatography on silica gel (petroleum ether/diethyl ether = 100:6), the product **6b** was obtained in 28% yield.

Physical state: yellow solid;

HRMS (*m*/*z*): calculated for $C_{13}H_{12}OH^+$ $[M+H]^+$, 185.0961; found, 185.0961;

^{1}H NMR (400 MHz, $CDCl_3$): δ 9.59 (d, *J* = 8.0 Hz, 1H), 7.46 (d, *J* = 7.4 Hz, 2H), 7.38-7.34 (m, 2H), 7.32-7.28 (m, 1H), 7.19 (dd, *J* = 15.2, 11.2 Hz, 1H), 6.92 (dd, *J* = 15.2, 10.3 Hz, 1H), 6.84-6.79 (m, 2H), 6.57 (dd, *J* = 14.0, 11.3 Hz, 1H), 6.20 (dd, *J* = 15.2, 8.0 Hz, 1H);

^{13}C NMR (100 MHz, $CDCl_3$): δ 193.5, 151.7, 142.7, 138.3, 136.3, 131.2, 130.1, 128.8, 127.7, 127.0.

(*E*)-4-phenylbut-3-en-2-one (6c)

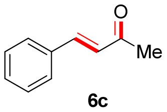

6c

6c (0.5 mmol scale) was synthesized following the *Method C* with slight modifications. The reaction was carried out with 4-phenylbutan-2-ol (0.0751g, 0.5 mmol), $Cu(OAc)_2$ (0.0090g, 0.05 mmol, 10 mol%), 4,4'-dimethoxy-2,2'-bipyridine (0.0106, 0.05 mmol, 10 mol%), TsOH (0.0088g, 0.05 mmol, 10 mol%) and TEMPO (0.1560g, 1.0 mmol) in 1,2-dichlorobenzene (2.0 mL) at 120 °C for 24 h. After concentration and purification by flash chromatography on silica gel (petroleum ether/diethyl ether = 100:6), the product **6c** was obtained in 73% yield.

Physical state: pale yellow oil;

HRMS (*m*/*z*): calculated for $C_{10}H_{10}OH^+$ $[M+H]^+$, 147.0804; found, 147.0804;

^{1}H NMR (400 MHz, $CDCl_3$): δ 7.54-7.50 (m, 3H), 7.41-7.40 (m, 3H), 6.72 (t, *J* = 16.3 Hz, 1H), 2.39 (s, 3H);

^{13}C NMR (100 MHz, $CDCl_3$): δ 198.3, 143.4, 134.4, 130.5, 128.9, 128.2, 127.1, 27.5.

methyl (2*E*,4*E*)-6-oxohexa-2,4-dienoate (6d)

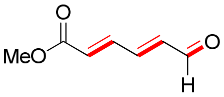

6d

6d (0.4 mmol scale) was synthesized following the *Method C* with slight modifications. The reaction was carried out with methyl 6-hydroxyhexanoate (0.0548g, 0.4 mmol), $Cu(OAc)_2$ (0.0072g, 0.04 mmol, 10 mol%) 4,4'-dimethoxy-2,2'-bipyridine (0.0088, 0.04 mmol, 10 mol%), TsOH (0.0070g, 0.04 mmol, 10 mol%) and TEMPO (0.1890g, 1.2 mmol) in *tert*-Amyl alcohol (3.0 mL) at 120 °C for 48 h. After concentration and purification by flash chromatography on silica gel (petroleum ether/diethyl ether = 100:10), the product **6d** was obtained in 51% yield.

Physical state: pale yellow solid;

HRMS (*m/z*): calculated for $C_7H_8O_3H^+$ $[M+H]^+$, 141.0546; found, 141.0547;

^{1}H NMR (400 MHz, $CDCl_3$): δ 9.68 (d, *J* = 7.7 Hz, 1H), 7.44 (dd, *J* = 15.4, 11.3 Hz, 1H), 7.18 (dd, *J* = 15.4, 11.3 Hz, 1H), 6.43 (dd, *J* = 15.4, 7.7 Hz, 1H), 6.32 (d, *J* = 15.4 Hz, 1H), 3.81 (s, 3H);

^{13}C NMR (100 MHz, $CDCl_3$): δ 192.9, 165.9, 147.1, 140.6, 137.1, 129.4, 52.1.

(2*E*,4*E*)-*N*-methyl-6-oxo-*N*-phenylhexa-2,4-dienamide (6e)

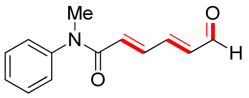

6e

6e (0.4 mmol scale) was synthesized following the *Method C* with slight modifications. The reaction was carried out with 6-hydroxy-*N*-methyl-*N*-phenylhexanamide (0.0885g, 0.4 mmol), $Cu(OAc)_2$ (0.0072g, 0.04 mmol, 10 mol%) 4,4'-dimethoxy-2,2'-bipyridine (0.0088, 0.04 mmol, 10 mol%), TsOH (0.0070g, 0.04 mmol, 10 mol%) and TEMPO (0.1890g, 1.2 mmol) in *tert*-Amyl alcohol (3.0 mL) at 120 °C for 48 h. After concentration and purification by flash chromatography on silica gel (petroleum ether/diethyl ether = 100:10), the product **6e** was obtained in 56% yield.

Physical state: pale yellow solid;

HRMS (*m/z*): calculated for $C_{13}H_{13}NO_2H^+$ $[M+H]^+$, 216.1019; found, 216.1019;

^{1}H NMR (400 MHz, $CDCl_3$): δ 9.56 (d, *J* = 7.8 Hz, 1H), 7.48-7.37 (m, 4H), 7.22-7.20 (m, 2H), 7.04-6.97 (m, 1H), 6.35 (q, *J* = 7.7 Hz, 1H), 6.26 (d, *J* = 14.9 Hz, 1H), 3.39 (s, 3H);

^{13}C NMR (100 MHz, $CDCl_3$): δ 193.0, 164.4, 148.2, 142.8, 137.6, 135.8, 130.5, 129.7, 127.9, 127.1, 37.5.

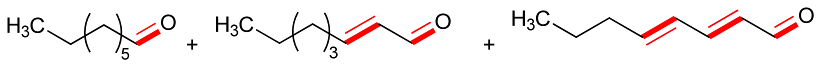

6f **6g** **6h**

6f + 6g + 6h (0.4 mmol scale) was synthesized following the *Method C* with slight modifications. The reaction was carried out with 1-octanol (0.0521g, 0.4 mmol), $Cu(OAc)_2$ (0.0072g, 0.04 mmol, 10 mol%) 4,4'-dimethoxy-2,2'-bipyridine (0.0088, 0.04 mmol, 10 mol%), TsOH (0.0070g, 0.04 mmol, 10 mol%) and TEMPO (0.1890g, 1.2 mmol) in *tert*-Amyl alcohol (3.0 mL) at 120 °C for 48 h. After the reaction was finished, the mixture products (**6f** + **6g** + **6h**)were detected by GC-MS.

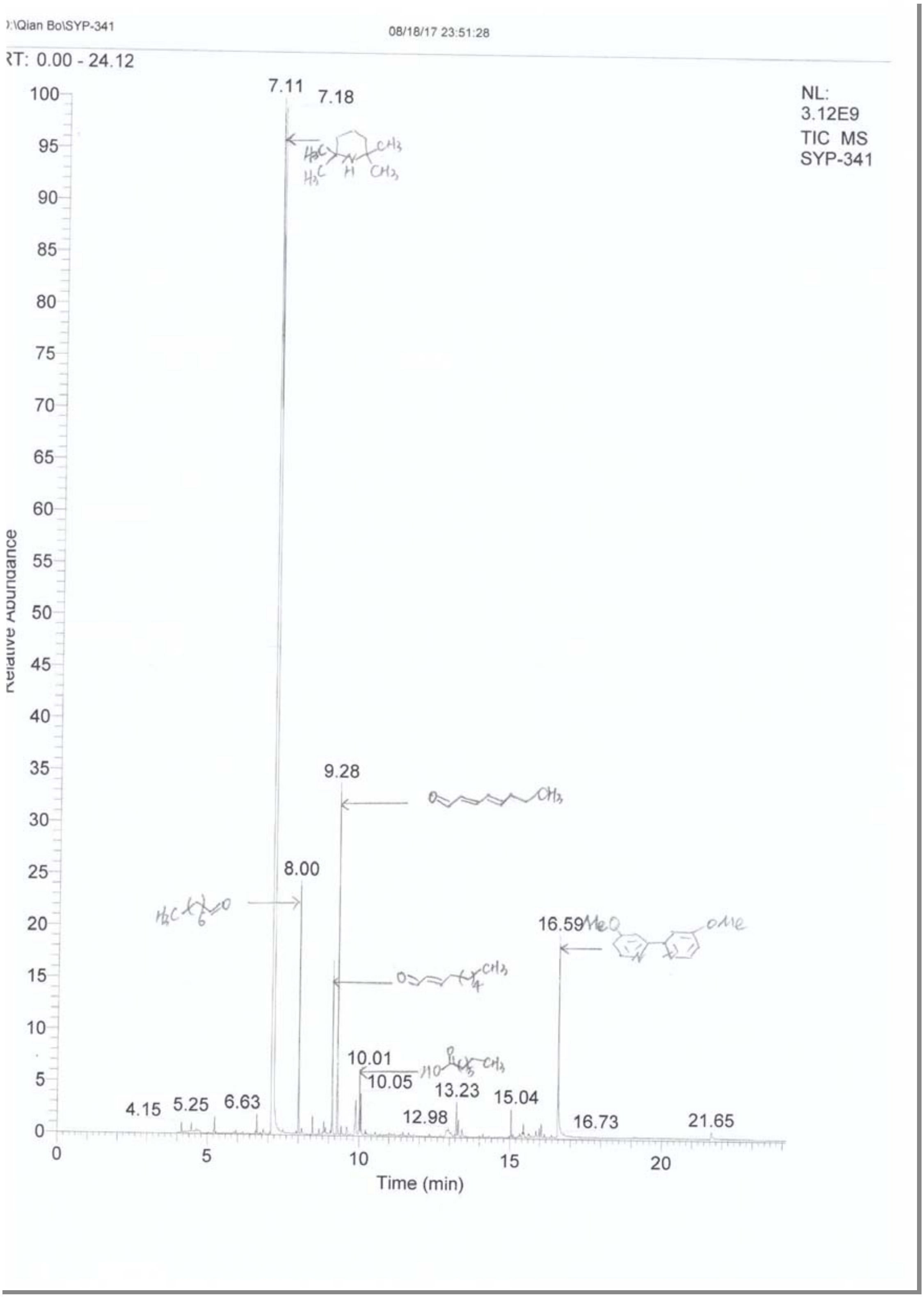

Supplementary Figure 187. The GC-MS spectrum for the mixture products (**6f + 6g + 6h**)

dimethyl (*E*)-2-(3-phenylallylidene)malonate (9a)

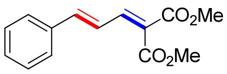

9a

9a (0.2 mmol scale) was synthesized following the *Method D*. The reaction was carried out with 3-phenylpropionaldehyde (0.0268g, 0.2mmol), $Cu(OAc)_2$ (0.0036g, 0.02 mmol, 10 mol%), 4,4'-dimethoxy-2,2'-bipyridine (0.0088g, 0.04 mmol, 20 mol%), TEMPO (0.0315g, 0.2 mmol) and dimethyl malonate (0.0661g, 0.5mmol) in *tert*-Amyl alcohol (1.0 mL) at 120 °C for 24 h. After concentration and purification by flash chromatography on silica gel (petroleum ether/diethyl ether = 100:8), the product **9a** was obtained in 87% yield.

Physical state: slight yellow solid;

HRMS (*m*/*z*): calculated for $C_{14}H_{14}O_4H^+$ $[M+H]^+$, 247.0965; found, 247.0965;

^{1}H NMR (400 MHz, $CDCl_3$): δ 7.58 (d, *J* = 11.6 Hz, 1H), 7.52-7.50 (m, 2H), 7.40-7.33 (m, 3H), 7.28-7.24 (m, 1H), 7.06 (d, *J* = 15.4 Hz, 1H), 3.91 (s, 3H), 3.83 (s, 3H);

^{13}C NMR (100 MHz, $CDCl_3$): δ 165.7, 165.1, 146.2, 145.2, 135.4, 129.9, 128.8, 127.8, 123.9, 123.2, 52.4, 52.3.

dimethyl (*E*)-2-(3-(p-tolyl)allylidene)malonate (9b)

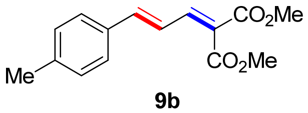

9b

9b (0.2 mmol scale) was synthesized following the *Method D*. The reaction was carried out with 3-(*p*-tolyl)propanal (0.0296g, 0.2mmol), $Cu(OAc)_2$ (0.0036g, 0.02 mmol, 10 mol%), 4,4'-dimethoxy-2,2'-bipyridine (0.0088g, 0.04 mmol, 20 mol%), TEMPO (0.0315g, 0.2 mmol) and dimethyl malonate (0.0661g, 0.5mmol) in *tert*-Amyl alcohol (1.0 mL) at 120 °C for 24 h. After concentration and purification by flash

chromatography on silica gel (petroleum ether/diethyl ether = 100:8), the product **9b** was obtained in 83% yield.

Physical state: yellow solid;

HRMS (*m/z*): calculated for $C_{15}H_{16}O_4H^+$ $[M+H]^+$,261.1121; found, 261.1120;

^{1}H NMR (400 MHz, $CDCl_3$): δ 7.57 (d, *J* = 11.6 Hz, 1H), 7.40 (d, *J* = 8.1 Hz, 2H), 7.27-7.17 (m, 3H), 7.03 (d, *J* = 15.4 Hz, 1H), 3.90 (s, 3H), 3.82 (s, 3H), 2.37 (s, 3H);

^{13}C NMR (100 MHz, $CDCl_3$): δ 165.8, 165.2, 146.7, 145.4, 140.4, 132.8, 129.6, 127.9, 123.2, 122.3, 52.3, 52.2, 21.4.

dimethyl (*E*)-2-(3-(m-tolyl)allylidene)malonate (9c)

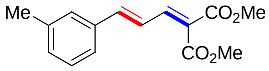

9c

9c (0.2 mmol scale) was synthesized following the *Method D*. The reaction was carried out with 3-(*m*-tolyl)propanal (0.0296g, 0.2mmol), $Cu(OAc)_2$ (0.0036g, 0.02 mmol, 10 mol%), 4,4'-dimethoxy-2,2'-bipyridine (0.0088g, 0.04 mmol, 20 mol%), TEMPO (0.0315g, 0.2 mmol) and dimethyl malonate (0.0661g, 0.5mmol) in *tert*-Amyl alcohol (1.0 mL) at 120 °C for 24 h. After concentration and purification by flash chromatography on silica gel (petroleum ether/diethyl ether = 100:8), the product **9c** was obtained in 76% yield.

Physical state: white solid;

HRMS (*m/z*): calculated for $C_{15}H_{16}O_4H^+$ $[M+H]^+$, 261.1121; found, 261.1121;

^{1}H NMR (400 MHz, $CDCl_3$): δ 7.56 (d, *J* = 11.6 Hz, 1H), 7.31-7.23 (m, 4H), 7.16 (m. *J* = 7.2 Hz, 1H), 7.02 (d, *J* = 15.4 Hz, 1H), 3.90 (s, 3H), 3.82 (s, 3H), 2.36 (s, 3H);

^{13}C NMR (100 MHz, $CDCl_3$): δ 165.7, 165.1, 146.4, 145.4, 138.5, 135.4, 130.8, 128.7, 128.4, 125.1, 123.7, 123.0, 52.3, 52.2, 21.2.

dimethyl (*E*)-2-(3-(4-fluorophenyl)allylidene)malonate (9d)

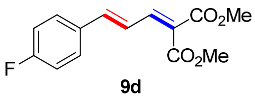

9d

9d (0.2 mmol scale) was synthesized following the *Method D*. The reaction was carried out with 3-(4-fluorophenyl)propanal (0.0304g, 0.2mmol), $Cu(OAc)_2$ (0.0036g, 0.02 mmol, 10 mol%), 4,4'-dimethoxy-2,2'-bipyridine (0.0088g, 0.04 mmol, 20 mol%), TEMPO (0.0315g, 0.2 mmol) and dimethyl malonate (0.0661g, 0.5mmol) in *tert*-Amyl alcohol (1.0 mL) at 120 °C for 24 h. After concentration and purification by flash chromatography on silica gel (petroleum ether/diethyl ether = 100:8), the product **9d** was obtained in 67% yield.

Physical state: pale yellow solid;

HRMS (*m/z*): calculated for $C_{14}H_{13}FO_4H^+$ $[M+H]^+$, 265.0871; found, 265.0870;

^{1}H NMR (400 MHz, $CDCl_3$): δ 7.55 (d, *J* = 11.5Hz, 1H), 7.51-7.47 (m, 2H), 7.23-7.17 (m, 1H), 7.09-7.00 (m, 3H), 3.90 (s, 3H), 3.82 (m, 3H);

^{19}F NMR (377 MHz): δ -109.88;

^{13}C NMR (100 MHz, $CDCl_3$): δ 165.6, 165.1, 163.6 (d, *J* = 251.1 Hz), 146.1, 143.7, 131.8 (d, *J* = 3.3 Hz), 129.6 (d, *J* = 8.3 Hz), 124.0, 123.0 (d, *J* = 2.2 Hz), 116.0 (d, *J* = 22.0 Hz), 52.4, 52.3.

dimethyl (*E*)-2-(3-(4-chlorophenyl)allylidene)malonate (9e)

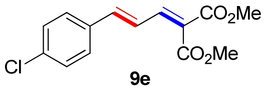

9e

9e (0.2 mmol scale) was synthesized following the *Method D*. The reaction was carried out with 3-(4-chlorophenyl)propanal (0.0337g, 0.2mmol), $Cu(OAc)_2$ (0.0036g, 0.02 mmol, 10 mol%), 4,4'-dimethoxy-2,2'-bipyridine (0.0088g, 0.04 mmol, 20 mol%), TEMPO (0.0315g, 0.2 mmol) and dimethyl malonate (0.0661g, 0.5mmol) in *tert*-Amyl alcohol (1.0 mL) at 120 °C for 24 h. After concentration and purification by flash chromatography on silica gel (petroleum ether/diethyl ether = 100:8), the product **9e** was obtained in 64% yield.

Physical state: pale yellow solid;

HRMS (*m/z*): calculated for $C_{14}H_{13}ClO_4H^+$ $[M+H]^+$, 281.0575; found, 281.0574;

^{1}H NMR (400 MHz, $CDCl_3$): δ 7.55 (d, *J* = 11.6 Hz, 1H), 7.43 (d, *J* = 8.5 Hz, 2H), 7.34 (d, *J* = 8.5 Hz, 2H), 7.28-7.21 (m, 1H), 7.00 (d, *J* = 15.4 Hz, 1H), 3.90 (s, 3H), 3.83 (s, 3H);

^{13}C NMR (100 MHz, $CDCl_3$): δ 165.6, 165.1, 145.8, 143.5, 135.7, 134.0, 129.1, 129.0, 124.4, 123.7, 52.4, 52.3.

dimethyl (*E*)-2-(3-(3-chlorophenyl)allylidene)malonate (9f)

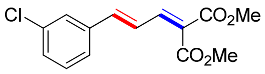

9f

9f (0.2 mmol scale) was synthesized following the *Method D* with slight modifications. The reaction was carried out with 3-(3-chlorophenyl)propanal (0.0337g, 0.2mmol), $Cu(OAc)_2$ (0.0036g, 0.02 mmol, 10 mol%), 4,4'-dimethoxy-2,2'-bipyridine (0.0088g, 0.04 mmol, 20 mol%), TEMPO (0.0315g, 0.2 mmol) and dimethyl malonate (0.0661g, 0.5mmol) in 1, 2-dichlorobenzene (1.0 mL) at 120 °C for 24 h. After concentration and purification by flash chromatography on silica gel (petroleum ether/diethyl ether = 100:8), the product **9f** was obtained in 73% yield.

Physical state: pale yellow solid;

HRMS (*m/z*): calculated for $C_{14}H_{13}ClO_4H^+$ $[M+H]^+$, 281.0575; found, 281.0574;

^{1}H NMR (400 MHz, $CDCl_3$): δ 7.54 (d, J = 11.6 Hz, 1H), 7.48 (s, 1H), 7.38-7.22 (m, 4H), 6.98 (d, J = 15.5 Hz, 1H), 3.91 (s, 3H), 3.83 (s, 3H);

^{13}C NMR (100 MHz, $CDCl_3$): δ 165.5, 165.0, 145.4, 143.1, 137.3, 134.9, 130.1, 129.7, 127.5, 126.0, 125.0, 124.4, 52.5, 52.4.

dimethyl (*E*)-2-(3-(2-chlorophenyl)allylidene)malonate (9g)

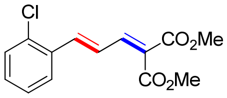

9g

9g (0.2 mmol scale) was synthesized following the *Method D*. The reaction was carried out with 3-(2-chlorophenyl)propanal (0.0337g, 0.2mmol), $Cu(OAc)_2$ (0.0036g, 0.02 mmol, 10 mol%), 4,4'-dimethoxy-2,2'-bipyridine (0.0088g, 0.04 mmol, 20 mol%), TEMPO (0.0315g, 0.2 mmol) and dimethyl malonate (0.0661g, 0.5mmol) in *tert*-Amyl alcohol (1.0 mL) at 120 °C for 24 h. After concentration and purification by flash

chromatography on silica gel (petroleum ether/diethyl ether = 100:8), the product **9g** was obtained in 73% yield.

Physical state: yellow oil;

HRMS (*m/z*): calculated for $C_{14}H_{13}ClO_4H^+$ $[M+H]^+$, 281.0575; found, 281.0575;

^{1}H NMR (400 MHz, $CDCl_3$): δ 7.67-7.64 (m, 1H), 7.60 (d, *J* = 11.5 Hz, 1H), 7.49 (d, *J* = 15.5 Hz, 1H), 7.42-7.37 (m, 1H), 7.30-7.23 (m, 3H), 3.90 (s, 3H), 3.83 (s, 3H);

^{13}C NMR (100 MHz, $CDCl_3$): δ 165.5, 164.9, 145.6, 140.3, 134.5, 133.6, 130.6, 130.1, 127.4, 127.0, 125.4, 125.1, 52.4, 52.3.

dimethyl (*E*)-2-(3-(4-bromophenyl)allylidene)malonate (9h)

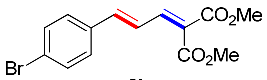

9h

9h (0.2 mmol scale) was synthesized following the *Method D* with slight modifications. The reaction was carried out with 3-(4-bromophenyl)propanal (0.0.0426g, 0.2mmol), $Cu(OAc)_2$ (0.0036g, 0.02 mmol, 10 mol%), 4,4'-dimethoxy-2,2'-bipyridine (0.0088g, 0.04 mmol, 20 mol%), TEMPO (0.0315g, 0.2 mmol) and dimethyl malonate (0.0661g, 0.5mmol) in 1, 2-dichlorobenzene (1.0 mL) at 120 °C for 24 h.. After concentration and purification by flash chromatography on silica gel (petroleum ether/diethyl ether = 100:8), the product **9h** was obtained in 63% yield.

Physical state: pale yellow solid;

HRMS (*m/z*): calculated for $C_{14}H_{13}BrO_4H^+$ $[M+H]^+$, 325.0070; found, 325.0068;

^{1}H NMR (400 MHz, $CDCl_3$): δ 7.56-7.49 (m, 3H), 7.36 (d, *J* = 8.5 Hz, 2H), 7.29-7.23 (m, 1H), 6.98 (d, *J* = 15.4 Hz, 1H), 3.90 (s, 3H), 3.83 (s, 3H);

^{13}C NMR (100 MHz, $CDCl_3$): δ 165.6, 165.1, 145.8, 143.5, 134.4, 132.1, 129.2, 124.5, 124.1, 123.8, 52.5, 52.4.

dimethyl (*E*)-2-(3-(3-bromophenyl)allylidene)malonate (9i)

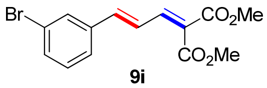

9i

9i (0.2 mmol scale) was synthesized following the *Method D*. The reaction was carried out with 3-(3-bromophenyl)propanal (0.0426g, 0.2mmol), $Cu(OAc)_2$ (0.0036g, 0.02 mmol, 10 mol%), 4,4'-dimethoxy-2,2'-bipyridine (0.0088g, 0.04 mmol, 20 mol%), TEMPO (0.0315g, 0.2 mmol) and dimethyl malonate (0.0661g, 0.5mmol) in *tert*-Amyl alcohol (1.0 mL) at 120 °C for 24 h. After concentration and purification on flash chromatography on silica gel (petroleum ether/diethyl ether, 100:8), the product **9i** was obtained in 65% yield.

Physical state: pale yellow solid;

HRMS (*m/z*): calculated for $C_{14}H_{13}BrO_4H^+$ $[M+H]^+$, 325.0070; found, 325.0068;

^{1}H NMR (400 MHz, $CDCl_3$): δ 7.63 (s, 1H), 7.53 (d, *J* = 11.6 Hz, 1H), 7.47-7.40 (m, 2H), 7.28-7.21 (m, 2H), 6.96 (d, *J* = 15.4 Hz, 1H), 3.91 (s, 3H), 3.83 (s, 3H);

^{13}C NMR (100 MHz, $CDCl_3$): δ 165.5, 164.9, 145.4, 143.0, 137.5, 132.6, 130.4, 130.3, 126.4, 125.0, 124.4, 123.0, 52.44, 52.4.

dimethyl (*E*)-2-(3-(4-nitrophenyl)allylidene)malonate (9j)

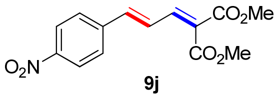

9j

9j (0.2 mmol scale) was synthesized following the *Method D*. The reaction was carried out with 4-(4-nitrophenyl)butanal (0.0360g, 0.2mmol), $Cu(OAc)_2$ (0.0036g, 0.02 mmol, 10 mol%), 4,4'-dimethoxy-2,2'-bipyridine (0.0044g, 0.02 mmol, 10 mol%), TEMPO (0.0315g, 0.2 mmol) and dimethyl malonate (0.0661g, 0.5mmol) in 1, 2-dichlorobenzene (1.0 mL) at 120 °C for 24 h. After concentration and purification by flash chromatography on silica gel (petroleum ether/diethyl ether = 100:12), the product **9j** was obtained in 56% yield.

Physical state: yellow solid;

HRMS (*m/z*): calculated for $C_{14}H_{13}NO_6H^+$ $[M+H]^+$, 292.0816; found, 292.0816;

^{1}H NMR (400 MHz, $CDCl_3$): δ 8.23 (d, *J* = 8.8 Hz, 2H), 7.65 (d, *J* = 8.8 Hz, 2H), 7.55 (d, *J* = 11.6 Hz, 1H), 7.40 (dd, *J* = 15.4 Hz, 11.6 Hz, 1H), 7.08 (d, *J* = 15.4 Hz, 1H), 3.92 (s, 3H), 3.85 (s. 3H);

^{13}C NMR (100 MHz, $CDCl_3$): δ 165.2, 164.7, 148.0, 144.4, 141.6, 141.4, 128.3, 127.1, 126.7, 124.2, 52.6, 52.5.

dimethyl (*E*)-2-(3-(4-(methoxycarbonyl)phenyl)allylidene)malonate (9k)

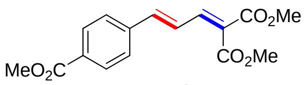

9k

9k (0.2 mmol scale) was synthesized following the *Method D*. The reaction was carried out with methyl 4-(4-oxobutyl)benzoate (0.0384g, 0.2mmol), $Cu(OAc)_2$ (0.0036g, 0.02 mmol, 10 mol%), 4,4'-dimethoxy-2,2'-bipyridine (0.0088g, 0.04 mmol, 20 mol%), TEMPO (0.0315g, 0.2 mmol) and dimethyl malonate (0.0661g, 0.5mmol) in 1, 2-dichlorobenzene (1.0 mL) at 120 °C for 24 h. After concentration and purification by flash chromatography on silica gel (petroleum ether/diethyl ether = 100:10), the product **9k** was obtained in 60% yield.

Physical state: white solid;

HRMS (*m/z*): calculated for $C_{16}H_{16}O_6H^+$ $[M+H]^+$, 305.1020; found, 305.1017;

^{1}H NMR (400 MHz, $CDCl_3$): δ 8.03 (d, *J* = 8.4 Hz, 2H), 7.57-7.54 (m, 3H), 7.35 (dd, *J* = 15.4 Hz, 11.6 Hz, 1H), 7.07 (d, *J* = 15.4 Hz, 1H), 3.93 (s, 3H), 3.92 (s, 3H), 3.84 (s, 3H);

^{13}C NMR (100 MHz, $CDCl_3$): δ 166.4, 165.4, 164.9, 145.2, 143.3, 139.7, 130.8, 130.0, 127.6, 125.4, 52.5, 52.4, 52.2.

dimethyl (*E*)-2-(3-(4-methoxyphenyl)allylidene)malonate (9l)

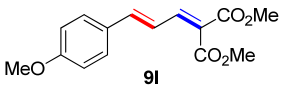

9l

9l (0.2 mmol scale) was synthesized following the *Method D*. The reaction was carried out with 4-(4-methoxyphenyl)butanal (0.0328g, 0.2mmol), $Cu(OAc)_2$ (0.0036g, 0.02 mmol, 10 mol%), 4,4'-dimethoxy-2,2'-bipyridine (0.0088g, 0.04 mmol, 20 mol%), TEMPO (0.0315g, 0.2 mmol) and dimethyl malonate (0.0661g, 0.5mmol) in *tert*-Amyl

alcohol (1.0 mL) at 120 °C for 24 h. After concentration and purification by flash chromatography on silica gel (petroleum ether/diethyl ether = 100:10), the product **9l** was obtained in 82% yield.

Physical state: yellow oil;

HRMS (*m/z*): calculated for $C_{15}H_{16}O_5H^+$ [M+H]$^+$, 277.1071; found, 277.1069;

^{1}H NMR (400 MHz, $CDCl_3$): δ 7.58 (d, *J* = 11.5 Hz, 1H), 7.46 (d, *J* = 8.8 Hz, 2H), 7.17 (dd, *J* = 15.4 Hz, 11.6 Hz, 1H), 7.02 (d, *J* = 15.4 Hz, 1H), 6.90 (d, *J* = 8.8 Hz, 2H), 3.90 (s, 3H), 3.84 (s, 3H), 3.82 (s, 3H);

^{13}C NMR (100 MHz, $CDCl_3$): δ 165.9, 165.4, 161.2, 147.1, 145.2, 129.6, 128.3, 122.4, 121.2, 114.3, 55.4, 52.3, 52.2.

dimethyl (*E*)-2-(3-(4-(trifluoromethyl)phenyl)allylidene)malonate (9m)

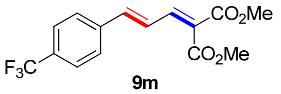

9m

9m (0.2 mmol scale) was synthesized following the *Method D*. The reaction was carried out with methyl 4-(4-(trifluoromethyl)phenyl)butanal (0.0404g, 0.2mmol), $Cu(OAc)_2$ (0.0036g, 0.02 mmol, 10 mol%), 4,4'-dimethoxy-2,2'-bipyridine (0.0088g, 0.04 mmol, 20 mol%), TEMPO (0.0315g, 0.2 mmol) and dimethyl malonate (0.0661g, 0.5mmol) in 1, 2-dichlorobenzene (1.0 mL) at 120 °C for 24 h. After concentration and purification by flash chromatography on silica gel (petroleum ether/diethyl ether = 100:8), the product **9m** was obtained in 54% yield.

Physical state: white solid;

HRMS (*m/z*): calculated for $C_{15}H_{13}F_3O_4H^+$ [M+H]$^+$, 315.0839; found, 315.0835;

^{1}H NMR (400 MHz, $CDCl_3$): δ 7.64-7.59 (m, 4H), 7.56 (d, *J* = 11.6 Hz, 1H), 7.34 (dd, *J* = 15.5 Hz, 11.6 Hz, 1H), 7.07 (d, *J* = 15.5 Hz, 1H), 3.92 (s, 3H), 3.84 (s, 3H);

^{19}F NMR (377 MHz): δ -62.79.

^{13}C NMR (100 MHz, $CDCl_3$): δ 165.4, 164.9, 145.1, 142.7, 138.8 (d, *J* = 1.1 Hz), 131.2 (q, *J* = 32.7 Hz), 127.9, 125.8 (q, *J* = 3.8 Hz), 125.6, 125.5, 123.8 (q, *J* = 272.1Hz), 52.5, 52.4.

dimethyl (*E*)-2-(3-(naphthalen-2-yl)allylidene)malonate (9n)

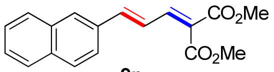

9n

9n (0.2 mmol scale) was synthesized following the *Method D*. The reaction was carried out with 4-(naphthalen-2-yl)butanal (0.0372g, 0.2mmol), $Cu(OAc)_2$ (0.0036g, 0.02 mmol, 10 mol%), 4,4'-dimethoxy-2,2'-bipyridine (0.0088g, 0.04 mmol, 20 mol%), TEMPO (0.0315g, 0.2 mmol) and dimethyl malonate (0.0661g, 0.5mmol) in *tert*-Amyl alcohol (1.0 mL) at 120 ^{o}C for 24 h. After concentration and purification by flash chromatography on silica gel (petroleum ether/diethyl ether = 100:8), the product **9n** was obtained in 67% yield.

Physical state: yellow oil;

HRMS (*m*/*z*): calculated for $C_{18}H_{16}O_4H^+$ $[M+H]^+$, 297.1121; found, 297.1122;

^{1}H NMR (400 MHz, $CDCl_3$): δ 8.12 (d, *J* = 8.3 Hz, 1H), 7.90-7.85 (m, 3H), 7.80 (d, *J* = 7.3 Hz, 1H), 7.72 (d, *J* = 11.7 Hz, 1H), 7.58-7.46 (m, 3H), 7.36 (dd, *J* = 15.2 Hz, 11.7 Hz, 1H), 3.91 (s, 3H), 3.85 (s, 3H);

^{13}C NMR (100 MHz, $CDCl_3$): δ 165.7, 165.1, 146.2, 141.6, 133.7, 132.6, 131.1, 130.3, 128.8, 126.8, 126.1, 125.6, 125.5, 125.0, 124.2, 123.0, 52.4, 52.3.

dimethyl (*E*)-2-(3-(thiophen-2-yl)allylidene)malonate (9o)

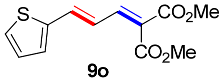

9o

9o (0.2 mmol scale) was synthesized following the *Method D*. The reaction was carried out with 4-(thiophen-2-yl)butanal (0.0280g, 0.2mmol), $Cu(OAc)_2$ (0.0036g, 0.02 mmol, 10 mol%), 4,4'-dimethoxy-2,2'-bipyridine (0.0088g, 0.04 mmol, 20 mol%), TEMPO (0.0315g, 0.2 mmol) and dimethyl malonate (0.0661g, 0.5mmol) in *tert*-Amyl alcohol (1.0 mL) at 120 ^{o}C for 24 h. After concentration and purification by flash chromatography on silica gel (petroleum ether/diethyl ether = 100:8), the product **9o** was obtained in 75% yield.

Physical state: yellow oil;

HRMS (*m*/*z*): calculated for $C_{12}H_{12}SO_4H^+$ $[M+H]^+$, 253.0529; found, 253.0528;

^{1}H NMR (400 MHz, $CDCl_3$): δ 7.52 (d, *J* = 11.5 Hz, 1H), 7.37 (d, *J* = 5.0 Hz, 1H), 7.21-7.16 (m, 2H), 7.09-7.03 (m, 2H), 3.90 (s, 3H), 3.82 (s, 3H);

^{13}C NMR (100 MHz, $CDCl_3$): δ 165.7, 165.2, 146.1, 141.0, 137.4, 130.1, 128.4, 128.1, 123.2, 122.7, 52.4, 52.3.

dimethyl (*E*)-2-(3-(pyridin-3-yl)allylidene)malonate (9p)

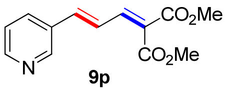

9p

9p (0.2 mmol scale) was synthesized following the *Method D*. The reaction was carried out with 4-(pyridin-3-yl)butanal (0.0270g, 0.2mmol), $Cu(OAc)_2$ (0.0036g, 0.02 mmol, 10 mol%), 4,4'-dimethoxy-2,2'-bipyridine (0.0088g, 0.04 mmol, 20 mol%), TEMPO (0.0315g, 0.2 mmol) and dimethyl malonate (0.0661g, 0.5mmol) in 1, 2-dichlorobenzene (1.0 mL) at 120 °C for 24 h. After concentration and purification by flash chromatography on silica gel (petroleum ether/ethyl acetate = 100:10), the product **9p** was obtained in 48% yield.

Physical state: white solid;

HRMS (*m/z*): calculated for $C_{13}H_{13}NO_4H^+$ $[M+H]^+$, 248.0917; found, 248.0916;

^{1}H NMR (400 MHz, $CDCl_3$): δ 8.71 (s, 1H), 8.57 (d, *J* = 4.2 Hz, 1H), 7.85 (d, *J* = 8.0 Hz, 1H), 7.56 (d, *J* = 11.6 Hz, 1H), 7.36-7.28 (m, 2H), 7.04 (d, *J* = 15.5 Hz, 1H), 3.91 (s, 3H), 3.84 (s, 3H);

^{13}C NMR (100 MHz, $CDCl_3$): δ 165.4, 164.9, 150.5, 149.6, 145.0, 140.7, 133.7, 131.3, 125.3, 125.1, 123.7, 52.5, 52.4.

dimethyl (*E*)-2-(3-(5-methylfuran-2-yl)allylidene)malonate (9q)

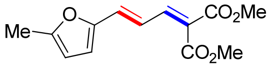

9q

9q (0.2 mmol scale) was synthesized following the *Method D*. The reaction was carried out with 4-(5-methylfuran-2-yl)butanal (0.0276g, 0.2mmol), $Cu(OAc)_2$ (0.0036g, 0.02 mmol, 10 mol%), 4,4'-dimethoxy-2,2'-bipyridine (0.0088g, 0.04 mmol, 20 mol%), TEMPO (0.0315g, 0.2 mmol) and dimethyl malonate (0.0661g, 0.5mmol) in *tert*-Amyl

alcohol (1.0 mL) at 120 °C for 24 h. After concentration and purification by flash chromatography on silica gel (petroleum ether/diethyl ether = 100:10), the product **9q** was obtained in 73% yield.

Physical state: yellow oil;

HRMS (*m*/*z*): calculated for $C_{13}H_{14}O_5H^+$ $[M+H]^+$, 251.0914; found, 251.0913;

^{1}H NMR (400 MHz, $CDCl_3$): δ 7.50 (d, *J* = 12.1 Hz, 1H), 7.06 (dd, *J* = 15.1 Hz, 12.1 Hz, 1H), 6.74 (d, *J* = 15.2 Hz, 1H), 6.48 (d, *J* = 3.2 Hz, 1H), 6.08-6.07 (m, 1H), 3.90 (s, 3H), 3.81 (s, 3H), 2.35 (s, 3H);

^{13}C NMR (100 MHz, $CDCl_3$): δ 165.9, 165.3, 155.7, 150.5, 146.4, 131.3, 122.4, 119.8, 116.0, 109.1, 52.3, 14.0.

dimethyl (*E*)-2-(hex-2-en-1-ylidene)malonate(9r)

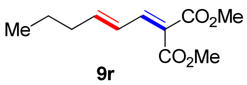

9r

9r (0.2 mmol scale) was synthesized following the *Method D* with slight modifications. The reaction was carried out with hexanal (0.0200g, 0.2mmol), $Cu(OAc)_2$ (0.0036g, 0.02 mmol, 10 mol%), 4,4'-dimethoxy-2,2'-bipyridine (0.0088g, 0.04 mmol, 20 mol%), TEMPO (0.0315g, 0.2 mmol), dimethyl malonate (0.0661g, 0.5mmol), TsOH (0.0035g, 0.02 mmol, 10 mol%) and piperidine (2 μL, 0.02 mmol, 10 mol%) in 1, 2-dichlorobenzene (1.0 mL) at 100 °C for 24 h. After concentration and purification by flash chromatography on silica gel (petroleum ether/diethyl ether = 100:10), the product **9r** was obtained in 66% yield.

Physical state: colorless oil;

HRMS (*m*/*z*): calculated for $C_{11}H_{16}O_4H^+$ $[M+H]^+$, 213.1121; found, 213.1121;

^{1}H NMR (400 MHz, $CDCl_3$): δ 7.38 (d, *J* = 11.5 Hz, 1H), 6.57-6.49 (m, 1H), 6.38-6.31 (m, 1H), 3.85 (s, 3H), 3.79 (s, 3H), 2.24-2.18 (m, 2H), 1.51-1.43 (m, 2H), 0.93 (t, *J* = 7.4 Hz, 3H);

^{13}C NMR (100 MHz, $CDCl_3$): δ 165.8, 165.2, 150.3, 146.5, 125.9, 122.6, 52.3, 52.2, 35.3, 21.7, 13.7.

dimethyl 2-(3-methylbut-2-en-1-ylidene)malonate (9s)

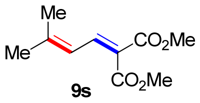

9s (0.2 mmol scale) was synthesized following the *Method D* with slight modifications. The reaction was carried out with 3-methylbutanal (0.0172g, 0.2mmol), $Cu(OAc)_2$ (0.0036g, 0.02 mmol, 10 mol%), 4,4'-dimethoxy-2,2'-bipyridine (0.0088g, 0.04 mmol, 20 mol%), TEMPO (0.0315g, 0.2 mmol), TsOH (0.0035g, 0.02 mmol, 10 mol%) , piperidine (2 μL, 0.02 mmol, 10 mol%) and dimethyl malonate (0.0661g, 0.5mmol) in *tert*-Amyl alcohol (1.0 mL) at 100 °C for 24 h. After concentration and purification by flash chromatography on silica gel (petroleum ether/diethyl ether = 100:10), the product **9s** was obtained in 51% yield.

Physical state: colorless oil;

HRMS (*m/z*): calculated for $C_{10}H_{14}O_4H^+$ $[M+H]^+$, 199.0965; found, 199.0966;

^{1}H NMR (400 MHz, $CDCl_3$): δ 7.69 (d, J = 12.3 Hz, 1H), 6.30-6.26 (m, 1H), 3.85 (s, 3H), 3.80 (s, 3H), 1.96 (s, 3H), 1.94 (s, 3H);

^{13}C NMR (100 MHz, $CDCl_3$): δ 166.3, 165.5, 152.4, 141.2, 122.0, 120.9, 52.3, 52.2, 27.2, 19.1.

dimethyl 2-((2*E*,4*E*)-5-phenylpenta-2,4-dien-1-ylidene)malonate (9t)

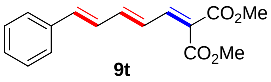

9t (0.2 mmol scale) was synthesized following the *Method D*. The reaction was carried out with 5-phenylpentanal (0.0324g, 0.2mmol), $Cu(OAc)_2$ (0.0036g, 0.02 mmol, 10 mol%), 4,4'-dimethoxy-2,2'-bipyridine (0.0088g, 0.04 mmol, 20 mol%), TEMPO (0.0630g, 0.4 mmol) and dimethyl malonate (0.0661g, 0.5mmol) in *tert*-Amyl alcohol (1.0 mL) at 120 °C for 72 h. After concentration and purification by flash chromatography on silica gel (petroleum ether/diethyl ether = 100:10), the product **9t** was obtained in 80% yield.

Physical state: yellow oil;

HRMS (*m/z*): calculated for $C_{16}H_{16}O_4H^+$ $[M+H]^+$, 273.1121; found, 273.1120;

^{1}H NMR (400 MHz, $CDCl_3$): δ 7.51-7.44 (m, 3H), 7.37-7.28 (m, 3H), 6.97-6.78 (m, 4H), 3.88 (s, 3H), 3.81 (s, 3H);

^{13}C NMR (100 MHz, $CDCl_3$): δ 165.7, 165.2, 145.9, 145.4, 139.1, 136.2, 128.9, 128.8, 127.8, 127.2, 127.1, 123.3, 52.3, 52.2.

dimethyl 2-((2*E*,4*E*,6*E*)-7-phenylhepta-2,4,6-trien-1-ylidene) malonate (9u)

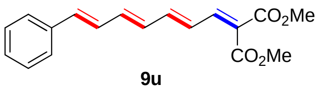

9u

9u (0.2 mmol scale) was synthesized following the *Method D* with slight modifications. The reaction was carried out with 7-phenylheptanal (0.0380g, 0.2mmol), $Cu(OAc)_2$ (0.0108g, 0.06 mmol, 30 mol%), 4,4'-dimethoxy-2,2'-bipyridine (0.0158g, 0.072 mmol, 36 mol%), TEMPO (0.1260g, 0.8 mmol) and dimethyl malonate (0.0661g, 0.5mmol) in *tert*-Amyl alcohol (1.0 mL) at 120 ^{o}C for 24 h. After concentration and purification by flash chromatography on silica gel (petroleum ether/diethyl ether = 100:10), the product **9u** was obtained in 35% yield.

Physical state: yellow solid;

HRMS (*m*/*z*): calculated for $C_{18}H_{18}O_4H^+$ $[M+H]^+$, 299.1278; found, 299.1277;

^{1}H NMR (400 MHz, $CDCl_3$): δ 7.49-7.43 (m, 3H), 7.36-7.32 (m, 2H), 7.29-7.27 (m, 1H), 6.91-6.84 (m, 1H), 6.80-6.64 (m, 4H), 6.51-6.45 (m, 1H), 3.87 (s, 3H), 3.81 (s, 3H);

^{13}C NMR (100 MHz, $CDCl_3$): δ 165.8, 165.2, 146.0, 145.3, 139.8, 136.7, 136.5, 131.9, 128.7, 128.4, 128.3, 127.0, 126.8, 123.0, 52.3, 52.2.

dimethyl 2-benzylidenemalonate (9v)

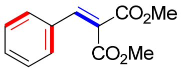

9v

9v (0.2 mmol scale) was synthesized following the *Method D* with slight modifications. The reaction was carried out with cyclohex-3-enecarbaldehyde (0.0220g, 0.2mmol), $Cu(OAc)_2$ (0.0036g, 0.02 mmol, 10 mol%), 4,4'-dimethoxy-2,2'-bipyridine (0.0088g, 0.04 mmol, 20 mol%), TEMPO (0.0630g, 0.4 mmol), TsOH (0.0035g, 0.02 mmol, 10

mol%) , piperidine (2 μL, 0.02 mmol, 10 mol%) and dimethyl malonate (0.0661g, 0.5mmol) in *tert*-Amyl alcohol (1.0 mL) at 100 °C for 48 h. After concentration and purification by flash chromatography on silica gel (petroleum ether/diethyl ether = 100:10), the product **9v** was obtained in 60% yield.

Physical state: colorless oil;

HRMS (*m*/*z*): calculated for $C_{12}H_{12}O_4H^+$ $[M+H]^+$, 221.0808; found, 221.0809;

^{1}H NMR (400 MHz, $CDCl_3$): δ 7.78 (s, 1H), 7.44-7.7.36 (m, 5H), 3.85 (s, 6H);

^{13}C NMR (100 MHz, $CDCl_3$): δ 167.1, 164.4, 142.9, 132.6, 130.6, 129.3, 128.8, 125.3, 52.7, 52.6.

quinolone (11a)

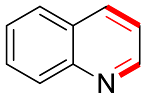

11a

11a (0.5 mmol scale) was synthesized following the *Method E*. The reaction was carried out with 1,2,3,4-tetrahydroquinoline (0.0666g, 0.5 mmol), $Cu(OAc)_2$ (0.0090g, 0.05 mmol, 10 mol%), 2,2'-bipyridine (0.0078, 0.05 mmol, 10 mol%), LiOAc (0.0330g, 0.5 mmol) and TEMPO (0.1563g, 1.0 mmol) in 1, 2-dichlorobenzene (1.0 mL). The reaction mixture was stirred at 120 °C for 24 h. After concentration and purification by flash chromatography on silica gel (petroleum ether/ethyl acetate = 100:10), the product **11a** was obtained in 96% yield.

Physical state: colorless oil;

^{1}H NMR (400 MHz, $CDCl_3$): δ 8.90 (dd, J = 4.2 Hz, 1.7 Hz, 1H), 8.13-8.10 (m, 2H), 7.78 (dd, J = 8.2 Hz, 1.0 Hz, 1H), 7.71-7.68 (m, 1H), 7.54-7.50 (m, 1H), 7.35 (dd, J = 8.3 Hz, 4.2 Hz, 1H);

^{13}C NMR (100 MHz, $CDCl_3$): δ 150.2, 148.1, 135.9, 129.3, 129.2, 128.1, 127.6, 126.4, 120.9.

6-bromoquinoline (11b)

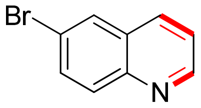

11b

11b (0.5 mmol scale) was synthesized following the *Method E*. The reaction was carried out with 6-bromo-1,2,3,4-tetrahydroquinoline (0.1061g, 0.5 mmol), $Cu(OAc)_2$ (0.0090g, 0.05 mmol, 10 mol%), 2,2'-bipyridine (0.0078, 0.05 mmol, 10 mol%), LiOAc (0.0330g, 0.5 mmol) and TEMPO (0.1563g, 1.0 mmol) in 1, 2-dichlorobenzene (1.0 mL). The reaction mixture was stirred at 120 °C for 24 h. After concentration and purification by flash chromatography on silica gel (petroleum ether/ethyl acetate = 100:10), the product **11b** was obtained in 95% yield.

Physical state: slight yellow oil;

^{1}H NMR (400 MHz, $CDCl_3$): δ 8.89 (dd, J = 4.2 Hz, 1.7 Hz, 1H), 8.01-7.93 (m, 2H), 7.90 (d, J = 2.2 Hz, 1H), 7.73 (dd, J = 9.0 Hz, 2.2 Hz, 1H), 7.36 (dd, J = 8.3 Hz, 4.2 Hz, 1H);

^{13}C NMR (100 MHz, $CDCl_3$): δ 150.5, 146.5, 134.8, 132.7, 131.0, 129.6, 129.1, 121.6, 120.2.

quinolin-7-amine (11c)

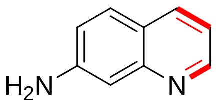

11c

11c (0.5 mmol scale) was synthesized following the *Method E*. The reaction was carried out with 7-amino-1,2,3,4-tetrahydroquinoline (0.0741g, 0.5 mmol), $Cu(OAc)_2$ (0.0090g, 0.05 mmol, 10 mol%), 2,2'-bipyridine (0.0078, 0.05 mmol, 10 mol%), LiOAc (0.0330g, 0.5 mmol) and TEMPO (0.1563g, 1.0 mmol) in 1, 2-dichlorobenzene (1.0 mL). The reaction mixture was stirred at 120 °C for 24 h. After concentration and purification by flash chromatography on silica gel (petroleum ether/ethyl acetate = 100:30), the product **11c** was obtained in 86% yield.

Physical state: slight yellow solid;

^{1}**H NMR** (400 MHz, $CDCl_3$): δ 8.74 (dd, J = 4.3 Hz, 1.6 Hz, 1H), 7.94 (dd, J = 8.1 Hz, 1.1 Hz, 1H), 7.58 (d, J = 8.7 Hz, 1H), 7.21 (d, J = 2.2 Hz, 1H), 7.11 (dd, J = 8.1 Hz, 4.3 Hz, 1H), 6.96 (dd, J = 8.1 Hz, 4.3 Hz, 1H), 4.20 (s, 2H);

^{13}**C NMR** (100 MHz, $CDCl_3$): δ 150.6, 149.9, 147.7, 135.6, 128.8, 122.1, 118.6, 117.6, 109.1.

6-methylquinoline (11d)

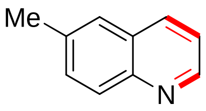

11d

11d (0.5 mmol scale) was synthesized following the *Method E*. The reaction was carried out with 6-methyl-1,2,3,4-tetrahydroquinoline (0.0736g, 0.5 mmol), $Cu(OAc)_2$ (0.0090g, 0.05 mmol, 10 mol%), 2,2'-bipyridine (0.0078, 0.05 mmol, 10 mol%), LiOAc (0.0330g, 0.5 mmol) and TEMPO (0.1563g, 1.0 mmol) in 1, 2-dichlorobenzene (1.0 mL). The reaction mixture was stirred at 120 °C for 24 h. After concentration and purification by flash chromatography on silica gel (petroleum ether/ethyl acetate = 100:10), the product **11d** was obtained in 97% yield.

Physical state: colorless oil;

^{1}**H NMR** (400 MHz, $CDCl_3$): δ 8.81 (dd, J = 4.2 Hz, 1.6 Hz, 1H), 8.01-7.98 (m, 2H), 7.50-7.49 (m, 2H), 7.29 (dd, J = 8.3 Hz, 4.2 Hz, 1H), 2.48 (s, 3H);

^{13}**C NMR** (100 MHz, $CDCl_3$): δ 149.2, 146.6, 136.1, 135.2, 131.5, 128.8, 128.1, 126.4, 120.8, 21.3.

7-nitroquinoline (11e)

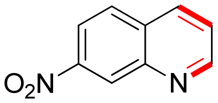

11e

11e (0.5 mmol scale) was synthesized following the *Method E*. The reaction was carried out with 7-nitro-1,2,3,4-tetrahydroquinoline (0.0891g, 0.5 mmol), $Cu(OAc)_2$

(0.0090g, 0.05 mmol, 10 mol%), 2,2'-bipyridine (0.0078, 0.05 mmol, 10 mol%), LiOAc (0.0330g, 0.5 mmol) and TEMPO (0.1563g, 1.0 mmol) in 1, 2-dichlorobenzene (1.0 mL). The reaction mixture was stirred at 120 °C for 24 h. After concentration and purification by flash chromatography on silica gel (petroleum ether/ethyl acetate = 100:15), the product **11e** was obtained in 98% yield.

Physical state: yellow solid;

^{1}H NMR (400 MHz, $CDCl_3$): δ 9.09-9.08 (m, 1H), 8.95 (s, 1H), 8.31-8.29 (m, 2H), 8.00 (d, *J* = 9.0 Hz, 1H), 7.62 (dd, *J* = 8.4 Hz, 4.2 Hz, 1H);

^{13}C NMR (100 MHz, $CDCl_3$): δ 152.6, 147.9, 147.0, 135.8, 131.3, 129.4, 125.7, 123.9, 119.9.

7-(trifluoromethyl)quinolone (11f)

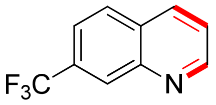

11f

11f (0.5 mmol scale) was synthesized following the *Method E* with slight modifications. The reaction was carried out with 7-(trifluoromethyl)-1,2,3,4-tetrahydroquinoline (0.1006g, 0.5 mmol), $Cu(OAc)_2$ (0.0090g, 0.05 mmol, 10 mol%), 2,2'-bipyridine (0.0078, 0.05 mmol, 10 mol%), TsOH (0.0086g, 0.05mmol) and TEMPO (0.1563g, 1.0 mmol) in 1, 2-dichlorobenzene (1.0 mL). The reaction mixture was stirred at 120 °C for 24 h. After concentration and purification by flash chromatography on silica gel (petroleum ether/ethyl acetate = 100:10), the product **11f** was obtained in 91% yield.

Physical state: white solid;

^{1}H NMR (400 MHz, $CDCl_3$): δ 9.03 (dd, *J* = 4.2 Hz, 1.6Hz, 1H), 8.43 (s, 1H), 8.23 (d, *J* = 8.2 Hz, 1H), 7.95 (d, *J* = 8.5 Hz, 1H), 7.73 (dd, *J* = 8.6 Hz, 1.7 Hz, 1H), 7.53 (dd, *J* = 8.4 Hz, 4.2 Hz, 1H);

^{19}F NMR (377 MHz): δ -62.66;

^{13}C NMR (100 MHz, $CDCl_3$): δ 151.7, 147.2, 136.0, 131.3 (q, *J* = 32.6 Hz), 129.7, 129.0, 127.3 (q, *J* = 4.4 Hz), 123.9 (q, *J* = 272.7 Hz), 122.9, 122.2 (q, *J* = 3.3 Hz).

6-fluoroquinoline (11g)

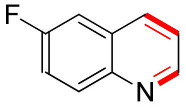

11g

11g (0.5 mmol scale) was synthesized following the *Method E*. The reaction was carried out with 6-fluoro-1,2,3,4-tetrahydroquinoline (0.0756g, 0.5 mmol), $Cu(OAc)_2$ (0.0090g, 0.05 mmol, 10 mol%), 2,2'-bipyridine (0.0078, 0.05 mmol, 10 mol%), LiOAc (0.0330g, 0.5 mmol) and TEMPO (0.1563g, 1.0 mmol) in 1, 2-dichlorobenzene (1.0 mL). The reaction mixture was stirred at 120 °C for 24 h. After concentration and purification by flash chromatography on silica gel (petroleum ether/ethyl acetate = 100:10), the product **11g** was obtained in 95% yield.

Physical state: colorless oil;

^{1}H NMR (400 MHz, $CDCl_3$): δ 8.86 (dd, J = 4.2 Hz, 1.6Hz, 1H), 8.11-8.03 (m, 2H), 7.49-7.44 (m, 1H), 7.40-7.35 (m, 2H);

^{19}F NMR (377 MHz): δ -113.26;

^{13}C NMR (100 MHz, $CDCl_3$): δ 160.1 (d, J = 248.1Hz), 149.5 (d, J = 2.7 Hz), 145.2, 135.2 (d, J = 5.3 Hz), 131.8 (d, J = 9.1 Hz), 128.7 (d, J = 10.1 Hz), 121.5, 119.5 (d, J = 25.9 Hz), 110.5 (d, J = 21.4Hz).

6-methoxyquinoline (11h)

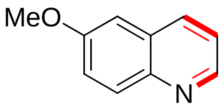

11h

11h (0.5 mmol scale) was synthesized following the *Method E* with slight modifications. The reaction was carried out with

6-methoxy-1,2,3,4-tetrahydroquinoline (0.0816g, 0.5 mmol), $Cu(OAc)_2$ (0.0090g, 0.05 mmol, 10 mol%), 2,2'-bipyridine (0.0078, 0.05 mmol, 10 mol%), TsOH (0.0086g, 0.05mmol) and TEMPO (0.1563g, 1.0 mmol) in 1, 2-dichlorobenzene (1.0 mL). The reaction mixture was stirred at 120 °C for 24 h. After concentration and purification by flash chromatography on silica gel (petroleum ether/ethyl acetate = 100:15), the product **11h** was obtained in 96% yield.

Physical state: colorless oil;

^{1}H NMR (400 MHz, $CDCl_3$): δ 8.70 (dd, *J* = 4.2 Hz, 2.0Hz, 1H), 7.97-7.92 (m, 2H), 7.32-7.29 (m, 1H), 7.26-7.22 (m, 1H), 6.95-6.94 (m, 1H), 3.82 (s, 3H);

^{13}C NMR (100 MHz, $CDCl_3$): δ 157.4, 147.6, 144.1, 134.5, 130.5, 129.0, 122.0, 121.1, 104.8, 55.2.

1H-indole (11i)

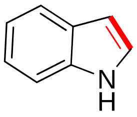

11i

(0.5 mmol scale) **11i** was synthesized following the *Method E* with slight modifications. The reaction was carried out with indoline (0.0596g, 0.5 mmol), $Cu(OAc)_2$ (0.0090g, 0.05 mmol, 10 mol%), 4,4'-dimethoxy-2,2'-bipyridine (0.0220g, 0.10 mmol, 20 mol%) and TEMPO (0.0780g, 0.5 mmol) in *tert*-Amyl alcohol (2.0 mL). The reaction mixture was stirred at 100 °C for 24 h. After concentration and purification by flash chromatography on silica gel (petroleum ether/ ethyl acetate = 100:5), the product **11i** was obtained in 96% yield.

Physical state: white solid;

^{1}H NMR (400 MHz, $CDCl_3$): δ 7.92 (s, 1H), 7.64 (d, *J* = 7.9 Hz, 1H), 7.32-7.30 (m, 1H), 7.20-7.16 (m, 1H), 7.14-7.09 (m, 2H), 6.54-6.52 (m, 1H);

^{13}C NMR (100 MHz, $CDCl_3$): δ 135.7, 127.7, 124.1, 121.9, 120.7, 119.7, 111.0, 102.5.

methyl 1H-indole-5-carboxylate (11j)

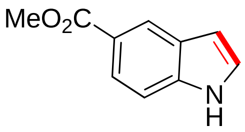

11j

(0.5 mmol scale) **11j** was synthesized following the *Method E* with slight modifications. The reaction was carried out with methyl indoline-5-carboxylate (0.0886g, 0.5 mmol), $Cu(OAc)_2$ (0.0090g, 0.05 mmol, 10 mol%), 4,4'-dimethoxy-2,2'-bipyridine (0.0220g, 0.10 mmol, 20 mol%) and TEMPO (0.0780g, 0.5 mmol) in *tert*-Amyl alcohol (2.0 mL). The reaction mixture was stirred at 100 ^{o}C for 24 h. After concentration and purification by flash chromatography on silica gel (petroleum ether/ ethyl acetate = 100:10), the product **11j** was obtained in 94% yield.

Physical state: white solid;

^{1}**H NMR** (400 MHz, $CDCl_3$): δ 8.91 (s, 1H), 8.44 (t, J = 0.7 Hz, 1H), 7.90 (dd, J = 8.6 Hz, 1.6 Hz, 1H), 7.36 (d, J = 8.6 Hz, 1H), 7.22-7.21 (m, 1H). 6.62-6.60 (m, 1H), 3.92 (s, 3H);

^{13}**C NMR** (100 MHz, $CDCl_3$): δ 168.5, 138.4, 127.4, 125.7, 123.7, 123.1, 121.5, 110.8, 103.7, 51.9.

6-chloro-1H-indole (11k)

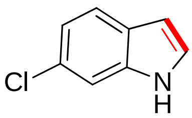

11k

(0.5 mmol scale) **11k** was synthesized following the *Method E* with slight modifications. The reaction was carried out with 6-chloro-2,3-dihydro-1H-indole hydrochloride (0.0950g, 0.5 mmol), $Cu(OAc)_2$ (0.0090g, 0.05 mmol, 10 mol%), 4,4'-dimethoxy-2,2'-bipyridine (0.0220g, 0.10 mmol, 20 mol%) and TEMPO (0.0780g, 0.5 mmol) in *tert*-Amyl alcohol (2.0 mL). The reaction mixture was stirred at 100 ^{o}C for 24 h. After concentration and purification by flash chromatography on silica gel (petroleum ether/ ethyl acetate = 100:5), the product **11k** was obtained in 90% yield.

Physical state: white solid;

^{1}**H NMR** (400 MHz, $CDCl_3$): δ 7.91 (s, 1H), 7.52 (d, *J* = 8.4 Hz, 1H), 7.27 (d, *J* = 0.6 Hz, 1H), 7.09-7.07 (m, 2H), 6.49 (s, 1H);

^{13}**C NMR** (100 MHz, $CDCl_3$): δ 136.0, 127.7, 126.3, 124.9, 121.5, 120.5, 110.9, 102.6.

5-bromo-1H-indole (11l)

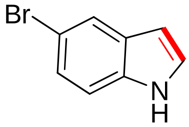

11l

(0.5 mmol scale) **11l** was synthesized following the *Method E* with slight modifications. The reaction was carried out with 5-bromoindoline (0.0990g, 0.5 mmol), $Cu(OAc)_2$ (0.0090g, 0.05 mmol, 10 mol%), 4,4'-dimethoxy-2,2'-bipyridine (0.0220g, 0.10 mmol, 20 mol%) and TEMPO (0.0780g, 0.5 mmol) in *tert*-Amyl alcohol (2.0 mL). The reaction mixture was stirred at 100 °C for 24 h. After concentration and purification by flash chromatography on silica gel (petroleum ether/ ethyl acetate = 100:5), the product **11l** was obtained in 75% yield.

Physical state: white solid;

^{1}**H NMR** (400 MHz, $CDCl_3$): δ 8.04 (s, 1H), 7.75 (d, *J* = 1.8 Hz, 1H), 7.25 (dd, *J* = 8.6 Hz, 1.9 Hz, 1H), 7.18 (d, *J* = 8.6 Hz, 1H), 7.13 (t, *J* = 2.8 Hz, 1H), 6.47-6.45 (m, 1H);

^{13}**C NMR** (100 MHz, $CDCl_3$): δ 134.3, 129.5, 125.4, 124.7, 123.1, 112.9, 112.4, 102.2.

(3*E*,5*E*,7*E*)-3-methyl-8-phenylocta-3,5,7-trien-2-one

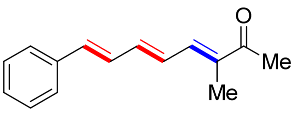

lignarenone B

Synthesis of **lignarenone B** : (1)The reaction was carried out with 5-phenyl-1-pentanol (1.3140g, 8.0 mmol), $Cu(OAc)_2$ (0.1440g, 0.8 mmol, 10 mol%), 4,4'-dimethoxy-2,2'-bipyridine (0.1760, 0.8 mmol, 10 mol%), TEMPO (3.7800g, 24.0 mmol), TsOH (0.1400g, 0.8 mmol, 10%). The reaction mixture was stirred at 120 °C in t-Amyl alcohol (60.0 mL) for 48 h. After concentration and purification by flash chromatography on silica gel (petroleum ether/diethyl ether = 100:6), the product **6a**

was obtained in 53% yield (0.670g); (2) the compound **6a** (0.6328g, 4.0 mmol) was added to the solution of 3-(triphenylphosphoranylidene)butan-2-one (1.9943g, 6.0 mmol) in dry CH_2Cl_2 (20 mL) at 80 °C for 30 h. After concentration and purification by flash chromatography on silica gel (petroleum ether/diethyl ether = 100:6), the product **lignarenone B** was obtained in 41% overall yield (0.701g).

Physical state: yellow solid;

HRMS (*m/z*): calculated for $C_{15}H_{16}OH^+$ $[M+H]^+$, 213.1274; found, 213.1274;

^{1}H NMR (400 MHz, $CDCl_3$): δ 7.45 (d, *J* = 7.4 Hz, 2H), 7.36-7.33 (m, 2H), 7.29-7.26 (m, 1H), 7.125 (d, *J* = 10.0 Hz, 1H), 6.95 (dd, *J* = 15.6, 9.6 Hz, 1H), 6.78-6.65 (m, 3H), 2.37 (s, 3H), 1.94 (s, 3H);

^{13}C NMR (100 MHz, $CDCl_3$): δ 199.4, 140.0, 139.1, 136.6, 136.3, 136.2, 128.7, 128.6, 128.4, 128.3, 126.7, 25.6, 11.6.

(3*E*,5*E*,7*E*,9*E*)-10-phenyldeca-3,5,7,9-tetraen-2-one

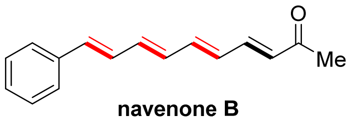

navenone B

Synthesis **navenone B** : (1)The reaction was carried out with 7-phenyl-1-pentanol (1.5384g, 8.0 mmol), $Cu(OAc)_2$ (0.1440g, 0.8 mmol, 10 mol%), 4,4'-dimethoxy-2,2'-bipyridine (0.1760, 0.8 mmol, 10 mol%), TEMPO (5.0400g, 32.0 mmol), TsOH (0.1400g, 0.8 mmol, 10%). The reaction mixture was stirred at 120 °C in t-Amyl alcohol (60.0 mL) for 48 h. After concentration and purification by flash chromatography on silica gel (petroleum ether/diethyl ether = 100:6), the product **6b** was obtained in 26% yield (0.380g); (2) the compound **6b** (0.3640g, 2.0 mmol) was added to the solution of 1-triphenylphosphoranylidene -2-propanone (0.9550g, 3.0 mmol) in dry CH_2Cl_2 (20 mL) at 80 °C for 30 h. After concentration and purification by flash chromatography on silica gel (petroleum ether/diethyl ether = 100:6), the product **navenone B** was obtained in 21% overall yield (0.370g).

Physical state: yellow solid;

HRMS (*m/z*): calculated for $C_{16}H_{16}OH^+$ $[M+H]^+$, 225.1274; found, 225.1274;

^{1}H NMR (400 MHz, $CDCl_3$): δ 7.42 (d, *J* = 7.4 Hz, 2H), 7.33 (t, *J* = 7.4 Hz, 2H), 7.27-7.25 (m, 1H), 7.18 (dd, *J* = 15.4, 11.2 Hz, 1H), 6.87 (dd, *J* = 15.5, 10.7 Hz, 1H), 6.72-6.65 (m, 2H), 6.58 (dd, *J* = 14.7, 10.8 Hz, 1H), 6.46-6.33 (m, 2H), 6.15 (d, *J* = 15.4 Hz, 1H), 2.28 (s, 3H);

^{13}C NMR (100 MHz, $CDCl_3$): δ 198.3, 143.2, 141.5, 137.7, 136.8, 135.3, 132.1, 130.5, 129.8, 128.7, 128.4, 128.1, 126.6, 27.4.

Supplementary References

(1) Mori, A. *et al.* Pd/C-catalyzed chemoselective hydrogenation in the presence of diphenylsulfide. *Org. Lett.* **8,** 3279-3281 (2006).

(2) Ferreira, I. M., Meira, E. B., Rosset, I. G. & Porto, A. L. M. Chemoselective biohydrogenation of α,β- and α,β,γ,δ-unsaturated ketones by the marine-derived fungus *Penicillium citrinum* CBMAI 1186 in a biphasic system. *Journal of Molecular Catalysis B: Enzymatic* **115,** 59–65 (2015)

(3) Jun, C.-H., Huh, C.-W. & Na, S.-J. Direct synthesis of ketones from primary alcohols and 1-alkenes. *Angew. Chem. Int. Ed.* **37,** 145-147 (1998).

(4) Mccarthy, T. D. & Naylor, A. Heterocyclic compounds and methods for their use. *Patent: WO2013/102242* A1 (2013).

(5) Wang, Z. *et al.* CF_3CO_2ZnEt-mediated highly regioselective rearrangement of bromohydrins to aldehydes. *Tetrahedron Letters* **52,** 5968-5971 (2011).

(6) Choi, S. E. & Pflum, M. K. H. The structural requirements of histone deacetylase inhibitors: suberoylanilide hydroxamic acid analogs modified at the C6 position. *Bioorg. Med. Chem. Lett.* **22,** 7084-7086 (2012).

(7) Lesimple, P. & Bigg, D. C. H. Aluminum chloride mediated aminolysis of lactones: a general method for the preparation of ω-hydroxyalkylamides. *Synthesis*, **4,** 306-308 (1991).

(8) Johns, B. A. *et al.* HIV integrase inhibitors. *Patent: US2015/225399* A1 (2015).

(9) Fang, F., Li, Y. & Tian, S.-K. Stereoselective olefination of *N*-sulfonyl imines with stabilized phosphonium ylides for the synthesis of electron-deficient alkenes. *Eur, J. Org. Chem.* **2011,** 1084-1091 (2011)

(10) Van Humbeck, J.F., Simonovich, S. P., Knowles, R. R. & MacMillan, D. W. C. Concerning the mechanism of the $FeCl_3$-catalyzed α-oxyamination of aldehydes: evidence for a non-SOMO activation pathway. *J. Am. Chem. Soc.* **132,** 10012-10014 (2010).

(11) Kim, H. Y. & Oh, K. 1,3-Dienones and 2H-Pyran-2-ones from Soft α-Vinyl Enolization of β-Chlorovinyl Ketones: Defined Roles of Brönsted and Lewis Base. *Org. Lett.* **17,** 6254-6257 (2015).
