# Supplementary material for: Dehydrogenative desaturation-relay via formation of multicenter-stabilized radical intermediates
Source: Nat Commun. 2017 Dec 22;8:2273. doi: 10.1038/s41467-017-02381-8 (PMC5741636; doi:10.1038/s41467-017-02381-8)
Supplement: Supplementary file 2 — Peer Review File [file 41467_2017_2381_MOESM2_ESM.pdf]

Reviewer #1 (Remarks to the Author):

This is an impressive work from Su and coworkers outlining a protocol for the successive dehydrogenation of various saturated organic substrates. While sequential dehydrogenation using other systems is known (e.g. ketone dehydrogenation by palladium catalyst), this work clearly demonstrated the strength of the Cu(II)/TEMPO system. The key to the successive dehydrogenation and the importance of this work lies in the second and following steps: the activation of significantly less acidic gamma- and farther C-H bond, which the Cu(II)/TEMPO system is shown to be capable of. From synthetic viewpoints, the successive dehydrogenation of long-chain linear alcohol is of great potential for the synthesis of important natural products or bioactive compounds. Thus, it is recommended that this manuscript publish in Nat. Communications after the following minor issues are addressed.
(a) The mechanistic proposal part needs to be further elaborated (Figure 1C). As the acidity of the gamma-C-H bond in enone is much less than the alpha-C-H bond of ketone (it would be better for the authors to display some data to make this clear), the transformation of B to C can actually be viewed as allylic C-H activation. While such a process is well known for palladium complex (e.g. palladium acetate), examples using copper catalyst are rare. It would be better if the authors could offer some literature supports and insights into this step. For example, is it proceeding through simple deprotonation or a radical process? The authors are also suggested to show the regeneration of Cu catalyst in the scheme for clarity. Also, the authors proposed that copper salt could also act as Lewis acid to polarize the carbonyl. This point also needs to be clarified. Is the deprotonation process involving two Cu species, one polarizing the carbonyl and the other deprotonate the C-H bond? If only one Cu catalyst is involved, what's the likely transition state for the step?
(b) It is known that palladium-catalyzed dehydrogenation can access phenol from cyclohexanone. For the substrate scope of this Cu/TEMPO system, does cyclic ketone work? Also, what's role of TsOH in the dehydrogenation of alcohols, and LiOAc in the dehydrogenation of N-heterocycles. The authors need to explain these in the manuscript.
(c) In figure 1b, it is confusing to use the structures of the right corner at the bottom to indicate quinoline and indole. It is simply better to show structures of both of them.
(d) The optimization part needs to be expanded (figure 2). For example, it would be better to show more information about the screening, such as the byproduct (if any) of the reaction with low yields. Does the electronic property of the ligand affect the reaction outcome (i.e. phenanthroline with electron-donating or -withdrawing substituent)?

Reviewer #2 (Remarks to the Author):

Su and co-workers describe a desaturation reaction this is of broad interest to the readership of Nature Communications. The copper-catalyzed process allows for oxidation of numerous types of organic substrates. The main innovation to develop this method was identifying the appropriate bidentate ligand to have high levels of activity.

The authors determine that the products are more readily oxidized than the starting materials due to the greater level of stabilization afforded due to increased conjugation of the intermediate radical. The products are highly conjugated materials including dienes, trienes, and aromatic heterocycles. As an added bonus, the authors highlight that desaturation can accompany an aldol reaction.

The ease of operation is one of the nice features of this chemistry and I suspect these reaction conditions will be used for numerous other transformations both by the PI and by others working in this area. Publication is suggested.

Reviewer #3 (Remarks to the Author):

In this manuscript Su and coworkers report a copper-catalyzed method for the multiple/sequential dehydrogenative desaturation of ketones, alcohols, and N-heterocyclic compounds. The reactivity reported is unusual and important and it fills a gap in current synthetic methodology. The work would be strengthened if a clearer understanding of the scope and mechanistic distinctions (such as the differences in product distributions between this system and related late-metal systems and Cu/TEMPO systems) were made clearer. A deeper understanding of these features would better enable the utilization of this new and interesting method. With additional scope and mechanistic understanding as described below this work may be suitable for Nature Commun.

Scope & Synthetic Utility

1. In general, the synthetic utility would be bolstered by a better understanding of the factors that enable this newly discovered dehydrogenative-relay type oxidation.

One premise of the system is that "the polarizing effect that carbonyls exert" with the added influence of a coordinating Lewis acid, in this case the copper salt, combined with "the choice of proper ligand would lead to the optimal metal catalyst for the successive dehydrogenation." While this approach is reasonable, it begs the question: How is this catalyst system different from those used for related alcohol oxidation reactions? Both systems employ a mixture of Cu salt, TEMPO and diimine ligand, and it's unclear what features of this new system enable the successive dehydrogenation of alcohols, while very similar conditions enable the selective oxidation of the alcohol without additional dehydrogenation steps? In addition, the ligand control data shown in Figure 2 suggests that ligand is not key to achieving this reactivity, as 29% yield of the product is formed in the absence of ligand.

The authors highlight the ability of this system to enable the successive dehydrogenation of alcohols following their oxidation to aldehydes, and note that the system will "tolerate ester and amide groups at the other end of carbon chains of alcohols (6d and 6e)." Does the catalyst system tolerate this functionality or does it require it? What product is formed if 1-octanol is employed under the same reaction conditions?

Why does this system oxidize linear aliphatic alcohols to unsaturated diene- and polyene-containing aldehyde systems instead of stopping after oxidation to the aldehyde? From the timecourse shown in Figure 5, it can not simply be a result of how long the reaction is run. Is there a difference in conditions and/or mechanism between this system and those previously reported for alcohol oxidation? Or, in other terms, how can a researcher who might want to use this method predict which product will form or the extent of oxidation/dehydrogenation that will occur?

2. The synthetic utility of the system would also be improved with a few competition studies. In order to apply this reaction to complex systems, it would be important to understand the relative reactivities of various substituents. For example, is the dehydrogenation of N-heterocycles faster or slower than that of alcohols?

3. The product yields are often low, with many below 60% including the two reactions intended to highlight the synthetic utility by synthesis of natural products (resulting in 53% yield and 26% yield, in Figure 4). Are the yields low because there are insufficient oxidizing equivalents (see comments below)? Do added equivalents of TEMPO result in higher yields? Can the reaction be run with O2 as the terminal oxidant instead?

4. On page 7, line 145, it is noted that α-substituents do not interfere with the dehydrogenation process. The only α-substitutes included are phenyl and cyclohexyl. Is this because other alkyl groups did not show similar reactivity or selectivity? One or two additional examples here might strengthen the work.

5. On page 8, line 171, it's not clear why LiOAc is needed for the dehydrogenation of N-heterocycles. Do the authors have insight here? Does the dehydrogenation of these substrates proceed through a different pathway? In addition, Figure 3e, shows LiOAc but it is missing from the general conditions on page 23.

Mechanistic Insights

In the abstract and introduction, the authors refer to the mechanistic studies that support the intermediacy of "an unusual radical intermediate stabilized by 5- or 7- or 9-center π-system." There seem to be only two mechanistic experiments conducted (TEMPO trapping and a reaction timecourse), neither of which confirms the involvement of such an intermediate. On page 10, the authors note that their reaction proceeds through "a stepwise process that consists of initial α,β-desaturation" to form the initial alkene product followed by an additional desaturation sequence to yield the products. Such a step-wise sequence seems very similar to the previously reported Ir- and Pd-catalyst systems and is independent of a π-stabilized radical intermediate?

The reaction appears to not be balanced. How do you achieve a four-electron oxidation (as shown in the optimization reaction of Figure 2) using only 2 equiv of TEMPO? Other products shown, such as 6b, require 8 oxidizing equivalents (!) but only 2 equiv of TEMPO are included.

Similarly, the individual mechanistic steps shown are not balanced. In Figure 5b, the authors show the conversion of 2a' to the TEMPO adduct. According to the proposed mechanism in Fig 1c, the authors suggest this step to occur via γ-C-H bond cleavage, isomerization, and homolysis of the CuII-C bond. This sequence, when balanced, should also form 1 equiv of HOAc and 1 equiv of CuI. Thus, in the absence of any additional oxidant, the maximum yield of TEMPO adduct would seem to be 10% (the loading of copper). How do the authors explain their 20% yield of this product?

The mechanism proposed in Figure 1C is unclear and has limited or no experimental support. Further mechanistic studies should be performed. In addition:
1. The first step, the conversion of A to B is not balanced and the reagents required for this step are not indicated.
2. The second step is surprising and presumably unprecedented? If precedence exists it should be discussed and referenced in the text. This step is also unbalanced as a copper(I) species should result from the proposed Cu-C bond homolysis.

The authors refer to a first-order dependence on the ketone that was measured in their related system in ref 15. Have the authors confirmed that this new reaction also shows a first order dependence? While I agree that it's likely, the conditions have changed and it appears that the kinetics may also have changed. The kinetics of the previous system reported in ref 15 showed 1st order dependencies on [ketone] and [Cu] and zero order dependence on [TEMPO]. With a zero order dependence on [TEMPO] in this current system, how is a TEMPO adduct isolated from reaction mixtures as shown in Figure 5b? Wouldn't the kinetics suggest that this intermediate rapidly eliminates to form product?

In the conversion of 1a to 2a what evidence is there for the proposed relay-type pathway instead of sequential but independent activation events to form each alkene?

Minor Comments
1. P4, L74. "reactivity" is misspelled
2. P9. L180-182. The mention of Lignarenone B should be referenced.

# Point-by-point responses to comments of reviewers

(Manuscript ID: NCOMMS-17-15772, By Shang et.al)

## 1. Responses to comments of Reviewer #1

***Comment #1***. *This is an impressive work from Su and coworkers outlining a protocol for the successive dehydrogenation of various saturated organic substrates. While sequential dehydrogenation using other systems is known (e.g. ketone dehydrogenation by palladium catalyst), this work clearly demonstrated the strength of the Cu(II)/TEMPO system. The key to the successive dehydrogenation and the importance of this work lies in the second and following steps: the activation of significantly less acidic gamma- and farther C-H bond, which the Cu(II)/TEMPO system is shown to be capable of. From synthetic viewpoints, the successive dehydrogenation of long-chain linear alcohol is of great potential for the synthesis of important natural products or bioactive compounds. Thus, it is recommended that this manuscript publish in Nat. Communications after the following minor issues are addressed.*

***Response:*** We appreciate the reviewer for his/her positive comments on our work and suggestions and questions that are helpful for us to improve our work. The following is the point-by-point replies to the reviewer's comments listed in his/her report.

***Comment #2.*** *The mechanistic proposal part needs to be further elaborated (Figure 1C). As the acidity of the gamma-C-H bond in enone is much less than the alpha-C-H bond of ketone (it would be better for the authors to display some data to make this clear), the transformation of B to C can actually be viewed as allylic C-H activation. While such a process is well known for palladium complex (e.g. palladium acetate), examples using copper catalyst are rare. It would be better if the authors could offer some literature supports and insights into this step. For example, is it proceeding through simple deprotonation or a radical process? The authors are also suggested to show the regeneration of Cu catalyst in the scheme for clarity. Also, the authors proposed that copper salt could also act as Lewis acid to polarize the carbonyl. This point also needs to be clarified. Is the deprotonation process involving two Cu species, one polarizing the carbonyl and the other deprotonate the C-H bond? If only one Cu catalyst is involved, what's the likely transition state for the step?*

***Response:*** To address the reviewer's concern with mechanistic proposal, we have conducted further experiments and literature survey.

In our previous studies of the mechanism of alpha, beta-dehydrogenative desaturation of saturated ketones (*J. Am. Chem. Soc.* **2016**, *138*, 5623), we observed that the rate-determining step is cleavage of alpha-C-H bond. The cleavage of such acidic alpha-C-H bond would occur via deprotonation to form Cu-enolate intermediate. Currently, no pKa value is available to assess acidity of the gamma-C-H bond in enone. However, a Cu-mediated gamma-C-H alkylation reaction of alpha, beta-unsaturated esters with allylic bromides as alkylating reagents, has been proposed to involve formation of copper dienolate intermediate via deprotonation at the gamma-position of alpha, beta-unsaturated ester (*J. Am. Chem.* Soc., **1974**, *96*, 5662), which is consistent with the copper ketone dienolate intermediate proposed by us for the successive

dehydrogenation of saturated ketones. *This work has been cited as ref. 26 in the revised manuscript, in response to reviewer's comment.*

Electron-withdrawing carbonyl group definitely reduce the electron density at the beta carbon of enone through pi-conjugated system, meanwhile, coordination of carbonyl group to Lewis acidic copper ion leads to decrease in electron density of oxygen of carbonyl group and therefore strengthens the electron-withdrawing ability of carbonyl group. As a result, carbonyl group, in combination with Lewis acidic copper catalyst, reduces the electron density at the beta carbon of enone so as to increase the acidity of adjacent gamma-C-H bond in enone. Accordingly, we speculate that the Cu-catalyzed successive dehydrogenation goes through deprotonation at gamma-position of enone to form copper dienolate intermediate. *In response to the reviewer's comment, we have revised the discussion that copper salt acts as a Lewis acid to further polarize carbonyl group and therefore strengthen the electron-withdrawing ability of carbonyl group.*

During the manuscript revision, we have determined the kinetic dependence of the enone gamma, delta-dehydrogenative desaturation on the concentration of catalyst $Cu(OAc)_2$/1,10-phenanthroline. This investigation showed that the initial rate for the enone gamma, delta-dehydrogenative desaturation is a first-order dependence on the concentration of catalyst $Cu(OAc)_2$/1,10-phenanthroline, suggesting that the enone deprotonation to copper dienolate intermediate may involve only one copper species. The transition state for this step might be the interaction between the gamma-C-H bond in the enone coordinating to Cu center and the OAc anion that is released by ligand substitution of $Cu(OAc)_2$ (please see Figure 1C in the revised manuscript). *The result of this kinetic study has been added to the "Kinetic Study part" in the Supplementary Information.*

Our previous studies have provided strong evidences to support that the alpha, beta-dehydrogenative desaturation of saturated ketones involves alpha-carbonyl radical intermediate (*J. Am. Chem. Soc.* 2016, *138*, 5623). To identify whether successive dehydrogenation to diene and polyene also involves radical intermediates, *radical clock experiments have been performed using gamma-cyclopropyl enone as a probe.* gamma-*cyclopropyl enone was found to undergo ring opening at 80 $^{o}$C under otherwise identical conditions to successive dehydrogenation to generate trienone in 32% yield and dienone with terminal TEMPO substituent in 10% yield (see 5d in Figure 5 of the revised manuscript), indicating that dehydrogenation of enone to dienecarbonyl occurs via gamma-enone radical.*

When investigating the Cu-catalyzed successive dehydrogenation of 1,5-diphenylpentan-1-one, we observed that the reaction produced a mixture of enone and conjugated diene ketone at the early stage of reaction, and gave only diene ketone product after 48 hours. In addition, alpha, beta-unsaturated carbonyl compounds proved to undergo dehydrogenation to form diene carbonyls, for example, 4c in Figure 3 of manuscript. These observations indicate that the successive dehydrogenation is likely initiated by alpha, beta-dehydrogenation, followed by remoter gamma, delta-dehydrogenation. In other words, the initial alpha, beta-dehydrogenation induces remoter dehydrogenation, constituting a successive dehydrogenation process. Since each dehydrogenative desaturation step involve radical intermediate, the successive dehydrogenation of saturated carbonyls proceeds via formation of two or more than two radical intermediates.

On the basis of the above discussion and experiments, both deprotonation and radical

formation steps are likely involved in the Cu-catalyzed successive dehydrogenative desaturation, in which radical generation step is subsequent to the deprotonation step. *The result of radical clock experiment has been added to the revised manuscript*.

Oxidation of the catalyst Cu(I) species to regenerate Cu(II) species have been showed in the proposed mechanism (Figure 1C). Our previous investigations revealed that both TEMPO and TEMPOH were able to oxidize Cu(I) complex with bipyridine ligand. In the Cu-catalyzed enone dehydrogenation reaction, we have also observed 2,2,6,6-tetramethylpiperidine that was generated from reduction of TEMPOH, which indirectly proved that oxidation of Cu(I) species by TEMPOH also occurs in this reaction.

As reviewer suggested, we have performed more experiments to obtain insights into successive dehydrogenation mechanism. Hopefully, these newly obtained data, in conjunction with literature reports, could offer a clearer picture for reaction mechanism and therefore address reviewer’s concerns.

***Comment #3.*** *(b) It is known that palladium-catalyzed dehydrogenation can access phenol from cyclohexanone. For the substrate scope of this Cu/TEMPO system, does cyclic ketone work? Also, what’s role of TsOH in the dehydrogenation of alcohols, and LiOAc in the dehydrogenation of N-heterocycles. The authors need to explain these in the manuscript.*

***Response**:* We have checked the reaction of cyclohexanone under standard conditions and found that cyclohexanone underwent dehydrogenation to produce a mixture of cyclohexenone (68% yield) and phenol (16% yield) with 11% of cyclohexanone recovered. This result have been added to the revised manuscript (Figure 1a).

TsOH and LiOAc as additives improved the yields of alcohol and N-heterocycle dehydrogenation reactions, respectively. Currently, the exact roles of them in respective reactions are unclear. TsOH additive may facilitate dissociation of strongly coordinating OAc anion from copper center and therefore enable weakly coordinating alcohol to ligate copper center to generate the Cu(II)-alkoxide species that is a key intermediate in the Cu(II)/TEMPO-promoted alcohol oxidation. LiOAc may act as a base for deprotonation of N-H and C-H bonds, which is required in N-heterocycle dehydrogenation to improve the yields of targeted products. Similarly to the Cu/TEMPO catalyzed aerobic oxidation of alcohols to carbonyl compounds in which bases were required or in-situ reduction of dioxygen generated hydroxyl anion, N-heterocycles may undergo dehydrogenation to form C-N double bonds with LiOAc as a base for accepting protons released from N-H bond. In the revised manuscript, we explained the roles of these two additives in the reactions.

***Comment #4.*** *(c) In figure 1b, it is confusing to use the structures of the right corner at the bottom to indicate quinoline and indole. It is simply better to show structures of both of them.*

***Response:*** We have revised Figure 1b, and added indole structure.

***Comment #5.*** *(d) The optimization part needs to be expanded (figure 2). For example, it would be better to show more information about the screening, such as the byproduct (if any) of the*

*reaction with low yields. Does the electronic property of the ligand affect the reaction outcome (i.e. phenanthroline with electron-donating or -withdrawing substituent)?*

***Response:*** Figure 2 that shows the results of optimization studies have been expanded to include substituent effect of phenanthroline. Information about conversion, byproduct and complete ligand screening has been added in the supplementary information.

We monitored the model reaction outcomes using GC, and found that reaction gave diene from successive dehydrogenation, and enone byproduct from uncomplete dehydrogenation. Due to mass unbalance in this reaction, we reasoned that this reaction system may produce higher molecular-weight byproducts that may form from radical-induced olefin oligomerization or polymerization. Actually, unsubstituted phenanthroline gives the best yield for the targeted product. Other phenanthroline ligands bearing electron-withdrawing or electron-donating substituents are inferior to unsubstituted phenanthroline, reflecting that the effect of ligand on the reaction outcome is complicated. The complication of ligand effect is also displayed by the fact that unsubstituted phenanthroline is the best ligand for ketone successive dehydrogenation while 5,5′-dimethoxy-2,2′-bipyridine is better than unsubstituted phenanthroline in the reactions of aldehyde, alcohol and diester.

## 2. Responses to comments of Reviewer #2

***Comment #1***. *Su and co-workers describe a desaturation reaction this is of broad interest to the readership of Nature Communications. The copper-catalyzed process allows for oxidation of numerous types of organic substrates. The main innovation to develop this method was identifying the appropriate bidentate ligand to have high levels of activity.*

*The authors determine that the products are more readily oxidized than the starting materials due to the greater level of stabilization afforded due to increased conjugation of the intermediate radical. The products are highly conjugated materials including dienes, trienes, and aromatic heterocycles. As an added bonus, the authors highlight that desaturation can accompany an aldol reaction.*

*The ease of operation is one of the nice features of this chemistry and I suspect these reaction conditions will be used for numerous other transformations both by the PI and by others working in this area. Publication is suggested.*

***Response:*** We appreciate the reviewer for his/her positive comment and encouragement on our work.

**3. Responses to comments of Reviewer #3**

***Comment #1.*** *In this manuscript Su and coworkers report a copper-catalyzed method for the multiple/sequential dehydrogenative desaturation of ketones, alcohols, and N-heterocyclic compounds. The reactivity reported is unusual and important and it fills a gap in current synthetic methodology. The work would be strengthened if a clearer understanding of the scope and mechanistic distinctions (such as the differences in product distributions between this system and related late-metal systems and Cu/TEMPO systems) were made clearer. A deeper understanding of these features would better enable the utilization of this new and interesting method. With additional scope and mechanistic understanding as described below this work may be suitable for Nature Commun.*

***Response:*** We appreciate the reviewer for his/her positive comments on our work and suggestions and questions that are helpful for us to improve our work. The following is the point-by-point replies to the reviewer's comments listed in his/her report.

***Comment #2:*** *1. In general, the synthetic utility would be bolstered by a better understanding of the factors that enable this newly discovered dehydrogenative-relay type oxidation.*

*One premise of the system is that "the polarizing effect that carbonyls exert" with the added influence of a coordinating Lewis acid, in this case the copper salt, combined with "the choice of proper ligand would lead to the optimal metal catalyst for the successive dehydrogenation." While this approach is reasonable, it begs the question: How is this catalyst system different from those used for related alcohol oxidation reactions? Both systems employ a mixture of Cu salt, TEMPO and diimine ligand, and it's unclear what features of this new system enable the successive dehydrogenation of alcohols, while very similar conditions enable the selective oxidation of the alcohol without additional dehydrogenation steps? In addition, the ligand control data shown in Figure 2 suggests that ligand is not key to achieving this reactivity, as 29% yield of the product is formed in the absence of ligand.*

***Response:*** Although both the successive dehydrogenation reaction and the related alcohol oxidation reaction employ Cu salt/diimine ligand as catalyst systems, the difference between these two reaction systems lies in the following two aspects: 1) the successive dehydrogenation reaction occurred in the absence of dioxygen using stoichiometric amount of TEMPO as oxidant, while the related alcohol oxidation reaction used dioxygen as terminal oxidant with a catalytic amount of TEMPO as co-catalyst; 2) the successive dehydrogenation reaction used non-coordinating solvent such as dichlorobenzene while the related alcohol reaction used coordinating MeCN as a solvent.

We have observed that dioxygen completely impeded the dehydrogenative desaturation of a variety of carbonyl compounds. The reason behind this observation is likely that dioxygen as a molecular biradical is able to trap the radical intermediate in successive dehydrogenative desaturation process (the experimental evidence for the radical intermediate in successive dehydrogenative desaturation has been established, please see our response to comment #9 of Reviewer #3).

Furthermore, MeCN, which was used as a solvent in the related alcohol oxidation, also impeded the successive dehydrogenative desaturation probably because strongly coordinating MeCN inhibits coordination of weakly coordinating carbonyl substrates to Cu catalyst center. In contrast, the Cu/diamine/TEMPO-catalyzed aerobic oxidation of alcohol involved Cu-hydroxyl species that could react with alcohol to generate Cu-alkoxide species and allow alcohol oxidation to occur (*J. Am. Chem. Soc.* **2013**, *135*, 2357).

Indeed, diimine ligand is not essential for the successive dehydrogenation reaction. However, diimine ligand may facilitate oxidation of Cu(I) to Cu(II) and accelerate reaction, as showed the Cu catalyst system for alcohol oxidation (*J. Am. Chem. Soc.* **2013**, *135*, 15742).

***Comment #3.*** *The authors highlight the ability of this system to enable the successive dehydrogenation of alcohols following their oxidation to aldehydes, and note that the system will "tolerate ester and amide groups at the other end of carbon chains of alcohols (6d and 6e)." Does the catalyst system tolerate this functionality or does it require it? What product is formed if 1-octanol is employed under the same reaction conditions?*

***Response:*** We have checked the successive dehydrogenation reaction of 1-octanol under standard conditions and found that 1-octanol underwent successive dehydrogenation to form a mixture of diene aldehyde, alpha, beta-unsaturated aldehyde and saturated aldehyde. Indeed, we presented two alcohol substrates with functional group at the other end of carbon chain to show functional group tolerance of this reaction. These two alcohols gave the desired successive dehydrogenation products in reasonable yields rather than a mixture, in which formation of stable, conjugated products is likely the driven force for the reaction. In a sense, amide and ester as terminal groups in alcohols are required for achieving complete dehydrogenation.

***Comment #4.*** *Why does this system oxidize linear aliphatic alcohols to unsaturated diene- and polyene-containing aldehyde systems instead of stopping after oxidation to the aldehyde? From the timecourse shown in Figure 5, it can not simply be a result of how long the reaction is run. Is there a difference in conditions and/or mechanism between this system and those previously reported for alcohol oxidation? Or, in other terms, how can a researcher who might want to use this method predict which product will form or the extent of oxidation/dehydrogenation that will occur?*

***Response:*** There exists a difference in reaction conditions and mechanism between our successive dehydrogenation reaction and the previously reported alcohol oxidation: our successive dehydrogenation occurs via radical intermediate (please see below our response to comment #9 of Reviewer #3) under anaerobic conditions, while the alcohol oxidation uses dioxygen as a terminal oxidant without involvement of radical intermediate (*J. Am. Chem. Soc.* **2014**, *136*, 12166). Dioxygen, as a molecular radical, would trap radical intermediate. As observed by us, dioxygen impeded the successive dehydrogenation of carbonyl compounds. Accordingly, it is understandable that the aerobic oxidation of alcohol gave carbonyl product without further dehydrogenative desaturation.

Stahl and co-workers have disclosed that the key step for oxidation of alcohol to aldehyde or ketone involves only Cu(II) species and TEMPO, and that dioxygen acted as a terminal oxidant to

oxidize Cu(I) and TEMPOH to regenerate Cu(II) and TEMPO catalyst. Thus, our reaction conditions enabled alcohol oxidation to form carbonyl products and subsequent successive dehydrogenation. As demonstrated in Figure 3 of the manuscript, it is predictable that alcohols with suitable functionality at the other end undergo complete successive dehydrogenation to form conjugated products.

***Comment #5.*** *The synthetic utility of the system would also be improved with a few competition studies. In order to apply this reaction to complex systems, it would be important to understand the relative reactivities of various substituents. For example, is the dehydrogenation of N-heterocycles faster or slower than that of alcohols?*

***Response:*** we agree with the reviewer's opinion regarding the importance of relative reactivities of various substrates. The competition reaction between equimolar 1,2,3,4-tetrahydroquinoline and 3-phenylpropanol has been run for 8 hours with varying reaction parameters, which showed that the dehydrogenation of N-heterocycle is faster than that of alcohol presumably because N-heterocycle dehydrogenation for aromatization has lower activation barrier. These results have been added to the revised manuscript and supplementary information.

***Comment #6.*** *The product yields are often low, with many below 60% including the two reactions intended to highlight the synthetic utility by synthesis of natural products (resulting in 53% yield and 26% yield, in Figure 4). Are the yields low because there are insufficient oxidizing equivalents (see comments below)? Do added equivalents of TEMPO result in higher yields? Can the reaction be run with $O_2$ as the terminal oxidant instead?*

***Response:*** We have attempted to improve the yield of successive dehydrogenation product by increasing the amount of TEMPO. However, this attempt failed to increase the yield. We reason that the undesired side-reaction of polyene, such as radical-induced oligomerization and polymerization, may occur to form unidentified compounds, due to the well-known high reactivity of polyene.

Previously, like the reviewer, we also wondered whether the added equivalents of oxidant are insufficient for multifold dehydrogenative desaturation. In later experimental investigations, we observed that the Cu/TEMPO-mediated dehydrogenation of saturated carbonyls also generated TEMPOH and 2,2,6,6-tetramethylpiperidine. We verified experimentally that TEMPOH was generated from the TEMPO elimination step that form the double bond. And we further verified that TEMPOH is able to oxidize bipyridien-Cu(I) complex to regenerate Cu(II) catalyst, consistent with the observed formation of 2,2,6,6-tetramethylpiperidine in dehydrogenative desaturation of ketone. These experimental investigations allowed us to draw conclusion that TEMPO acts as three-electron oxidant through abstraction of hydrogen to form TEMPOH and oxidation of Cu(I) species by TEMPOH to cleave N-O bond and form 2,2,6,6-tetramethylpiperidine (*J. Am. Chem. Soc.* 2016, *138*, 5623). Accordingly, the added equivalents of TEMPO as oxidant are sufficient for reaction to go to completion.

We have tried various oxidants such as peroxides and $O_2$, and found that several oxidants

such as $O_2$ shut down the reaction. The use of catalytic amount of TEMPO for successive dehydrogenation in the presence of oxidant is under way.

***Comment #7.*** *On page 7, line 145, it is noted that α-substituents do not interfere with the dehydrogenation process. The only α-substitutes included are phenyl and cyclohexyl. Is this because other alkyl groups did not show similar reactivity or selectivity? One or two additional examples here might strengthen the work.*

***Response:*** We have tested one more aldehyde with alpha-methyl substitute and found that this aldehyde also underwent dehydrogenation. The newly obtained result has been added in the revised manuscript (product **4e** in Figure 3b).

***Comment #8.*** *On page 8, line 171, it's not clear why LiOAc is needed for the dehydrogenation of N-heterocycles. Do the authors have insight here? Does the dehydrogenation of these substrates proceed through a different pathway? In addition, Figure 3e, shows LiOAc but it is missing from the general conditions on page 23.*

***Response:*** We speculate that LiOAc may act as a base for deprotonation of N-H and C-H bonds, which is required in N-heterocycle dehydrogenation to improve the yields of the targeted products . Similarly to the Cu/TEMPO catalyzed aerobic oxidation of alcohols in which bases were required or in-situ reduction of dioxygen generated hydroxyl anion, N-heterocycles may undergo dehydrogenation to form C-N double bonds with LiOAc as a base for accepting protons released from N-H bond. After formation of C-N double bond, N-heterocycles undergo oxidative dehydrogenation for aromatization. For tetrahydroquinolines, further dehydrogenation may be similar to the gamma, delta-dehydrogenation of carbonyls. For indolines, further dehydrogenation may occur via deprotonation, Cu(II)-N bond homolysis to form N-center radical and hydrogen-abstract from TEMPOH by N-center radical to form indole, which was different from the gamma, delta-dehydrogenation of carbonyls due to the lack of delta-C-H.

Thanks to reviewer's reminding, we have added LiOAc to the general conditions on page 23.

***Comment #9.*** *In the abstract and introduction, the authors refer to the mechanistic studies that support the intermediacy of "an unusual radical intermediate stabilized by 5- or 7- or 9-center π-system." There seem to be only two mechanistic experiments conducted (TEMPO trapping and a reaction timecourse), neither of which confirms the involvement of such an intermediate. On page 10, the authors note that their reaction proceeds through "a stepwise process that consists of initial α, β-desaturation" to form the initial alkene product followed by an additional desaturation sequence to yield the products. Such a step-wise sequence seems very similar to the previously reported Ir- and Pd-catalyst systems and is independent of a π-stabilized radical intermediate?*

***Response:*** We agree with the reviewer's opinion regarding the need for evidences to support radical intermediate stabilized by by 5- or 7- or 9-center π-system.

In our previous studies, radical clock probe and other experiments proved that Cu/TEMPO-promoted alpha, beta-dehydrogenative desaturation of ketones involves formation of

alpha-carbonyl radical intermediate (*J. Am. Chem. Soc.* **2016**, *138*, 5623). *During revision of this manuscript, radical clock experiments have also been performed using gamma-cyclopropyl enone as a probe. gamma-cyclopropyl enone was found to undergo ring opening at 80 $^{o}$C under conditions similar to successive dehydrogenation to generate trienone in 32% yield and dienone with terminal TEMPO substituent in 10% yield* (see 5d in Figure 5 of the revised manuscript), indicating that dehydrogenation of enone to dienecarbonyl occurs via gamma-enone radical.

When investigating the Cu-catalyzed successive dehydrogenation of 1,5-diphenylpentan-1-one, we observed that the reaction produced a mixture of enone and conjugated diene ketone at the early stage of reaction, and gave only diene ketone product after 48 hours. In addition, alpha, beta-unsaturated carbonyl compounds proved to undergo dehydrogenation to form diene carbonyls, for example, 4c in Figure 3 of manuscript. These observations indicate that the successive dehydrogenation is likely initiated by alpha, beta-dehydrogenation, followed by remoter gamma, delta-dehydrogenation. In other words, the initial alpha, beta-dehydrogenation induces remoter dehydrogenation, constituting a successive dehydrogenation process. Since each dehydrogenative desaturation step involve radical intermediate, the successive dehydrogenation of saturated carbonyls proceeds via formation of two or more than two radical intermediates. These radical intermediates, for example, gamma-enone radical, would conjugate with adjacent π-systems such as enone, dienone, and trienone to form delocalized radicals.

In the previously reported Ir- and Pd-catalyst systems, the dehydrogenation processes proceed through β-H elimination, which involve no radical intermediate formation. In the case of Pd-catalyzed successive dehydrogenation of cyclohexanone, the reaction was proposed to involve dehydrogenative desaturation at two identical alpha, beta-positions, and subsequent tautomerization, in which two dehydrogenative steps are mutually independent. In Ir-catalyzed successive dehydrogenation of alkane to benzene, the first dehydrogenation to form olefin should activate adjacent C-H bond, therefore facilitate the subsequent dehydrogenation step. As such, in some extent, our Cu/TEMPO is similar to the Ir-catalyzed successive dehydrogenation, since in our Cu-catalyzed successive dehydrogenation, the initial alpha, beta-dehydrogenation induces subsequent gamma, delta-dehydrogenation and remoter dehydrogenation. However, Cu-catalyzed successive dehydrogenation involves radical intermediate while the Ir-catalyzed successive dehydrogenation does not involve radical intermediate.

***Comment #10.*** *The reaction appears to not be balanced. How do you achieve a four-electron oxidation (as shown in the optimization reaction of Figure 2) using only 2 equiv of TEMPO? Other products shown, such as 6b, require 8 oxidizing equivalents (!) but only 2 equiv of TEMPO are included.*

*Similarly, the individual mechanistic steps shown are not balanced. In Figure 5b, the authors show the conversion of 2a' to the TEMPO adduct. According to the proposed mechanism in Fig 1c, the authors suggest this step to occur via gamma-C-H bond cleavage, isomerization, and homolysis of the CuII-C bond. This sequence, when balanced, should also form 1 equiv of HOAc and 1 equiv of CuI. Thus, in the absence of any additional oxidant, the maximum yield of TEMPO adduct would seem to be 10% (the loading of copper). How do the authors explain their 20% yield of this product?*

***Response:*** As above described in the response to comment #6 of this reviewer, the Cu/TEMPO-mediated dehydrogenation of saturated carbonyls generated TEMPOH and 2,2,6,6-tetramethylpiperidine. since TEMPOH is able to oxidize bipyridien-Cu(I) complex to regenerate Cu(II) catalyst with the formation of 2,2,6,6-tetramethylpiperidine, TEMPO could act as three-electron oxidant through abstraction of hydrogen to form TEMPOH and oxidation of Cu(I) species by TEMPOH to cleave N-O bond and form 2,2,6,6-tetramethylpiperidine (*J. Am. Chem. Soc.* **2016**, ***138*****,** 5623). Consequently, 2 equiv TEMPO should be sufficient for the optimization reaction of Figure 2 to go to completion (four-electron oxidation).

For the product 6b, the conditions illustrated in footnote are the general conditions. Since the required amount of oxidant varies according to different substrates, the specific reaction conditions for each substrate are listed in the Supplementary Information. In our experience, one equivalent of TEMPO is suitable for each double-bond formation. In view of this, 6b requires 4 equivalents of TEMPO. (please see Figure 4b and the experimental details for 6b product in the Supplementary Information).

Since TEMPO may act as three-electron oxidant, the 20% yield of the TEMPO adduct in Figure 5b can be explained.

***Comment #11.*** *The mechanism proposed in Figure 1C is unclear and has limited or no experimental support. Further mechanistic studies should be performed. In addition:*
*1. The first step, the conversion of A to B is not balanced and the reagents required for this step are not indicated.*
*2. The second step is surprising and presumably unprecedented? If precedence exists it should be discussed and referenced in the text. This step is also unbalanced as a copper(I) species should result from the proposed Cu-C bond homolysis.*

***Response:*** The mechanism picture in Figure 1c has been re-drawn for a clearer demonstration. The evidence for radical intermediate has been added (please see 5d in Figure 5 of the revised manuscript).

As mentioned in reply to comment #2 of reviewer 1, a copper dienolate intermediate was proposed to form via deprotonation at the gamma-position of alpha, beta-unsaturated ester (*J. Am. Chem.* Soc., 1974, *96*, 5662), which is consistent with the copper ketone dienolate intermediate proposed by us for the successive dehydrogenation of saturated ketones. *This work has been cited as ref. 26 in the revised manuscript, in response to reviewer's comment.*

To the best of our knowledge, the process of generating gamma-radical intermediate and subsequent dehydrogenation is unprecedented. I*n the revised manuscript, the scheme of gamma, delta-dehydrogenation was re-drawn as a representative for the successive dehydrogenation. The regeneration of Cu catalyst has also been shown and balanced in the scheme for better clarity.*

***Comment #12.*** *The authors refer to a first-order dependence on the ketone that was measured in their related system in ref 15. Have the authors confirmed that this new reaction also shows a first order dependence? While I agree that it's likely, the conditions have changed and it appears that the kinetics may also have changed. The kinetics of the previous system reported in*

*ref 15 showed 1st order dependencies on [ketone] and [Cu] and zero order dependence on [TEMPO]. With a zero order dependence on [TEMPO] in this current system, how is a TEMPO adduct isolated from reaction mixtures as shown in Figure 5b? Wouldn't the kinetics suggest that this intermediate rapidly eliminates to form product?*

***Response:*** In current system, the gamma, delta-dehydrogenation shows a first order dependence on [ketone] and [Cu]. The dependence on [TEMPO] is complicated and hard to explain at present stage, which shows a first order dependence on low [TEMPO] and a zero order dependence on high [TEMPO]. The related results have been added to "Kinetic Study part" in the Supplementary Information.

The kinetics of our previously reported alpha, beta-dehydrogenation showed a zero order dependence on [TEMPO] and 1st order dependency on [Cu/bpy]. Consequently, we obtained the alpha-TEMPO adduct by using stoichiometric copper salt and bipyridine instead of catalytic conditions (ref. 15). In current system, the TEMPO adduct shown in Figure 5b cannot be isolated at 120 $^{o}$C due to fast elimination but could be obtained in 80 $^{o}$C, which is much lower than the required temperature for the successive dehydrogenation (120 $^{o}$C). As a matter of fact, the initial alpha, beta-dehydrogenation requires higher temperature (120 $^{o}$C). But for the gamma, delta-dehydrogenation, lower temperature (80 $^{o}$C) is operable. This result may attributed to the more delocalized π-system in gamma-radical intermediate when compared to the corresponding alpha-radical intermediate. Additionally, in reactions of isolating gamma-TEMPO adduct, the ratio of TEMPO to enone is low (1:1), which should be in the first order dependence range according to kinetic studies (Please see "Kinetic Study part" in the Supplementary Information. However, in the successive dehydrogenation of saturated ketone to dienone, the alpha, beta-dehydrogenated enone is just an intermediate. In such case TEMPO is in large excess to enone.). Consequently, TEMPO adduct shown in Figure 5b could be isolated at a relatively mild condition (80 $^{o}$C). Furthermore, in the radical clock experiment, a dienone product with terminal TEMPO substituent could also be isolated in 10% yield (Figure 5d).

***Comment #13.*** *In the conversion of **1a** to **2a** what evidence is there for the proposed relay-type pathway instead of sequential but independent activation events to form each alkene?*

***Response:*** In the conversion of **1a** to **2a,** we observed that the reaction produced a mixture of enone and conjugated diene ketone at the early stage of reaction, and gave only diene ketone product after 48 hours. In addition, α, β-unsaturated carbonyl compounds proved to undergo dehydrogenation to form diene carbonyls, for example, 4c in Figure 3 of manuscript. These observations indicate that the successive dehydrogenation is likely initiated by α, β-dehydrogenation, followed by remoter γ, δ-dehydrogenation. In other words, the initial α, β-dehydrogenation induces remoter dehydrogenation, constituting a successive dehydrogenation process. In this context, the formation of first alkene is the basis for the second dehydrogenation.

***Comment #15.*** *Minor Comments*

1. *P4, L74. "reactivity" is misspelled;*
2. *P9. L180-182. The mention of Lignarenone B should be referenced.*

***Response:*** The misspelled word “reactivity” has been corrected and the paper about Lignarenone B has been cited as reference 38 in the revised manuscript.

Reviewer #1 (Remarks to the Author):

The reviewer appreciated the authors' efforts to address the comments and believed the revised manuscript offers a clearer better understanding of this interesting transformation. Publication in Nat. Comm. is recommended.

Reviewer #3 (Remarks to the Author):

In their revised version of the manuscript "Dehydrogenative Desaturation-Relay via Formation of 2 Multicenter-Stabilized Radical Intermediates" and in their response to the reviews, Su and coworkers have thoroughly addressed the reviewers comments and concerns. This work is synthetically important and raises many interesting mechanistic questions and in its revised form this manuscript is now suitable for publication in Nature Communications.
